# Supplementary material for: Sex differences and risk factors for bleeding in Alagille syndrome
Source: EMBO Mol Med. 2022 Nov 8;14(12):e15809. doi: 10.15252/emmm.202215809 (PMC9728057; doi:10.15252/emmm.202215809)

**EMM-2022-15809 Appendix**

**Table of Contents**

# Contents


Appendix Figure S1 ........ 2
Appendix Figure S2 ........ 3
Appendix Figure S3 ........ 4
Appendix Table S1 ........ 5
Appendix Table S2 ........ 22
Appendix Supplementary Materials and Methods ........ 27
Systematic review search strategy ........ 27
Prisma 2020 Checklist ........ 30
Prisma 2020 Flow Diagram ........ 33

## Appendix Figure S1

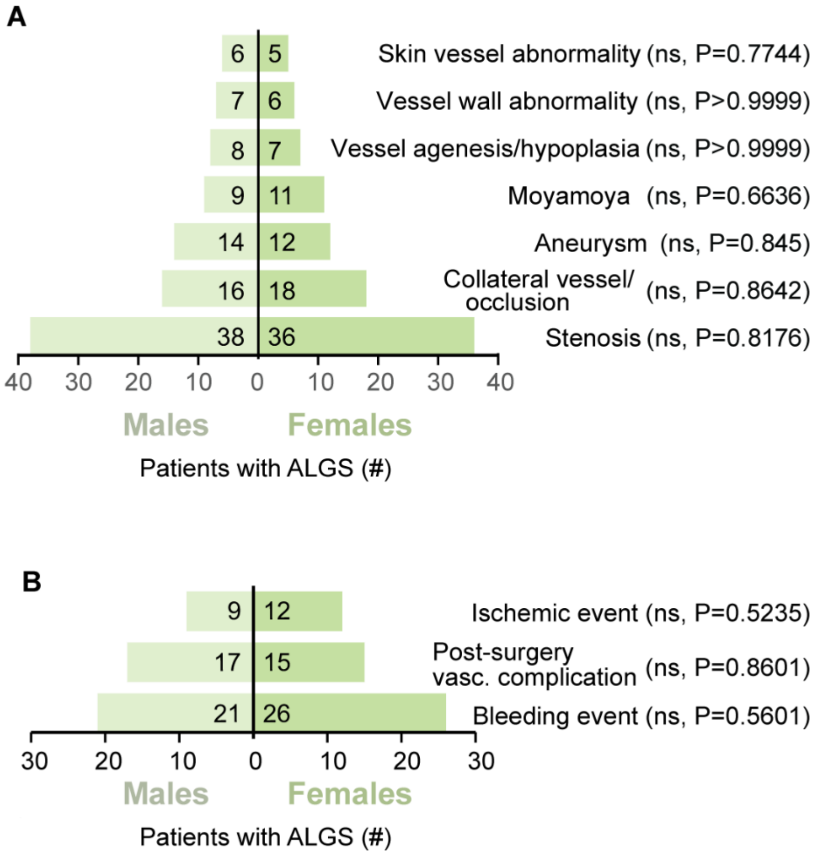


**Appendix Figure S1. Summary of most common events identified in Systematic Review, with ALGS, vascular anomalies or bleeding and sex data reported.** (A) Structural vascular abnormalities grouped by prevalence in male or female patients with ALGS. Differences between males and females are not significant (Binomial exact test of observed vs expected 50%/50%, two-sided for conditions n≥5. Overall number male vs females: 95 vs 81 ns P= 0.3271. Skin vessel abnormalities P=0.7744, vessel wall abnormalities P=>0.9999, vessel agenesis /hypoplasia P>0.9999, Moyamoya P=0.5235, Aneurysms P>0.9999, Collateral vessels occlusions P=0.8679, Stenoses P=0.8199. Please note that vascular bruits may include pulmonary stenosis, and Moyamoya includes stenosis and collateral vessels. (B) Functional vascular abnormalities grouped by prevalence in male or female patients with ALGS. All differences not significant. (Binomial exact test of observed vs expected 50%/50%, two-sided for conditions n≥5. Ischemic events p=0.8388, Surgery-related vascular complications P=0.8601, Bleeding events (not including surgery-related) P=0.8776). The numbers inside the graph bars in A and B depict the number of individuals reported with a given anomaly per sex. Some patients had multiple vascular events and are included in several categories. For references and full details, see also Source data for Figure 1.

# Appendix Figure S2

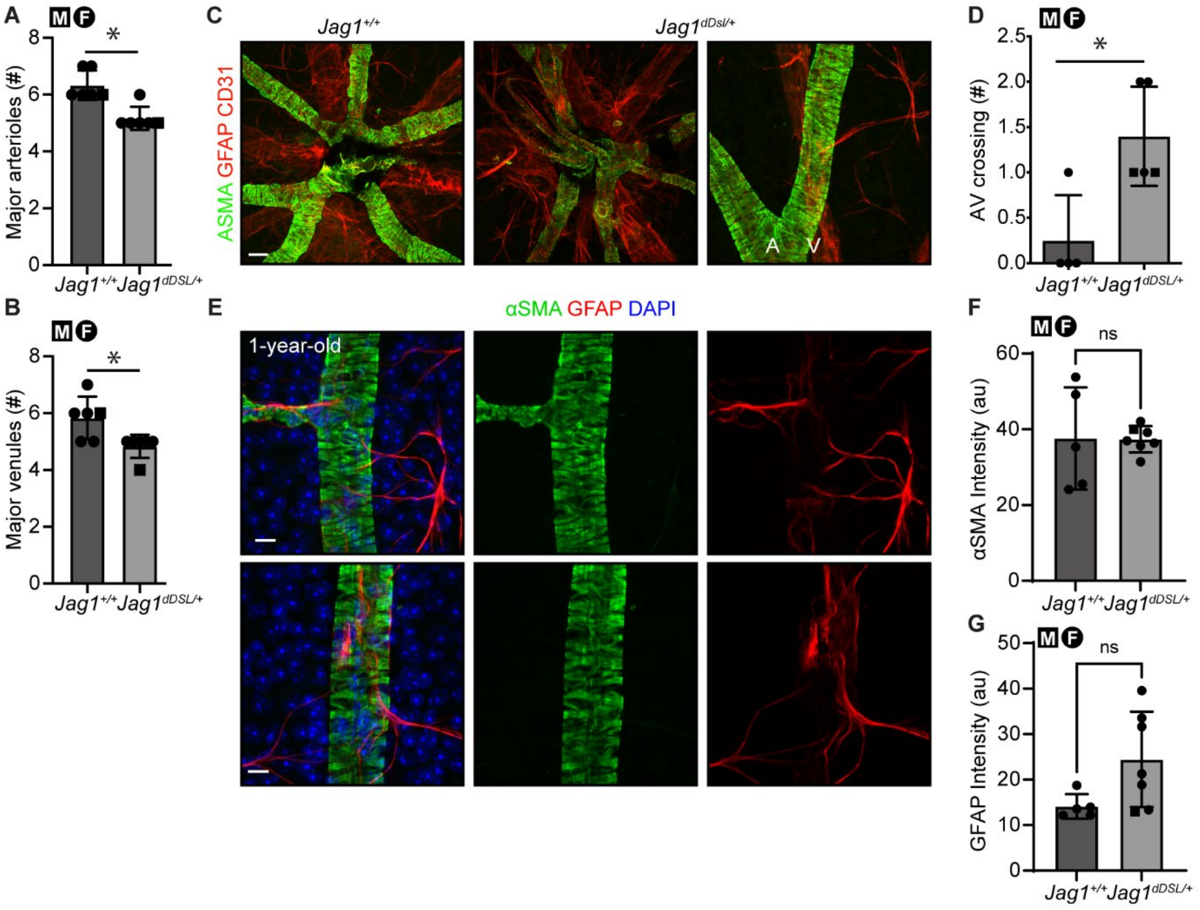


**Appendix Fig S2. *Jag1$^{\Delta Dsl/+}$* mice display a mild vascular phenotype.** (A,B) Quantification of arterioles (A) and venules (B) in control and *Jag1$^{\Delta Dsl/+}$* mice at 1 year of age (Mann-Whitney test, Arterioles P=0.0108, Venules P=0.0455). (C,D) Staining for αSMA-positive arterioles and GFAP/CD31-positive venules (C), and quantification of artery-vein crossings at all stages investigated (Mann-Whitney test, P=0.0317) (D). (E) Immunofluorescence staining for αSMA and GFAP in 1-year-old control and *Jag1$^{\Delta Dsl/+}$* retinas. (F,G) Quantification of αSMA (Mann-Whitney test, P=0.7551) (D) and GFAP (Mann-Whitney test, P=0.0732) (E) intensity per area. au= arbitrary units.

# Appendix Figure S3

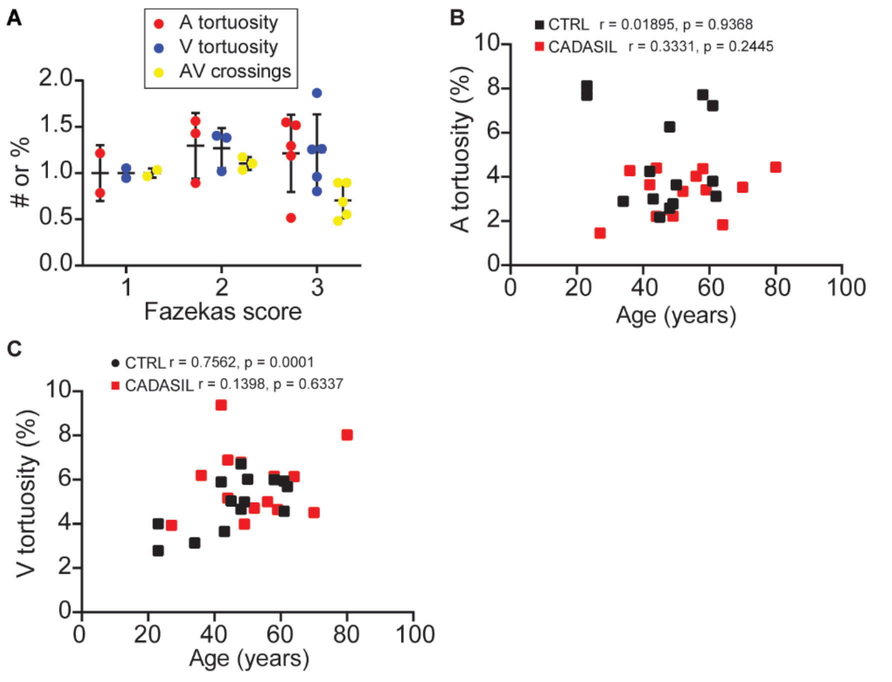


**Appendix Figure S3. CADASIL vessel tortuosity correlation analyses.** (A) Lack of correlation for arteriolar and venous tortuosity and arteriovenous crossing to Fazekas scores for CADASIL and control patients. The data for each parameter are normalized to the average value of each parameter corresponding to Fazekas score 1 value. Analyzed by 2-way ANOVA, Interaction P=0.3818, Fazekas score P=0.4829, Phenotype P=0.1373, n=10. (B) Correlation analysis between arterial tortuosity and age for CADASIL and age-matched control patients, n=14 per group, analyzed by Pearson r. (C) Correlation analysis between venous tortuosity and age for CADASIL and age matched control patients, n=14 per group, analyzed by Pearson r. Each dot represents one individual; graphs depict mean values ± standard deviation. A, arteriole; AV, arteriovenous; CADASIL, cerebral autosomal dominant arteriopathy with subcortical infarcts and leukoencephalopathy; V, venule

# Appendix Table S1

**Appendix Table S1. Systematic literature review.** Table describes patients' sex, structural anomaly or functional event. F, female; M, male; MAPCA, major aortopulmonary collateral arteries. Please note that all cases of Moyamoya are also described as having collaterals and stenosis. "Stenosis / coarctation" includes mid-aortic syndrome. Ischemic events include embolism, stroke, thrombosis and inadequate circulation.

| Patient number | Sex | Stenosis/ coarctation | Hemorrhages/ bleeding | Hematoma | Ischemic event | Collateral vessel/ occlusion | Moyamoya | Aneurysm | Vessel agenesis / hypoplasia | Vessel wall abnormality | Skin blood vessel abnormalities | Post-surgery complications | Ref | Additional notes |
|---|---|---|---|---|---|---|---|---|---|---|---|---|---|---|
| 1 | F | Y | | | | | | | | | | Y | 1 | |
| 2 | F | | Y | | | | | | | | | | 2 | |
| 3 | F | | Y | | | | | | | | | | 2 | |
| 4 | F | | Y | | | | | | | | | | 2 | |
| 5 | F | | Y | | | | | | | | | | 2 | |
| 6 | F | | Y | | | | | | | | | | 2 | |
| 7 | F | | Y | | | | | | | | | | 3 | |
| 8 | F | | | | | | | | | | | Y | 4 | Nephrotic syndrome 13 years after liver transplant- Arteriolar hyalinosis. |
| 9 | F | | | | | | | | | | | Y | 5 | Female died post-surgery due to profound vasodilation, unresponsive to vasopressin, norepinephrine, and oxygen. |
| 10 | F | Y | | | Y | Y | Y | | | | | | 6,7 | Baird et al Patient 1 = Emerick et al Patient 25 |
| 11 | F | Y | Y | | Y | Y | Y | | | | | | 7 | Baird et al Patient 5 |
| 12 | F | | | | | | | | | | | Y | 8 | Evans syndrome and arterial thrombosis after liver transplant. |
| 13 | F | | | | Y | | | | | | | | 9 | |
| 14 | F | | | | | | | | | | | | 10 | Persistent falcine sinus. |
| 15 | F | | | | | | | | | | | | 10 | Developmental venous anomaly. |
| 16 | F | | | | | Y | | | | | | | 11 | MAPCAs. |
| 17 | F | | | | | | | | | | | | 12 | Tortuous vessels in retina and retinopathy. |

| | | | | | | | | | | | | | | |
|---|---|---|---|---|---|---|---|---|---|---|---|---|---|---|
| 18 | F | | Y | | | | | | | Y | | | 13 | |
| 19 | F | | | | Y | | | | | | | | 14 | Deep vein thrombosis after valvuloplasty for pulmonary stenosis. Normal clotting, treated with anticoagulants. |
| 20 | F | | Y | | | | | | | | | | 6 | Emerick et al patient #2 |
| 21 | F | | Y | | | | | Y | | | | | 6 | Emerick et al patient #5 |
| 22 | F | | | | | | | Y | | | | | 6 | Emerick et al patient #6 |
| 23 | F | Y | | | Y | | | | | | | | 6 | Emerick et al patient #8 |
| 24 | F | | | | Y | | | | | | | | 6 | Emerick et al patient #1 |
| 25 | F | | | | | | | | | | | Y | 15 | |
| 26 | F | Y | | | | | | | | | | | 16 | Hypotonia requiring dopamine. |
| 27 | F | Y | | | | | | | | | | Y | 17 | |
| 28 | F | | Y | Y | | | | | | | | | 18 | |
| 29 | F | | | | | | | | Y | | | | 19 | |
| 30 | F | | Y | | | | | | | | | | 20 | |
| 31 | F | Y | Y | | | Y | Y | Y | | | | | 21 | |
| 32 | F | | | | | | | | | | | Y | 22 | |
| 33 | F | Y | | | | | | | | | | | 23 | |
| 34 | F | | Y | | | | | | | Y | | | 24 | |
| 35 | F | Y | | | | | | | | | | | 25 | Takayashi arteritis |
| 36 | F | | | | | | | Y | | | | | 25 | Arteriosclerosis |
| 37 | F | Y | | | | | | | | | | | 26 | |
| 38 | F | Y | | | Y | Y | Y | | | | | | 27 | Article in Korean, figure legends in English. |
| 39 | F | Y | | | Y | Y | Y | Y | | | Y | | 28 | Livedo reticularis |
| 40 | F | | Y | | | | | | | | | | 29 | |
| 41 | F | Y | | | Y | Y | | | | | | | 30 | Arteriosclerosis, MAPCAs |
| 42 | F | | | | | Y | | | | | | | 31 | MAPCAs. |
| 43 | F | | | | | Y | | | Y | | | | 32 | |

| | | | | | | | | | | | | | | |
|---|---|---|---|---|---|---|---|---|---|---|---|---|---|---|
| 44 | F | | Y | | | | | Y | | | | | 33 | |
| 45 | F | Y | | | | Y | Y | Y | Y | | | | 34 | |
| 46 | F | Y | Y | Y | | Y | Y | Y | | Y | | | 35 | |
| 47 | F | | | | | | | | | | | Y | 36 | Portal stenosis at anastomosis site 2.5 years after transplant. |
| 48 | F | Y | | | | Y | | | | | | | 37,38 | |
| 49 | F | Y | Y | | | Y | Y | | | | | | 39 | |
| 50 | F | | | | Y | | | | | | | | 40 | |
| 51 | F | | | | | | | | | | | Y | 41 | |
| 52 | F | Y | | | | Y | Y | Y | | Y | | | 42 | |
| 53 | F | Y | | | | Y | | | | | | | 43 | |
| 54 | F | Y | | | | | | | | | | Y | 44 | |
| 55 | F | Y | | | | | | | | | | | 44 | |
| 56 | F | Y | | | | | | | | | | | 44 | |
| 57 | F | Y | | | | | | | Y | | | | 45 | |
| 58 | F | | | | | | | | | | | Y | 46 | Vasogenic edema after liver transplant. |
| 59 | F | Y | Y | Y | | | | Y | | Y | | | 47 | |
| 60 | F | Y | | | | | | | | Y | | Y | 48 | Hepatic and splenic artery rupture during transplant. Unusually small abdominal aorta. Unusual arrangement of elastic fibers in hepatic artery. |
| 61 | F | Y | Y | | | | | Y | | | Y | | 49,50 | Spitting blood due to broken blood vessels in lungs. Dieulafoy lesion: large tortuous arteriole that erodes and bleeds. |
| 62 | F | Y | | | | | | | | | | | 51 | |
| 63 | F | Y | | | | | | | Y | | | | 52 | Absent subclavian artery. Arteriolar narrowing in eye. |
| 64 | F | | | | | | | | | | | Y | 53 | |
| 65 | F | | Y | Y | | | | Y | | | | | 54 | |
| 66 | F | Y | | | | | | | | | | | 55 | |
| 67 | F | Y | | | | | | | | | | | 56 | |

| | | | | | | | | | | | | | | |
|---|---|---|---|---|---|---|---|---|---|---|---|---|---|---|
| 68 | F | | | | | | | | | | | Y | 57 | Perfusion defects in lungs. Right hemiparesis due to a cerebral thromboembolism during cardiac catheterization. |
| 69 | F | | | Y | | | | | | | | | 58 | |
| 70 | F | Y | | | | | | | | | | | 59 | |
| 71 | F | | | | | | | | | | Y | | 60 | |
| 72 | F | | | | | | | | | | Y | | 60 | |
| 73 | F | Y | | | | Y | | | Y | | | | 61 | |
| 74 | F | Y | | | Y | Y | Y | | | | | | 62 | |
| 75 | F | Y | | | Y | Y | Y | | | | | | 62 | |
| 76 | F | | | | | | | | | | | Y | 63 | |
| 77 | F | Y | | | | | | | Y | | | | 64 | Abdominal aorta with 50% concentric narrowing. |
| 78 | F | Y | | | | | | | | | | | 64 | |
| 79 | F | | | | | | | | | | Y | | 65 | Double mutant NOTCH2 RASA1, AVM. type II Abernethy malformation in the hepatic portal vein |
| 80 | M | | | | | | | | | | Y | | 66 | |
| 81 | M | | Y | | | | | | | | | | 2 | |
| 82 | M | | Y | | | | | | | | | | 2 | |
| 83 | M | | Y | | | | | | | | | | 2 | |
| 84 | M | | Y | | | | | | | | | | 2 | |
| 85 | M | | Y | | | | | | | | | | 2 | |
| 86 | M | | Y | | | | | | | | | | 2 | |
| 87 | M | | Y | | | | | | | | | | 2 | |
| 88 | M | | Y | | | | | | | | | | 2 | |
| 89 | M | | Y | | | | | | | | | | 2 | |
| 90 | M | Y | | | | | | | | | | | 67 | |
| 91 | M | | | | | | | | | | | Y | 68 | |
| 92 | M | Y | | | Y | Y | Y | | | | | | 69 | |
| 93 | M | Y | | | Y | Y | Y | | | | | | 6,7 | Baird et al Patient 3 = Emerick et al Patient 20 |

| | | | | | | | | | | | | | | |
|---|---|---|---|---|---|---|---|---|---|---|---|---|---|---|
| 94 | M | Y | | | Y | Y | Y | | | | | | 6,7 | Baird et al Patient 2 = Emerick et al Patient 9 |
| 95 | M | Y | | | Y | Y | Y | | | | | | 7 | Baird et al Patient 4 |
| 96 | M | | Y | | | | | Y | | | | | 70 | |
| 97 | M | | | | | | | | | | | | 10 | Persistent falcine sinus. |
| 98 | M | Y | | | Y | Y | Y | | | | | | 10 | |
| 99 | M | | | | | | | Y | | Y | | | 10 | |
| 100 | M | | | | | | | Y | | Y | | | 10 | |
| 101 | M | | | | | | | | | Y | | | 10 | Dolichoectasia |
| 102 | M | Y | | | Y | Y | Y | | | | | | 10 | |
| 103 | M | | | | | | | Y | | Y | | | 10 | Persistent falcine sinus |
| 104 | M | Y | | | | | | | | | | | 71 | |
| 105 | M | Y | | | Y | Y | Y | | | | | | 72 | |
| 106 | M | Y | Y | | | | | Y | | | | | 73 | |
| 107 | M | | | Y | | | | | | | | | 6 | Emerick et al patient 10 |
| 108 | M | | | | | | | | | | | Y | 6,74 | Emerick et al patient 19 & single patient reported by sex in Emerick et al 1999 |
| 109 | M | Y | | | Y | | | | Y | | | | 6 | Emerick et al patient 21 |
| 110 | M | Y | | | | | | | | | | | 6 | Emerick et al patient 22 |
| 111 | M | | | | | | | | Y | | | | 6 | Emerick et al Patient 23 |
| 112 | M | Y | | | | | | | | | | | 6 | Emerick et al Patient 24 |
| 113 | M | | | | | | | | | | | Y | 15 | Mid -aortic syndrome Boy in Fig 1 |
| 114 | M | Y | | | | Y | | | | | | Y | 75 | |
| 115 | M | | | | | | | | Y | | | | 76 | Two-vessel cord (only one artery in umbilical cord- usually there are two.) Stillborn |
| 116 | M | | | | | | | | | | | Y | 22 | |

| | | | | | | | | | | | | | | |
|---|---|---|---|---|---|---|---|---|---|---|---|---|---|---|
| 117 | M | | | | | | | | | Y | | | 77 | Arterial hyalinization in kidneys (with IgA nephritis) Microhematuria |
| 118 | M | Y | | | | | | | | | | | 78 | |
| 119 | M | Y | | | | | | | | | | | 79 | |
| 120 | M | | | Y | | | | | | | | | 24 | |
| 121 | M | | | Y | | | | | | | | | 24 | |
| 122 | M | | | | | | | | | | | Y | 80 | Vascular bruits/turbulent flow. |
| 123 | M | Y | | | | Y | | Y | | | | | 81 | |
| 124 | M | | Y | Y | | | | | | Y | | | 82 | |
| 125 | M | | | | | | | | | | | Y | 83 | Arterial reconstruction. |
| 126 | M | | | | | | | | | | | Y | 83 | Surgery-related stroke. |
| 127 | M | Y | | | | | | | Y | | | | 84 | Vascular bruits/turbulent flow. |
| 128 | M | Y | | | | | | | | | | | 85 | |
| 129 | M | | | | | | | | | | | | 86 | Arteriovenous malformation, connection between hepatic artery and portal vein |
| 130 | M | | | | | | | | | | | | 87 | Perthes Disease in a patient with ALGS. |
| 131 | M | | Y | | | | | | | | | | 88 | |
| 132 | M | | | | | | | Y | | | | | 89 | |
| 133 | M | | | | | | | Y | | | | | 90 | |
| 134 | M | | Y | | | | | | | | Y | | 91 | Nosebleeds. Dilated blood vessels on abdomen (likely secondary to portal vein hypertension). |
| 135 | M | Y | | | | Y | Y | | | Y | Y | | 92–94 | Three conference abstracts, same patient. |
| 136 | M | | | | | | | | | | | Y | 95 | |
| 137 | M | | Y | | | | | | | | | | 96 | Nose bleeds and bruising. Death by major blood vomiting, but no esophageal varices. |
| 138 | M | | | | | | | | | | | Y | 36 | 4 years after transplant. Celiac origin stenosis and portosystemic collaterals. |
| 139 | M | | | Y | Y | | | | | | | | 97 | |
| 140 | M | Y | | | | | | | | | | | 98 | |

| | | | | | | | | | | | | | | |
|---|---|---|---|---|---|---|---|---|---|---|---|---|---|---|
| 141 | M | Y | | | | Y | | Y | | | | | 43 | |
| 142 | M | Y | | | | Y | | Y | Y | | | | 43 | |
| 143 | M | Y | | | | | | | | | | | 43 | |
| 144 | M | Y | | | | Y | | | | | | | 43 | |
| 145 | M | Y | | | | | | | | | | | 44 | |
| 146 | M | Y | | | | | | | | | | | 44 | |
| 147 | M | Y | | | | | | | | | | | 44 | |
| 148 | M | Y | | | | | | | | | | Y | 44 | |
| 149 | M | | | | | | | | | | | Y | 44 | |
| 150 | M | | | | | | | | | | | Y | 44 | |
| 151 | M | Y | | | | | | | Y | | | | 45 | |
| 152 | M | | Y | | | | | | | | | | 45 | |
| 153 | M | Y | | | | | | | Y | | | | 99 | Boy with anomalies as described. Father with renal failure and cerebrovascular complications - died before diagnosis - no liver defects. |
| 154 | M | Y | | | | | | Y | | | | Y | 100 | |
| 155 | M | Y | | | | | | | | | | | 51 | |
| 156 | M | Y | | | | | | | | | | | 51 | |
| 157 | M | | | | | Y | | | | | | | 101 | Systemic to pulmonary artery collateral vessels but did not resemble a true ductus arteriosus. |
| 158 | M | | | | | | | Y | | | | | 102 | |
| 159 | M | Y | | | | Y | Y | | | | | | 103 | |
| 160 | M | | | | | | | Y | | | | | 56 | |
| 161 | M | | | | | | | Y | | | | | 104 | |
| 162 | M | | | | | | | | | | Y | | 60 | |
| 163 | M | | | | | | | | | | Y | | 60 | |
| 164 | M | | | | | | | | | | Y | | 60 | |
| 165 | M | | | | | | | | | | | Y | 63 | |
| 166 | M | | | | | | | | | | | Y | 63 | |

| | | | | | | | | | | | | | | |
|---|---|---|---|---|---|---|---|---|---|---|---|---|---|---|
| 167 | M | | | | | | | | | | | Y | 63 | |
| 168 | M | Y | | | | Y | | | | | | | 105 | Vascular bruits/turbulent flow. |
| 169 | M | | | | | | | | | | | | 64 | Fragile renal arteries. |
| 170 | M | | | | | | | | | | | | 64 | Fragile renal and abdominal arteries. |
| 171 | M | Y | | | | | | | | | | | 106 | |
| 172 | M | Y | | | | | | | Y | | | | 107 | NOTCH2 mutation. Filiform endings of peripheral vessels. |
| Sum Females | | 36 | 21 | 5 | 12 | 18 | 11 | 12 | 7 | 6 | 5 | 15 | | |
| Sum Males | | 38 | 16 | 5 | 9 | 16 | 9 | 14 | 8 | 7 | 6 | 17 | | |


1. Adachi T, Murakawa M, Uetsuki N, Segawa H. Living related donor liver transplantation in a patient with severe aortic stenosis. *Br J Anaesth* 1999;**83**:488–490.

2. Adamson GT, Peng LF, Lin A, Wise-Faberowski L, McElhinney DB. Pulmonary hemorrhage during cardiac catheterization is common in children with alagille syndrome. *Catheter Cardiovasc Interv* 2019;**93**:S126–S127.

3. Agrawal S, Chennuri V, Agrawal P. Genetic Diagnosis in an Indian Child with Alagille Syndrome. *Indian J Pediatr* 2015;**82**:653–654.

4. Ayva ES, Ozdemir BH, Baskin E, Haberal M. Lupus Nephritis after liver transplantation. *Virchows Arch* 2017;**471**:S209.

5. Bacha EA, Hardin J, Cronin DC, Brady L, Millis MJ, Starr JP, Koenig P, Daves S, Kahana M. Open-heart surgery in pediatric patients with end-stage liver disease. *Ann Thorac Surg* 2004;**78**:e30–e33.

6. Emerick KM, Krantz ID, Kamath BM, Darling C, Burrowes DM, Spinner NB, Whitington PF, Piccoli DA. Intracranial vascular abnormalities in patients with Alagille syndrome. *J Pediatr Gastroenterol Nutr* 2005;**41**:99–107.

7. Baird LC, Smith ER, Ichord R, Piccoli DA, Bernard TJ, Spinner NB, Scott RM, Kamath BM. Moyamoya syndrome associated with Alagille syndrome: outcome after surgical revascularization. *J Pediatr* 2015;**166**:470–473.

8. Briem-Richter A, Grabhorn E, Ganschow R. Evans syndrome in a child after liver transplantation for Alagille syndrome. *Pediatr Transplant* 2009;**13**:112.

9. Byrne JLB, Harrod MJE, Friedman JM, Howard-Peebles PN. Del(20P) With Manifestations of Arteriohepatic Dysplasia. *Am J Med Genet* 1986;**24**:673–678.

10. Carpenter CD, Linscott LL, Leach JL, Vadivelu S, Abruzzo T. Spectrum of cerebral arterial and venous abnormalities in Alagille syndrome. *Pediatr Radiol* 2018;**48**:602–608.

11. Castro Rodriguez J, Dessy H, Demanet H. Implantation of an Absorb bioresorbable vascular scaffold in the stenotic aortopulmonary collateral artery of a young child with Alagille syndrome. *Catheter Cardiovasc Interv* 2015;**86**:E76–E80.

12. Cheema MR, Stone LG, Sellar PW, Quinn S, Clark SC, Martin RJ, O'Brien JM, Warriner C, Browning AC. Long-term follow-up of a patient with JAG1-associated retinopathy. *Doc Ophthalmol* 2021;**20**:20.

13. Doberentz E, Kuchelmeister K, Madea B. Subarachnoid hemorrhage due to aneurysm rupture in a young woman with Alagille syndrome - A rare cause of sudden death. *Leg Med (Tokyo)* Elsevier Ireland Ltd; 2015;**17**:309–312.

14. Dzumhur SM, Goss DE, Cohen AT. Low-molecular-weight heparin for venous thrombosis in a neonate. *Lancet (London, England)* 1995;**346**:1487.

15. Englert C, Grabhorn E, Burdelski M, Ganschow R. Liver transplantation in children with Alagille syndrome: Indications and outcome. *Pediatr Transplant* 2006;**10**:154–158.

16. Esther AR, Francesca C, Jose U. Neonatal hyperbilirrubinemia and prematurity as cause of green deciduous teeth. *Curr Pediatr Res* 2017;**21**:298–300.

17. Ferreira-Magalhães M, Correia-Costa L, Teixeira A, Rocha-Silva A, Pinto H, Caldas-Afonso A. Renovascular hypertension related with alagille syndrome: A rare and severe clinical case. *Pediatr Nephrol* 2014;**29**:1719.

18. Fiorda-Diaz J, Shabsigh M, Dimitrova G, Soghomonyan S, Sandhu G. Perioperative Management of Subarachnoid Hemorrhage in a Patient with Alagille Syndrome and Unrepaired Tetralogy of Fallot: Case Report. *Front Surg* 2017;**4**:72.

19. Fitzgerald RT, Zuccoli G. Agenesis of the internal carotid artery: Associated malformations including a high rate of aortic and cardiac malformations. *Pediatr Radiol* 2012;**42**:1333–1338.

20. Frongillo F, Bianco G, Silvestrini N, Lirosi MC, Sanchez AM, Nure E, Gaspari R, Avolio AW, Sganga G, Agnes S. Acute Liver Failure in

an Adult, a Rare Complication of Alagille Syndrome: Case Report and Brief Review. *Transplant Proc* 2015;**47**:2179–2181.

21. Gaba RC, Shah RP, Muskovitz AA, Guzman G, Michals EA. Synchronous moyamoya syndrome and ruptured cerebral aneurysm in Alagille syndrome. *J Clin Neurosci* 2008;**15**:1395–1398.

22. Gibelli NEM, Tannuri ACA, Andrade WC, Ricardi LRS, Tannuri U. Centrilobular necrosis as a manifestation of venous outflow block in pediatric malnourished liver transplant recipients - Case reports. *Pediatr Transplant* 2012;**16**:383–387.

23. Hirai H, Santo Y, Kogaki S, Kurotobi S, Etani Y, Mushiake S, Nakatsuchi Y, Nakajima S, Ozono K. Successful stenting for renal artery stenosis in a patient with Alagille syndrome. *Pediatr Nephrol* 2005;**20**:831–833.

24. Hoffenberg EJ, Narkewicz MR, Sondheimer JM, Smith DJ, Silverman A, Sokol RJ. Outcome of syndromic paucity of interlobular bile ducts (Alagille syndrome) with onset of cholestasis in infancy. *J Pediatr* 1995;**127**:220–224.

25. Jacquet A, Guiochon-Mantel A, Noël LH, Sqalli T, Bedossa P, Hadchouel M, Grünfeld JP, Fakhouri F. Alagille Syndrome in Adult Patients: It Is Never Too Late. *Am J Kidney Dis* 2007;**49**:705–709.

26. Kuang J, Zheng X-R, Zhang G-Y, Wang X, Liu C-T, Wu M-L, Tang Y-J. [Liver dysfunction for 8 years with hypertension for 1 week in an 8-year-old girl]. *Zhongguo Dang Dai Er Ke Za Zhi* 2019;**21**:282–286.

27. Lim MR, Lee SY, Kim DS, Kim KM, Ko TS. A Case of Moyamoya Disease in a Child with Alagille Syndrome. J. Korean Pediatr. Soc. Korean Pediatric Society; 2003 Jan.

28. Listernick R. A 6-year-old with a Complex Medical History and Recurrent Strokes. *Pediatr Ann* 2003;**32**:781–785.

29. Lykavieris P, Crosnier C, Trichet C, Meunier-Rotival M, Hadchouel M. Bleeding tendency in children with Alagille syndrome. *Pediatrics* 2003;**111**:167–170.

30. May L, Hanley FL, Connolly AJ, Reddy S. Atherosclerosis causing recurrent catastrophic aortopulmonary shunt dehiscence in a patient with alagille syndrome. *Pediatr Cardiol* 2013;**34**:1945–1948.

31. Micaglio E, Andronache AA, Carrera P, Monasky MM, Locati ET, Pirola B, Presi S, Carminati M, Ferrari M, Giamberti A, Pappone C. Novel JAG1 Deletion Variant in Patient with Atypical Alagille Syndrome. *Int J Mol Sci* 2019;**20**.

32. Moog U, Engelen J, Albrechts J, Hoorntje T, Hendrikse F, Schrander-Stumpel C. Alagille syndrome in a family with duplication 20p11. Clin. Dysmorphol. 1996. p. 279.

33. O'Connell D, Kaliaperumal C, Fanning N, Wyse G, Kaar G. Superior cerebellar aneurysm causing subarachnoid haemorrhage in a 17-year-old with alagille syndrome. *Br J Neurosurg* 2012;**26**:287–289.

34. Pavanello M, Severino M, D'Antiga L, Castellan L, Calv A, Colledan M, Gandolfo C. Pretransplant Management of Basilar Artery Aneurysm and Moyamoya Disease in a Child With Alagille Syndrome. *Liver Transplant* 2015;**21**:1227–1230.

35. Petaros A, Miletic D, Stifter S, Slaus M, Stemberga V. Alagille syndrome case report: implications for forensic pathology and anthropology. *Int J Legal Med* 2015;**129**:543–549.

36. Pimpalwar S, Annam A, Justino H, Hernandez A. Trans-splenic recanalization and angioplasty of anastomotic portal vein occlusion post OLT. *Pediatr Radiol* 2013;**43**:S382–S383.

37. Quek SC, Tan L, Quek ST, Yip W, Aw M, Quak SH. Abdominal coarctation and Alagille syndrome. *Pediatrics* 2000;**106**:E9.

38. Hia CPP, Quak SH, Quek SC. Mid aortic syndrome and Alagille syndrome. *Heart* 2004;**90**:1150 LP – 1150.

39. Rachmel A, Zeharia A, Neuman-Levin M, Weitz R, Shamir R, Dinari G. Alagille syndrome associated with Moyamoya disease. *Am J Med Genet* 1989;**33**:89–91.

40. Radi E, Formichi P, Maio G Di, Battisti C, Federico A. Oxidative stress-induced apoptosis in two patients with Alagille syndrome. *J Neurol Sci* 2011;**308**:49–56.

41. Rahmoune FC, Bruyre M, Tecsy M, Benhamou D. Alagille syndrome and pregnancy: Anesthetic management for cesarean section. *Int J Obstet Anesth* 2011;**20**:355–358.

42. Rocha R, Soro I, Leitão A, Silva ML, Leão M. Moyamoya vascular pattern in alagille syndrome. *Pediatr Neurol* 2012;**47**:125–128.

43. Salem JE, Bruguiere E, Iserin L, Guiochon-Mantel A, Plouin PF. Hypertension and aortorenal disease in Alagille syndrome. *J Hypertens* 2012;**30**:1300–1306.

44. Sanada Y, Naya I, Katano T, Hirata Y, Yamada N, Okada N, Ihara Y, Onishi Y, Mizuta K. Visceral artery anomalies in patients with

Alagille syndrome. *Pediatr Transplant* 2019;**23**:1–7.

45. Santamaria R, Lopez-Aguilera J, Gonzalez De Caldas R, Sanchez-Frias M, Garcia-Rios A, Buendia P, Aljama P. Vascular abnormalities as predominant feature in alagille syndrome patients with a novel mutation in JAG1 gene. *Journal of Hypertension* 2016.

46. Santos MM, Tannuri ACA, Gibelli NE, Ayoub AA, Maksoud-Filho JG, Andrade WC, Velhote MCP, Silva MM, Pinho ML, Miyatani HT, Susuki L, Tannuri U. Posterior reversible encephalopathy syndrome after liver transplantation in children: A rare complication related to calcineurin inhibitor effects. *Pediatr Transplant* 2011;**15**:157–160.

47. Schlosser H-G, Kerner T, Woiciechowsky C, Benndorf G. Multiple cerebral aneurysms and subarachnoid hemorrhage in a patient with Alagille syndrome. *AJNR Am J Neuroradiol* 2004;**25**:1366–1367.

48. Shehata MR, Yagi S, Ogura Y, Ogawa K, Iida T, Mori A, Sumiyoshi S, Haga H, Uemoto S. Use of recipient left colic artery for arterial reconstruction during liver transplantation in alagille syndrome with vasculopathy: A case report. *Transplantation* 2012;**93**:e20–e21.

49. Sheth HS, Maldonado F, Lentz RJ. Two cases of Dieulafoy lesions of the bronchus with novel comorbid associations and endobronchial ablative management. *Medicine (Baltimore)* 2018;**97**:e9754.

50. Lentz R, Merrick C, Rickman O, Maldonado F. Endobronchial Nd:YAP Laser Ablation of a Bleeding Dieulafoy Lesion of the Bronchus. *Chest* 2016;**150**:989A.

51. Shrivastava R, Williams A, Mikhail A, Roberts D, Richards M, Aithal V. An unusual cause of hypertension and renal failure: a case series of a family with Alagille syndrome. *Nephrol Dial Transplant* 2010;**25**:1501–1506.

52. Shulman SA, Hyams JS, Gunta R, Greenstein RM, Cassidy SB. Arteriohepatic dysplasia (Alagille syndrome): Extreme variability among affected family members. *Am J Med Genet* 1984;**19**:325–332.

53. Sugimoto K-I, Ohmori M, Fujimura A, Sakamoto K, Sudoh T, Yamauchi H, Mizuta K, Hishikawa S, Kitamura T, Kawarasaki H. Hepatic venous stenosis-related reduction of cyclosporin clearance in a pediatric patient after liver transplantation. *Ther Drug Monit* 2003;**25**:294–296.

54. Tumialán LM, Dhall SS, Tomak PR, Barrow DL. Alagille Syndrome and Aneurysmal Subarachnoid Hemorrhage. *Pediatr Neurosurg* 2006;**42**:57–61.

55. Uberos J, Moreno L, Muñoz-Hoyos A. Hypertension and Biliary Ductopenia in a Patient with Duplication of Exon 6 of the *JAG1* Gene. *Clin Med Insights Pediatr* 2012;**6**:CMPed.S9621.

56. Gucht I Van, Bento JR, Berendoncks A Van, Craenenbroeck E Van, Saenen J, Laga S, Meester J, Verstraeten A, Boeckx N, Laer L Van, Loeys B. Aortic aneurysm and dissection, an underestimated finding in JAG1 mutation carriers. *Eur J Hum Genet* 2020;**28**:257–258.

57. Villani MF, Pizzoferro M, Secinaro A, Nobili V, Chiapparelli S, Garganese MC. The "dear old" lung perfusion scan: Does it still have a role? A complex case of alagille syndrome. *Clin Transl Imaging* 2013;**1**:S132.

58. Vorstman EBA, Anslow P, Keeling DM, Haythornthwaite G, Bilolikar H, McShane MT. Brain haemorrhage in five infants with coagulopathy. *Arch Dis Child* 2003;**88**:1119–1121.

59. Voykov B, Guenova E, Sturm E, Deuter C. Alagille syndrome associated with myelinated retinal nerve fibers. *Ophthalmologica* 2009;**223**:348–350.

60. Watson GH, Miller V. Arteriohepatic dysplasia. Familial pulmonary arterial stenosis with neonatal liver disease. *Arch Dis Child* 1973;**48**:459–466.

61. Wauters A, Dejagere T, Devriendt K, Vanrenterghem Y, Maes B. Triangular face and vascular malformation. The links to renal failure. *Nephrol Dial Transplant* 2003;**18**:436–439.

62. Woolfenden AR, Albers GW, Steinberg GK, Hahn JS, Johnston DCC, Farrell K. Moyamoya Syndrome in Children With Alagille Syndrome: Additional Evidence of a Vasculopathy. *Pediatrics* 1999;**103**:505–508.

63. Yang W-H, Zhang L, Xue F-S, Riaz A, Zhu Z-J. Pediatric Liver Transplantation for Alagille Syndrome: Anesthetic Evaluation and Perioperative Management. *Ann Transplant* 2020;**25**:e924282.

64. Yucel H, Hoorntje SJ, Bravenboer B. Renal abnormalities in a family with Alagille syndrome. *Neth J Med* 2010;**68**:38–39.

65. Zheng Y, Peng Y, Zhang S, Li L, Peng Y, Yin Q. Capillary Malformation-Arteriovenous Malformation Combined Alagille Syndrome in a Patient With Double Gene Variations of RASA1 and NOTCH2. *Front Genet* 2019;**10**:1–7.

66. Adams PC. Hepatocellular carcinoma associated with arteriohepatic dysplasia. *Dig DisSci* 1986;**31**:438–442.

67. Agarwal V. Recurrent coarctation in a patient with Alagille syndrome. *Interact Cardiovasc Thorac Surg* 2005;**4**:514–516.

68. Akagi K, Tanaka T, Baba S. Successful living donor liver transplantation after stent implantation in a patient with Alagille syndrome and severe bilateral pulmonary artery stenosis. *Cardiol Young* 2018;**28**:1465–1467.

69. Amlie-Lefond C, Ellenbogen RG. Factors Associated with the Presentation of Moyamoya in Childhood. *J Stroke Cerebrovasc Dis* 2015;**24**:1204–1210.

70. Boudjemline Y, Agnoletti G, Ou P, Stos B, Villain E, Bonnet D, Sidi D. Transcatheter exclusion of a giant mycotic aneurysm of the left pulmonary artery using covered stents. *Arch Mal Coeur Vaiss* 2006;**99**:503–506.

71. Cheng K-W, Hunag J-J, Wang C-H, Chen C-L, Jawan B. Anesthetic Management of a Patient with Alagille's Syndrome Undergoing Living Donor Liver Transplantation without Blood Transfusion. *Chang Gung Med J* 2004;**27**:449–453.

72. Connor SEJ, Hewes D, Ball C, Jarosz JM. Alagille syndrome with angiographic associated moyamoya. *Childs Nerv Syst* 2002;**18**:186–190.

73. Cowan JA, Barkhoudarian G, Yang LJS, Thompson BG. Progression of a posterior communicating artery infundibulum into an aneurysm in a patient with Alagille syndrome. Case report. *J Neurosurg* 2004;**101**:694–696.

74. Emerick KM, Rand EB, Goldmuntz E, Krantz ID, Spinner NB, Piccoli DA. Features of Alagille syndrome in 92 patients: frequency and relation to prognosis. *Hepatology* W.B. Saunders; 1999;**29**:822–829.

75. Galvez D, Ruch B, Sharma A, Sydnor M, Levy M, Sharma A. Recurrent Hepatocellular Carcinoma in an Adult With Alagille Syndrome Treated With Liver Resection Followed by Liver Transplantation: A Rare Case Report. *Transplant direct* 2020;**6**:e606.

76. Ghidini A, Incerti M, Andreani M. Alagille Syndrome: Prenatal Sonographic Findings. *J Clin Ultrasound* 2006;156–158.

77. Gilboa N, Hopp L, Agostini RM. IgA nephritis in a patient with Alagille syndrome and a transplanted liver. *Pediatr Nephrol* 1992;**6**:559–561.

78. Harendza S, Hübner CA, Gläser C, Burdelski M, Thaiss F, Hansmann I, Gal A, Stahl RAK. Renal failure and hypertension in Alagille syndrome with a novel JAG1 mutation. *J Nephrol* 2005;**18**:312–317.

79. Harris M, Cao QL, Waight D, Hijazi ZM. Successful combined orthotopic liver transplant and transcatheter management of atrial septal defect, patent ductus arteriosus, and peripheral pulmonic stenosis in a small infant with Alagille syndrome. *Pediatr Cardiol* 2002;**23**:650–654.

80. Karakayali H, Boyvat F, Coskun M, Isiklar I, Sözen H, Filik L, Yilmaz U, Gür G, Haberal M. Venous complications after orthotopic liver transplantation. *Transplant Proc* 2006;**38**:604–606.

81. Kayhan A, Ilkhchoui Y, Venu N, Jensen DM, Oto A. Multiple Abdominal Vascular Anomalies in a Patient with Alagille Syndrome. *J Vasc Interv Radiol* 2010;**21**:937–940.

82. Kazi R, Fakhoury J, Beck R. A Case of Recurrent Intracranial Bleeds in the Setting of Alagille Syndrome. *Pediatrics* 2018;**142**.

83. Kohaut J, Pommier R, Guerin F, Pariente D, Jacquemin E, Martelli H, Branchereau S. Abdominal Arterial Anomalies in Children With Alagille Syndrome: Surgical Aspects and Outcomes of Liver Transplantation. *J Pediatr Gastroenterol Nutr* 2017;**64**:888–891.

84. Labrecque DR, Mitros FA, Nathan RJ, Romanchuk KG, Judisch GF, El-Khoury GH. Four Generations of Arteriohepatic Dysplasia. *Hepatology* 1982;**2**:467S-474S.

85. Levin SE, Zarvos P, Milner S, Schmaman A. Arteriohepatic dysplasia: association of liver disease with pulmonary arterial stenosis as well as facial and skeletal abnormalities. *Pediatrics* 1980;**66**:876–883.

86. Lopez RN, Rawahi Y Al, Stormon M, Chennapragada M, Ooi CY. Hepatobiliary and Pancreatic: Hepatic arterioportal fistula: A novel and treatable feature of Alagille syndrome. *J Gastroenterol Hepatol* 2018;**34**:633.

87. Massobrio M, Antonietti G, Necci F, Esposito C. Perthes-like disease in Alagille syndrome. *J Pediatr Orthop Part B* 2011;**20**:299–302.

88. Matsuura G, Higashimoto Y, Iwai J, Murayama K, Horie H, Suzuki T. A case of Alagille syndrome with hepatic-duct obliteration presenting intracranial hemorrhage. *Chiba Med J* 2013;**89**:181–185.

89. Molinero-Herguedas E, Labrador-Fuster T, Rios-Lazaro M, Carmaniu-Tobal J. Aortic aneurysm in Alagille syndrome. *Rev Esp Cardiol* 2008;**61**:658–659.

90. Moreau S, Bourdon N, Jokic M, Rugy MG de, Babin E, Valdazo A, Courtheoux P. Alagille syndrome with cavernous carotid artery aneurysm. *Int J Pediatr Otorhinolaryngol* 1999;**50**:139–143.

91. Nishikawa A, Mori H, Takahashi M, Ojima A, Shimokawa K, Fueuta T. ALAGILLE’S SYNDROME: A Case with a Hamartomatous Nodule of the Liver. *Pathol Int* 1987;**37**:1319–1326.

92. Tobin WO, Connor GO, Doherty CP, Guiney M, Redmond JMT, McCabe DJH. Low-flow retinopathy, anterograde amnesia and moya-moya phenomenon in alagille syndrome associated with a missence mutation in the Jagged 1 gene. *J Neurol Neurosurg Psychiatry* 2009;**80**.

93. Delaney S, O’Connor G, Reardon W, Murphy SJX, Tierney S, Ryan BM, Delaney H, Doherty CP, Guiney M, Brennan P, Tobin WO, McCabe DJH. Extracranial and Intracranial Vasculopathy With ‘Moyamoya Phenomenon’ in Association With Alagille Syndrome. *Front Neurol* **9**:1194.

94. O’Connor G, McCabe DJH, Guiney M, Redmond JMT, Doherty CP. Low flow retinopathy,anterograde amnesia and moya-moya phenomenon in Alagille syndrome. *Cerebrovasc Dis* 2009;**27**:124.

95. Özçay F, Varan B, Tokel K, Çetin I, Dalgiç A, Haberal M. Severe peripheral pulmonary artery stenosis is not a contraindication to liver transplantation in Alagille syndrome. *Pediatr Transplant* 2006;**10**:108–111.

96. Pati GK, Singh A, Nath P, Narayan J, Padhi PK, Parida PK, Pattnaik K, Panda C, Singh SP. A 10-year-old child presenting with syndromic paucity of bile ducts (Alagille syndrome): a case report. *J Med Case Rep* Journal of Medical Case Reports; 2016;**10**:1–5.

97. Quiros-Tejeira RE, Ament ME, Heyman MB, Martin MG, Rosenthal P, Hall TR, McDiarmid S V, Vargas JH. Variable morbidity in alagille syndrome: a review of 43 cases. *J Pediatr Gastroenterol Nutr* 1999;**29**:431–437.

98. Raas-Rothschild A, Shteyer E, Lerer I, Nir A, Granot E, Rein AJJT. Jagged1 gene mutation for abdominal coarctation of the aorta in Alagille syndrome. *Am J Med Genet* 2002;**112**:75–78.

99. Shefler AG, Chan MKH, Ostman-Smith I. Middle Aortic Syndrome in a Boy with Arteriohepatic Dysplasia (Alagille Syndrome). *Pediatr Cardiol* 1997;**18**:232–234.

100. Shimohata H, Imagawa K, Yamashita M, Ohgi K, Maruyama H, Takayasu M, Hirayama K, Kobayashi M. An Adult Patient with Alagille Syndrome Showing Mainly Renal Failure and Vascular Abnormality without Liver Manifestation. *Intern Med* 2020;**59**:2907–2910.

101. Silberbach M, Lashley D, Reller MD, Kinn WF, Terry A, Sunderland CO. Arteriohepatic dysplasia and cardiovascular malformations. *Am Heart J* 1994;**127**:695–699.

102. Takenouchi T, Shimozato S, Kosaki K, Momoshima S, Takahashi T. Reversible diffuse white matter lesion in alagille syndrome. *Pediatr Neurol* 2011;**45**:54–56.

103. Tschernigg M. Moyamoya in combination with Alagille-syndrome. *Am J Med Genet* 2002;**114**:823.

104. Wali AR, Kang KM, Rennert R, Santiago-Dieppa D, Khalessi AA, Levy M. First-in-Human Clinical Experience Using High-Definition Exoscope with Intraoperative Indocyanine Green for Clip Reconstruction of Unruptured Large Pediatric Aneurysm. *World Neurosurg* 2021;**151**:52.

105. Yokoyama K, Minami T, Seki M, Okada Y, Kumagai H, Yamagata T. A boy with Alagille syndrome coexisting with mid-aortic syndrome and renovascular hypertension. *J Cardiol cases* **21**:28–31.

106. Zhang W, Zhao XY, Huang J, Ou XJ, Jia JD. Alagille syndrome: an uncommon cause of intrahepatic cholestasis in adults. *Rev Esp Enfermedades Dig* 2019;**111**.

107. Zweier C, Hammersen G, Behrens R, Gläser C, Schmjdp C, Müller A, Ulmer HE, Raucji A. Severe course of alagille syndrome associated with a novel NOTCH2 mutation. *Genet Couns* 2010;**21**:132–133.

## Appendix Table S2

Appendix Table S2

| Measured parameter | P value | Significant difference for sex? | Additional Comments |
|---|---|---|---|
| 1B Spontaneous intracranial bleeds (PRISMA review) | 0.0117 | YES | 10 females with bleeds vs 1 male |
| 2A Survival of *Jag1*$^{Ndr/Ndr}$ mice vs WTs, by sex | >0.05 | NO | Female *Jag1*$^{Ndr/Ndr}$ mice die earlier than males and survival at P10 is lower, but the differences are not statistically significant between male and female *Jag1*$^{Ndr/Ndr}$ mice. |
| 2B Total Bilirubin at P10 | Not tested | Not tested | Differences between sexes not suggested by first 6 animals, additional biological replicates not justified. |
| 2C TAT at P10 | Not tested | Not tested | Differences between sexes, or between *Jag1*$^{Ndr/Ndr}$ mice vs WT mice, not supported by first 6 animals, additional biological replicates not justified. |
| 2D Fibrinogen at P10 | Not tested | Not tested | Differences between sexes, or between *Jag1*$^{Ndr/Ndr}$ mice vs WT mice, not supported by first 6 animals, additional biological replicates not justified. |
| 2E INR at P10 | Not tested | Not tested | Differences between sexes, or between *Jag1*$^{Ndr/Ndr}$ mice vs WT mice, not supported by first 6 animals, additional biological replicates not justified. |
| 2F Tail bleeding time at P10 | Not tested | Not tested | Differences between sexes, or between *Jag1*$^{Ndr/Ndr}$ mice vs WT mice, not supported by first 6 animals, additional biological replicates not justified. |
| 2H Skull Full thickness bone volume | >0.05 | No interaction of genotype with sex in two-way ANOVA | One outlier *Jag1*$^{Ndr/Ndr}$ male with very low skull volume: this male was inordinately small compared to other *Jag1*$^{Ndr/Ndr}$ mice. This is a sporadic phenomenon in the *Jag1*$^{Ndr/Ndr}$ colony. |

| | | | |
|---|---|---|---|
| 2I Skull compact bone volume | >0.05 | No interaction of genotype with sex in two-way ANOVA | One outlier *Jag1*$^{Ndr/Ndr}$ male with very low skull volume: this male was inordinately small compared to other *Jag1*$^{Ndr/Ndr}$ mice. This is a sporadic phenomenon in the *Jag1*$^{Ndr/Ndr}$ colony. |
| 2K Resin leakage outside blood vessels in liver casts | >0.05 | No interaction of genotype with sex in two-way ANOVA and no difference in t-test of male vs female *Jag1*$^{Ndr/Ndr}$ mice | All three *Jag1*$^{Ndr/Ndr}$ females exhibit some vascular weakness/resin leakage, and two of three had very large provoked leakage outside blood vessels. Two of three male *Jag1*$^{Ndr/Ndr}$ mice had small vascular leakages. |
| 2L Evans Blue in brain | >0.05 | No difference in t-test of male vs female *Jag1*$^{Ndr/Ndr}$ mice | Animal with largest amount of Evans blue in brain (outlier compared to controls) is a male *Jag1*$^{Ndr/Ndr}$ mouse. |
| 3B Arteriovenous crossings in adult retina | >0.05 | No interaction of genotype with sex in two-way ANOVA and no difference in Tukey's multiple comparisons test of male vs female Jag1Ndr/Ndr mice | |
| 3C Number of Major Arterioles in adult retina | >0.05 | No interaction of genotype with sex in two-way ANOVA and no difference in Tukey's multiple comparisons test of male vs female Jag1Ndr/Ndr mice | |
| 3D Number of Major venules in adult retina | 0.0302 | Interaction of genotype with sex in two-way ANOVA (p= 0,044) and difference in Tukey's multiple comparisons test of male vs female | Sex-specific venous defect: Adult *Jag1*$^{Ndr/Ndr}$ females have significantly fewer major venules in retina than males |

| | | | |
|---|---|---|---|
| | | Jag1Ndr/Ndr mice (p=0,0302) | |
| 3E Arterial Tortuosity by stage | Not tested | Not tested | |
| 3F Arterial Tortuosity by sex | >0.05 | No interaction of genotype with sex in two-way ANOVA and no difference in Tukey's multiple comparisons test of male vs female Jag1Ndr/Ndr mice | |
| 3G Venous Tortuosity by stage | Not tested | Not tested | |
| 3H Venous Tortuosity by sex | 0.0031 | Interaction of genotype with sex in two-way ANOVA (p= 0,0223) and difference in Tukey's multiple comparisons test of male vs female Jag1Ndr/Ndr mice (p=0,0031) | Sex-specific venous defect: Adult *Jag1*$^{Ndr/Ndr}$ females have significantly higher venous tortuosity in retina than males |
| 3J Middle Cerebral Artery Tortuosity at P10 | >0.05 | No interaction of genotype with sex in two-way ANOVA | |
| 3L Middle Cerebral Artery Branching at P10 | >0.05 | No interaction of genotype with sex in two-way ANOVA | |
| 4F aSMA gaps per age group | Not tested | Not tested | 1 out of 3 of adult *Jag1*$^{Ndr/Ndr}$ show aSMA gaps in retina, increased to 3 out of 6 mice in 1-year-old *Jag1*$^{Ndr/Ndr}$ age group (all males) |
| 4G aSMA negative gaps per retina | Not tested | Not tested | 1-year-old *Jag1*$^{Ndr/Ndr}$ mice (N=6) had significantly more aSMA gaps in retina compared to 1-year-old WT (of which the three males had the highest increase) |

| | | | |
|---|---|---|---|
| 4J Cleaved Caspase 3+ cells | Not tested | Not tested | Significantly more apoptotic cells in 1-year-old *Jag1*$^{Ndr/Ndr}$ mice compared to 1-year-old WT (the three males had the highest increase) |
| 4L Middle Cerebral Artery aSMA nuclei | Not tested | Not tested | |
| 4N Mean Blood pressure | >0.05 | No interaction of genotype with sex in two-way ANOVA | |
| 4O Systolic Blood pressure | Not tested | Not tested | |
| 4P Evans Blue-Angiotensin II in adult brain | Not tested | Not tested | |
| 4S aSMA negative gaps Angiotensin II | Not tested | Not tested | |
| 5B SCP Branching Points P30 | Not tested | Not tested | |
| 5C ICP Vascular Length P30 | Not tested | Not tested | |
| 5D ICP Branching Points P30 | Not tested | Not tested | |
| 5F SCP Branching Points 3-6 months | Not tested | Not tested | |
| 5G ICP Vascular Length 3-6 months | Not tested | Not tested | |
| 5H ICP Branching Points 3-6 months | Not tested | Not tested | |
| 5J Collagen IV sleeve 3-6 months | Not tested | Not tested | Empty COLIV sleeves per are increased in ICP of two *Jag1*$^{Ndr/Ndr}$ females |
| 5L Vertical sprouts 3-6 months | Not tested | Not tested | |
| 5M Vertical Sprouts 1 year | Not tested | Not tested | |
| 5P Neurofilament coverage | Not tested | Not tested | |
| 6B CADASIL AV Crossings | Not tested | Not tested | |
| 6C CADASIL Number of Major Arterioles in retina | Not tested | Not tested | |
| 6D CADASIL Number of Major Venules in retina | Not tested | Not tested | |
| 6E CADASIL Arterial Tortuosity | Not tested | Not tested | |
| 6F CADASIL Venous Tortuosity | Not tested | Not tested | |

| | | | |
|---|---|---|---|
| 6G ALGS AV Crossings | Not tested | Not tested | |
| 6H ALGS Number of Major Arterioles in retina | Not tested | Not tested | |
| 6I ALGS Number of Major Venules in retina | Not tested | Not tested | |
| 6J ALGS Arterial Tortuosity | Not tested | Not tested | |
| 6K ALGS Venous Tortuosity | Not tested | Not tested | |

# Appendix Supplementary Materials and Methods

## Systematic review search strategy

The Medline (Ovid), Embase and Web of Science Core Collection database were used for publication search. After the initial search, automated deduplication was performed by the Karolinska Institutet library. The databases were searched up to June 14$^{th}$, 2021. Two reviewers manually screened, reviewed and collected each record independently. The systematic review followed the Prisma 2000 guidelines (*25*). The systematic review was not registered, and a review protocol was not prepared. Records that had no abstract available in English and publications that were not accessible were excluded. Publications that were included in the systematic review contained information about the ALGS diagnosis, patient sex and vascular events except for pulmonary artery stenosis, which is a hallmark of ALGS. Patients that explicitly did not meet the criteria for diagnosis (genetically confirmed, or presenting three of five diagnostic features, or relatives of confirmed cases with at least two hallmarks) were excluded. Additional reasons for exclusion were bleeding as a consequence of hepatocellular carcinoma, displaced blood vessels secondary to other primary defects, splenic hamartoma (benign splenic mass, not thought of as a vascular lesion), or aortic stenosis stated to be a consequence of a bicuspid valve. Assumption was made about the patient's sex if the patient was referred to as "he" (male) and "she" (female). Non-peer reviewed abstracts from conferences were included. Structural abnormalities and vascular events were tabulated for all reports. Structural abnormalities quantified included stenosis, collateral vessel or occlusion, aneurysm, Moyamoya, vessel agenesis or hypoplasia, vessel wall abnormality, skin vessel abnormality, vascular bruits and persistent falcine sinus. Vascular events included ischemic or hemorrhagic events or post-surgical vascular complications. The findings of the studies were tabulated and compared for males and females. If a study contained a vascular abnormality that was not part of any group of the synthesis, the patient's information was included only in the patient overview table (Table S1). After the full screen by two reviewers, the two independent

lists were compared and discussed to identify any mismatches and determine final inclusion/exclusion. Articles with relevant vascular information but lacking sex data were pursued by contacting study authors for more information. None of the study authors were able to provide sex data where this was missing from the original publication. One potential source of bias in the reports included is presented by the fact that the majority come from hepatology centers, which follow the patients for liver involvement and may therefore exhibit publication bias towards more overt vascular events such as intracranial hemorrhage. Therefore, different types of bleeding events (eg nose bleeds versus intracranial bleeds) should not be compared with one another. There is no obvious risk of bias comparing impact of sex within each vascular event. To help reduce publication bias from double reporting we describe the total number of assessed publications as "Reports included in studies" and the total number of assessed publication per patient as "Studies included in review". Data are presented as summary of reported events in males and females and the difference between males and females was tested using two-tailed binominal exact test. For observed vs expected outcomes, a 1:1 distribution of cases between males and females was assumed to be expected.

Search strategy for Medline:

Field labels: exp/ = exploded MeSH term; / = non exploded MeSH term; .ti,ab,kf. = title, abstract and author keywords; adjx = within x words, regardless of order; * = truncation of word for alternate endings
1. Alagille Syndrome/
2. (alagill* adj3 (syndrome* or watson or disease)).ti,ab,kf.
3. (watson miller or arteriohepatic dysplasia or cardiovertebral syndrome or hepatic ductular hypoplasia or hepatofacioneurocardiovertebral or cholestasis with peripheral pulmonary stenosis or paucity of interlobular bile ducts or hepatic ductular hypoplasia).ti,ab,kf. or/1-3
5. exp Cardiovascular System/ab, pa [Abnormalities, Pathology]
6. exp Vascular Diseases/
7. exp Hemorrhage/
8. exp Vascular Malformations/
9. (vascula* or cerebrovasc* or stroke* or aneurysm* or blood vessel* or artery or arteries or vein* or venous or moyamoya or moya moya or bleed* or hemorrhag*).ti,ab,kf.
10. or/5-9
11. 4 and 10
12. remove duplicates from 11

Search strategy for Embase:

Field labels: /exp = exploded Emtree term; /de = non exploded Emtree term; ti,ab = title and abstract; NEAR/x = within x words, regardless of order; * = truncation of word for alternate endings (('alagille syndrome'/de) OR ((alagill* NEAR/3 (syndrome* OR watson OR disease)):ti,ab,kw) OR ('watson miller':ti,ab,kw OR 'arteriohepatic dysplasia':ti,ab,kw OR 'cardiovertebral syndrome':ti,ab,kw OR hepatofacioneurocardiovertebral:ti,ab,kw OR 'cholestasis with peripheral pulmonary stenosis':ti,ab,kw OR 'paucity of interlobular bile ducts':ti,ab,kw OR 'hepatic ductular hypoplasia':ti,ab,kw)) AND (('cardiovascular system'/exp AND (abnormal* OR patholog*)) OR ('vascular disease'/exp) OR ('bleeding'/exp) OR (vascula*:ti,ab,kw OR cerebrovasc*:ti,ab,kw OR stroke*:ti,ab,kw OR aneurysm*:ti,ab,kw OR 'blood vessel*':ti,ab,kw OR artery:ti,ab,kw OR arteries:ti,ab,kw OR vein*:ti,ab,kw OR venous:ti,ab,kw OR moyamoya:ti,ab,kw OR 'moya moya':ti,ab,kw OR bleed*:ti,ab,kw OR hemorrhag*:ti,ab,kw))

Search strategy for Web of Science Core Collection:

Field labels: TS/Topic = title, abstract, author keywords and Keywords Plus; NEAR/x = within x words, regardless of order; * = truncation of word for alternate endings TS=(alagill* NEAR/3 (syndrome* OR watson OR disease)) OR TS=("watson miller" OR "arteriohepatic dysplasia" OR "cardiovertebral syndrome" OR hepatofacioneurocardiovertebral OR "cholestasis with peripheral pulmonary stenosis" OR "paucity of interlobular bile ducts" OR "hepatic ductular hypoplasia") AND TS=(vascula* OR cerebrovasc* OR stroke* OR aneurysm* OR "blood vessel*" OR artery OR arteries OR vein* OR venous OR moyamoya OR"moya moya" OR bleed* OR hemorrhag*)

## Prisma 2020 Checklist

| Section and Topic | Item # | Checklist item | Location where item is reported |
|---|---|---|---|
| **TITLE** | | | |
| Title | 1 | Identify the report as a systematic review. | Materials and Methods, Introduction, Results |
| **ABSTRACT** | | | |
| Abstract | 2 | See the PRISMA 2020 for Abstracts checklist. | Abstract |
| **INTRODUCTION** | | | |
| Rationale | 3 | Describe the rationale for the review in the context of existing knowledge. | Introduction |
| Objectives | 4 | Provide an explicit statement of the objective(s) or question(s) the review addresses. | Introduction & Results |
| **METHODS** | | | |
| Eligibility criteria | 5 | Specify the inclusion and exclusion criteria for the review and how studies were grouped for the syntheses. | Appendix Materials and Methods |
| Information sources | 6 | Specify all databases, registers, websites, organisations, reference lists and other sources searched or consulted to identify studies. Specify the date when each source was last searched or consulted. | Appendix Materials and Methods |
| Search strategy | 7 | Present the full search strategies for all databases, registers and websites, including any filters and limits used. | Appendix Materials and Methods |
| Selection process | 8 | Specify the methods used to decide whether a study met the inclusion criteria of the review, including how many reviewers screened each record and each report retrieved, whether they worked independently, and if applicable, details of automation tools used in the process. | Appendix Materials and Methods |
| Data collection process | 9 | Specify the methods used to collect data from reports, including how many reviewers collected data from each report, whether they worked independently, any processes for obtaining or confirming data from study investigators, and if applicable, details of automation tools used in the process. | Appendix Materials and Methods |
| Data items | 10a | List and define all outcomes for which data were sought. Specify whether all results that were compatible with each outcome domain in each study were sought (e.g. for all measures, time points, analyses), and if not, the methods used to decide which results to collect. | Appendix Materials and Methods Figure 1 Source Data, Hankeova et al Systematic Review Table Included Reports |
| | 10b | List and define all other variables for which data were sought (e.g. participant and intervention characteristics, funding sources). Describe any assumptions made about any missing or unclear information. | Appendix Materials and Methods |

| Section and Topic | Item # | Checklist item | Location where item is reported |
|---|---|---|---|
| Study risk of bias assessment | 11 | Specify the methods used to assess risk of bias in the included studies, including details of the tool(s) used, how many reviewers assessed each study and whether they worked independently, and if applicable, details of automation tools used in the process. | Appendix Materials and Methods |
| Effect measures | 12 | Specify for each outcome the effect measure(s) (e.g. risk ratio, mean difference) used in the synthesis or presentation of results. | Appendix Materials and Methods, Figure 1B, Appendix Figure S1 |
| Synthesis methods | 13a | Describe the processes used to decide which studies were eligible for each synthesis (e.g. tabulating the study intervention characteristics and comparing against the planned groups for each synthesis (item #5)). | Appendix Materials and Methods |
| | 13b | Describe any methods required to prepare the data for presentation or synthesis, such as handling of missing summary statistics, or data conversions. | Appendix Materials and Methods |
| | 13c | Describe any methods used to tabulate or visually display results of individual studies and syntheses. | Appendix Materials and Methods, Figure 1 Source Data |
| | 13d | Describe any methods used to synthesize results and provide a rationale for the choice(s). If meta-analysis was performed, describe the model(s), method(s) to identify the presence and extent of statistical heterogeneity, and software package(s) used. | Appendix Materials and Methods, Figure 1 Source Data |
| | 13e | Describe any methods used to explore possible causes of heterogeneity among study results (e.g. subgroup analysis, meta-regression). | NA |
| | 13f | Describe any sensitivity analyses conducted to assess robustness of the synthesized results. | NA |
| Reporting bias assessment | 14 | Describe any methods used to assess risk of bias due to missing results in a synthesis (arising from reporting biases). | NA |
| Certainty assessment | 15 | Describe any methods used to assess certainty (or confidence) in the body of evidence for an outcome. | NA |
| **RESULTS** | | | |
| Study selection | 16a | Describe the results of the search and selection process, from the number of records identified in the search to the number of studies included in the review, ideally using a flow diagram. | Appendix Materials and Methods, Flow Diagram at the end of this Appendix |
| | 16b | Cite studies that might appear to meet the inclusion criteria, but which were excluded, and explain why they were excluded. | NA |
| Study characteristics | 17 | Cite each included study and present its characteristics. | Appendix Table S1 |
| Risk of bias in studies | 18 | Present assessments of risk of bias for each included study. | Appendix Materials and Methods |

| Section and Topic | Item # | Checklist item | Location where item is reported |
|---|---|---|---|
| Results of individual studies | 19 | For all outcomes, present, for each study: (a) summary statistics for each group (where appropriate) and (b) an effect estimate and its precision (e.g. confidence/credible interval), ideally using structured tables or plots. | Figure 1, Figure S1 |
| Results of syntheses | 20a | For each synthesis, briefly summarise the characteristics and risk of bias among contributing studies. | NA |
| | 20b | Present results of all statistical syntheses conducted. If meta-analysis was done, present for each the summary estimate and its precision (e.g. confidence/credible interval) and measures of statistical heterogeneity. If comparing groups, describe the direction of the effect. | Figure 1, Source data for Figure 1 |
| | 20c | Present results of all investigations of possible causes of heterogeneity among study results. | NA |
| | 20d | Present results of all sensitivity analyses conducted to assess the robustness of the synthesized results. | NA |
| Reporting biases | 21 | Present assessments of risk of bias due to missing results (arising from reporting biases) for each synthesis assessed. | NA |
| Certainty of evidence | 22 | Present assessments of certainty (or confidence) in the body of evidence for each outcome assessed. | NA |
| **DISCUSSION** | | | |
| Discussion | 23a | Provide a general interpretation of the results in the context of other evidence. | Results section |
| | 23b | Discuss any limitations of the evidence included in the review. | Results section |
| | 23c | Discuss any limitations of the review processes used. | Results section |
| | 23d | Discuss implications of the results for practice, policy, and future research. | Results section |
| **OTHER INFORMATION** | | | |
| Registration and protocol | 24a | Provide registration information for the review, including register name and registration number, or state that the review was not registered. | Appendix Materials and Methods |
| | 24b | Indicate where the review protocol can be accessed, or state that a protocol was not prepared. | Appendix Materials and Methods |
| | 24c | Describe and explain any amendments to information provided at registration or in the protocol. | NA |
| Support | 25 | Describe sources of financial or non-financial support for the review, and the role of the funders or sponsors in the review. | Acknowledgements and Funding section |
| Competing interests | 26 | Declare any competing interests of review authors. | Conflict of interest statement |
| Availability of data, code and other materials | 27 | Report which of the following are publicly available and where they can be found: template data collection forms; data extracted from included studies; data used for all analyses; analytic code; any other materials used in the review. | Table 1, Table S1 |

*From:* Page MJ, McKenzie JE, Bossuyt PM, Boutron I, Hoffmann TC, Mulrow CD, et al. The PRISMA 2020 statement: an updated guideline for reporting systematic reviews. BMJ 2021;372:n71. doi: 10.1136/bmj.n71

For more information, visit: http://www.prisma-statement.org/

Prisma 2020 Flow Diagram

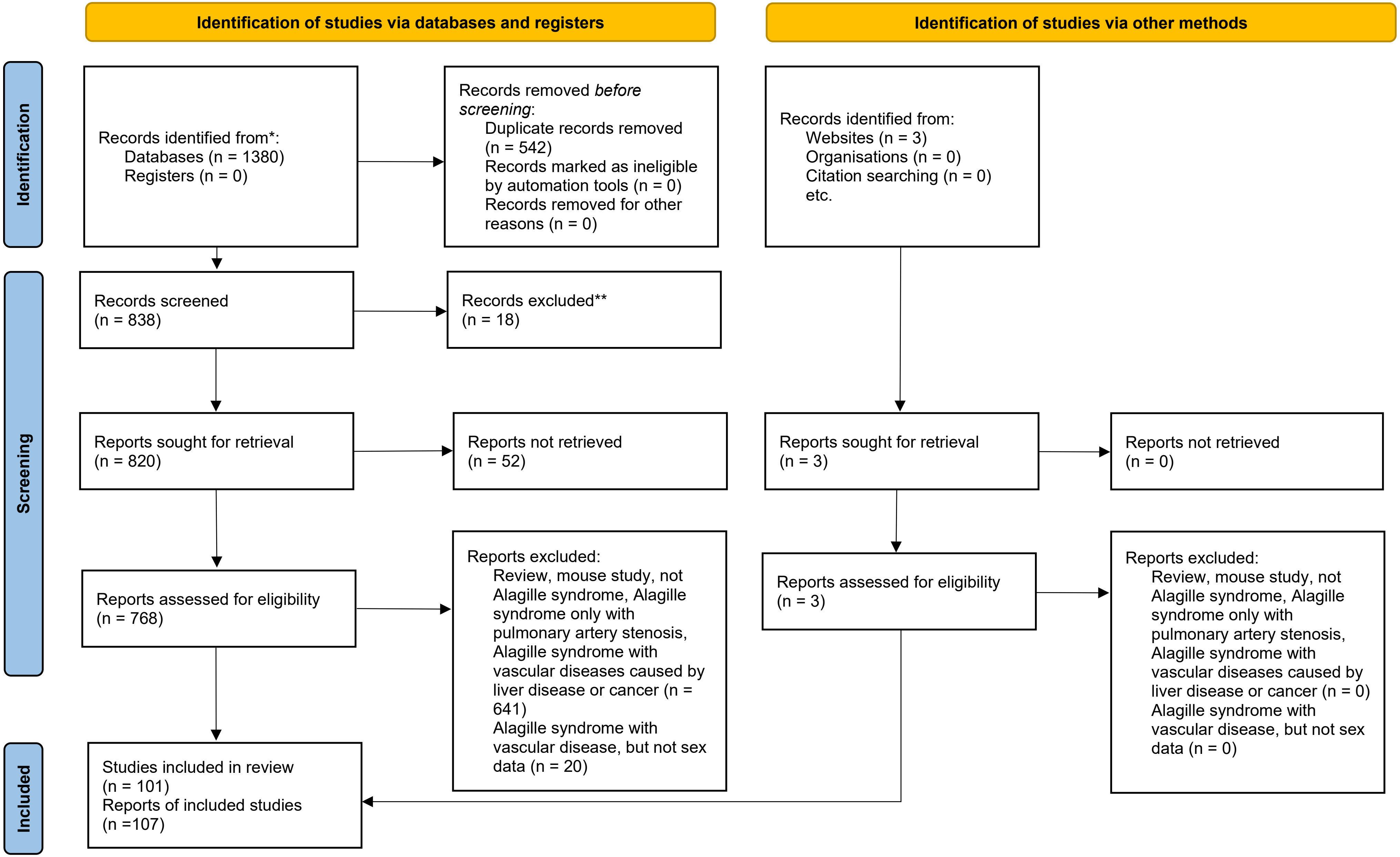

Supplement: Supplementary file 1 — Appendix [file EMMM-14-e15809-s007.pdf]
