# Supplementary material for: Sex differences and risk factors for bleeding in Alagille syndrome
Source: EMBO Mol Med. 2022 Nov 8;14(12):e15809. doi: 10.15252/emmm.202215809 (PMC9728057; doi:10.15252/emmm.202215809)
Supplement: Supplementary file 2 — Expanded View Figures PDF [file EMMM-14-e15809-s013.pdf]

Expanded View Figures

Figure EV1. *Jag1*$^{Ndr/Ndr}$ mice display thinner skulls.

A Micro computed tomography (μCT) of P30 skulls. Blue arrow marks side skull protrusions in *Jag1*$^{Ndr/Ndr}$ mice.
B, C (B) Color map displaying skull full thickness. (C) Cross-section of dorsal cranium in fully segmented skull.
D, E (D) Color map displaying cranial compact bone thickness. (E) Cross-section of dorsal cranium of segmented compact bones.
F Skull length from occipital bone to nasal bone (measured in mid line), (n = 5-6 per group, Two-way ANOVA followed by Šídák's multiple comparison test, Interaction P = 0.9309, Sex P = 0.0069, Genotype **P = 0.0071).
G, H (G) Segmented temporal bone. (H) Temporal bone volume (n = 5-6 per group, Two-way ANOVA followed by Šídák's multiple comparison test, Interaction P = 0.9960, Sex P = 0.3691, Genotype *P = 0.0249).

Data information: Bar graphs depict mean values ± standard deviation, each dot represents one biological replicate. Circles represent females, squares represent males. For details/results of statistical analyses, please see source data.
Source data are available online for this figure.

© 2022 The Authors

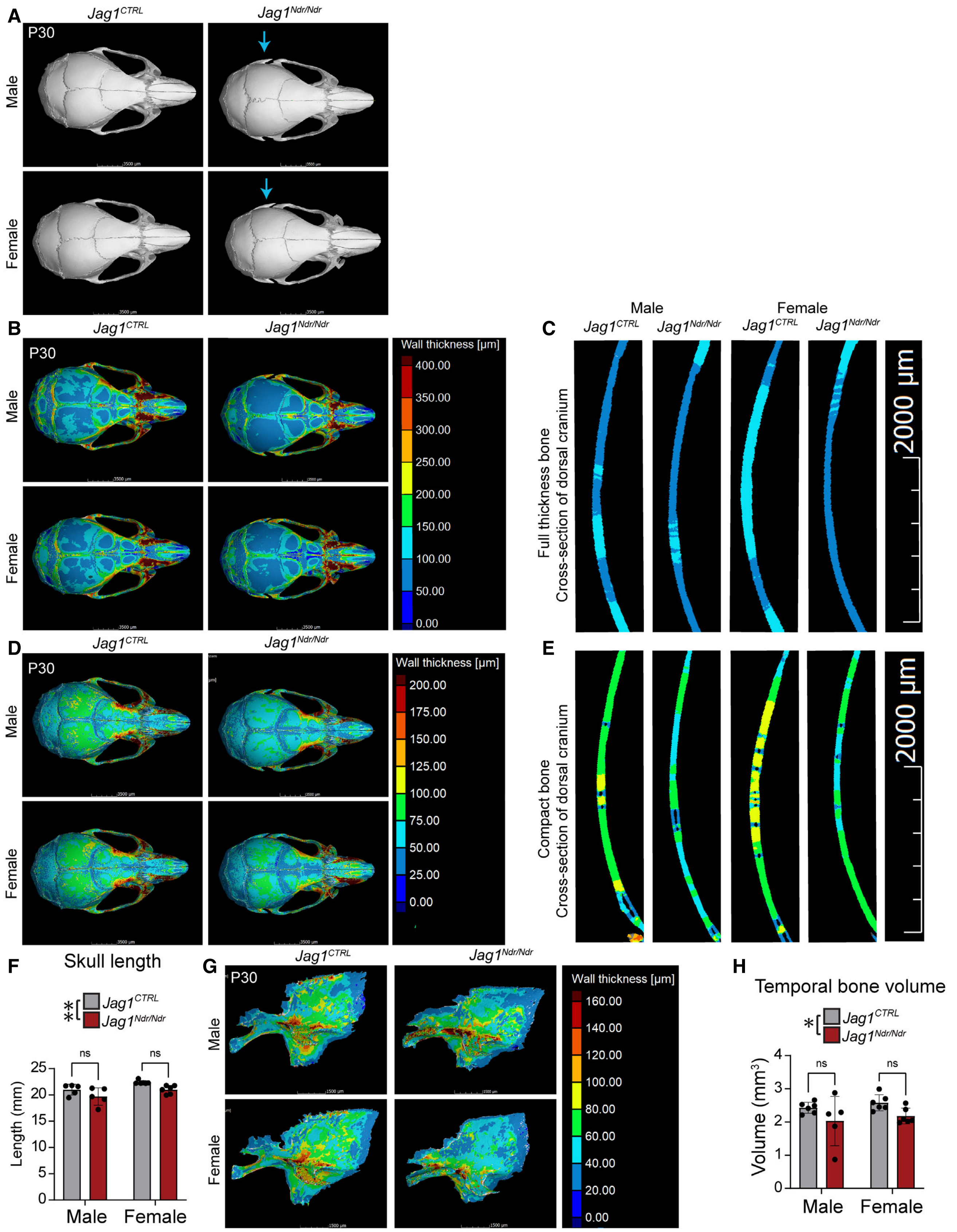
A
Jag1CTRL
Jag1Ndr/Ndr
P30
Male
Female
B
Jag1CTRL
Jag1Ndr/Ndr
P30
Male
Female
Wall thickness [µm]
400.00
350.00
300.00
250.00
200.00
150.00
100.00
50.00
0.00
C
Male
Female
Jag1CTRL
Jag1Ndr/Ndr
Jag1CTRL
Jag1Ndr/Ndr
Full thickness bone
Cross-section of dorsal cranium
2000 µm
D
Jag1CTRL
Jag1Ndr/Ndr
P30
Male
Female
Wall thickness [µm]
200.00
175.00
150.00
125.00
100.00
75.00
50.00
25.00
0.00
E
Compact bone
Cross-section of dorsal cranium
2000 µm
F
Skull length
Jag1CTRL
Jag1Ndr/Ndr
**
ns
ns
Length (mm)
0
5
10
15
20
25
Male
Female
G
Jag1CTRL
Jag1Ndr/Ndr
P30
Male
Female
Wall thickness [µm]
160.00
140.00
120.00
100.00
80.00
60.00
40.00
20.00
0.00
H
Temporal bone volume
Jag1CTRL
Jag1Ndr/Ndr
*
ns
ns
Volume (mm3)
0
1
2
3
4
Male
Female

Figure EV1.

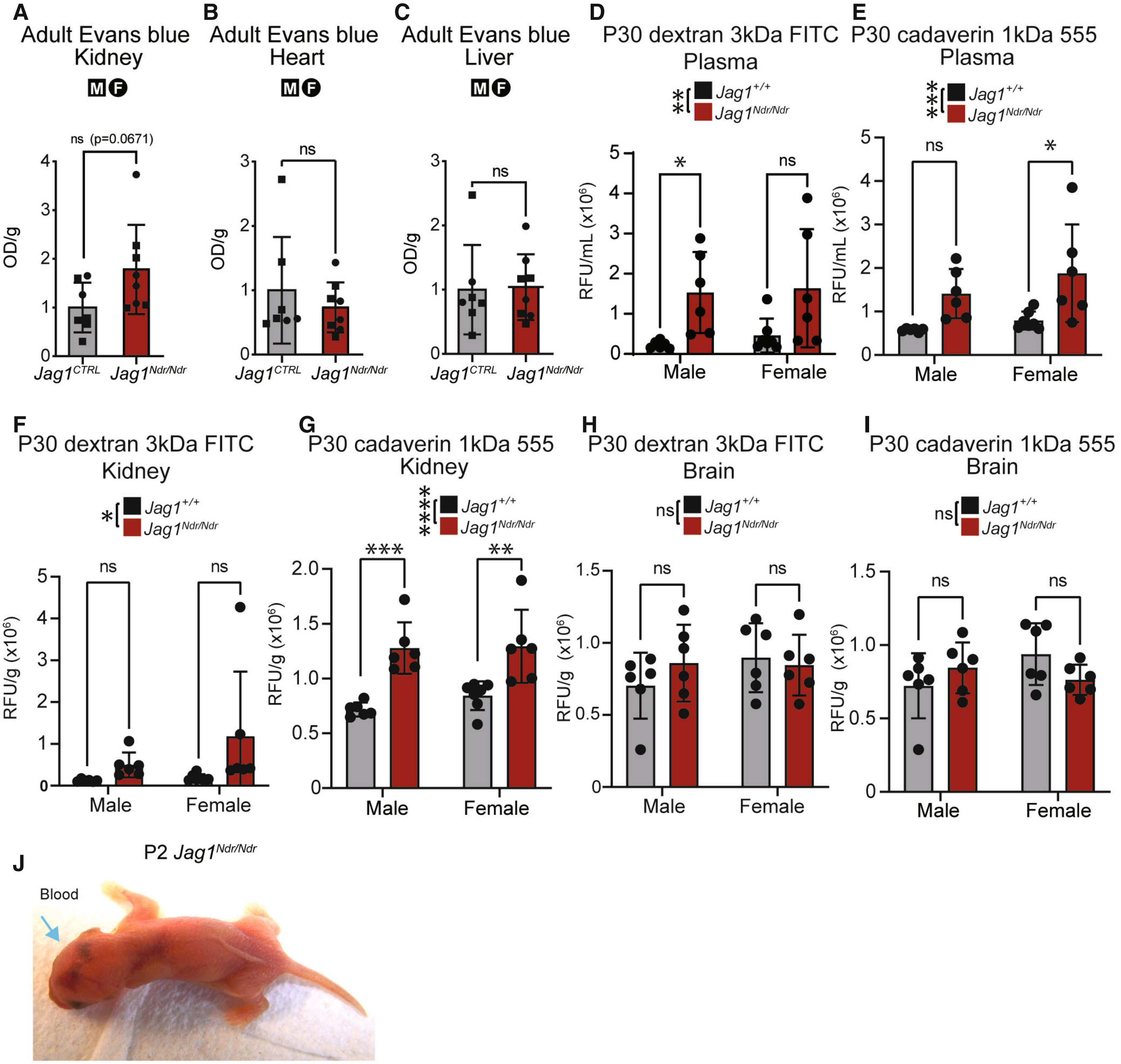

Figure EV2. *Jag1*$^{Ndr/Ndr}$ mice exhibit selective renal vascular permeability.

A–C Relative vascular leakage as assessed by Evans blue extracted from adult (A) kidney ($P = 0.0671$, ns), (B) heart ($P = 0.4316$, ns), and (C) liver ($P = 0.8988$, ns) of *Jag1*^{CTRL} and *Jag1*$^{Ndr/Ndr}$ adult mice (n = 7-8 per group, Unpaired t-test).

D, E Relative fluorescence in P30 plasma (D) of 3 kDa Dextran FITC (Two-way ANOVA on biological replicates/individual mice, followed by Šídák's multiple comparison test; Interaction $P = 0.08663$, Sex $P = 0.6541$, Genotype $^{**}P = 0.0025$) or (E) 1 kDa Cadaverin 555 (Two-way ANOVA on biological replicates/individual mice followed by Šídák's multiple comparison test; Interaction $P = 0.625$, Sex $P = 0.186$, Genotype $^{***}P = 0.0009$).

F, G Relative vascular permeability in P30 kidney assessed by relative fluorescence of (F) 3 kDa Dextran FITC (Two-way ANOVA on biological replicates/individual mice, followed by Šídák's multiple comparison test; Interaction $P = 0.3296$, Sex $P = 0.2374$, Genotype $^{*}P = 0.0384$) (G) or 1 kDa Cadaverin 555 (Two-way ANOVA on biological replicates/individual mice, followed by Šídák's multiple comparison test; Interaction $P = 0.5308$, Sex $P = 0.4112$, Genotype $^{****}P < 0.0001$).

H, I Relative vascular permeability in P30 brain assessed by relative fluorescence of (H) 3 kDa Dextran FITC (Two-way ANOVA on biological replicates/individual mice, followed by Šídák's multiple comparison test; Interaction $P = 0.2942$, Sex $P = 0.3624$, Genotype $P = 0.591$) or (I) 1 kDa Cadaverin 555 (Two-way ANOVA on biological replicates/individual mice, followed by Šídák's multiple comparison test; Interaction $P = 0.0596$, Sex $P = 0.3806$, Genotype $P = 0.7301$).

J Hemorrhages in brain and body of one *Jag1*$^{Ndr/Ndr}$ pup at P2, corresponding to brain data in Fig 2M, brain dissected out the next day at P3

Data information: Bar graphs depict mean values ± standard deviation, each dot represents one biological replicate. Circles represent females, squares represent males (A-C). For details/results of statistical analyses, please see source data.
Source data are available online for this figure.

 © 2022 The Authors

Figure EV3. Delayed retinal vascular outgrowth and remodeling in *Jag1*$^{Ndr/Ndr}$ mice.

A Schematic depicting retinal angiogenesis between P0 and P15. S, superficial; I, intermediate; D, deep capillary plexus.

B–F (B) P5 retinal vascular outgrowth, (C) quantified (n = 6 per group, unpaired t-test, *P = 0.0388). Scale bar 100 μm. (D) P5 vascular front with tip cells (boxed region). White arrowhead points to ERG+ tip cell nucleus, (E) quantified (n = 6, unpaired t-test, P = 0.1281, ns), blue arrowheads point to tips (bundles of filopodia) of tip cell (F) quantified (n = 6, unpaired t-test, **P = 0.0059). Scale bar 20 μm.

G–M Retinal vasculature at (G) P5, (H) P10, (I) P15. Scale bar (G) 50 μm, (H, I) 20 μm. Retinal blood vessel remodeling quantification at P5, P10, and P15 (J) vascular length per field (Two-way ANOVA on biological replicates/individual mice. Interaction ****P < 0.0001, Age ***P = 0.0006, Genotype P = 0.2023. Šídák's multiple comparison test: P5 *Jag1*^{CTRL} vs. *Jag1*$^{Ndr/Ndr}$ ****P < 0.0001; P15 *Jag1*^{CTRL} vs. *Jag1*$^{Ndr/Ndr}$ *P = 0.0233), (K) number of ERG+ cells per field (Two-way ANOVA on biological replicates/individual mice. Interaction ****P < 0.0001, Age ****P < 0.0001, Genotype P = 0.7656. Šídák's multiple comparison test: P5 *Jag1*^{CTRL} vs *Jag1*$^{Ndr/Ndr}$ **P = 0.0013; P15 *Jag1*^{CTRL} vs *Jag1*$^{Ndr/Ndr}$ *P = 0.0217), (L) number of ERG+ cells per vascular length (Two-way ANOVA on biological replicates/individual mice. Interaction P = 0.4617, Age **P = 0.0018, Genotype P = 0.2235), (M) number of branching points per field (n = 6 per group, Two-way ANOVA on biological replicates/individual mice. Interaction ****P < 0.0001, Age ****P < 0.0001, Genotype P = 0.2719. Šídák's multiple comparison test: P5 *Jag1*^{CTRL} vs. *Jag1*$^{Ndr/Ndr}$ ****P < 0.0001).

N Immunofluorescence of PH3+ proliferating CD31+ endothelial cells at P5. The dotted line labels the edge of the vascular front. Quantification of the number of proliferating cells per radial zone, normalized to area size at P5 (n = 4, Two-way ANOVA on biological replicates/individual mice. Interaction P = 0.3044, Zone ****P < 0.0001, Genotype *P = 0.0165). Scale bar 50 μm.

O, P (O) Delta like 4 in P5 vasculature. Scale bar 20 μm. White brackets denote high DLL4 activity. (P) *Dll4* relative mRNA levels in whole retina lysates (n = 6, unpaired t-test, *P = 0.0461).

Data information: Bar graphs depict mean values ± standard deviation, each dot represents one biological replicate. Circles represent females, squares represent males. For details/results of statistical analyses, please see source data.
Source data are available online for this figure.

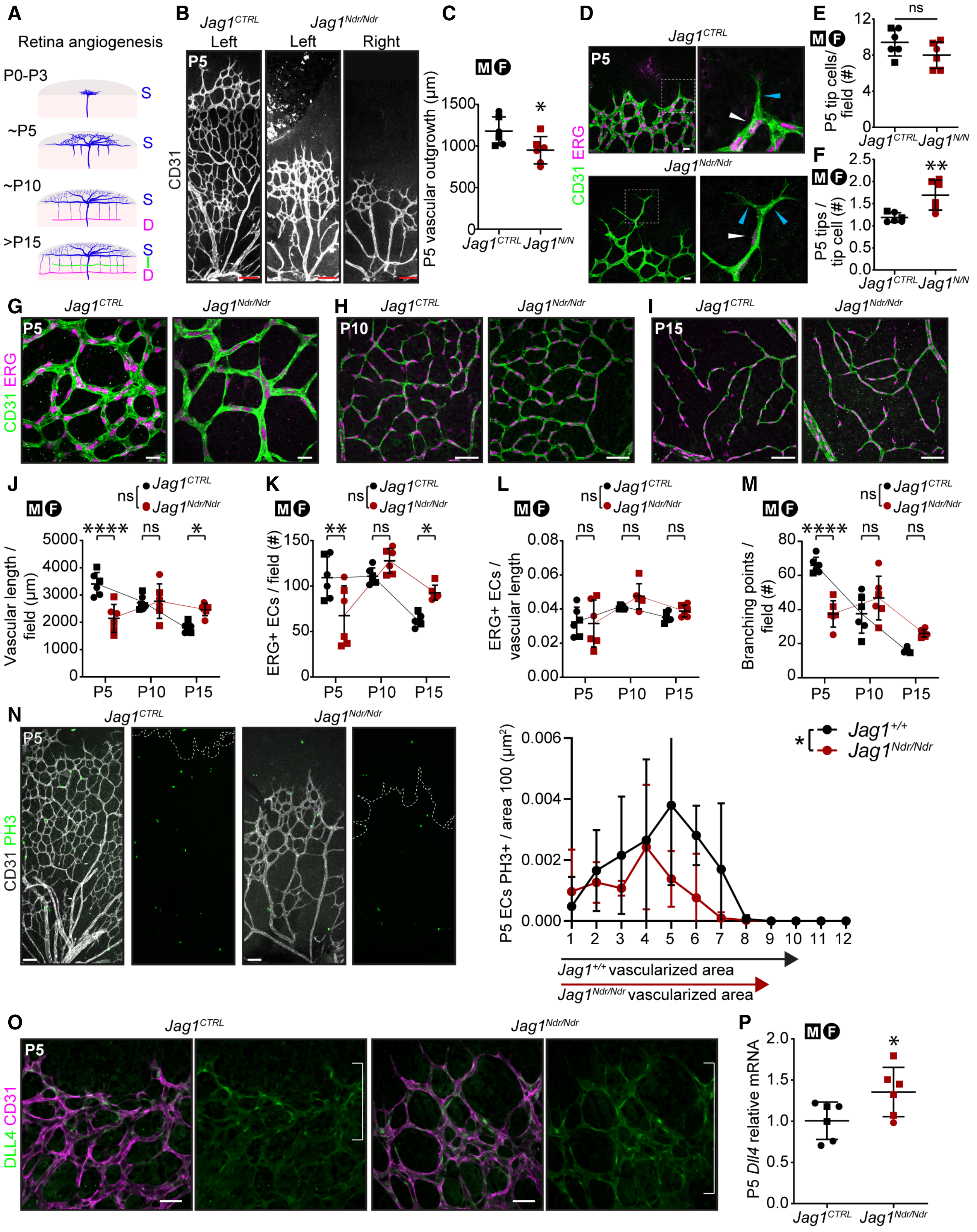

Figure EV3.

© 2022 The Authors

Figure EV4. *Jag1$^{Ndr/Ndr}$* mice display CADASIL-like sparse vascular smooth muscle cell coverage of arteries with an increase in artery-associated reactive astrocytes.

A, B (A) CD13 pericyte coverage of blood vessels was not reduced in adult *Jag1$^{Ndr/Ndr}$* mice. Scale bars 20 μm. (B) Quantification of CD13 intensity per field (n = 5-6 per group, Two-way ANOVA followed by Šídák's multiple comparison test, Interaction P = 0.0737, Sex P = 0.1642, Genotype P = 0.3706).

C Transmission electron microscopy of coronary arteries of adult mice. Vascular smooth muscle cells (SMC) are pseudo-colored in magenta and endothelial cells (ECs) in green. White arrowheads label SMC edges and the distances between SMCs. Green arrowhead marks the tight junctions. Scale bars 2 μm.

D, E (D) Staining for contractile (αSMA, SM22) and synthetic (VIM) vascular SMC (E) with quantification (n = 6–9 per group, Multiple unpaired t-tests, αSMA *P = 0.0214, SM22 *P = 0.0302, VIM P = 0.6801). Boxed region indicates region with αSMA-negative gap (labeled by white arrowhead). Scale bar 20 μm.

F, G (F) Staining for contractile (αSMA, MYH11) and synthetic (COLI) vascular SMC. Boxed region indicates region with αSMA-negative gap (labeled by white arrowhead). Scale bar 20 μm. (G) Relative decrease in fluorescence intensity of different vascular SMC markers within gap compared to within vascular SMC (set to 100%), (n = 5-11 per group, one-way ANOVA $P < 0.0001$, followed by Dunnett's multiple comparisons test: ASMA vs. COLI Adjusted **P = 0.0066, ASMA vs. VIM adjusted ****$P < 0.0001$).

H–K (H) GFAP+ astrocytes are more prevalent around adult *Jag1$^{Ndr/Ndr}$* arterioles (I) but not veins. White arrowheads label reactive astrocytes. Scale bars 20 μm. (J, K) Quantification of mean GFAP intensity on retinal (J) arterioles (n = 4, unpaired t-test, **P = 0.0052), (K) venules (n = 4, unpaired t-test, P = 0.6547).

L, M (L) Number of arteriolar first-generation branch points in 1-year-old mice (n = 6, unpaired t-test, P = 0.6823). (M) Number of cCasp3+ cells associated with first-generation arteriolar branching point (n = 6, unpaired t-test, *P = 0.0153).

N Evans blue extracted from liver, kidney, and heart of mice treated with PBS or Angiotensin II (n = 4-5 per group, two-way ANOVA with Subject matching. Interaction P = 0.2576, Organ P = 0.0011, Genotype & Treatment P = 0.2086 Subject ***P = 0.0004, followed by Tukey's multiple comparison test, *Jag1$^{+/+}$* vs. *Jag1$^{+/+}$* AngII *$P < 0.05$).

Data information: Bar graphs depict mean values ± standard deviation, each dot represents one biological replicate. Circles represent females, squares represent males. For details/results of statistical analyses, please see source data. A, arteriole; AngII, Angiotensin II; EC, endothelial cell; SMC, smooth muscle cell; V, venule.
Source data are available online for this figure.

© 2022 The Authors

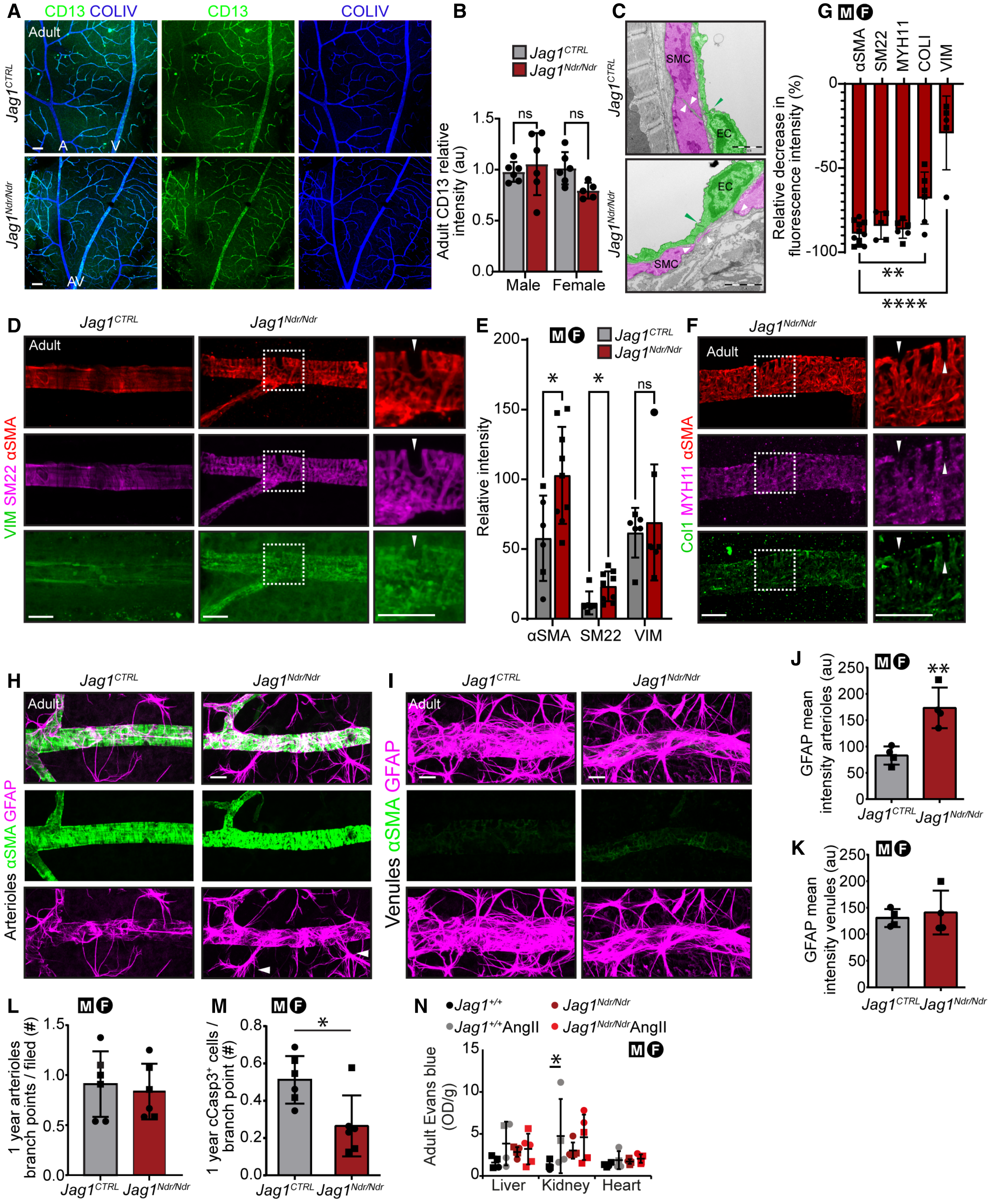

Figure EV4.

 © 2022 The Authors

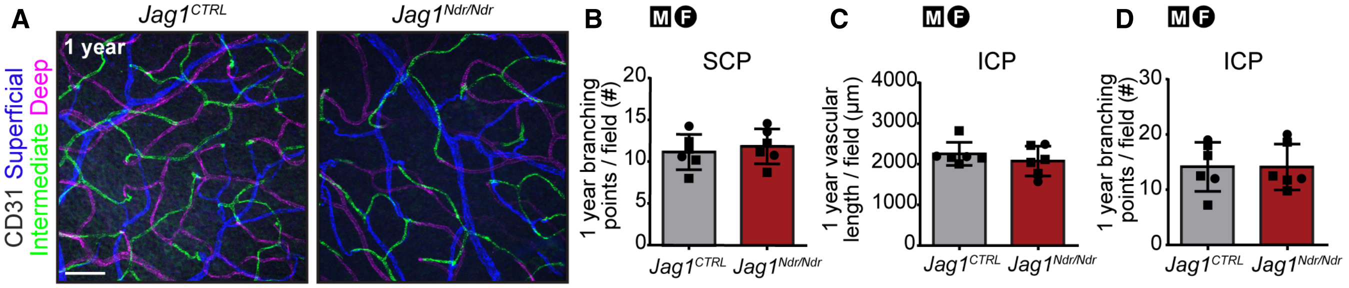

Figure EV5. Aging retina capillary network in 1-year-old mice.

A One-year-old retina three CD31+ capillary layers. Scale bar 50 µm.
B One-year-old SCP branching point number ($n = 6$ per group, unpaired t-test, $P = 0.5730$).
C One-year-old ICP vascular length ($n = 6$ per group, unpaired t-test, $P = 0.3686$).
D One-year-old ICP branching point number ($n = 6$ per group, unpaired t-test, $P = 0.9869$).

Data information: Bar graphs depict mean values ± standard deviation, each dot represents one biological replicate. Circles represent females, squares represent males. For details/results of statistical analyses, please see source data. ICP, intermediate capillary plexus; SCP, superficial capillary plexus.
Source data are available online for this figure.
