# Supplementary material for: Sex differences and risk factors for bleeding in Alagille syndrome
Source: EMBO Mol Med. 2022 Nov 8;14(12):e15809. doi: 10.15252/emmm.202215809 (PMC9728057; doi:10.15252/emmm.202215809)
Supplement: Supplementary file 3 — Table EV1 [file EMMM-14-e15809-s008.pdf]

Table EV1

Diagnosis	Sex	Age eye exam (y)	Bilirubin total/ conjugated (µM/L)	Bile acids (µM/L)	INR	Platelet count (10^9/L)	Vascular event (age of event, * denotes age for which blood analysis at right is described)	Bilirubin total/ conjugated (µM/L)	Bile acids (µM/L)	INR	Platelet count (10^9/L)	Follow up until (y)
reference value			16/4	< 7	< 1.2	200-400		16/4	< 7	< 1.2	200-400	
ALGS	F	9	76/58	239	1.21	141	Surgery-related hemorrhage (10*), nosebleed (15), GI bleed (Grade 1 at 10, Grade 4 at 17), splenic infarction (18)	58/39	239	1.38	117	18
ALGS	F	10	6/1	11	1.02	281	Nosebleed (10*)	6/1	11	1.02	281	18
ALGS	F	12	9/2	25	1	267	N/A					18
ALGS	F	2	6/<2	3	1	486	N/A					6
ALGS	F	5	4/<2	3	1	295	N/A					13
ALGS	M	4	5/<2	3	1	334	Coagulopathy-related bleed (1m*)	62/37	79	>8	417	4
BA	F	8	24/8	285	1.14	65	Bruising (9*), esophageal varices (10)	32/16	312	1.17	60	18
BA	M	15					N/A					18
BA	F	11	8/1	4	0.99	141	Nosebleed, GI bleeding, bruising (all three events at age 14* and 15)	6/1		0.9	222	18
BA	F	10	11/3		1.2	67	GI bleeding (7*)	5/1		1.16	58	18
