# Supplementary figures and images for: Sex differences and risk factors for bleeding in Alagille syndrome
Source: EMBO Mol Med. 2022 Nov 8;14(12):e15809. doi: 10.15252/emmm.202215809 (PMC9728057; doi:10.15252/emmm.202215809)

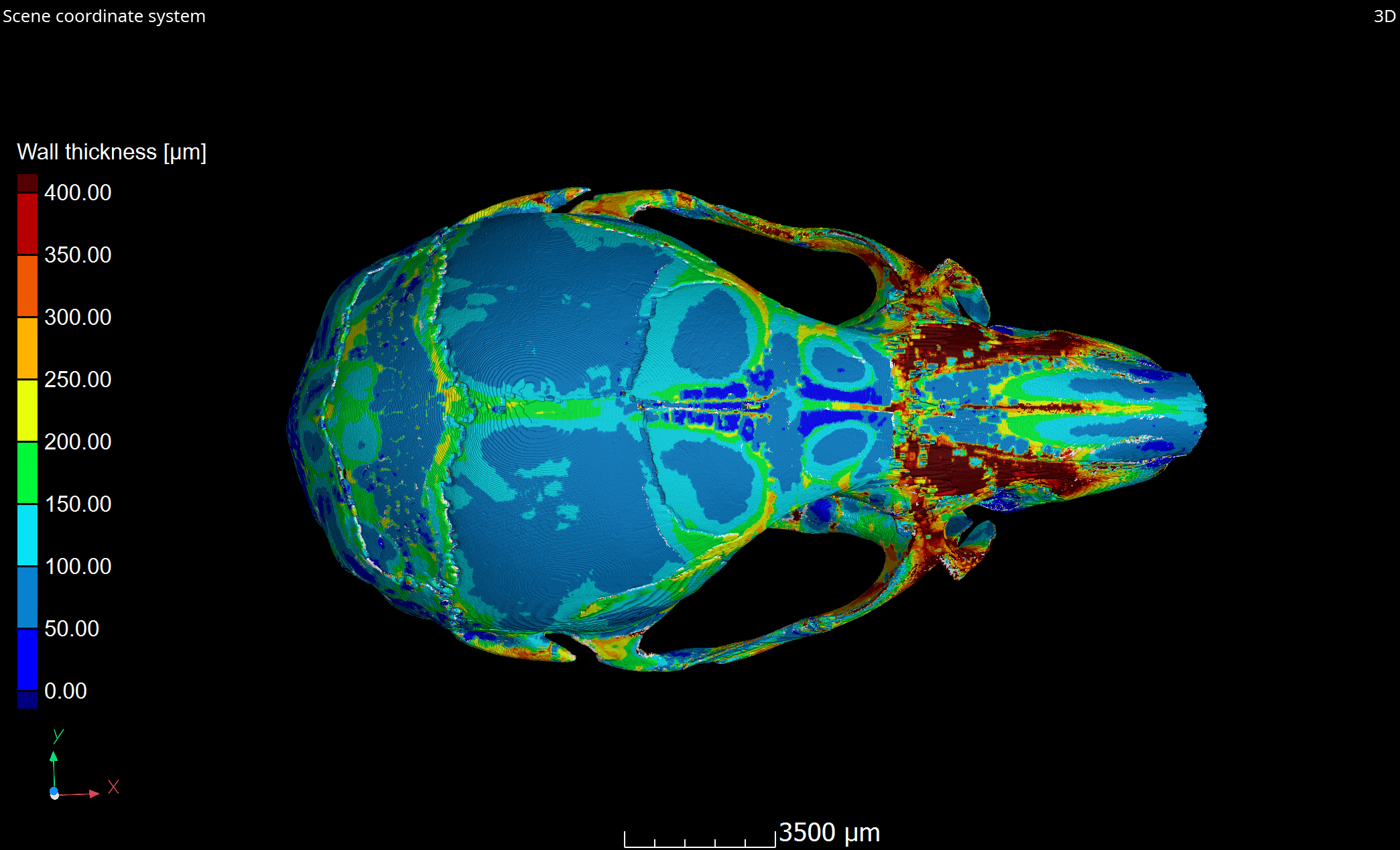

Supplement: Supplementary file 8 — Source Data for Figure 2 [file EMMM-14-e15809-s005.zip › Figure 2 Source Data/Figure 2G skull/2G NDR_female_P30_1428_Full_thickness.bmp]

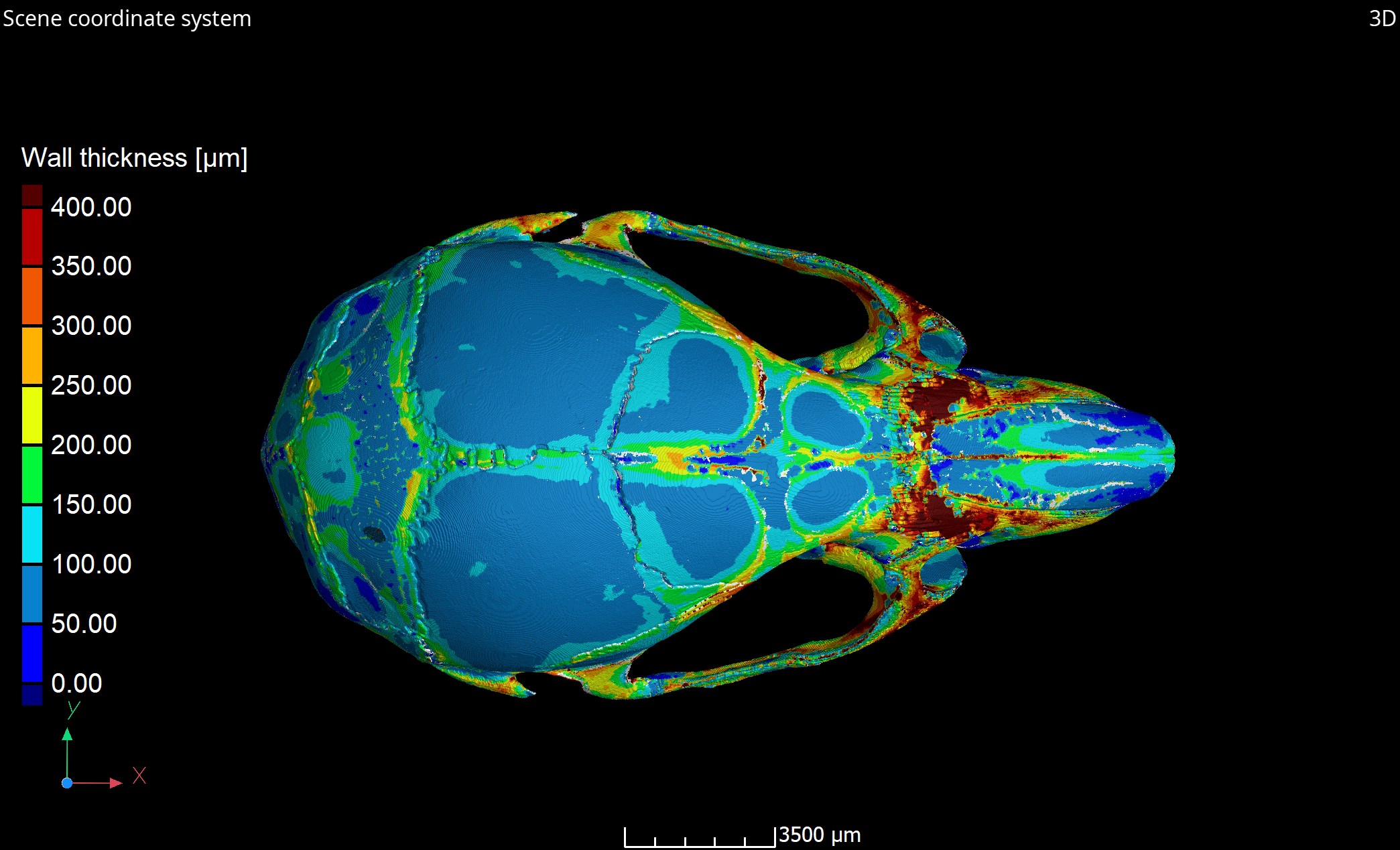

Supplement: Supplementary file 8 — Source Data for Figure 2 [file EMMM-14-e15809-s005.zip › Figure 2 Source Data/Figure 2G skull/2G NDR_male_P30_1327_Full_thickness.bmp]

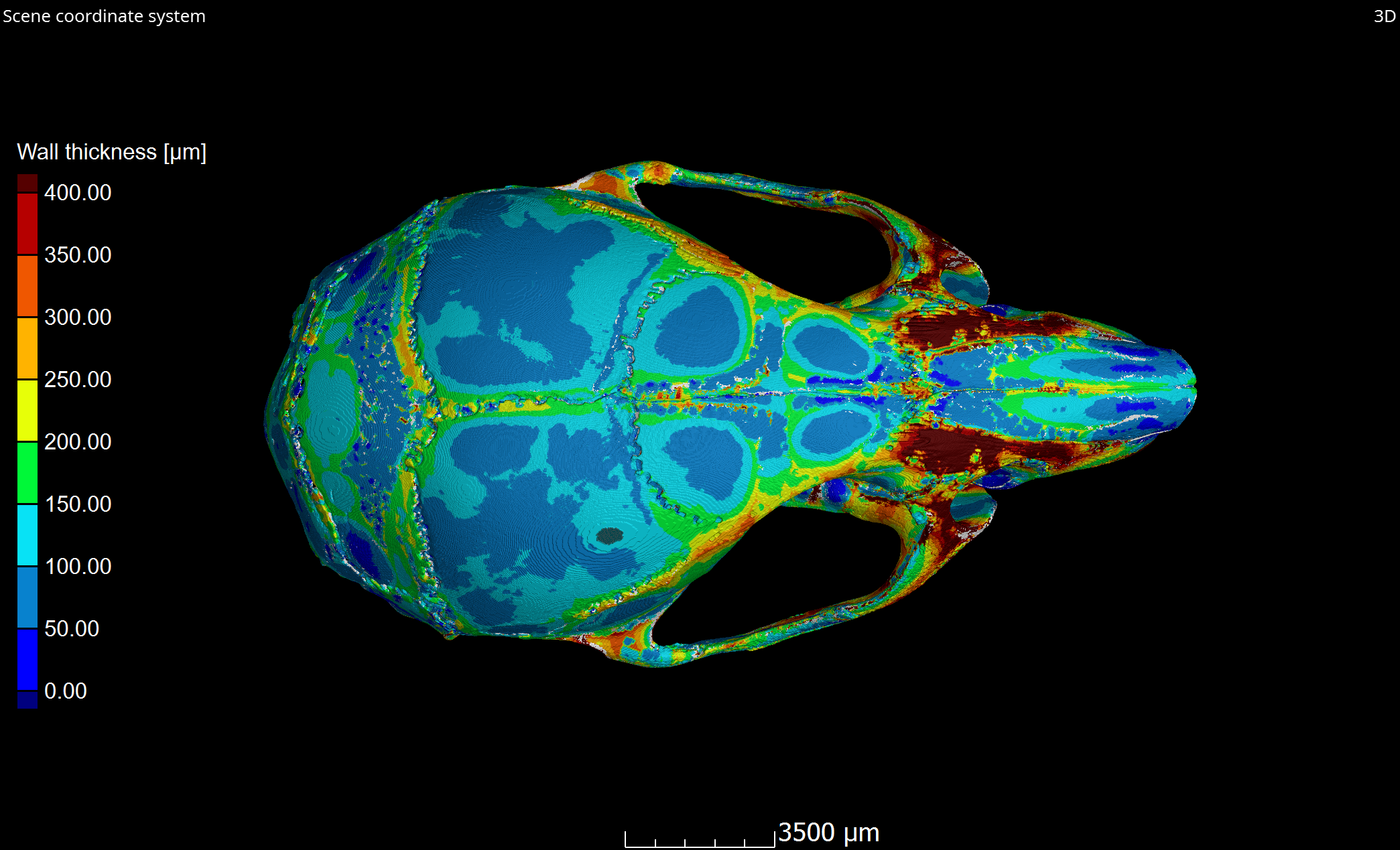

Supplement: Supplementary file 8 — Source Data for Figure 2 [file EMMM-14-e15809-s005.zip › Figure 2 Source Data/Figure 2G skull/2G WT_female_P30_1265_Full_thickness.bmp]

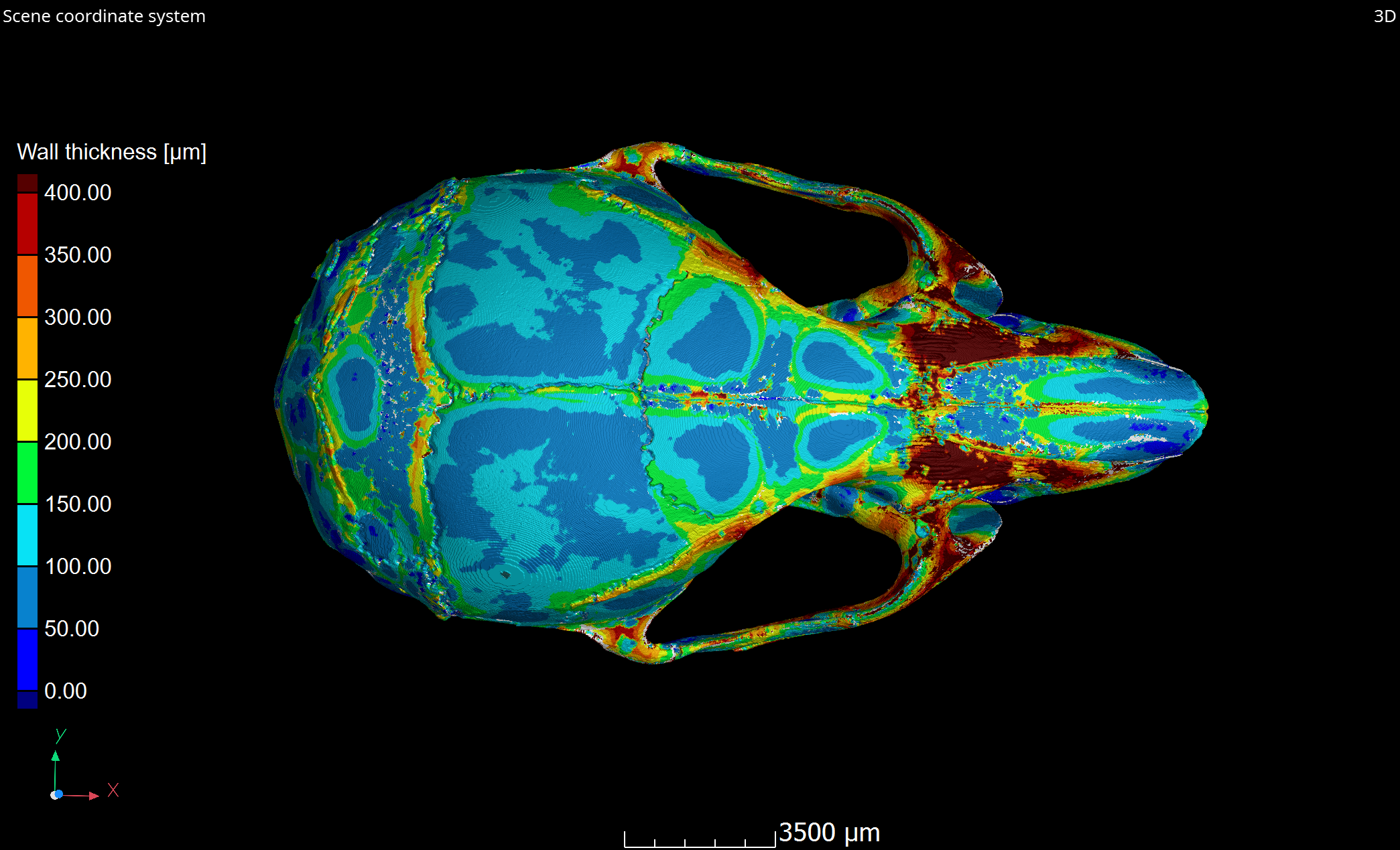

Supplement: Supplementary file 8 — Source Data for Figure 2 [file EMMM-14-e15809-s005.zip › Figure 2 Source Data/Figure 2G skull/2G WT_male_P30_1262_Full_thickness.bmp]

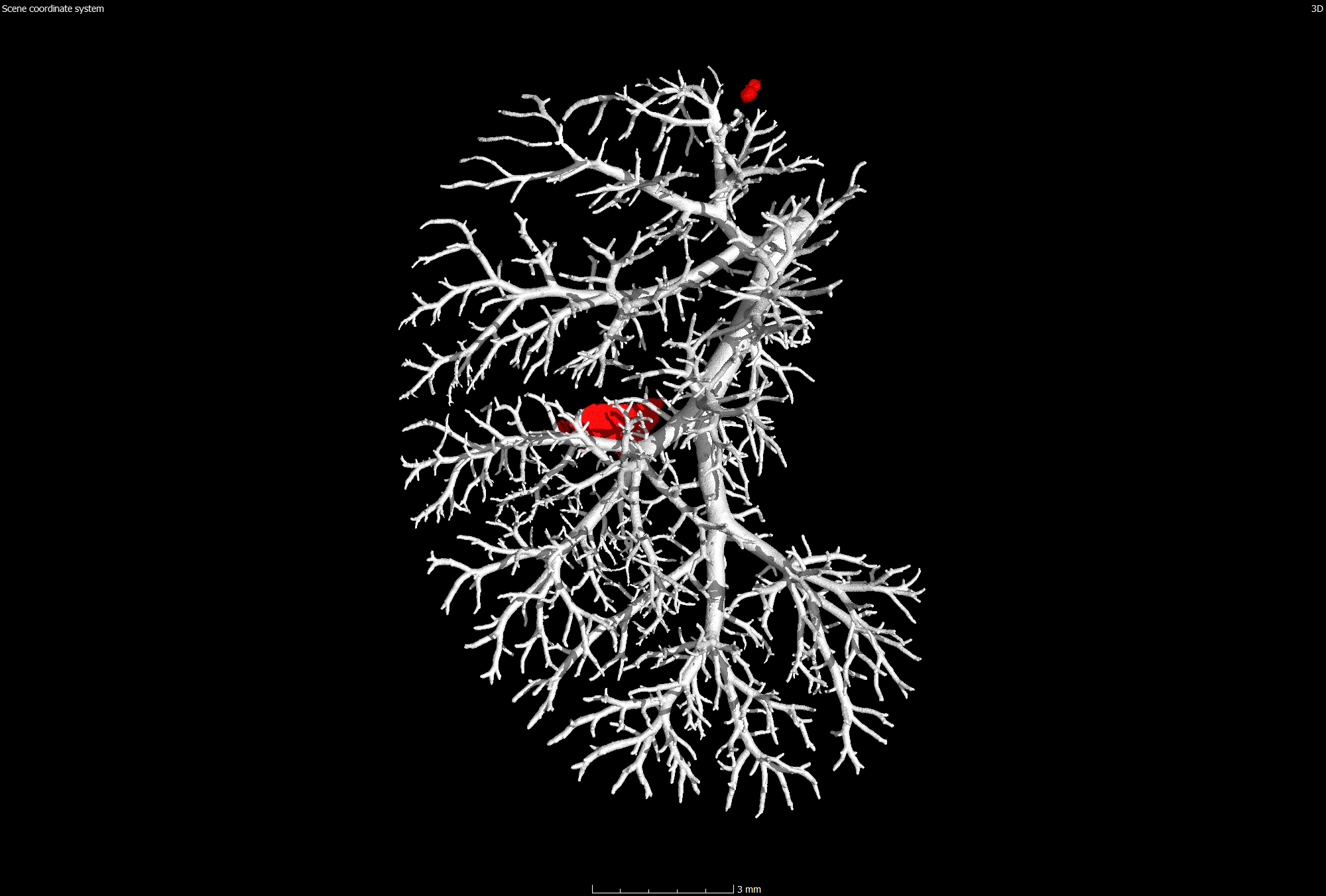

Supplement: Supplementary file 8 — Source Data for Figure 2 [file EMMM-14-e15809-s005.zip › Figure 2 Source Data/Figure 2L liver rupture/Adult_liver_NDR_2369_PV+BD_casting.tif]

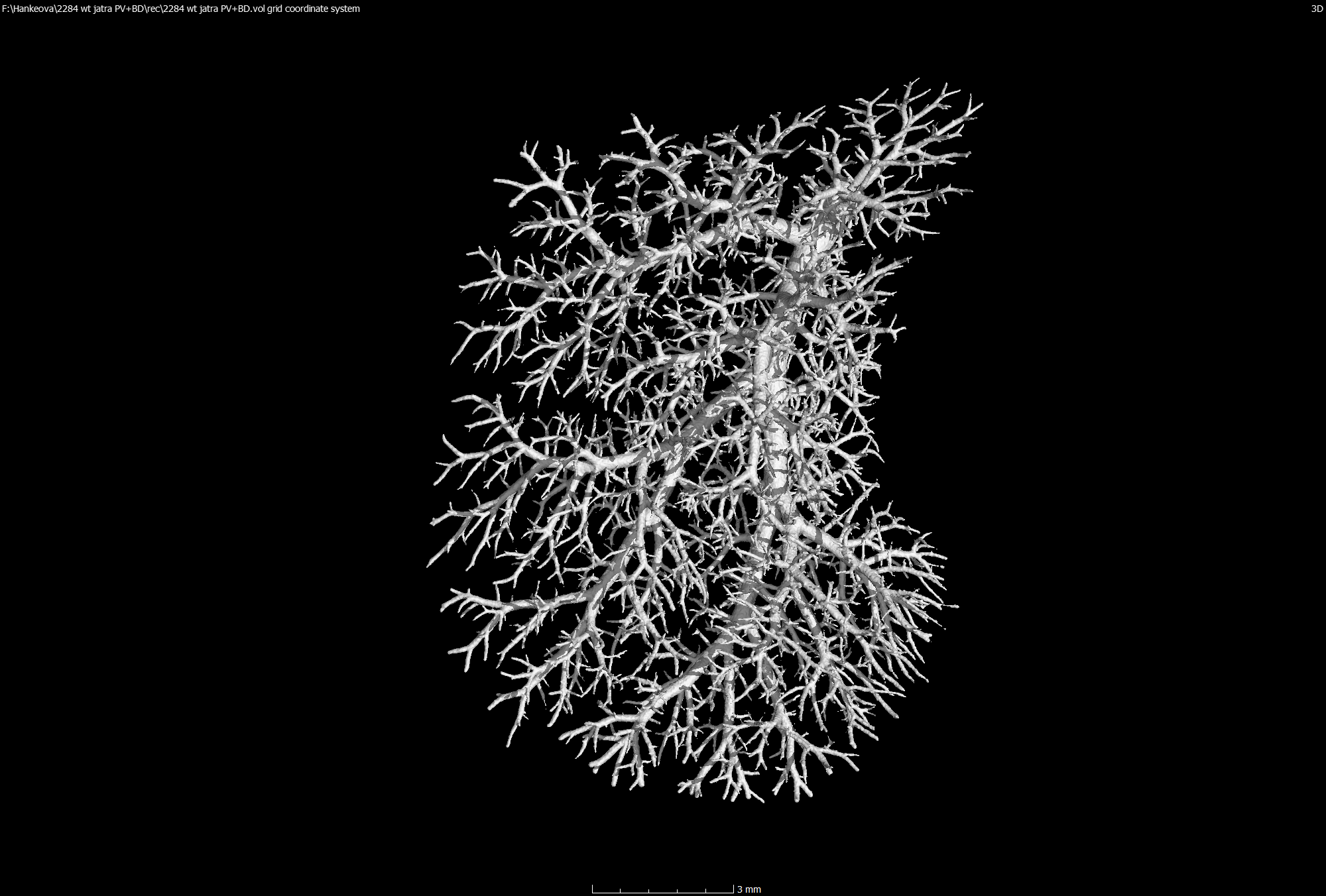

Supplement: Supplementary file 8 — Source Data for Figure 2 [file EMMM-14-e15809-s005.zip › Figure 2 Source Data/Figure 2L liver rupture/Adult_liver_WT_2284_PV+BD_casting.tif]

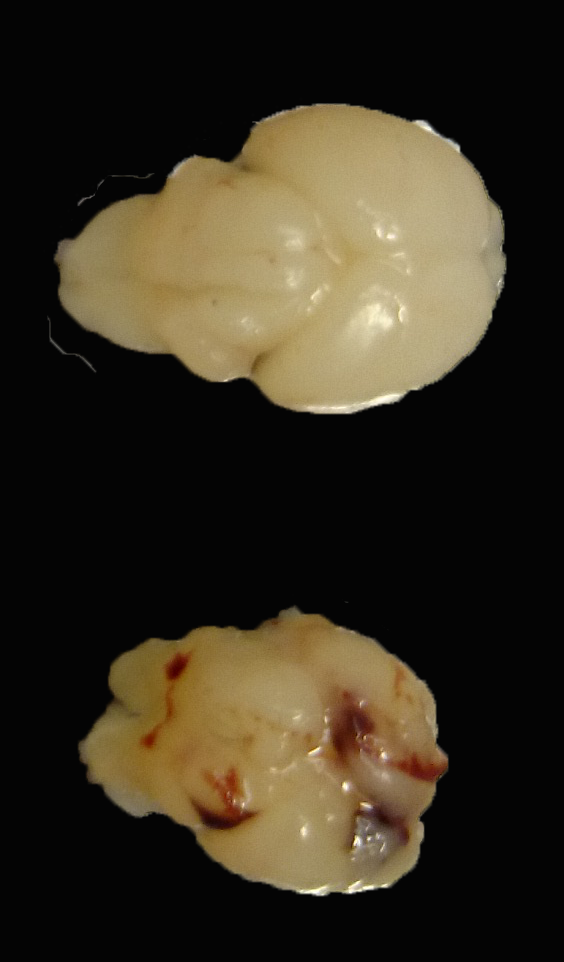

Supplement: Supplementary file 8 — Source Data for Figure 2 [file EMMM-14-e15809-s005.zip › Figure 2 Source Data/Figure 2O brain bleed/Fig2O_brain bleed DSC_0561.tif]

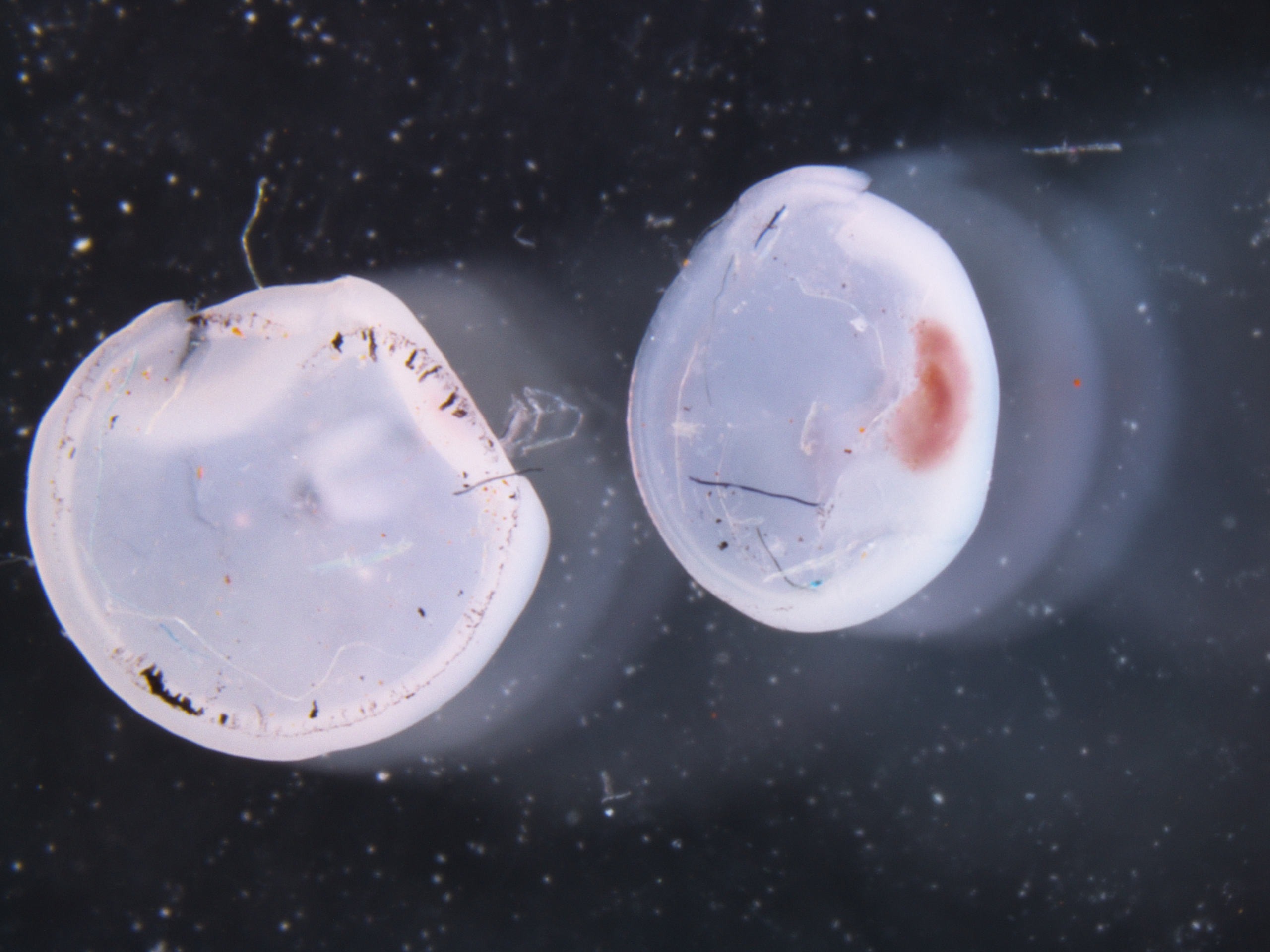

Supplement: Supplementary file 8 — Source Data for Figure 2 [file EMMM-14-e15809-s005.zip › Figure 2 Source Data/Figure 2P retina bleed/Fig2P_WT_NDR_P15 retina black.tif]

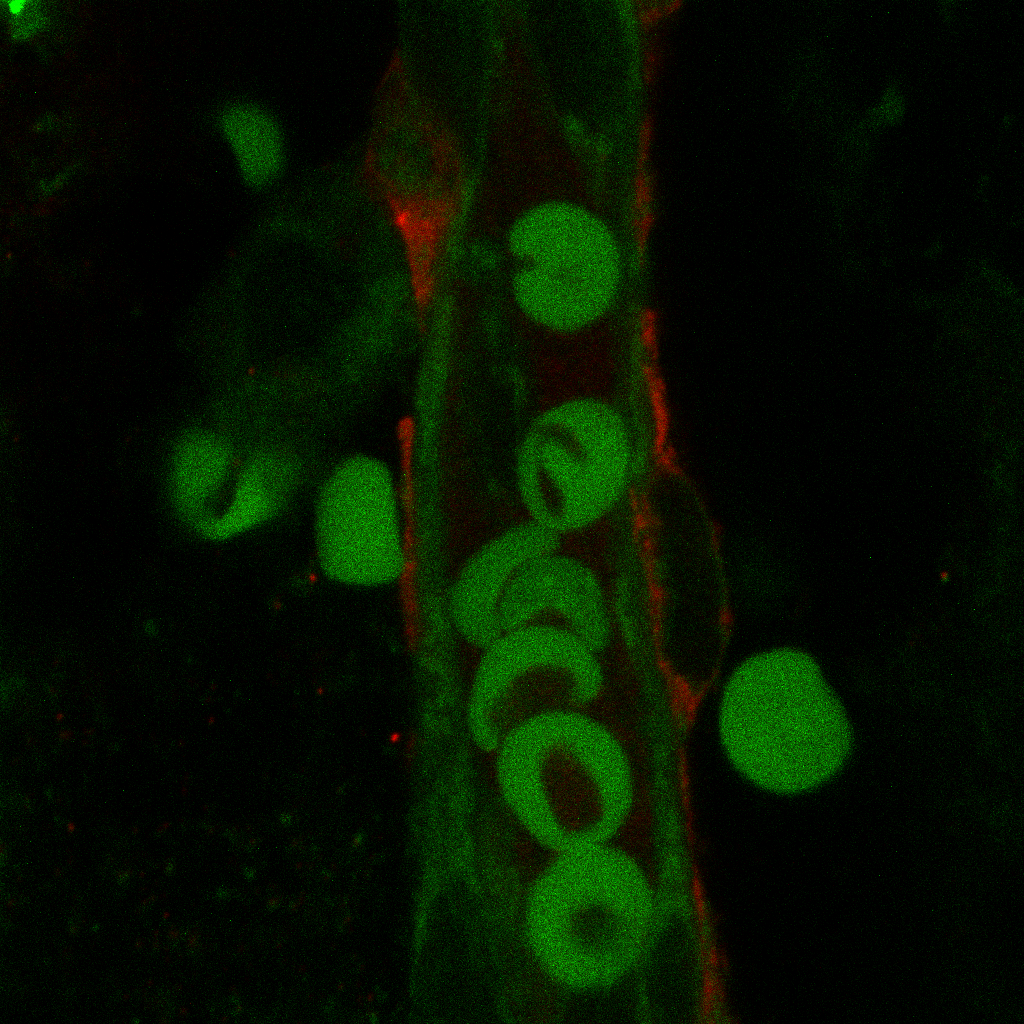

Supplement: Supplementary file 8 — Source Data for Figure 2 [file EMMM-14-e15809-s005.zip › Figure 2 Source Data/Figure 2Q retina red blood cells/Fig2Q NDR P10_retina.tif]

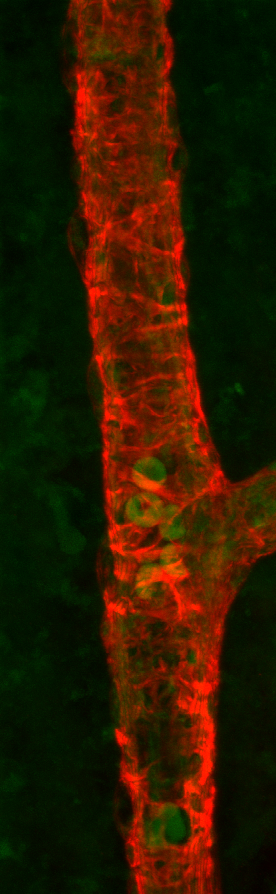

Supplement: Supplementary file 8 — Source Data for Figure 2 [file EMMM-14-e15809-s005.zip › Figure 2 Source Data/Figure 2Q retina red blood cells/Fig2Q WT P10_retina.tif]

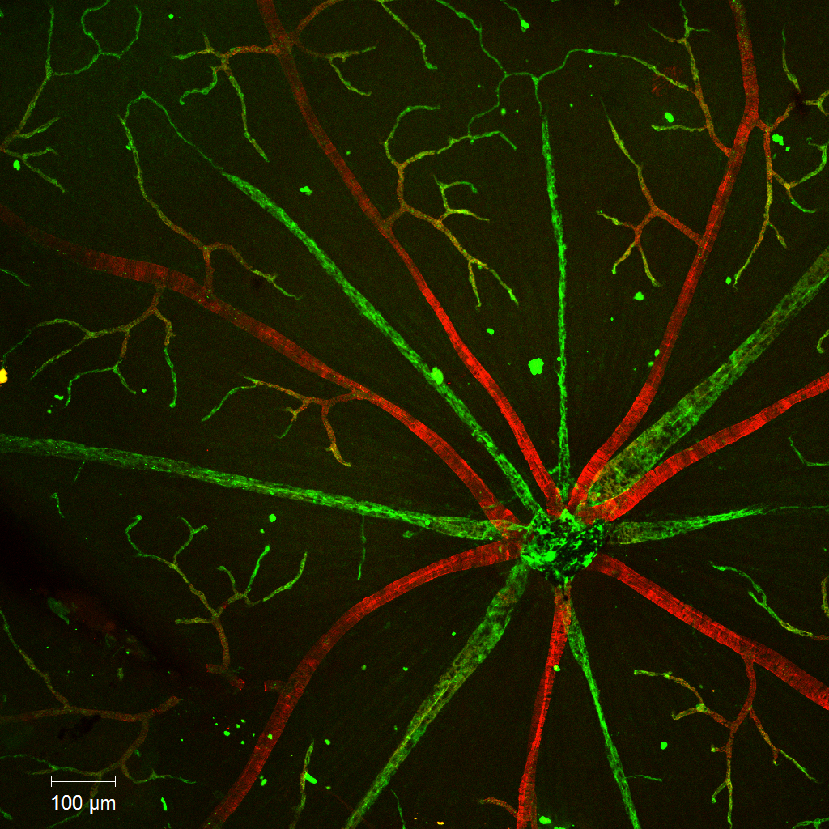

Supplement: Supplementary file 9 — Source Data for Figure 3 [file EMMM-14-e15809-s010.zip › Figure 3 Source Data/Figure 3A WT_4854_asma cd31 10x.tif]

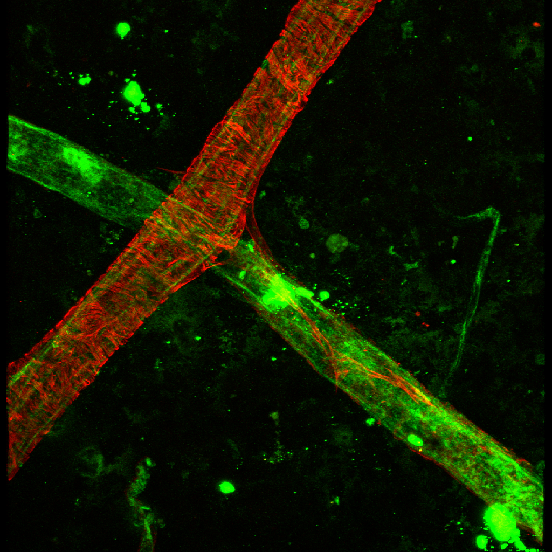

Supplement: Supplementary file 9 — Source Data for Figure 3 [file EMMM-14-e15809-s010.zip › Figure 3 Source Data/Figure 3A_crossing_NDR_5575 asma cd31 63x.tif]

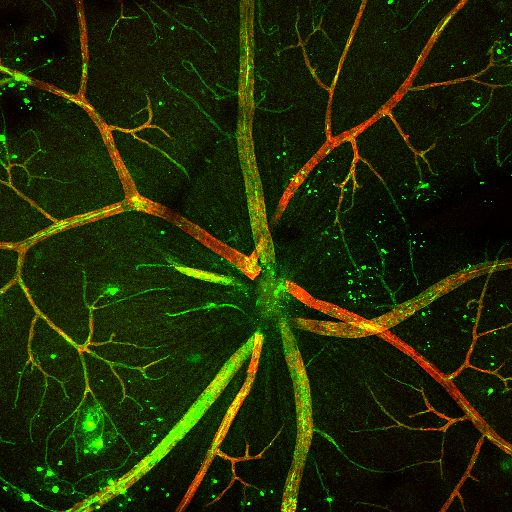

Supplement: Supplementary file 9 — Source Data for Figure 3 [file EMMM-14-e15809-s010.zip › Figure 3 Source Data/Figure 3A_NDR_4830 asma cd31 10x .tif]

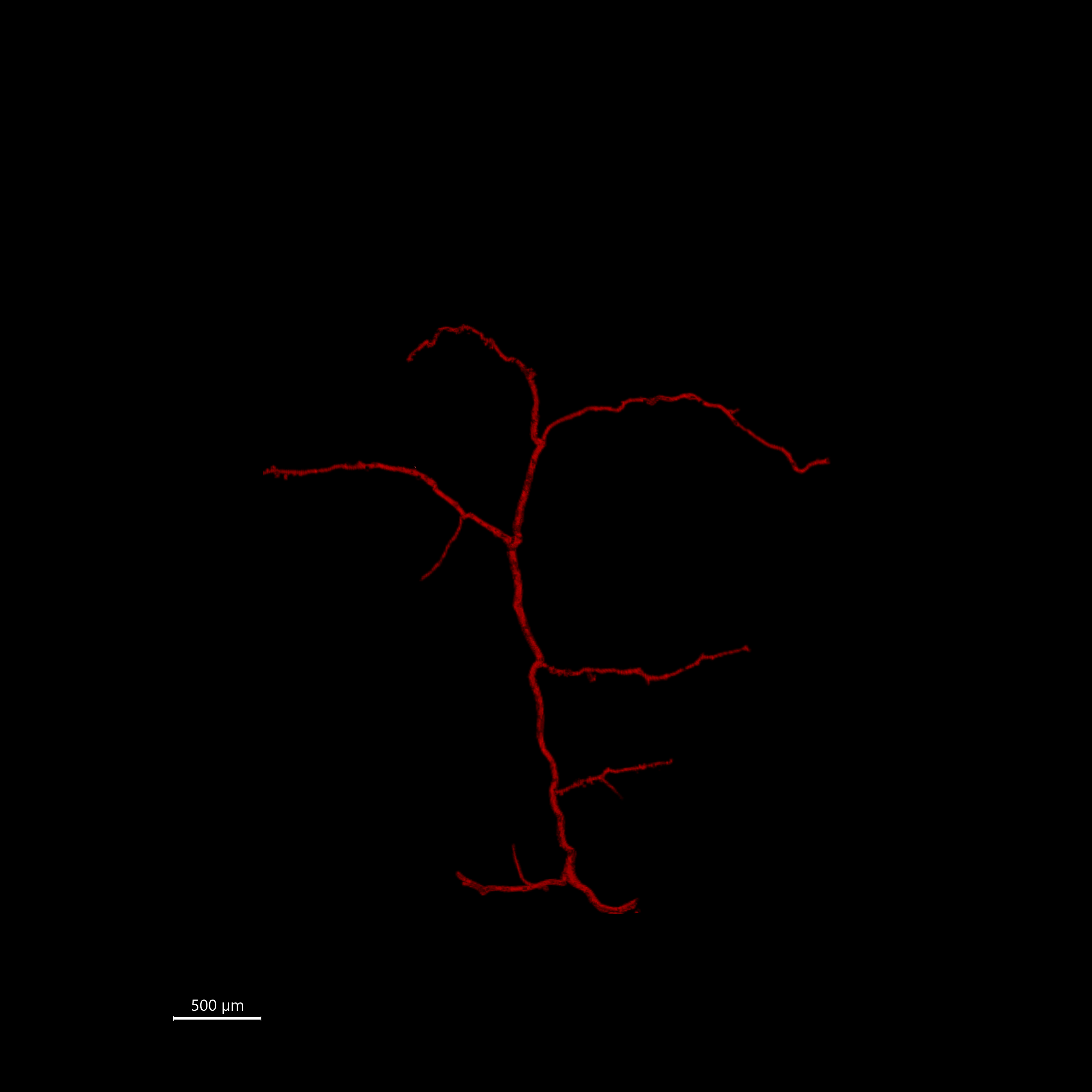

Supplement: Supplementary file 9 — Source Data for Figure 3 [file EMMM-14-e15809-s010.zip › Figure 3 Source Data/Figure 3K_Ndr_aSMA_MCA.tif]

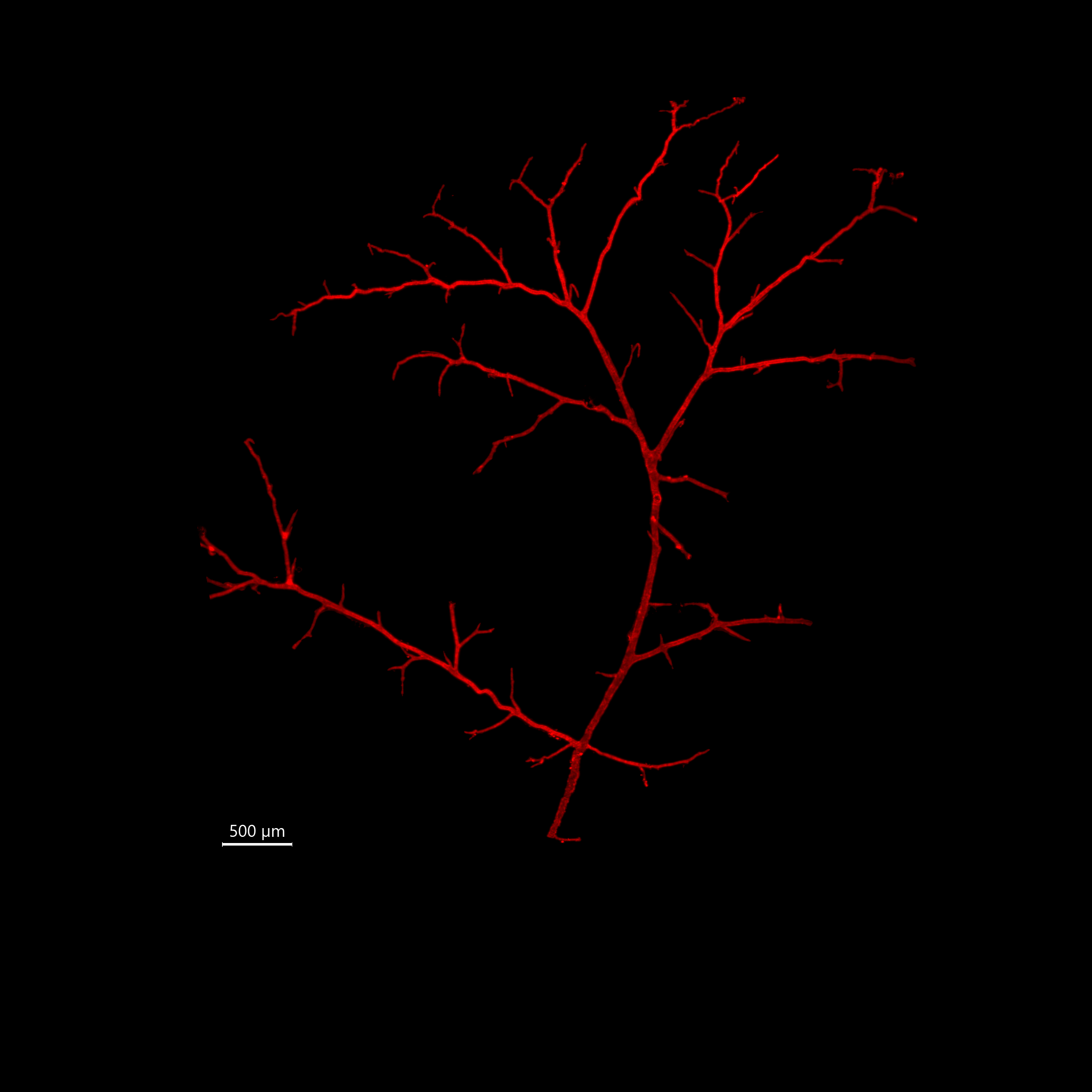

Supplement: Supplementary file 9 — Source Data for Figure 3 [file EMMM-14-e15809-s010.zip › Figure 3 Source Data/Figure 3K_wt_aSMA_MCA.tif]

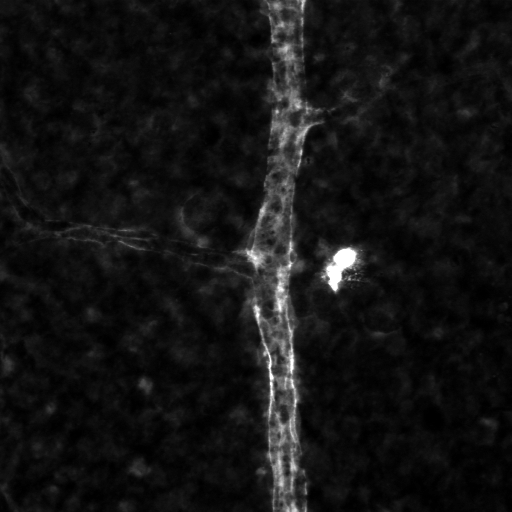

Supplement: Supplementary file 10 — Source Data for Figure 4 [file EMMM-14-e15809-s011.zip › Figure 4 Source Data/Figure 4A P10/Fig4A_NDR_1022-3_40xartery10_adjusted.tif]

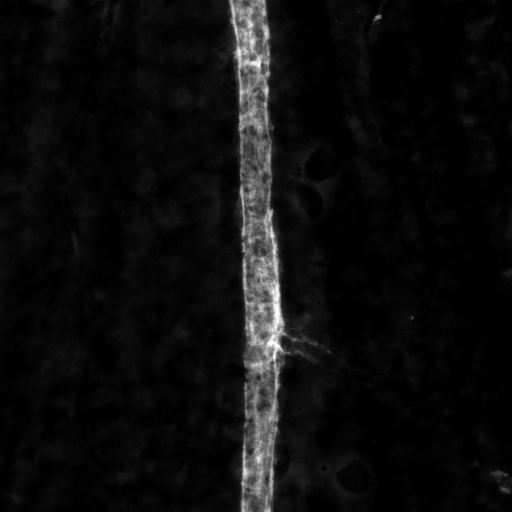

Supplement: Supplementary file 10 — Source Data for Figure 4 [file EMMM-14-e15809-s011.zip › Figure 4 Source Data/Figure 4A P10/Fig4A_WT_1022-1_40xartery1_adjusted.tif]

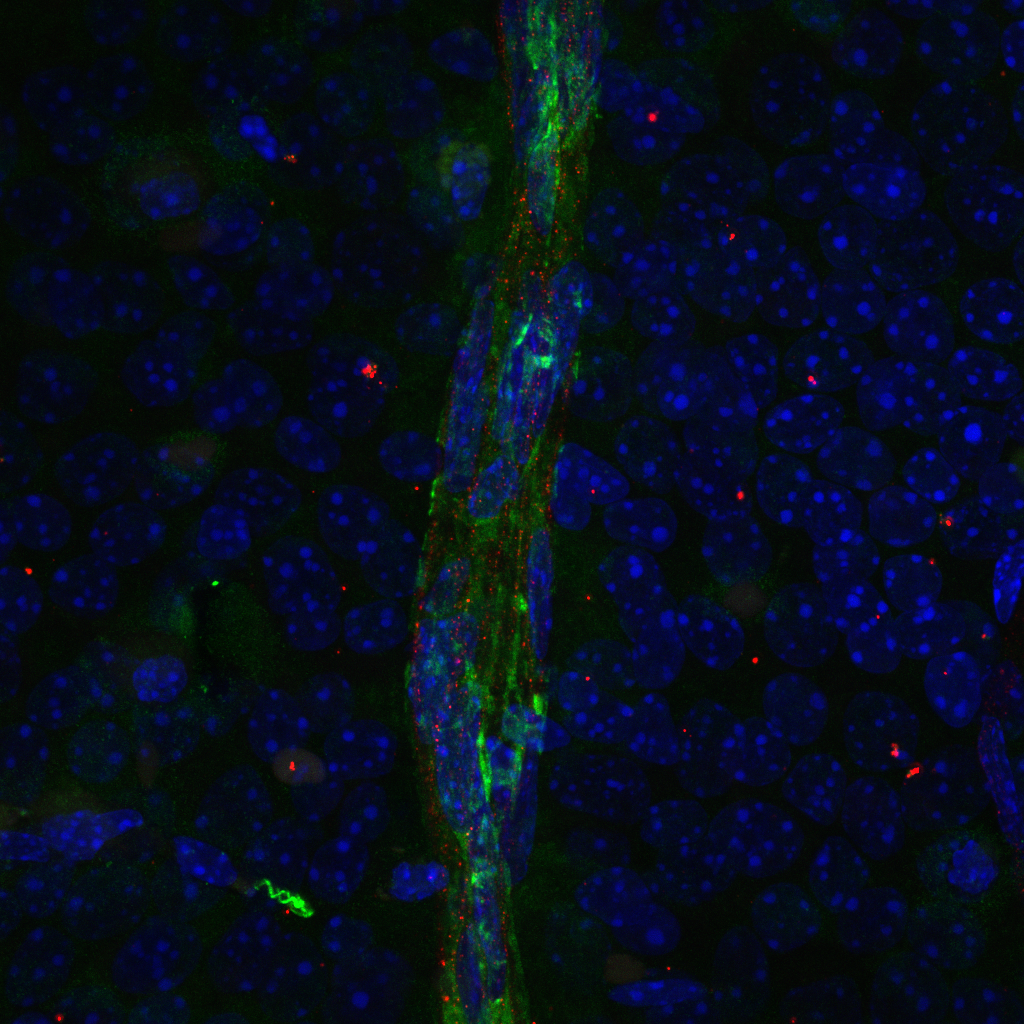

Supplement: Supplementary file 10 — Source Data for Figure 4 [file EMMM-14-e15809-s011.zip › Figure 4 Source Data/Figure 4B P15/Fig4B_NDR_P15 asma cd31 63x1_merged.tif]

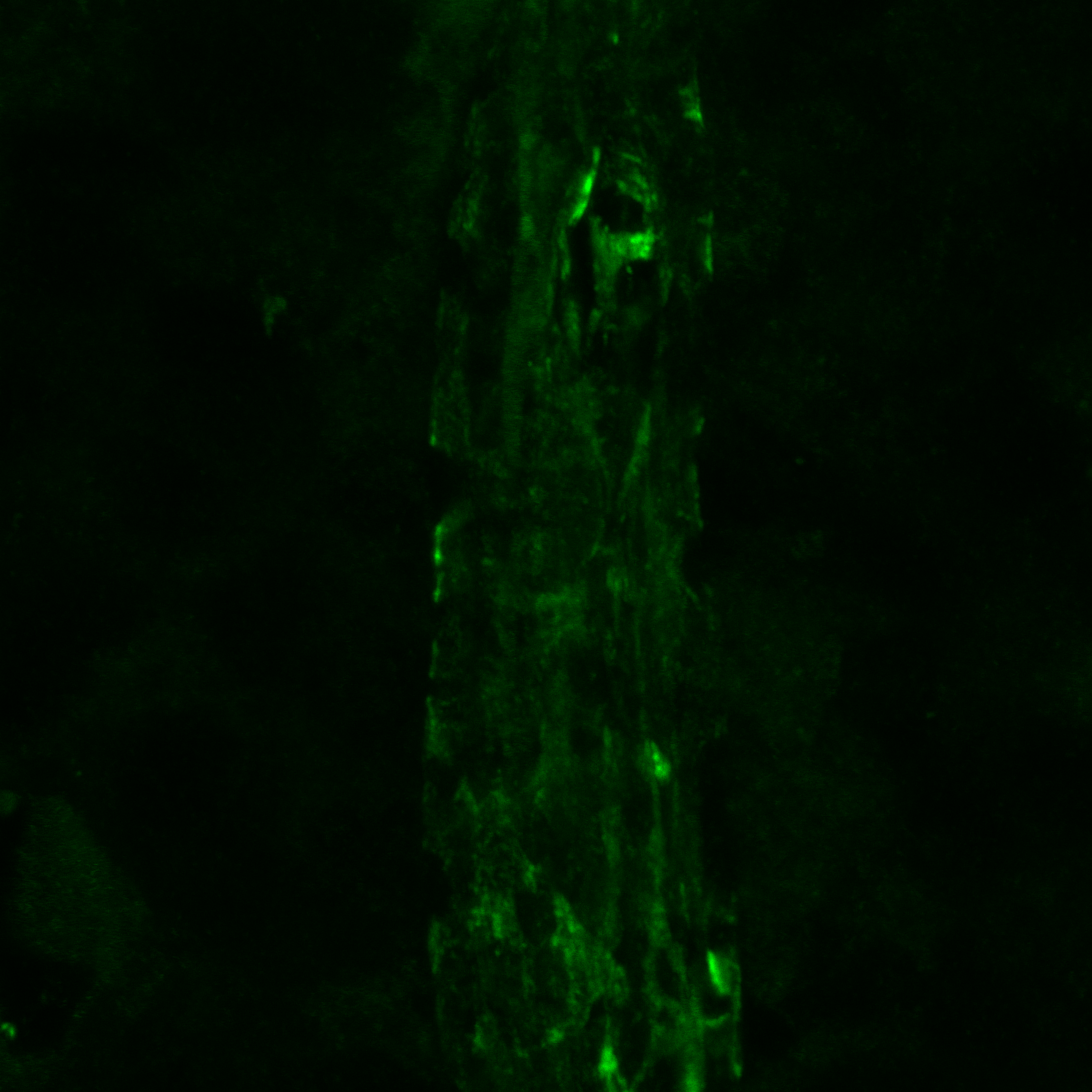

Supplement: Supplementary file 10 — Source Data for Figure 4 [file EMMM-14-e15809-s011.zip › Figure 4 Source Data/Figure 4B P15/Fig4B_NDR_P15 asma cd31 63x2.tif]

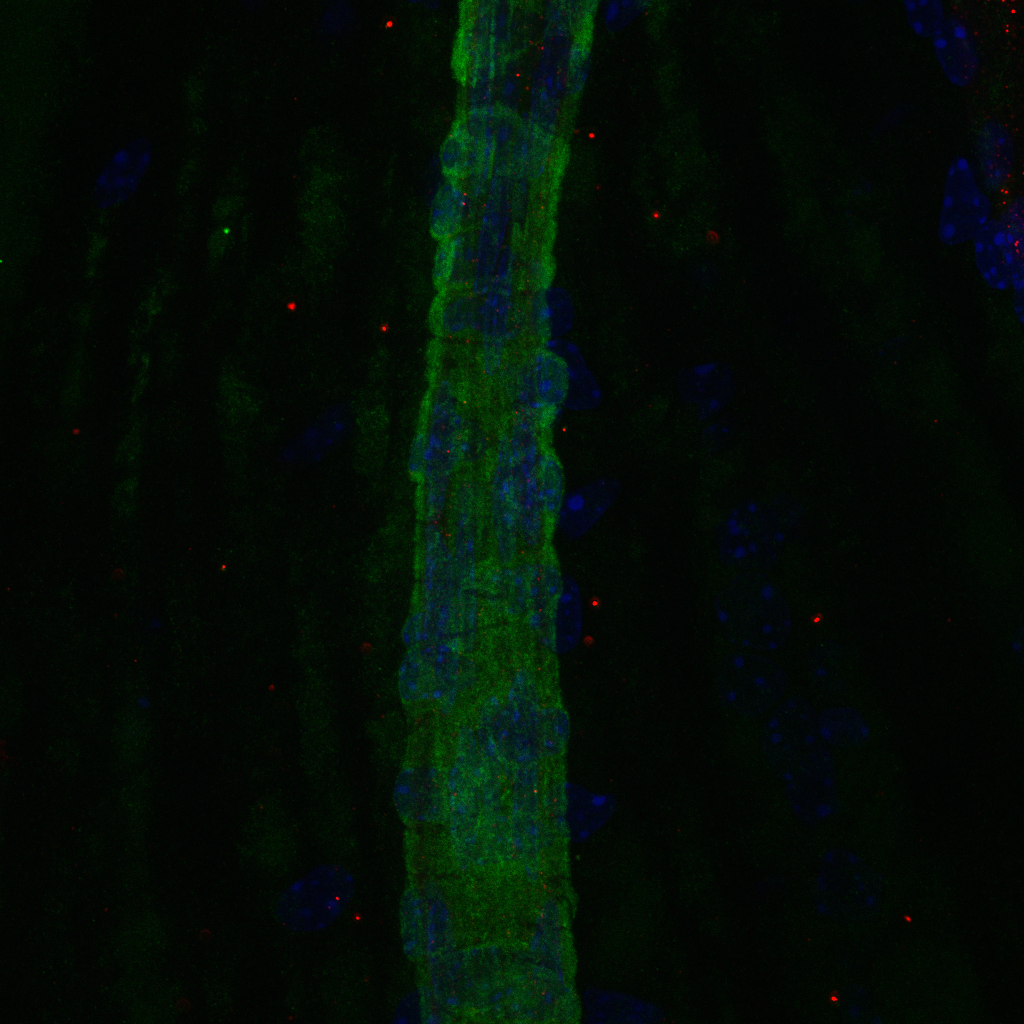

Supplement: Supplementary file 10 — Source Data for Figure 4 [file EMMM-14-e15809-s011.zip › Figure 4 Source Data/Figure 4B P15/Fig4B_wt_P15 asma cd31 63x1_merged.tif]

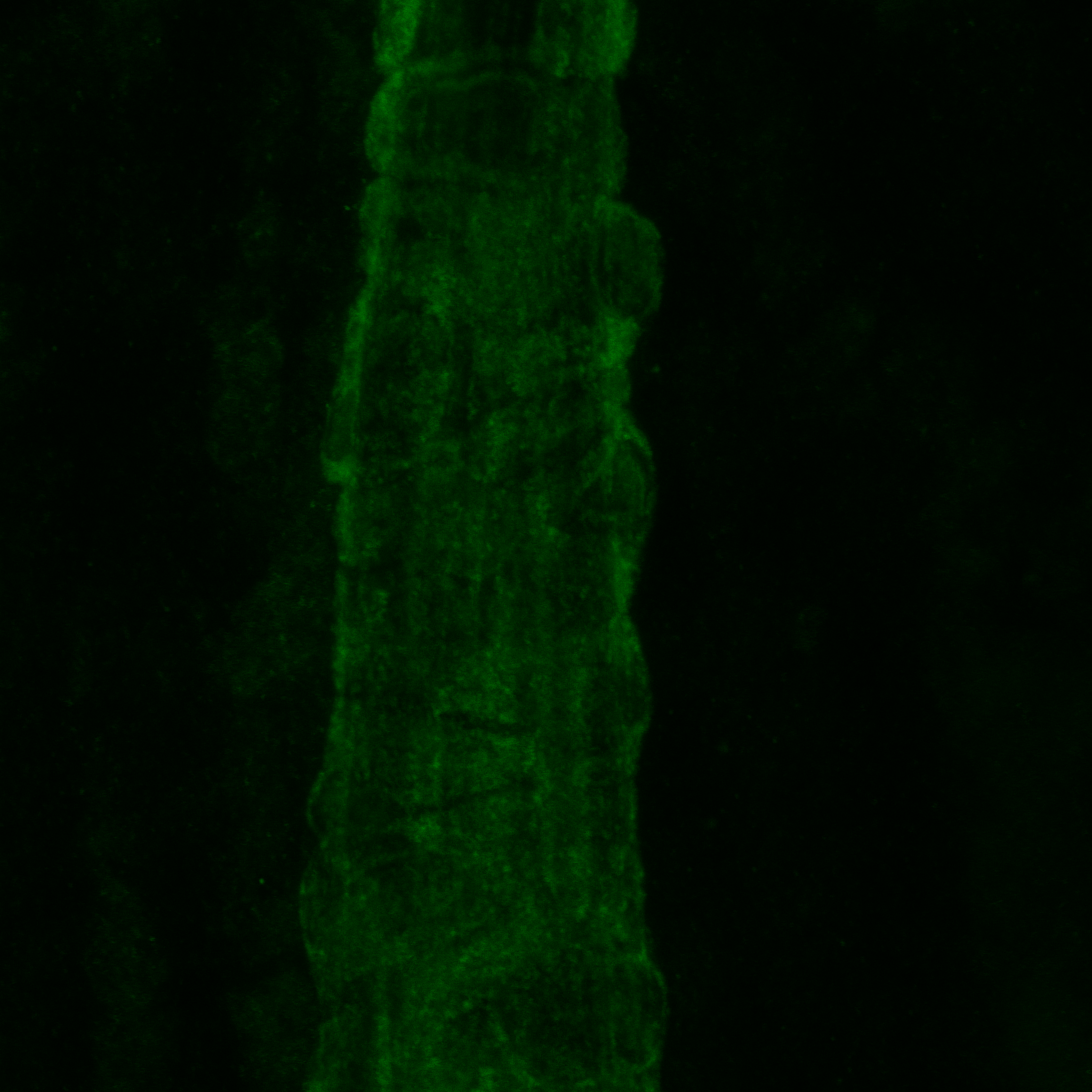

Supplement: Supplementary file 10 — Source Data for Figure 4 [file EMMM-14-e15809-s011.zip › Figure 4 Source Data/Figure 4B P15/Fig4B_wt_P15 asma cd31 63x2.tif]

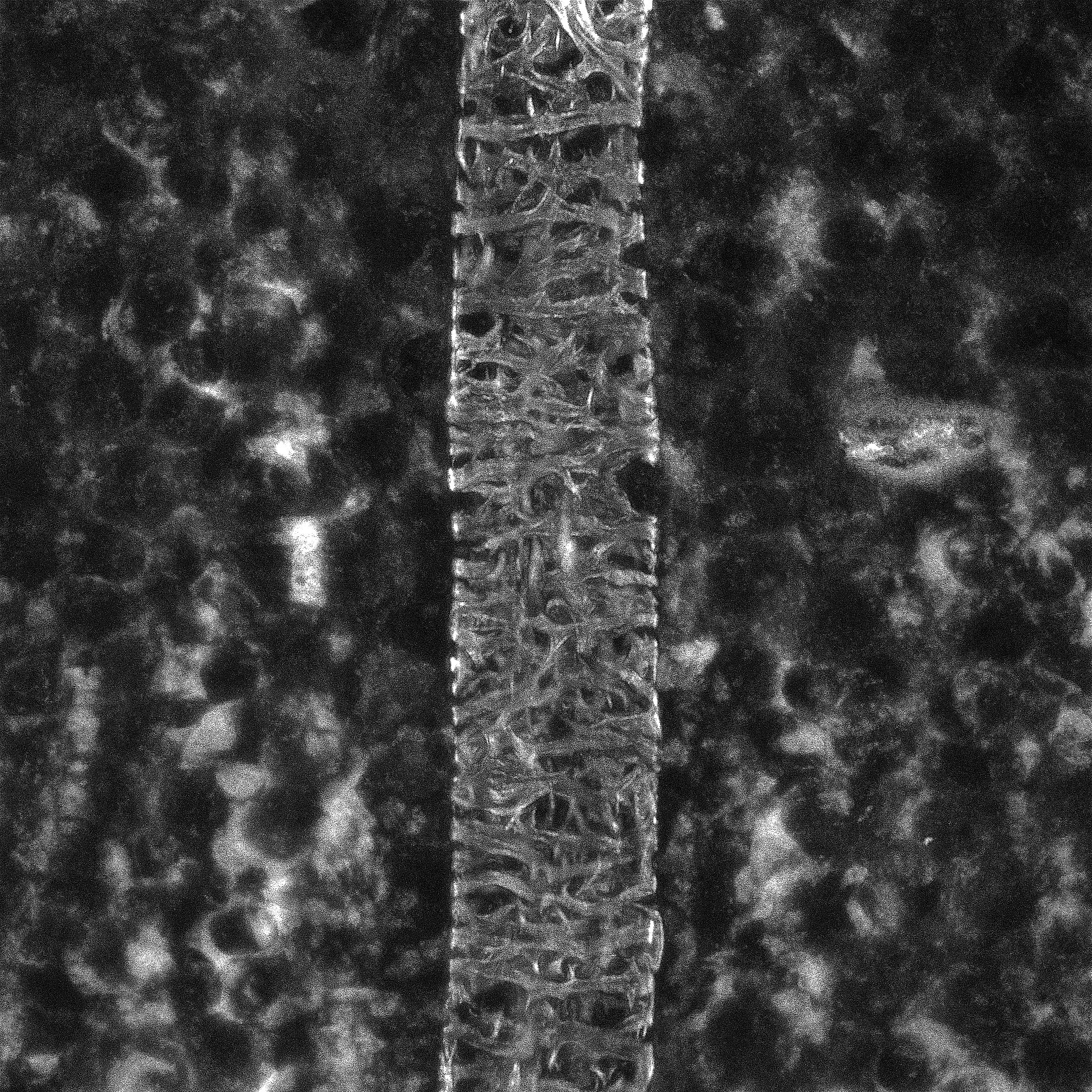

Supplement: Supplementary file 10 — Source Data for Figure 4 [file EMMM-14-e15809-s011.zip › Figure 4 Source Data/Figure 4C P30/Fig4C_NDR_2014_aSMA_63x_3-1.tif]

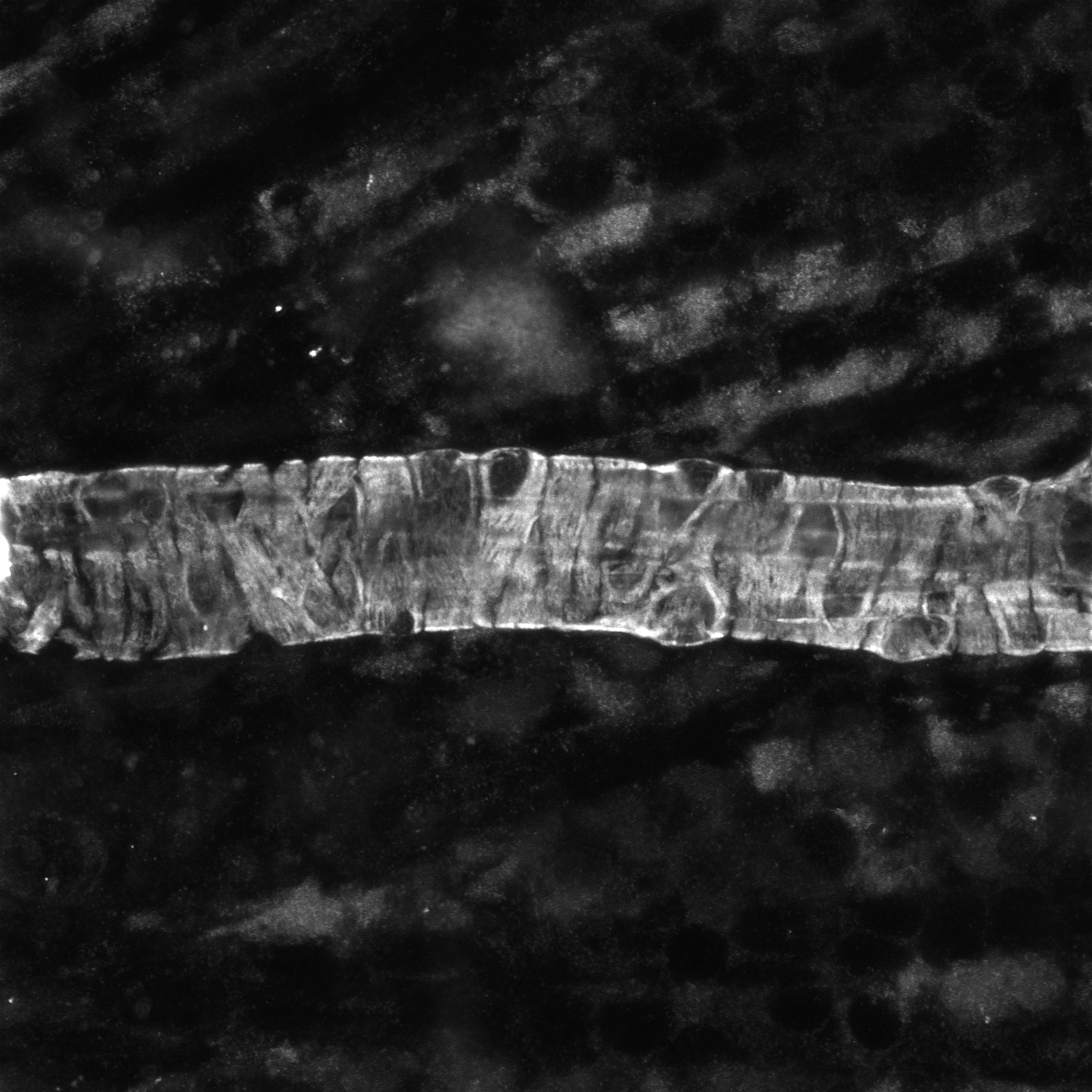

Supplement: Supplementary file 10 — Source Data for Figure 4 [file EMMM-14-e15809-s011.zip › Figure 4 Source Data/Figure 4C P30/Fig4C_WT_2017_aSMA_63x_2.tif]

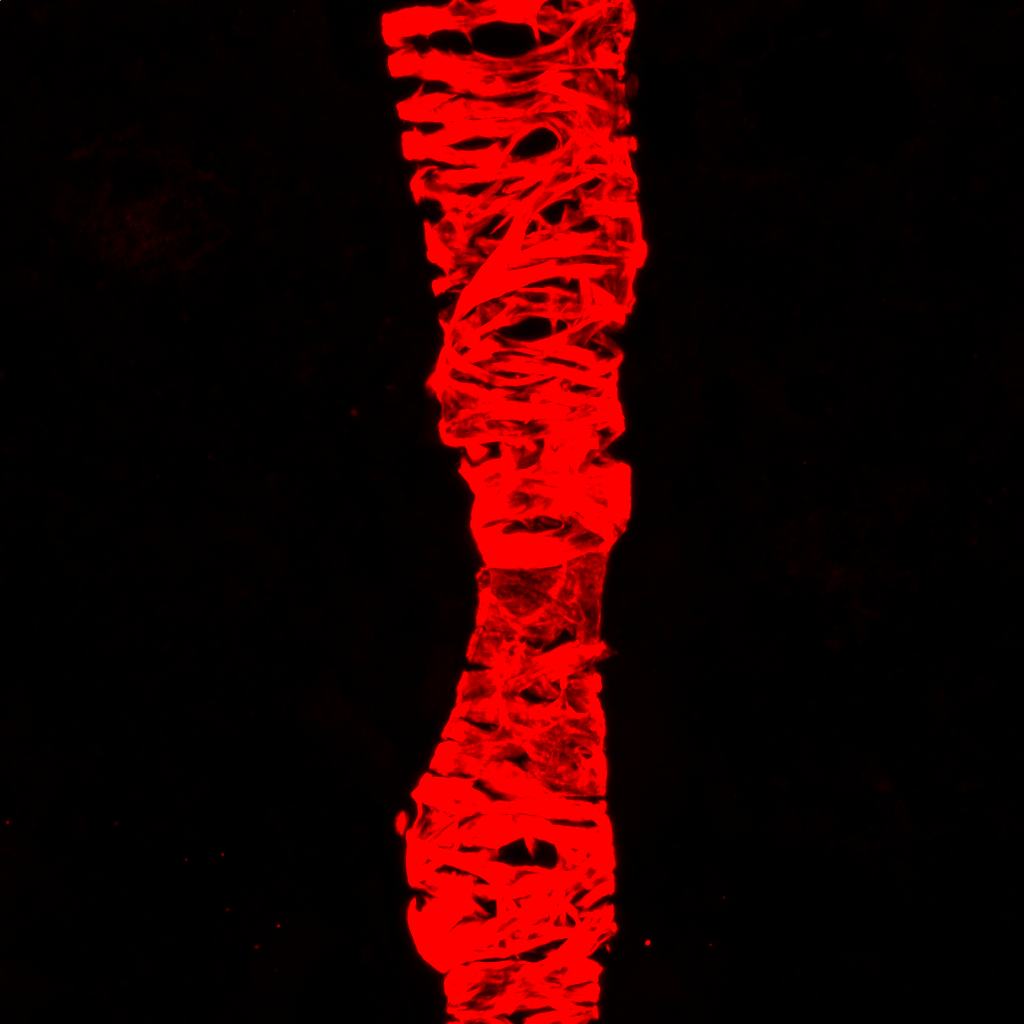

Supplement: Supplementary file 10 — Source Data for Figure 4 [file EMMM-14-e15809-s011.zip › Figure 4 Source Data/Figure 4D adult/Fig4D_NDR_ adult 2395 aSMA 63x (RGB).tif]

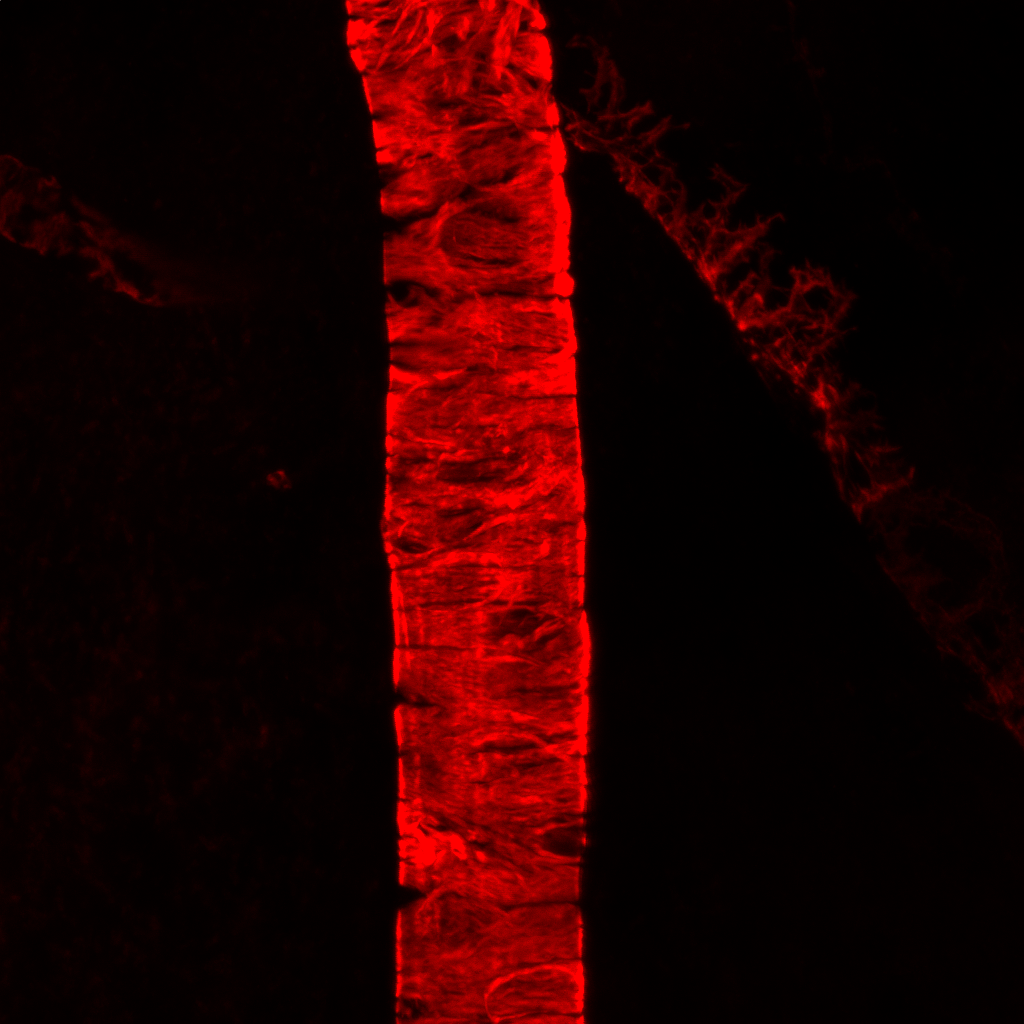

Supplement: Supplementary file 10 — Source Data for Figure 4 [file EMMM-14-e15809-s011.zip › Figure 4 Source Data/Figure 4D adult/Fig4D_WT_adult 614 63x aSMA.tif]

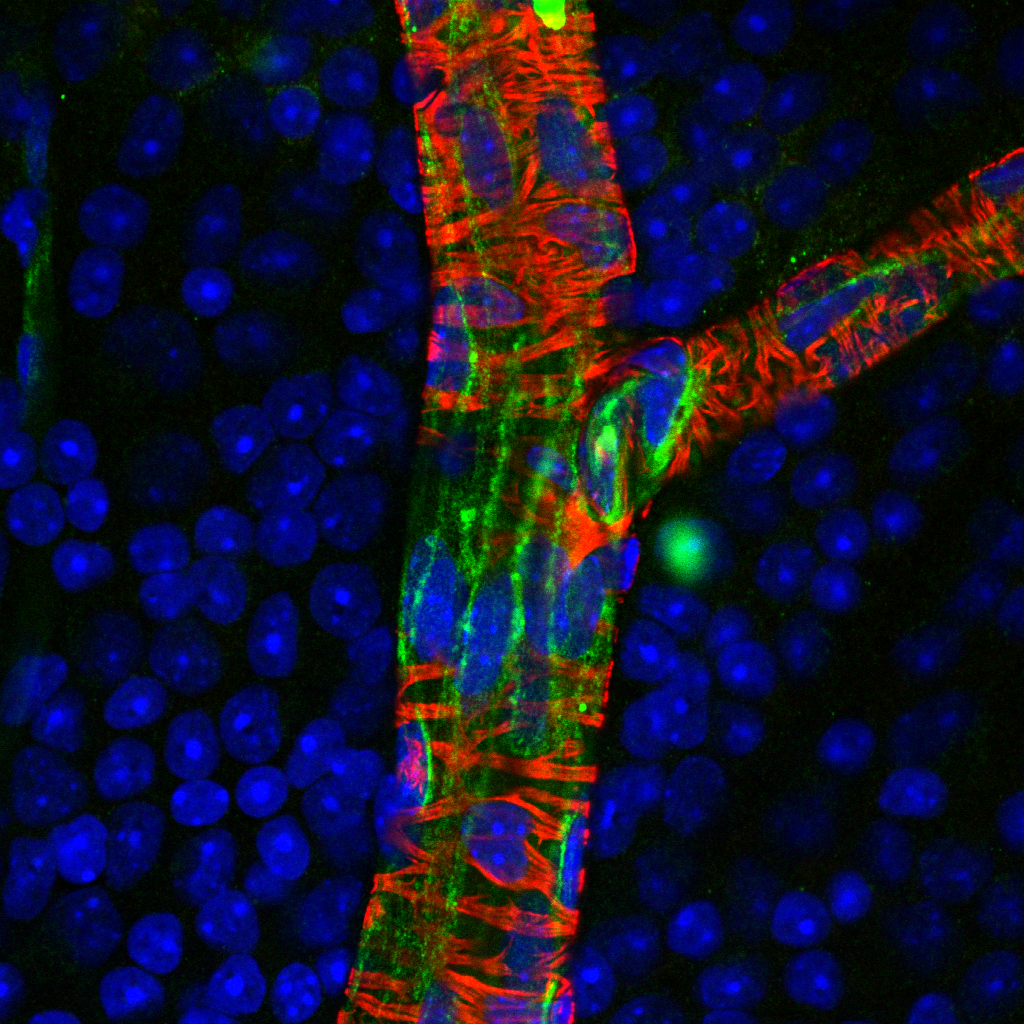

Supplement: Supplementary file 10 — Source Data for Figure 4 [file EMMM-14-e15809-s011.zip › Figure 4 Source Data/Figure 4E 1 year/Fig4E_NDR_5575_aSMA-CD31_63x_VSMCmissing (RGB).tif]

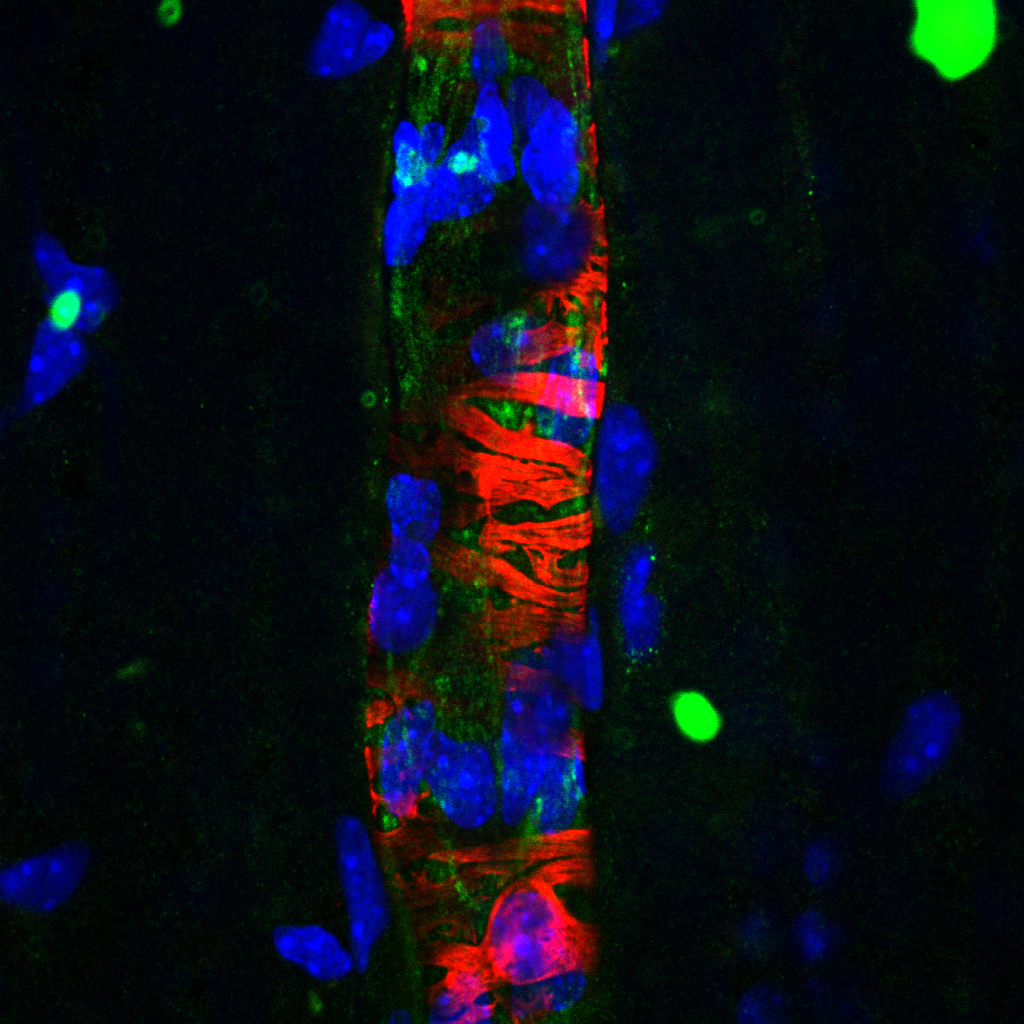

Supplement: Supplementary file 10 — Source Data for Figure 4 [file EMMM-14-e15809-s011.zip › Figure 4 Source Data/Figure 4E 1 year/Fig4E_NDR_5575_aSMA-CD31_63x_VSMCmissing3 (RGB).tif]

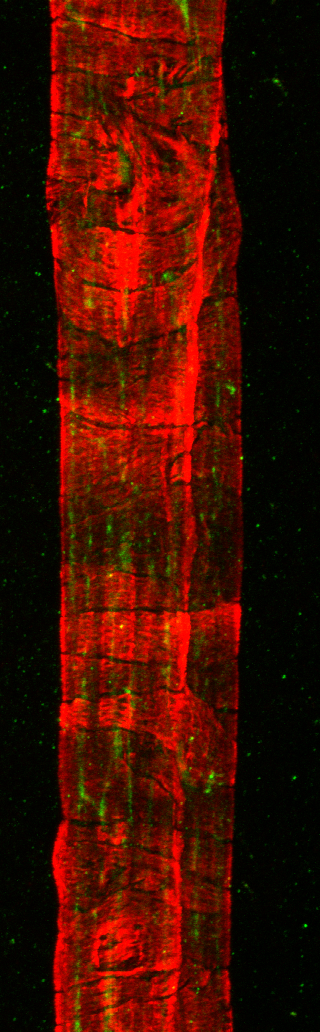

Supplement: Supplementary file 10 — Source Data for Figure 4 [file EMMM-14-e15809-s011.zip › Figure 4 Source Data/Figure 4E 1 year/Fig4E_WT_5577_aSMA-CD31_63x (RGB).tif]

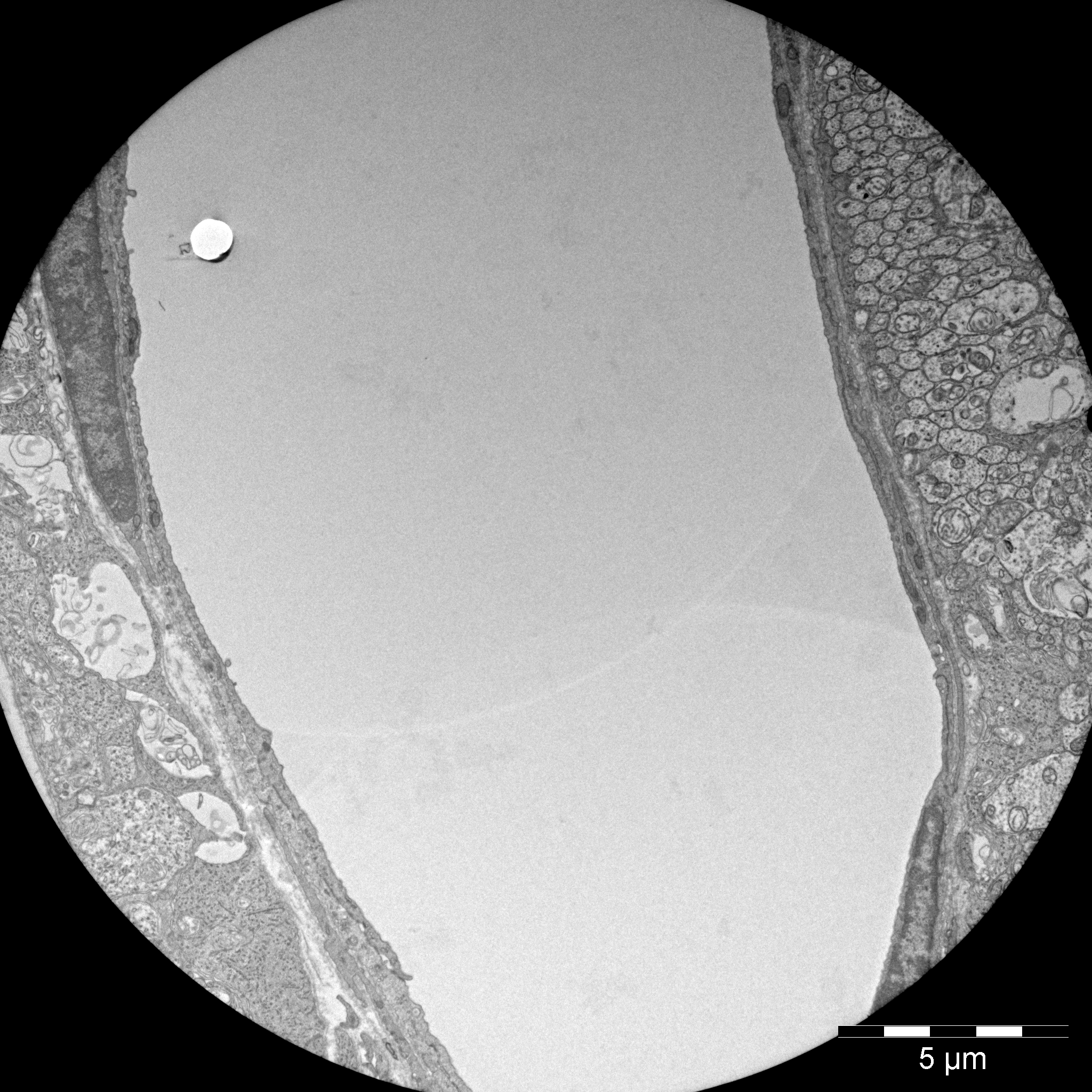

Supplement: Supplementary file 10 — Source Data for Figure 4 [file EMMM-14-e15809-s011.zip › Figure 4 Source Data/Figure 4H EM/Fig4H_NDR_1493_C258-1r SiHaE2-5 [1].tif]

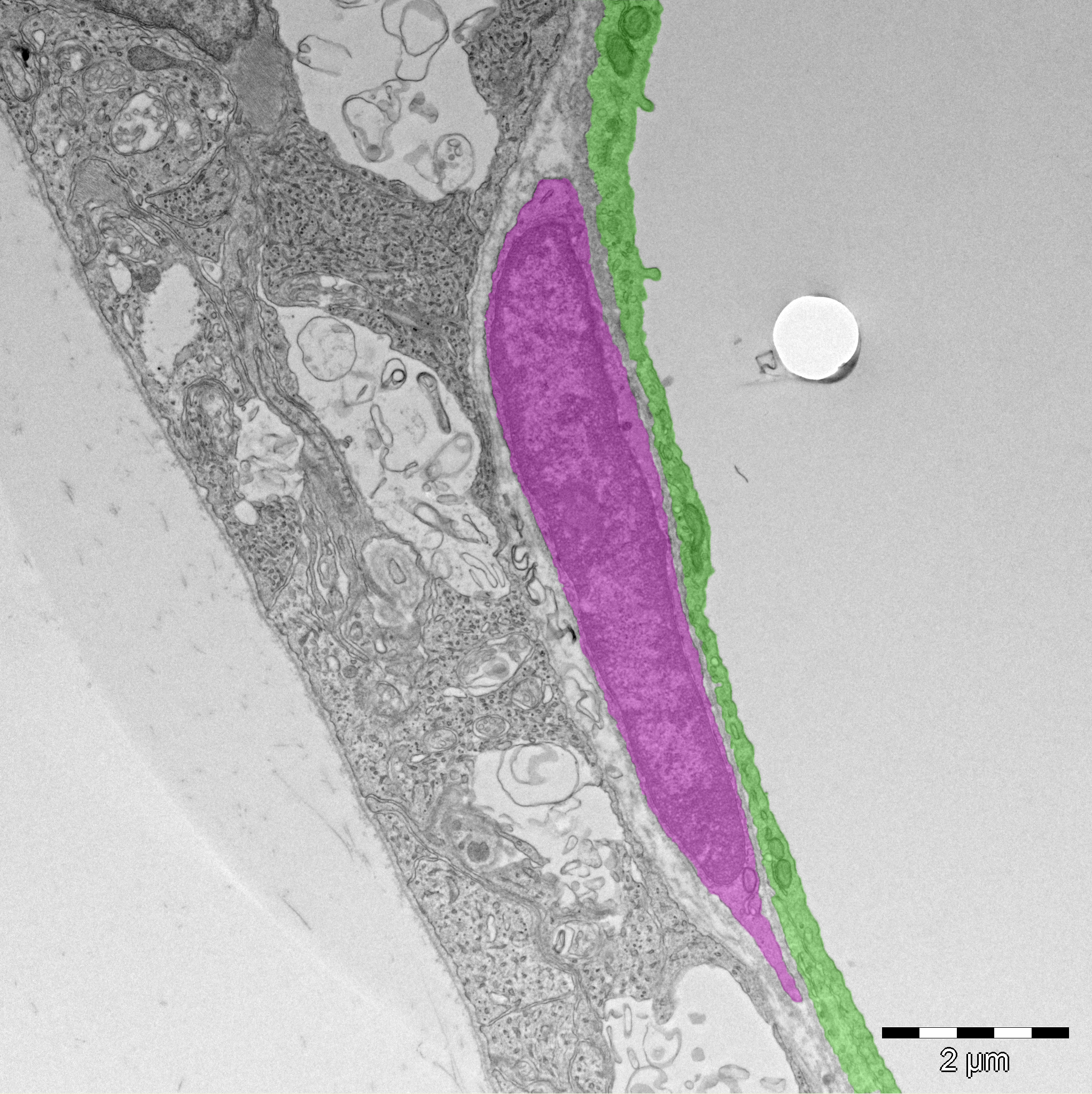

Supplement: Supplementary file 10 — Source Data for Figure 4 [file EMMM-14-e15809-s011.zip › Figure 4 Source Data/Figure 4H EM/Fig4H_NDR_1493_color copy.tif]

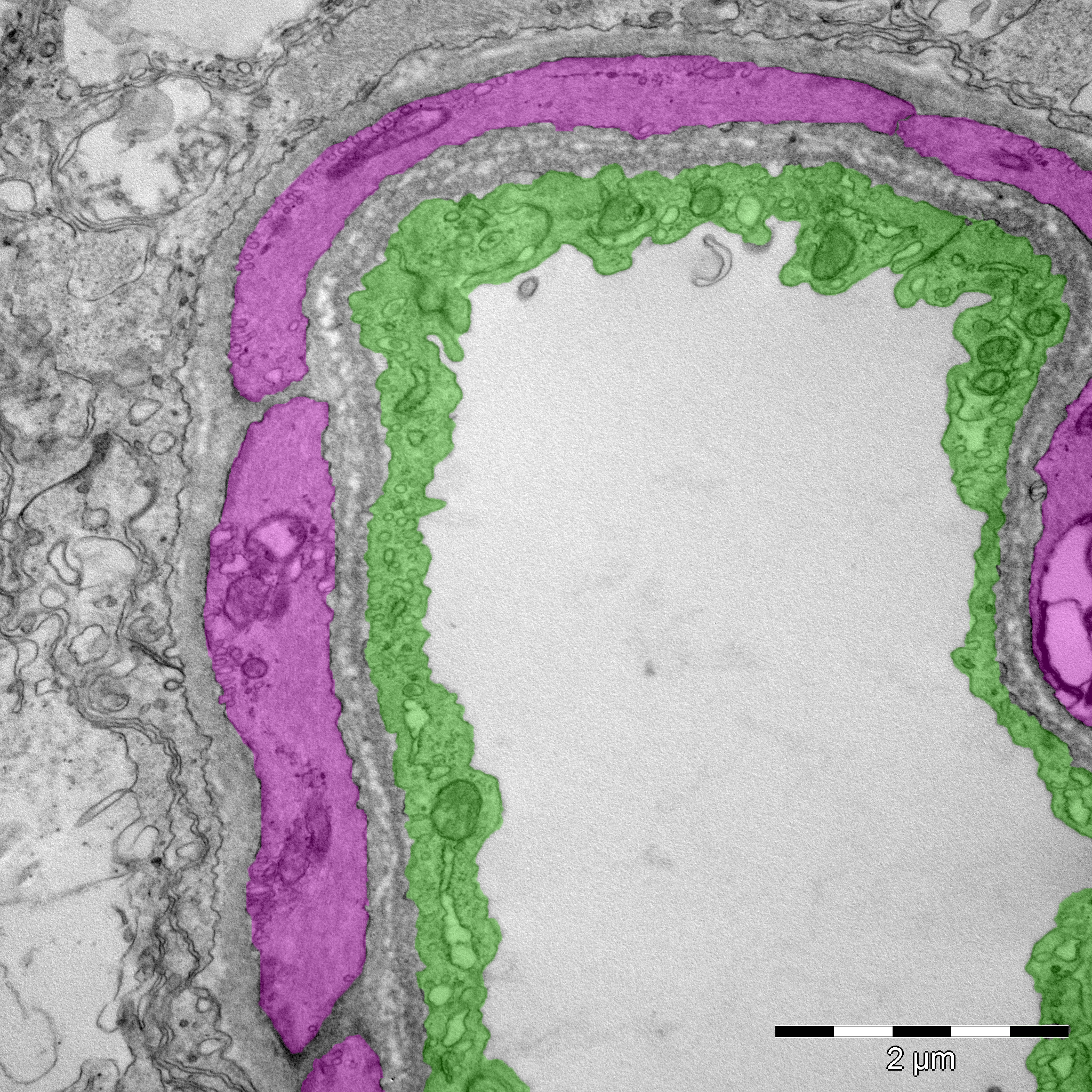

Supplement: Supplementary file 10 — Source Data for Figure 4 [file EMMM-14-e15809-s011.zip › Figure 4 Source Data/Figure 4H EM/Fig4H_WT_1492_color copy.tif]

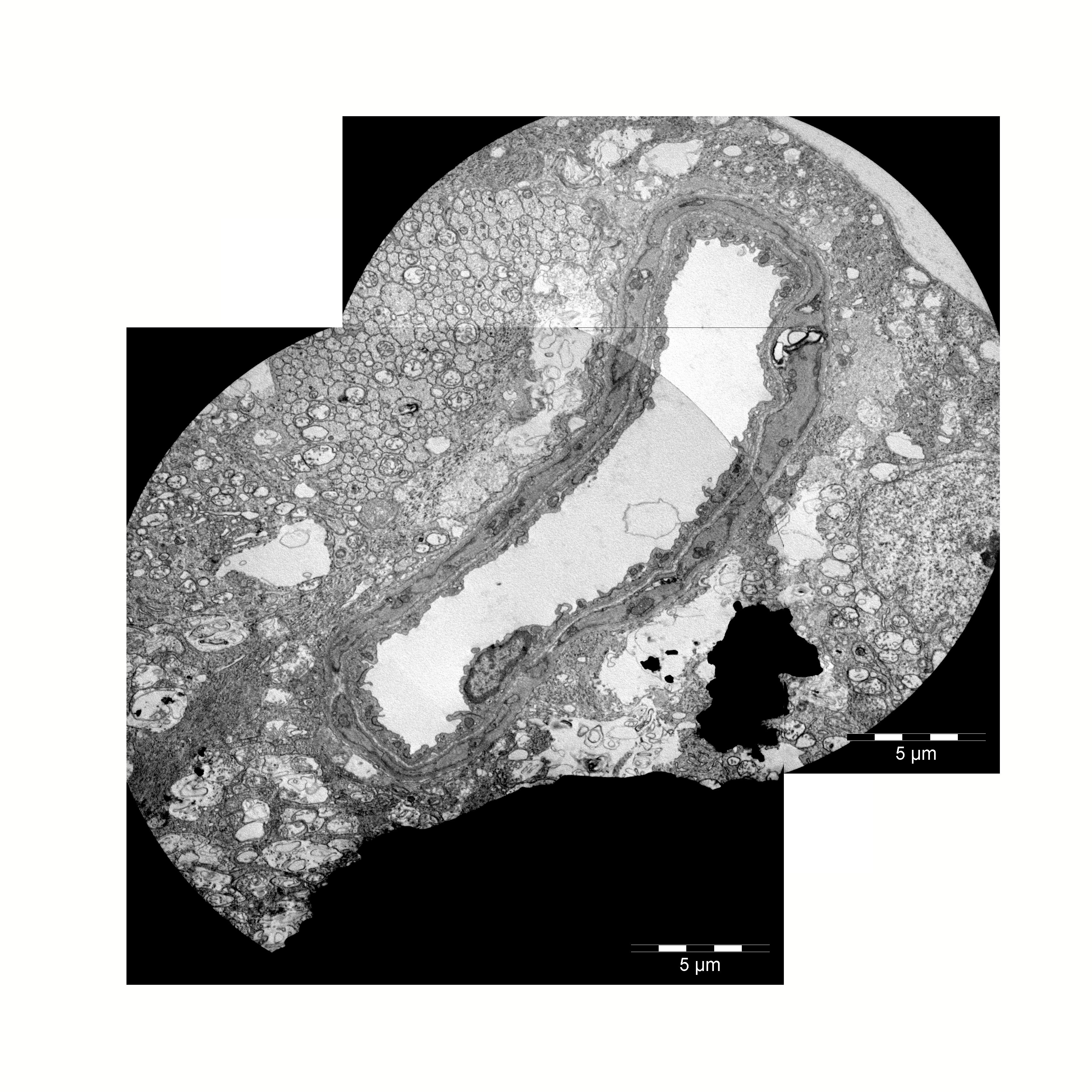

Supplement: Supplementary file 10 — Source Data for Figure 4 [file EMMM-14-e15809-s011.zip › Figure 4 Source Data/Figure 4H EM/Fig4H_WT_1492_wt_Retina_5um_2.tif]

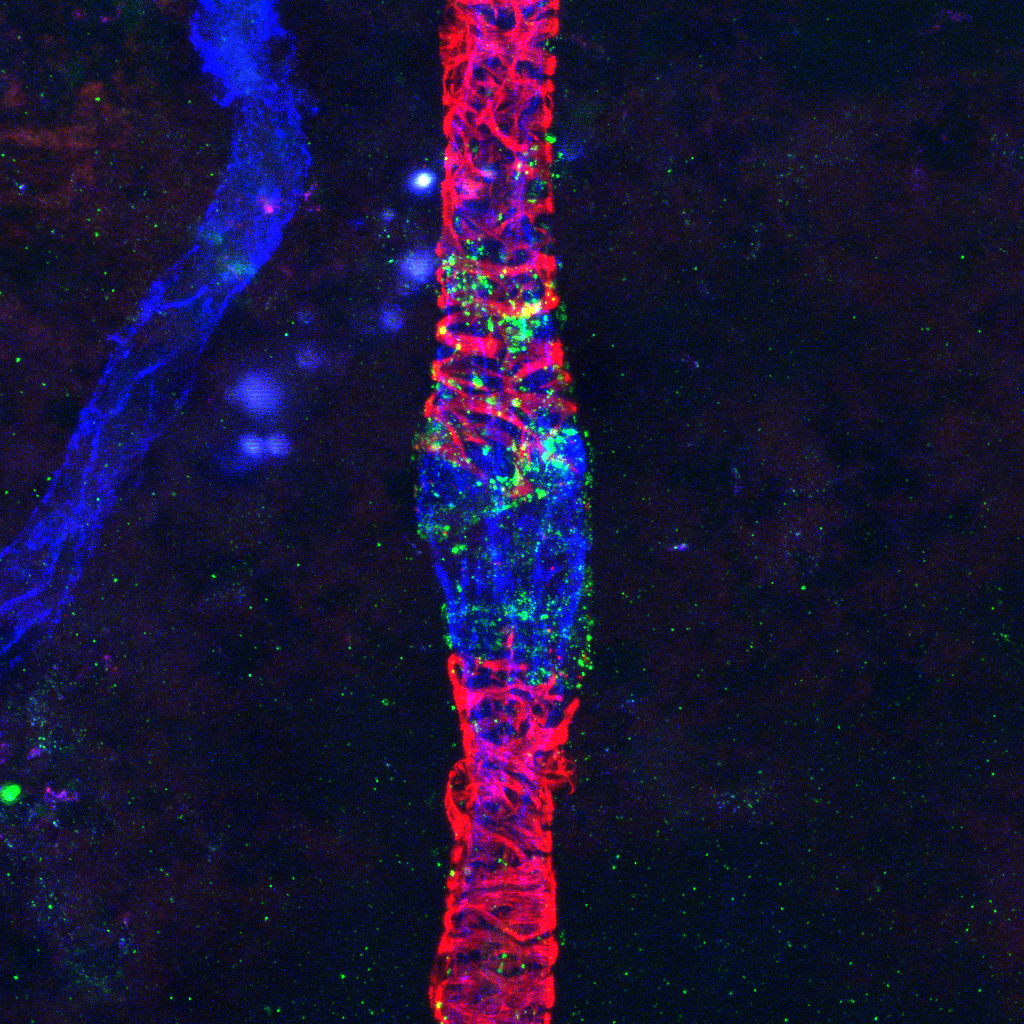

Supplement: Supplementary file 10 — Source Data for Figure 4 [file EMMM-14-e15809-s011.zip › Figure 4 Source Data/Figure 4I caspase3/Fig4I_NDR_ casp3 1 year 1532 casp3 asma cd31 63x9.tif]

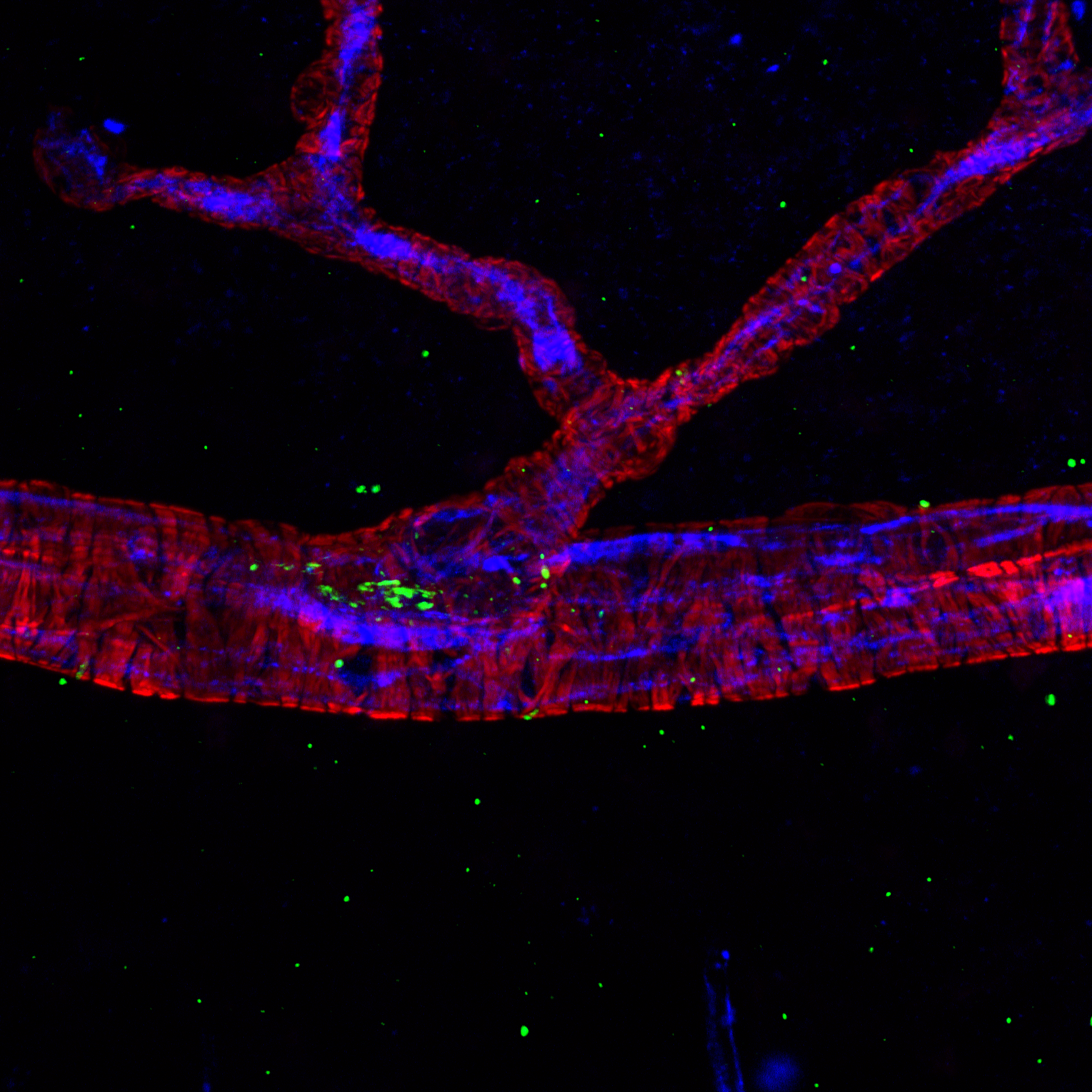

Supplement: Supplementary file 10 — Source Data for Figure 4 [file EMMM-14-e15809-s011.zip › Figure 4 Source Data/Figure 4I caspase3/Fig4I_WT_ casp3 1 year 2210 casp3 asma cd31.tif]

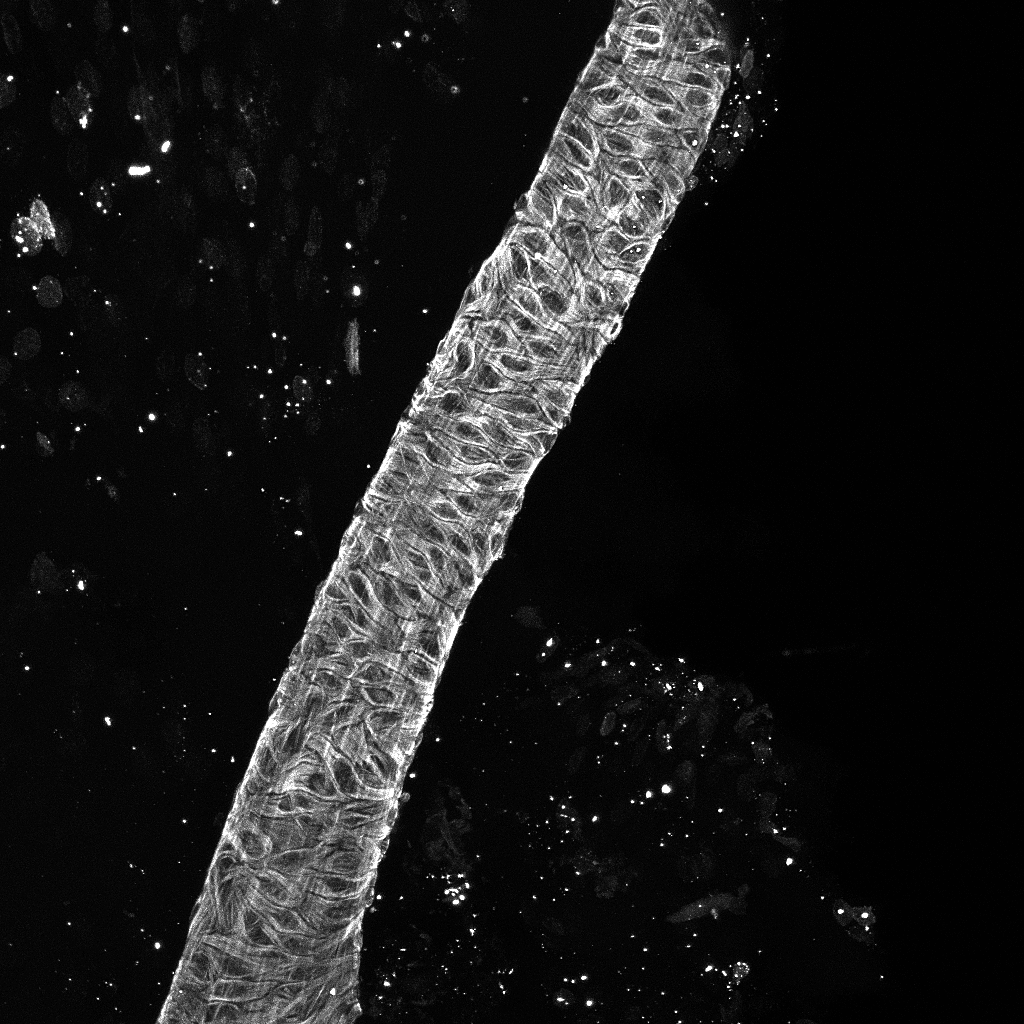

Supplement: Supplementary file 10 — Source Data for Figure 4 [file EMMM-14-e15809-s011.zip › Figure 4 Source Data/Figure 4K MCA/Fig4K_NDR_P10 brain 1mm slice asma Zstack4 20x-1.tif]

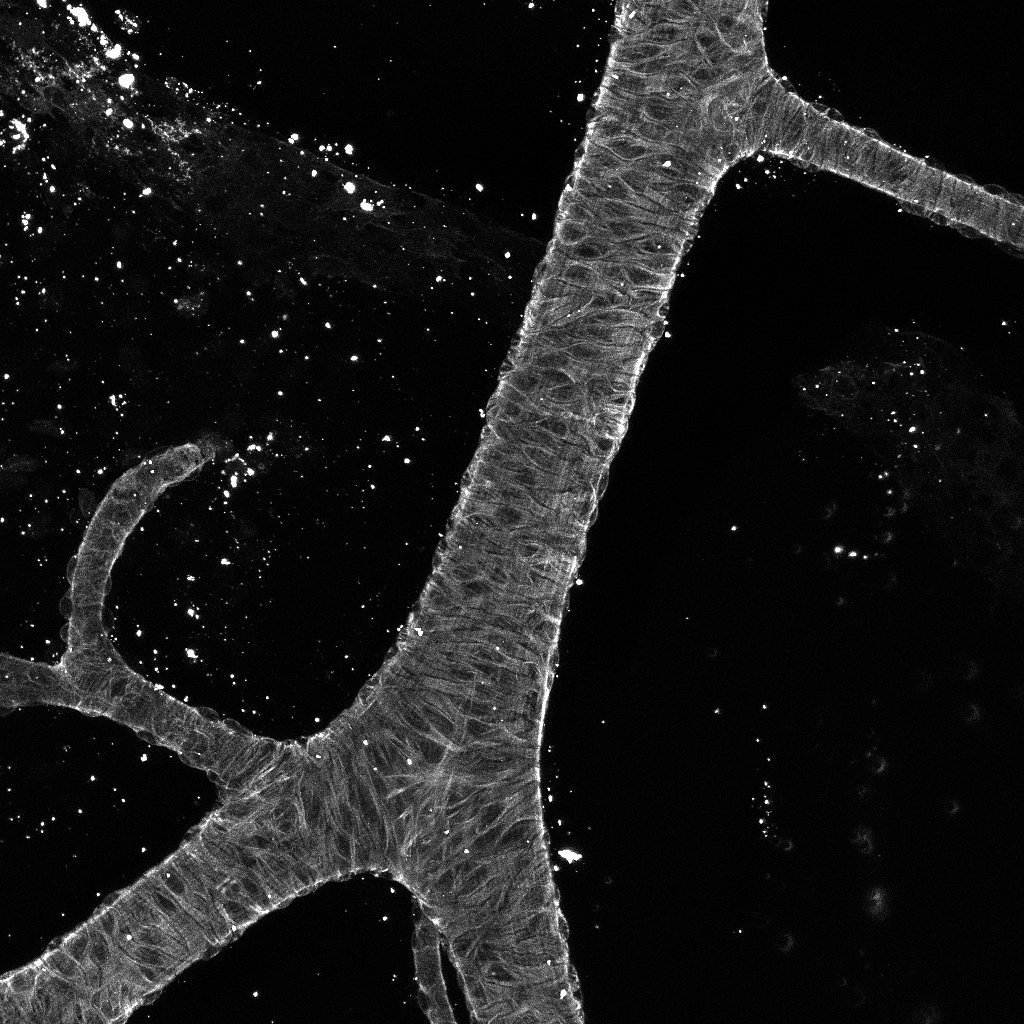

Supplement: Supplementary file 10 — Source Data for Figure 4 [file EMMM-14-e15809-s011.zip › Figure 4 Source Data/Figure 4K MCA/Fig4K_WT_ P10 brain 1mm slice asma Zstack5 20x.tif]

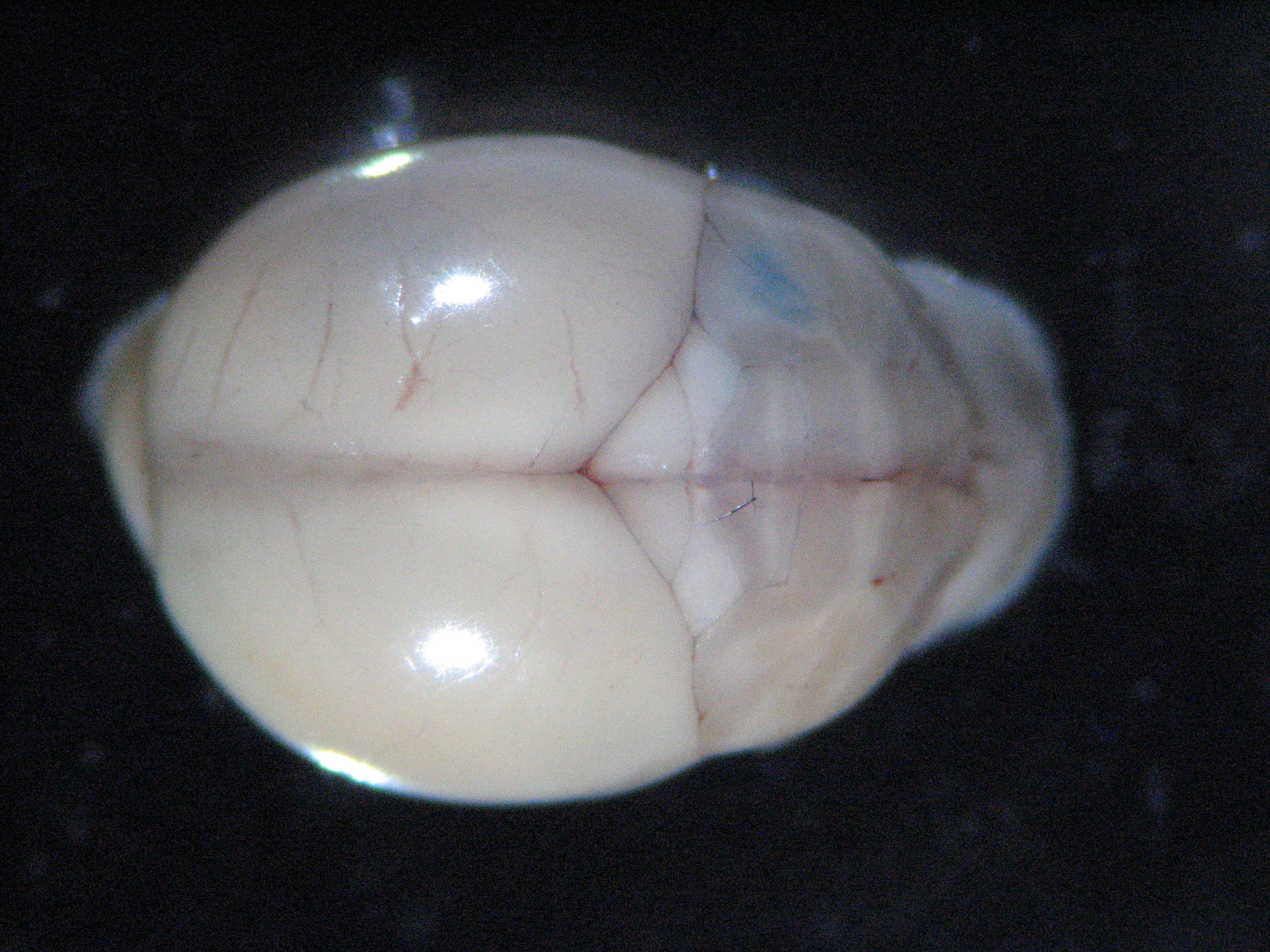

Supplement: Supplementary file 10 — Source Data for Figure 4 [file EMMM-14-e15809-s011.zip › Figure 4 Source Data/Figure 4Q Evans Blue/Fig4Q NDR brain_3004_2.JPG]

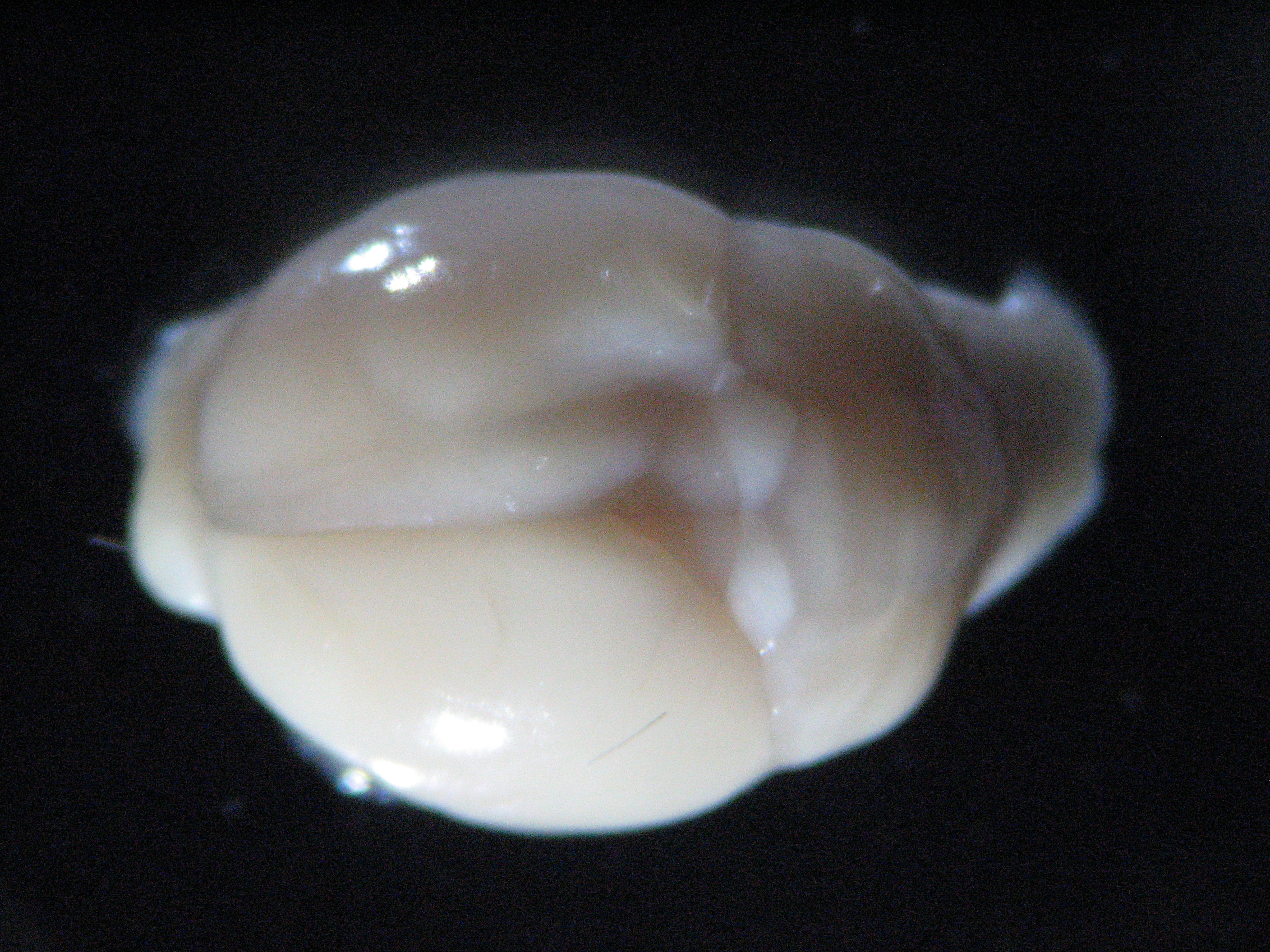

Supplement: Supplementary file 10 — Source Data for Figure 4 [file EMMM-14-e15809-s011.zip › Figure 4 Source Data/Figure 4Q Evans Blue/Fig4Q WT brain_3000-1.JPG]

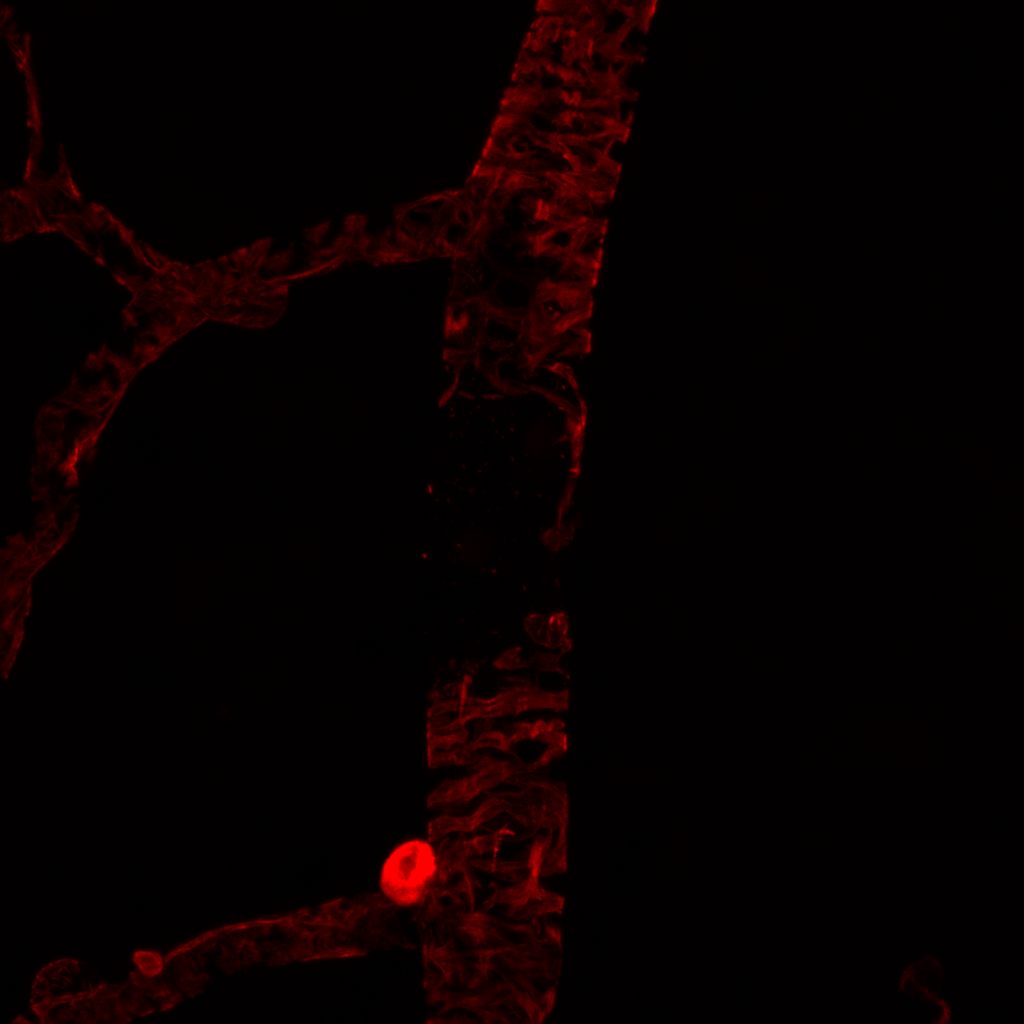

Supplement: Supplementary file 10 — Source Data for Figure 4 [file EMMM-14-e15809-s011.zip › Figure 4 Source Data/Figure 4R AngII asma/Fig4R_ndr_angII_3004-asma_cd31_stack_63x9.tif]

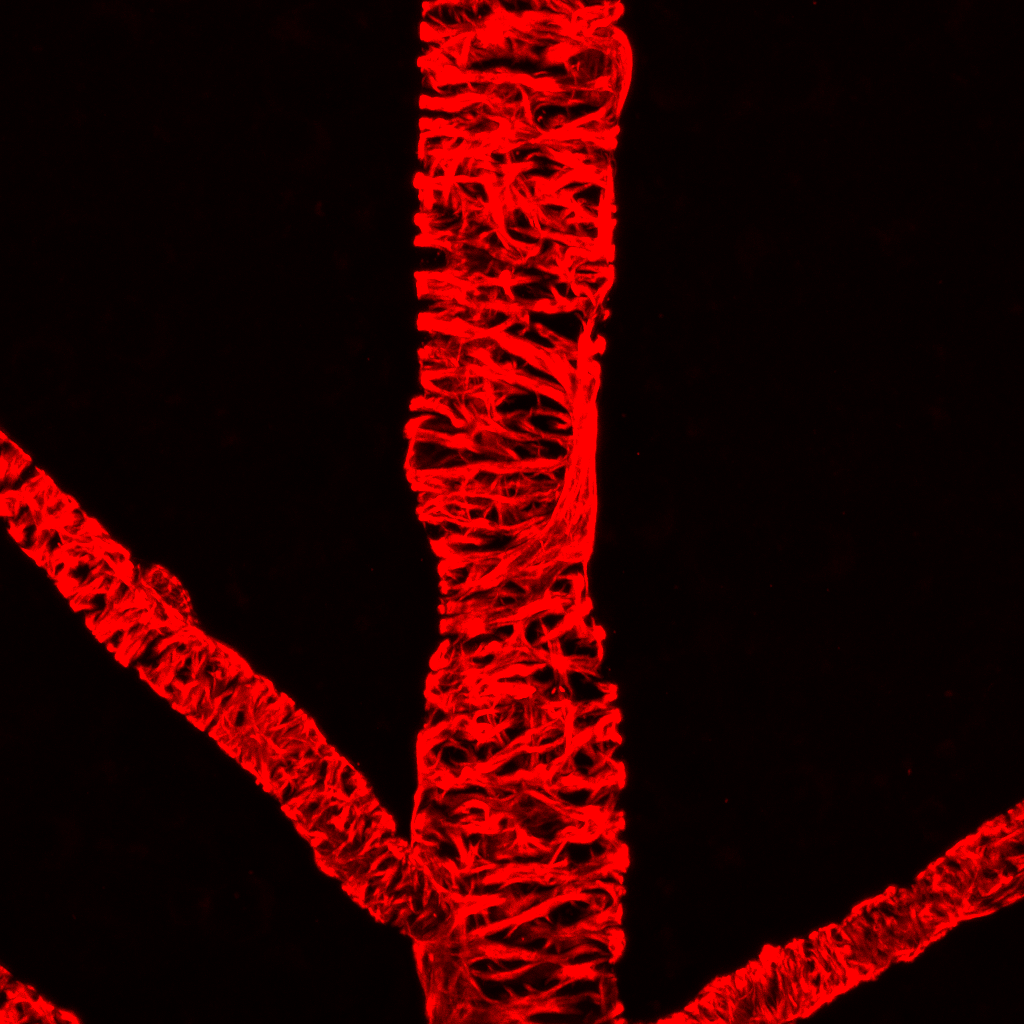

Supplement: Supplementary file 10 — Source Data for Figure 4 [file EMMM-14-e15809-s011.zip › Figure 4 Source Data/Figure 4R AngII asma/Fig4R_ndr_ctrl_2948-asma_cd31_stack_63x1.tif]

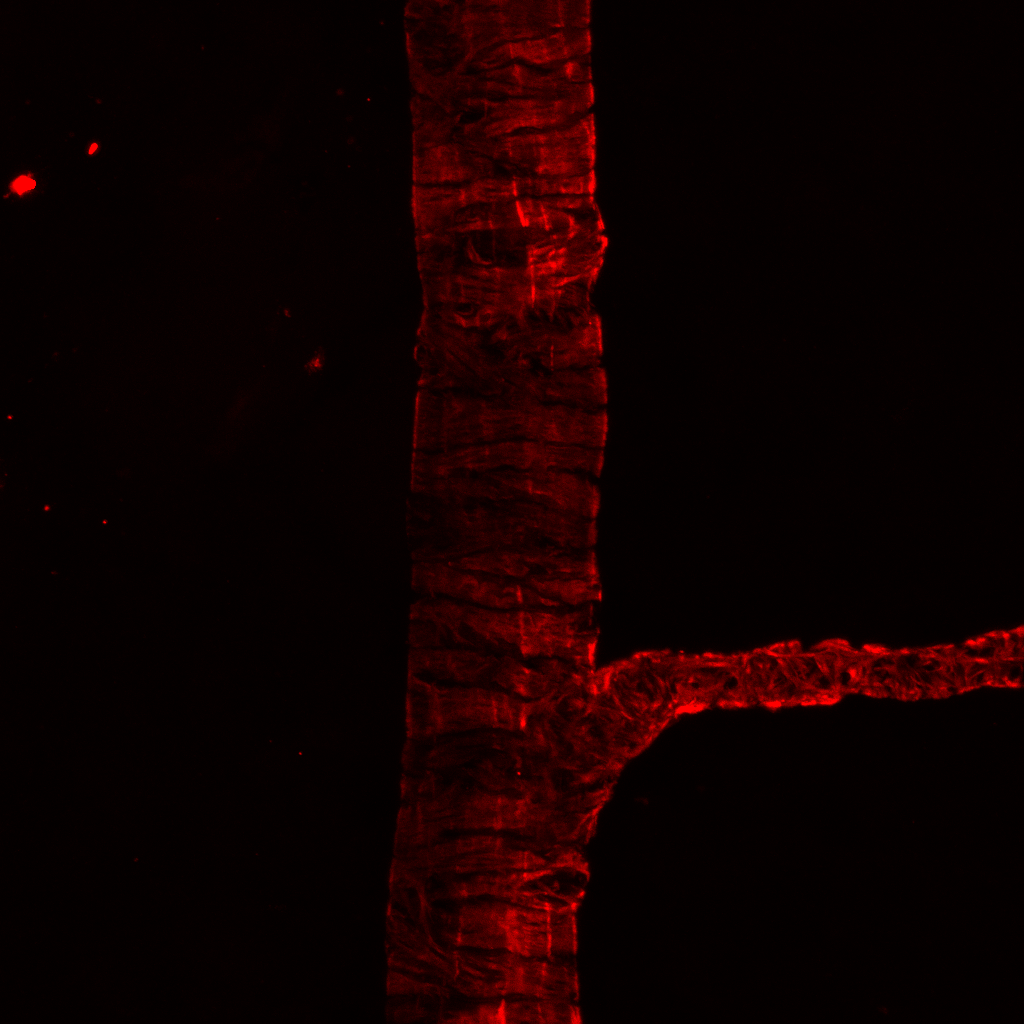

Supplement: Supplementary file 10 — Source Data for Figure 4 [file EMMM-14-e15809-s011.zip › Figure 4 Source Data/Figure 4R AngII asma/Fig4R_wt_angII_3000-asma_cd31_stack_63x3.tif]

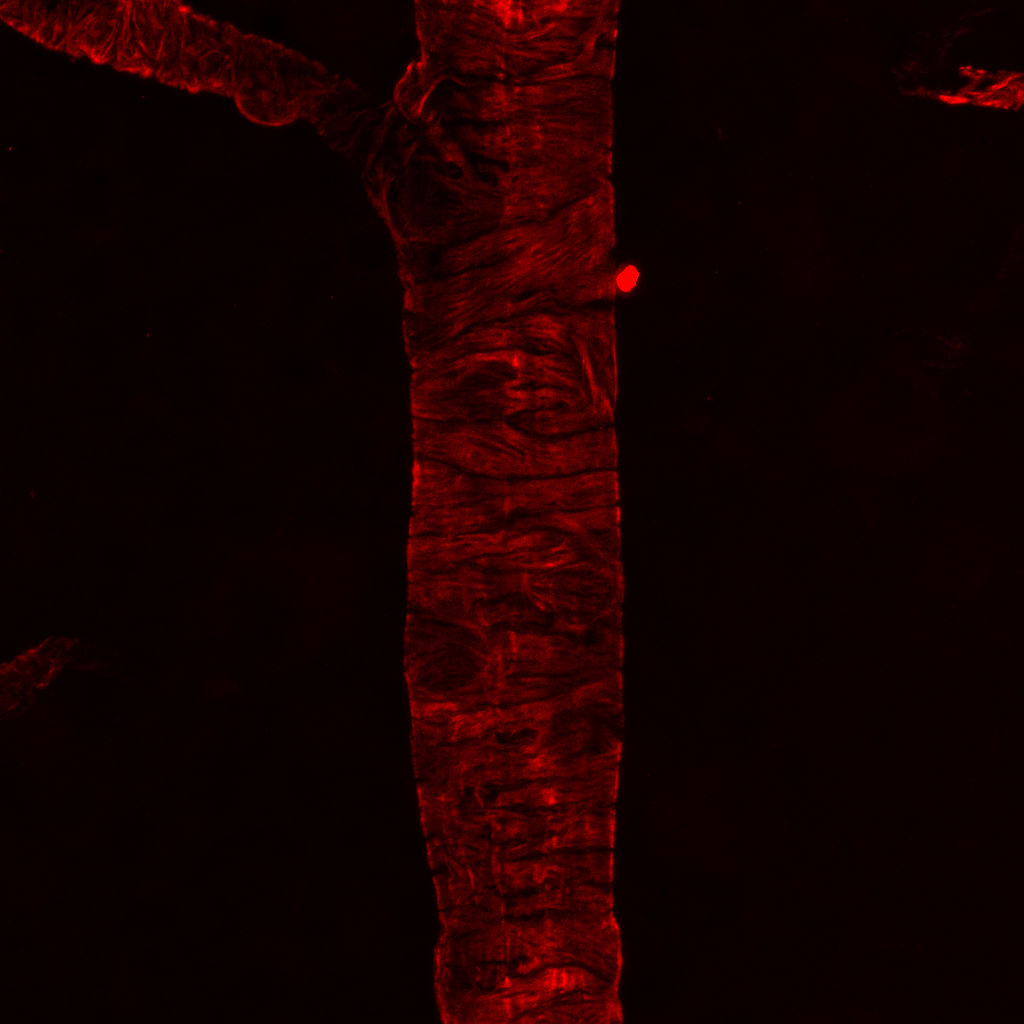

Supplement: Supplementary file 10 — Source Data for Figure 4 [file EMMM-14-e15809-s011.zip › Figure 4 Source Data/Figure 4R AngII asma/Fig4R_wt_ctrl_2961-asma_cd31_stack_63x2.tif]

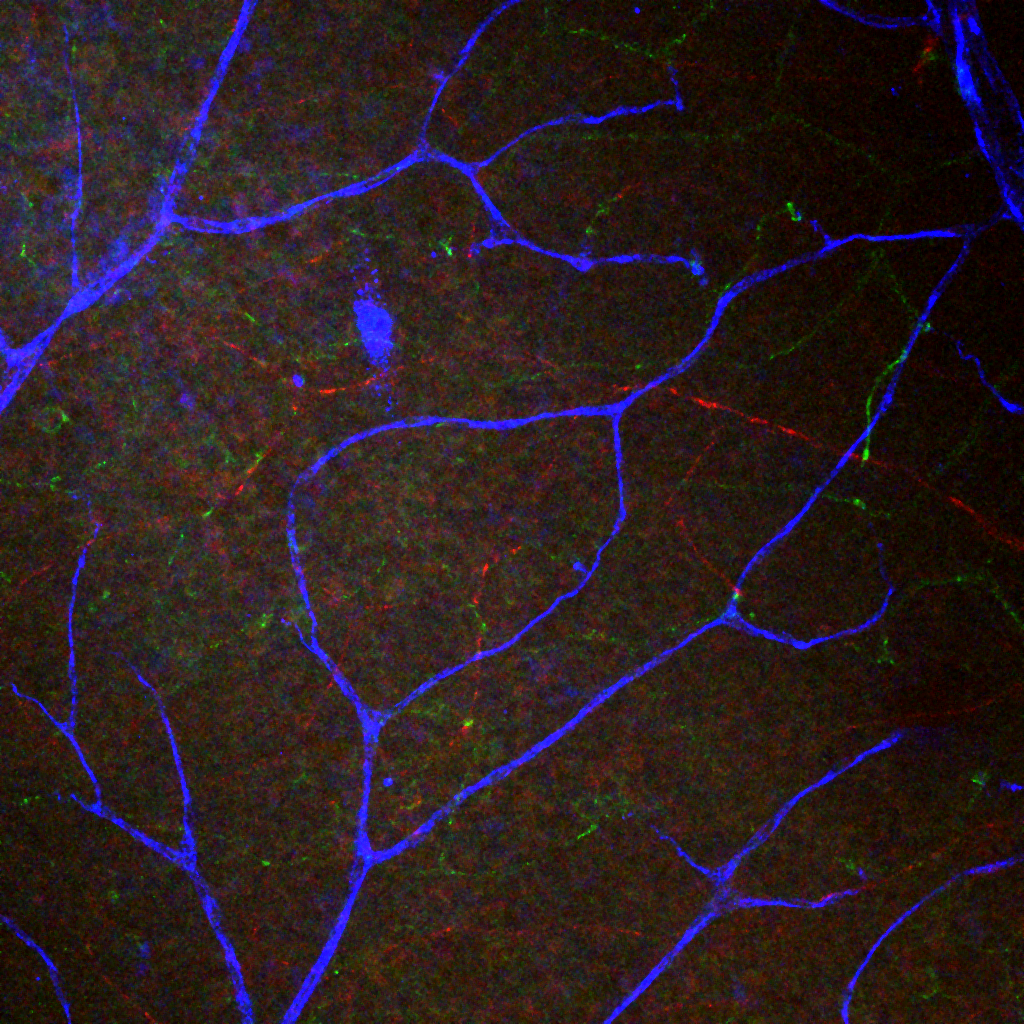

Supplement: Supplementary file 11 — Source Data for Figure 5 [file EMMM-14-e15809-s006.zip › Figure 5 Source Data/Figure 5A P30 3 layers/Fig5A_NDR_2014_P30_cd31_40x3_Levels.tif]

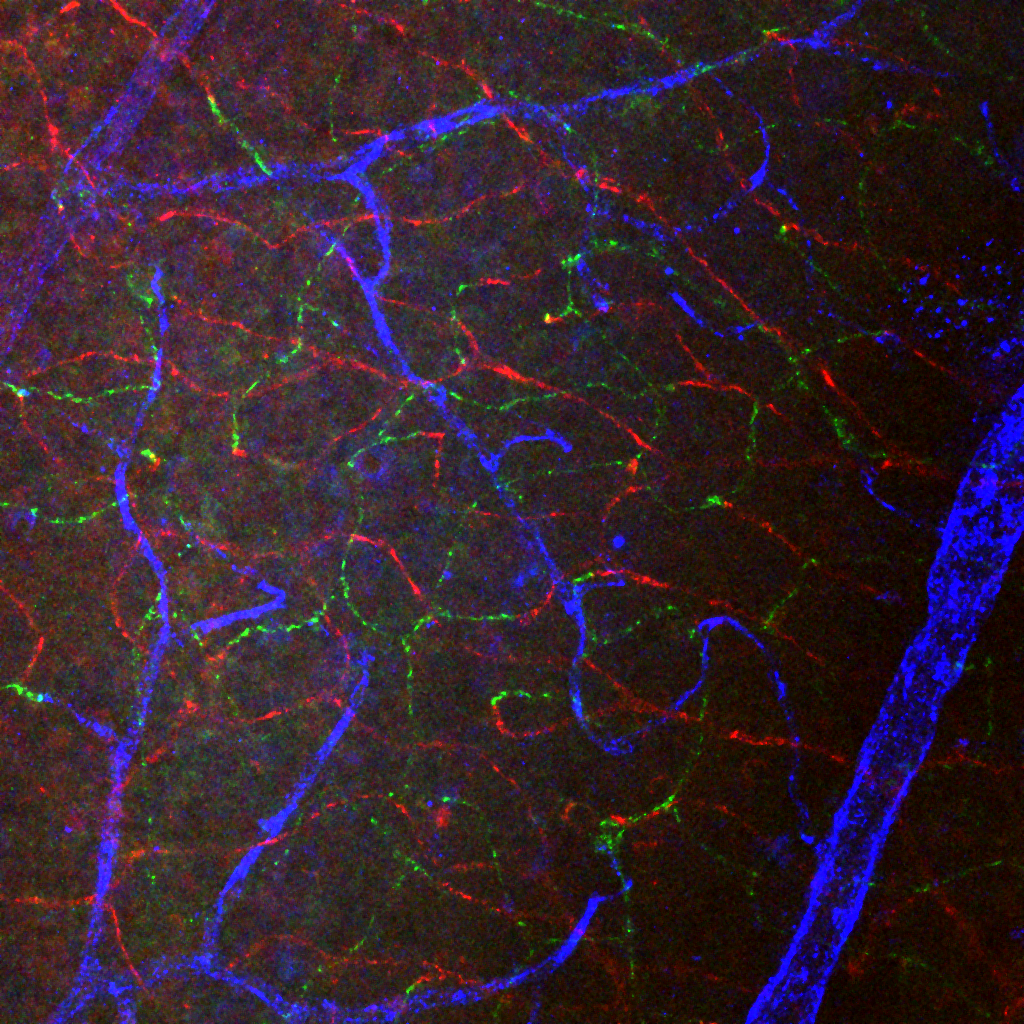

Supplement: Supplementary file 11 — Source Data for Figure 5 [file EMMM-14-e15809-s006.zip › Figure 5 Source Data/Figure 5A P30 3 layers/Fig5A_WT_1907_P30_cd31_40x1_Levels.tif]

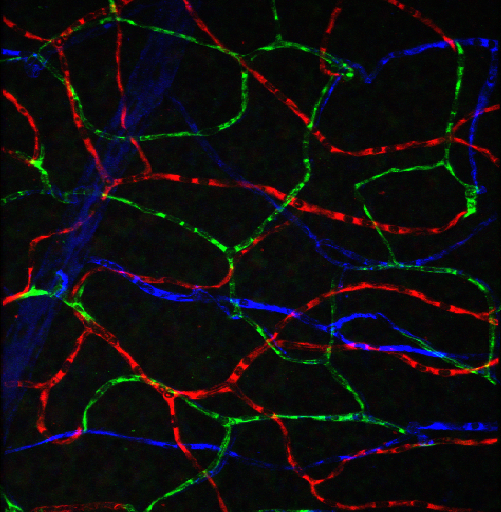

Supplement: Supplementary file 11 — Source Data for Figure 5 [file EMMM-14-e15809-s006.zip › Figure 5 Source Data/Figure 5E adult 3 layers/Fig5E_NDR_1366 all layers.tif]

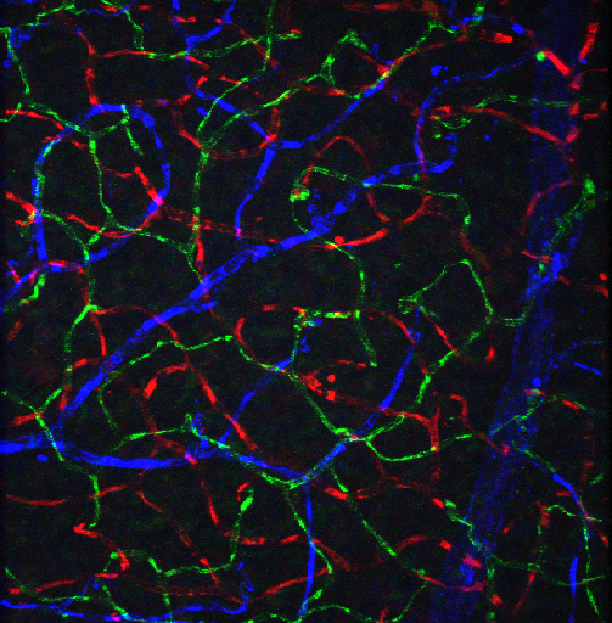

Supplement: Supplementary file 11 — Source Data for Figure 5 [file EMMM-14-e15809-s006.zip › Figure 5 Source Data/Figure 5E adult 3 layers/Fig5E_WT_1445 all layers.tif]

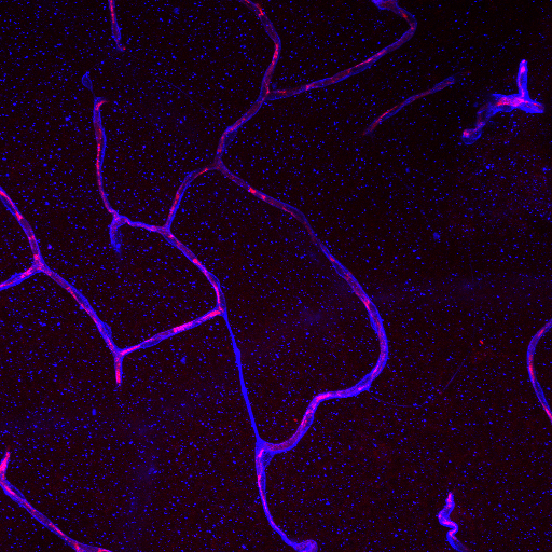

Supplement: Supplementary file 11 — Source Data for Figure 5 [file EMMM-14-e15809-s006.zip › Figure 5 Source Data/Figure 5I coliv sleeve/Fig5I_NDR_1383 40x ICP 3.tif]

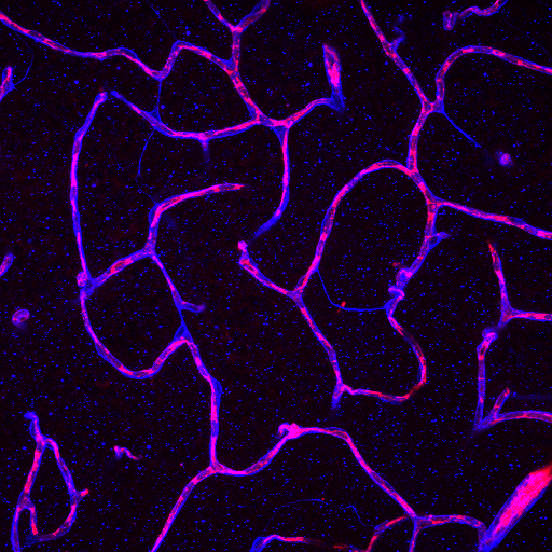

Supplement: Supplementary file 11 — Source Data for Figure 5 [file EMMM-14-e15809-s006.zip › Figure 5 Source Data/Figure 5I coliv sleeve/Fig5I_WT_1382 40x ICP 1.tif]

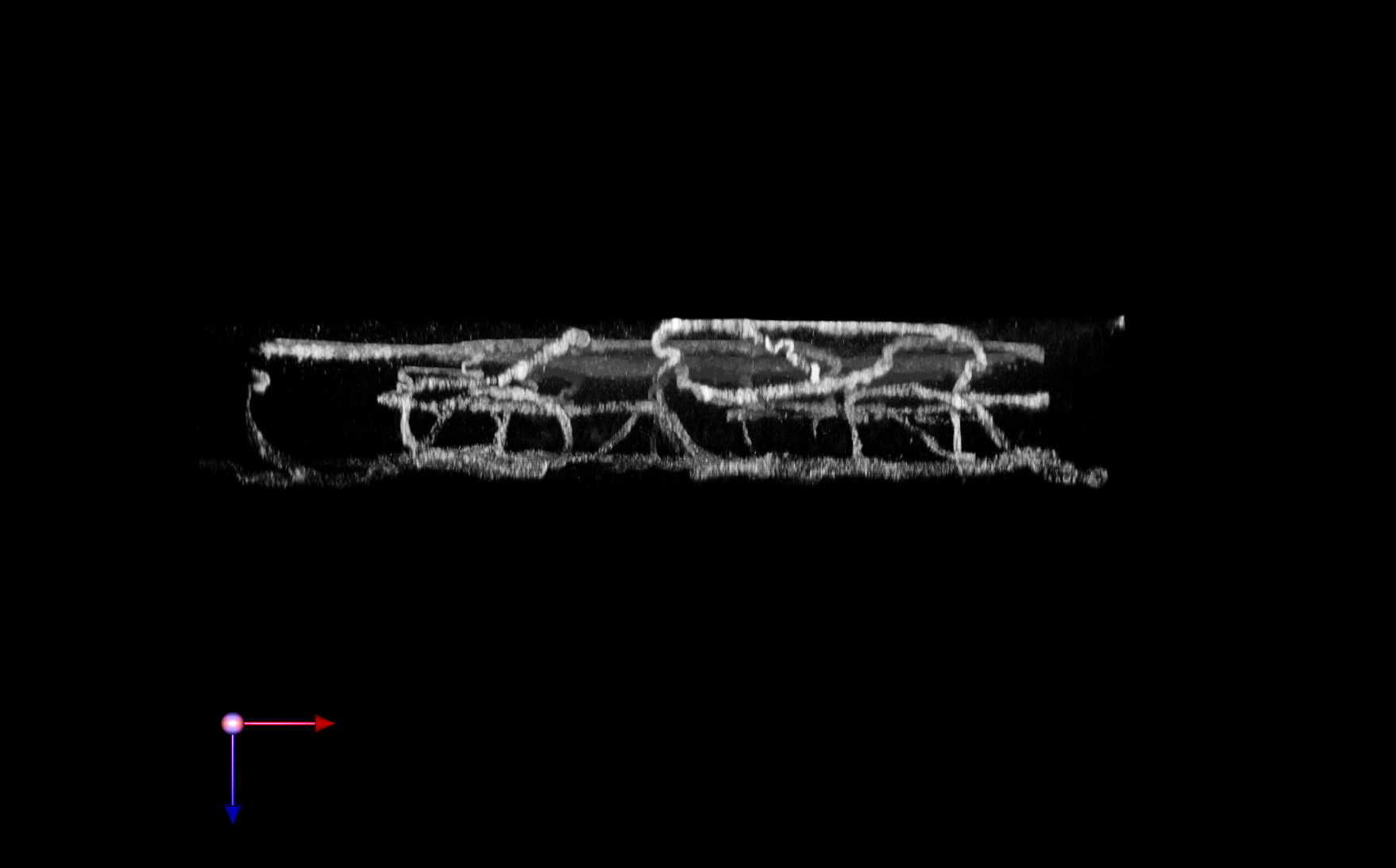

Supplement: Supplementary file 11 — Source Data for Figure 5 [file EMMM-14-e15809-s006.zip › Figure 5 Source Data/Figure 5K vertical retina/Fig5K_NDR_1year_811 cd31 40x1 G.tif]

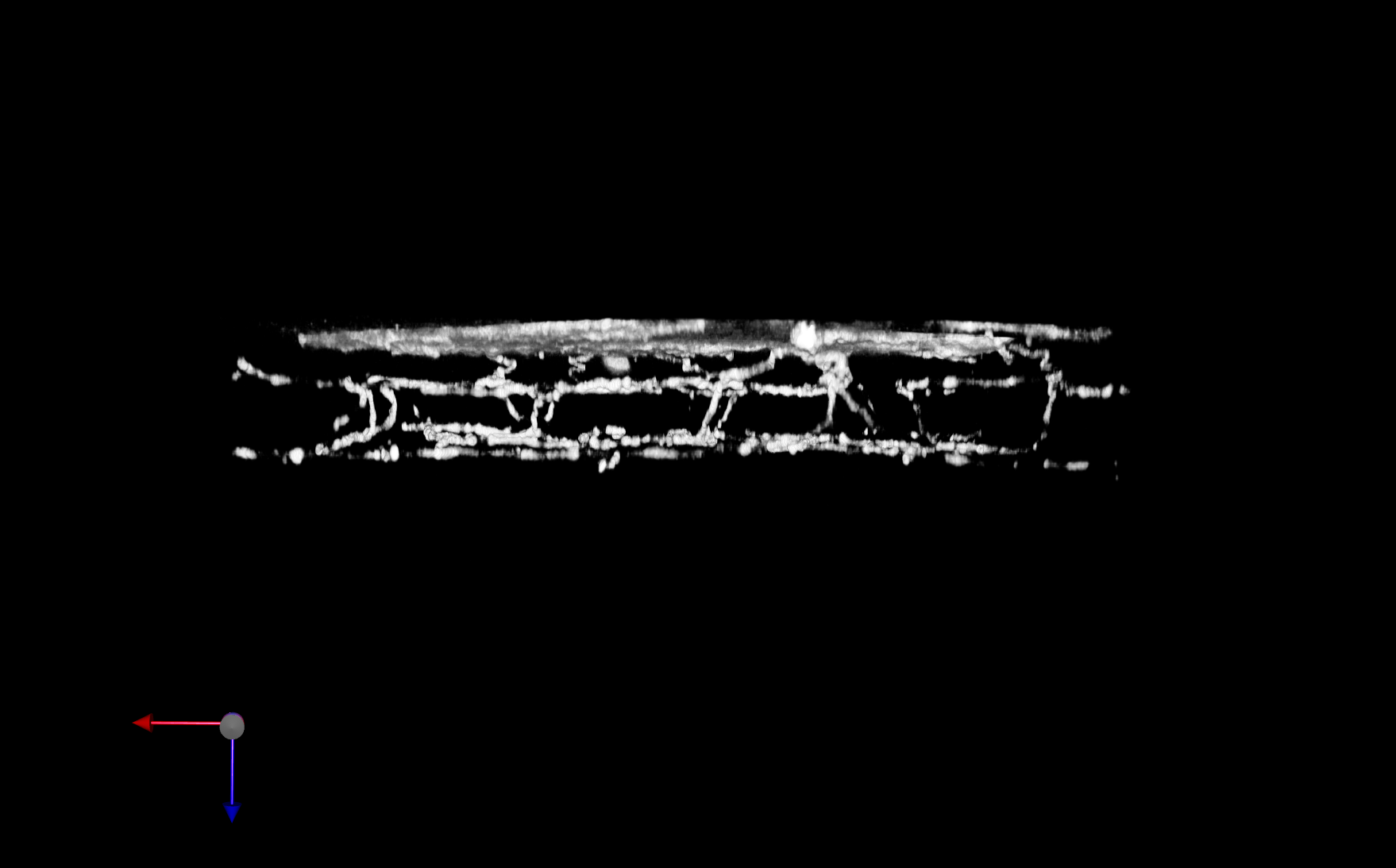

Supplement: Supplementary file 11 — Source Data for Figure 5 [file EMMM-14-e15809-s006.zip › Figure 5 Source Data/Figure 5K vertical retina/Fig5K_NDR_adult_1383 cd31 40x.tif]

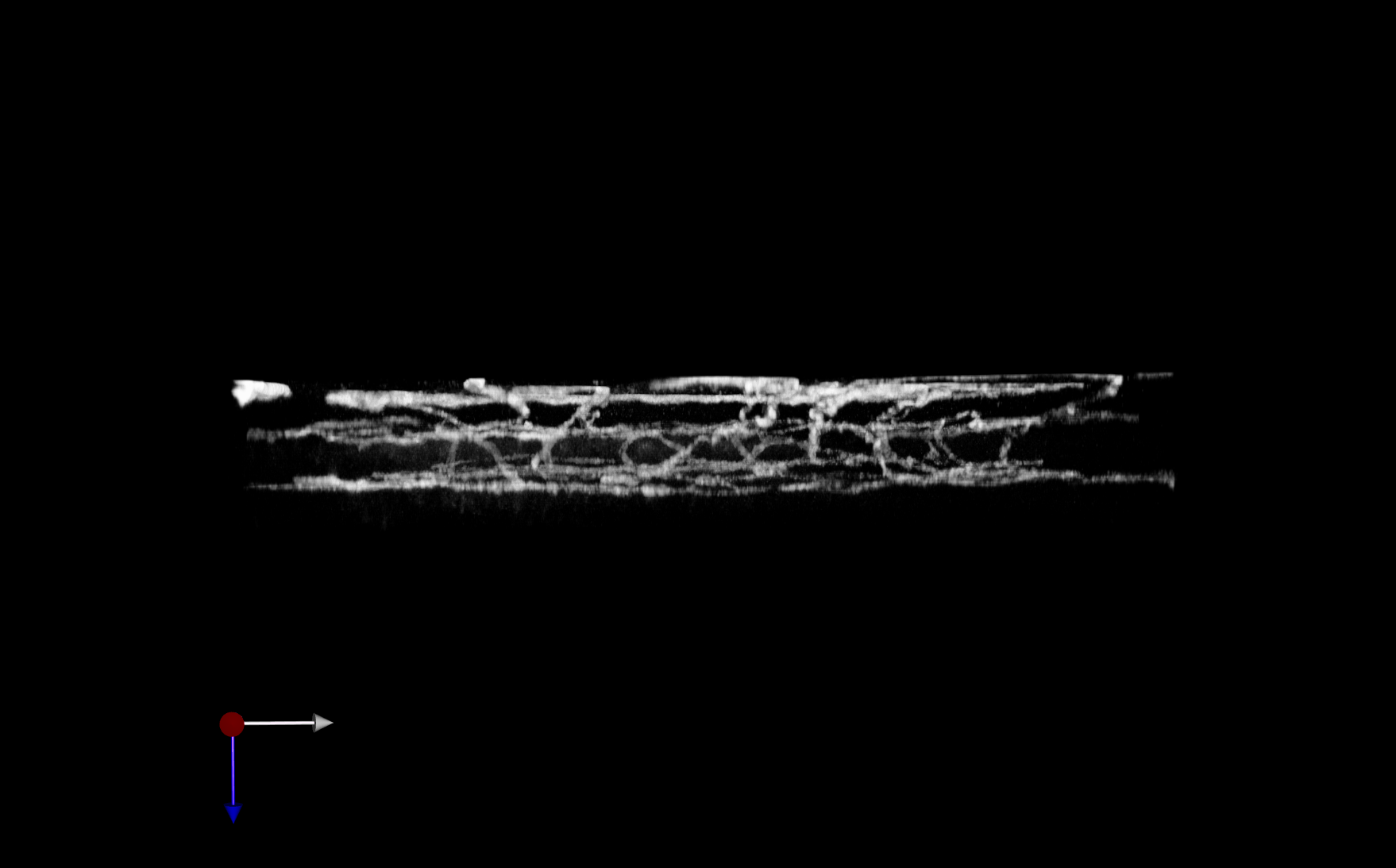

Supplement: Supplementary file 11 — Source Data for Figure 5 [file EMMM-14-e15809-s006.zip › Figure 5 Source Data/Figure 5K vertical retina/Fig5K_WT_1year_733 cd31 40x1G.tif]

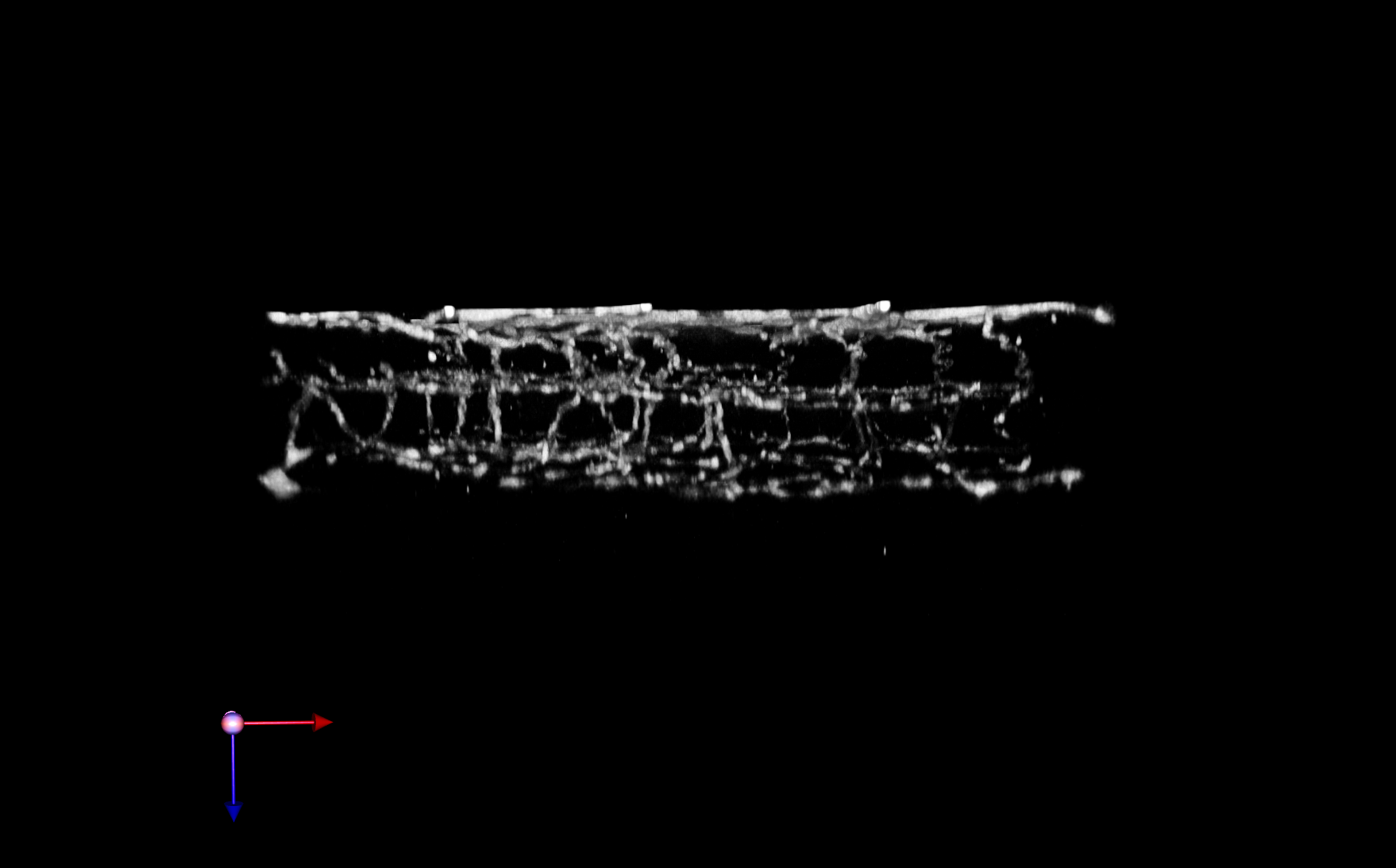

Supplement: Supplementary file 11 — Source Data for Figure 5 [file EMMM-14-e15809-s006.zip › Figure 5 Source Data/Figure 5K vertical retina/Fig5K_WT_adult_1435 cd31 40x.tif]

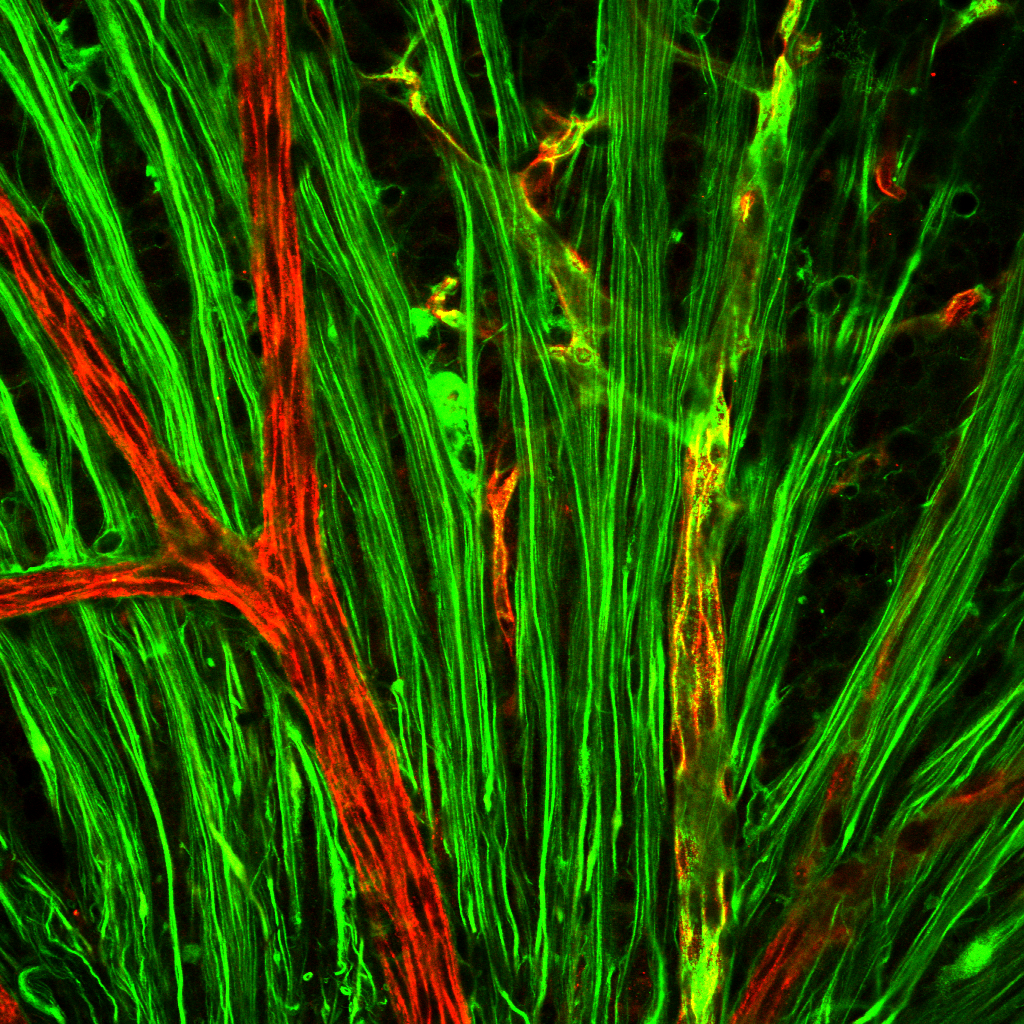

Supplement: Supplementary file 11 — Source Data for Figure 5 [file EMMM-14-e15809-s006.zip › Figure 5 Source Data/Figure 5N P10 NF/Fig5N_NDR_181-1_nf_40x1-1.lsm (RGB).tif]

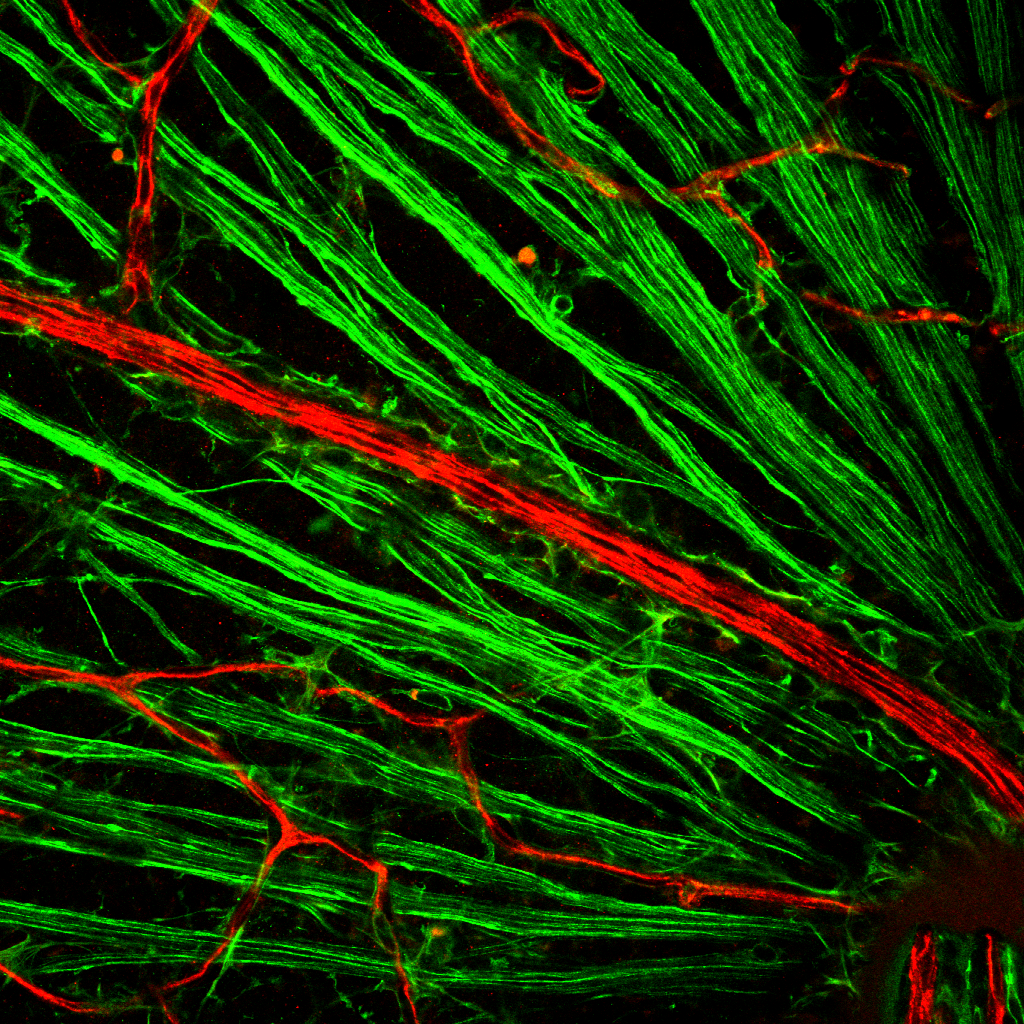

Supplement: Supplementary file 11 — Source Data for Figure 5 [file EMMM-14-e15809-s006.zip › Figure 5 Source Data/Figure 5N P10 NF/Fig5N_WT_181_nf_40x0.lsm (RGB).tif]

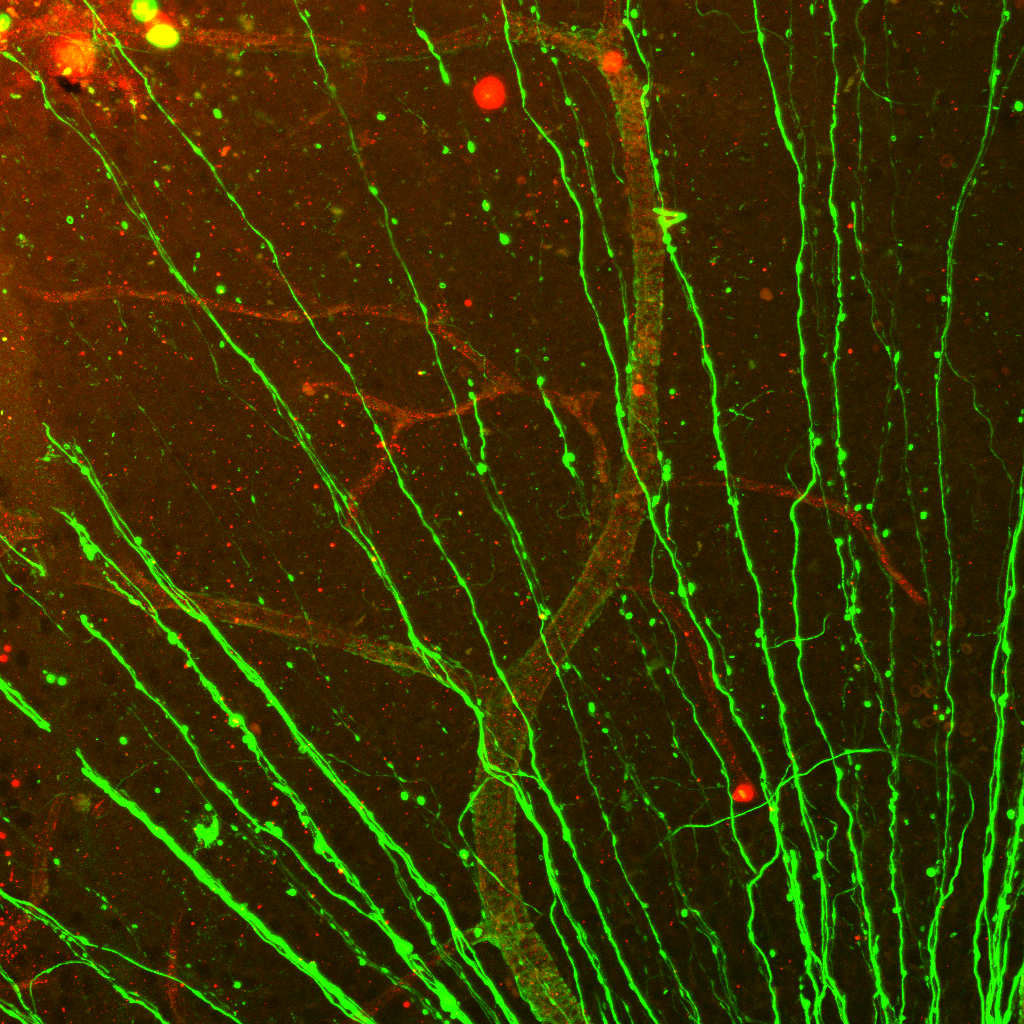

Supplement: Supplementary file 11 — Source Data for Figure 5 [file EMMM-14-e15809-s006.zip › Figure 5 Source Data/Figure 5O P40 NF/Fig5O_NDR_N805 ndr NF cd31 20x2.tif]

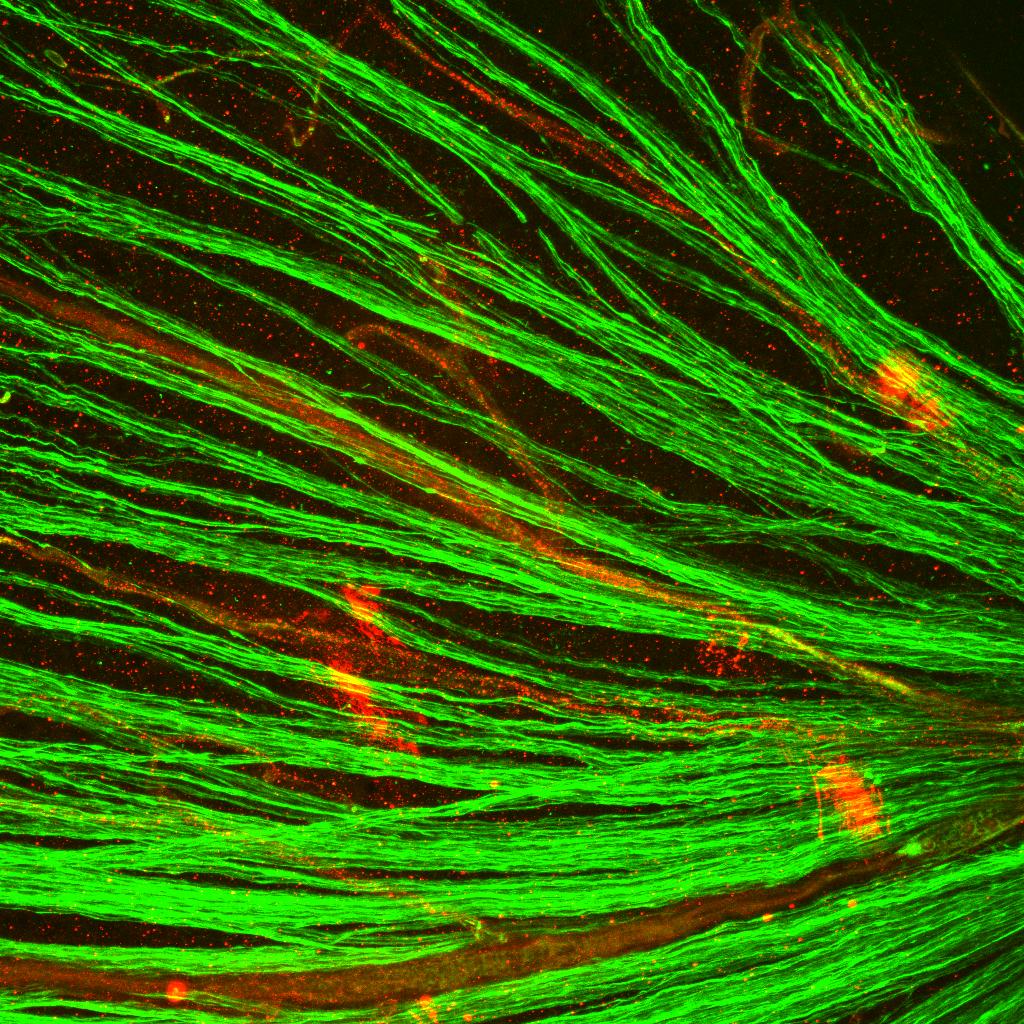

Supplement: Supplementary file 11 — Source Data for Figure 5 [file EMMM-14-e15809-s006.zip › Figure 5 Source Data/Figure 5O P40 NF/Fig5O_WT_N785 NF cd31 20x4.tif]

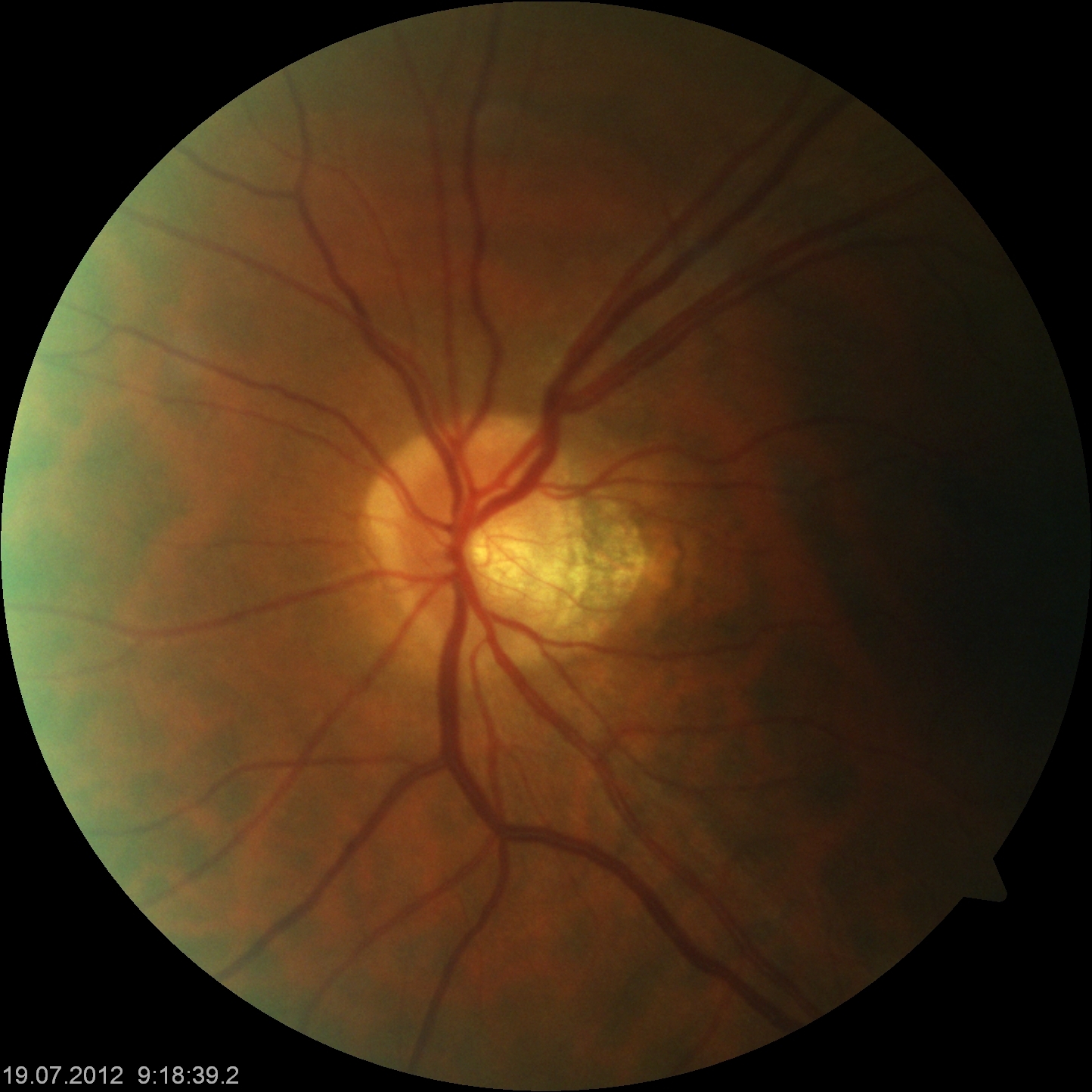

Supplement: Supplementary file 12 — Source Data for Figure 6 [file EMMM-14-e15809-s004.zip › Figure 6 Source Data/Figure 6 source data images/Fig6A adult CTRL 20 OS.jpg]

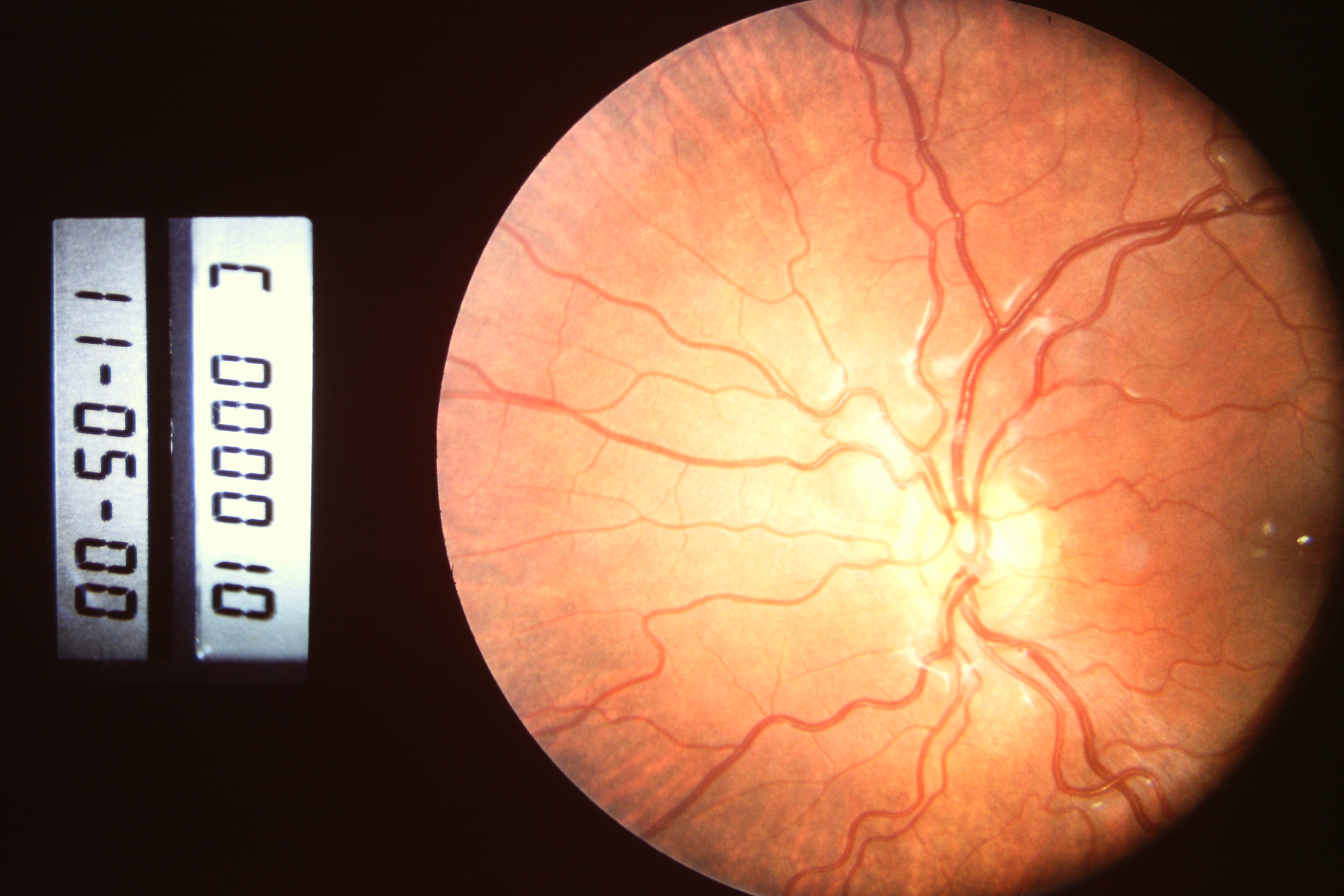

Supplement: Supplementary file 12 — Source Data for Figure 6 [file EMMM-14-e15809-s004.zip › Figure 6 Source Data/Figure 6 source data images/Fig6A ALGS PICT0023.JPG]

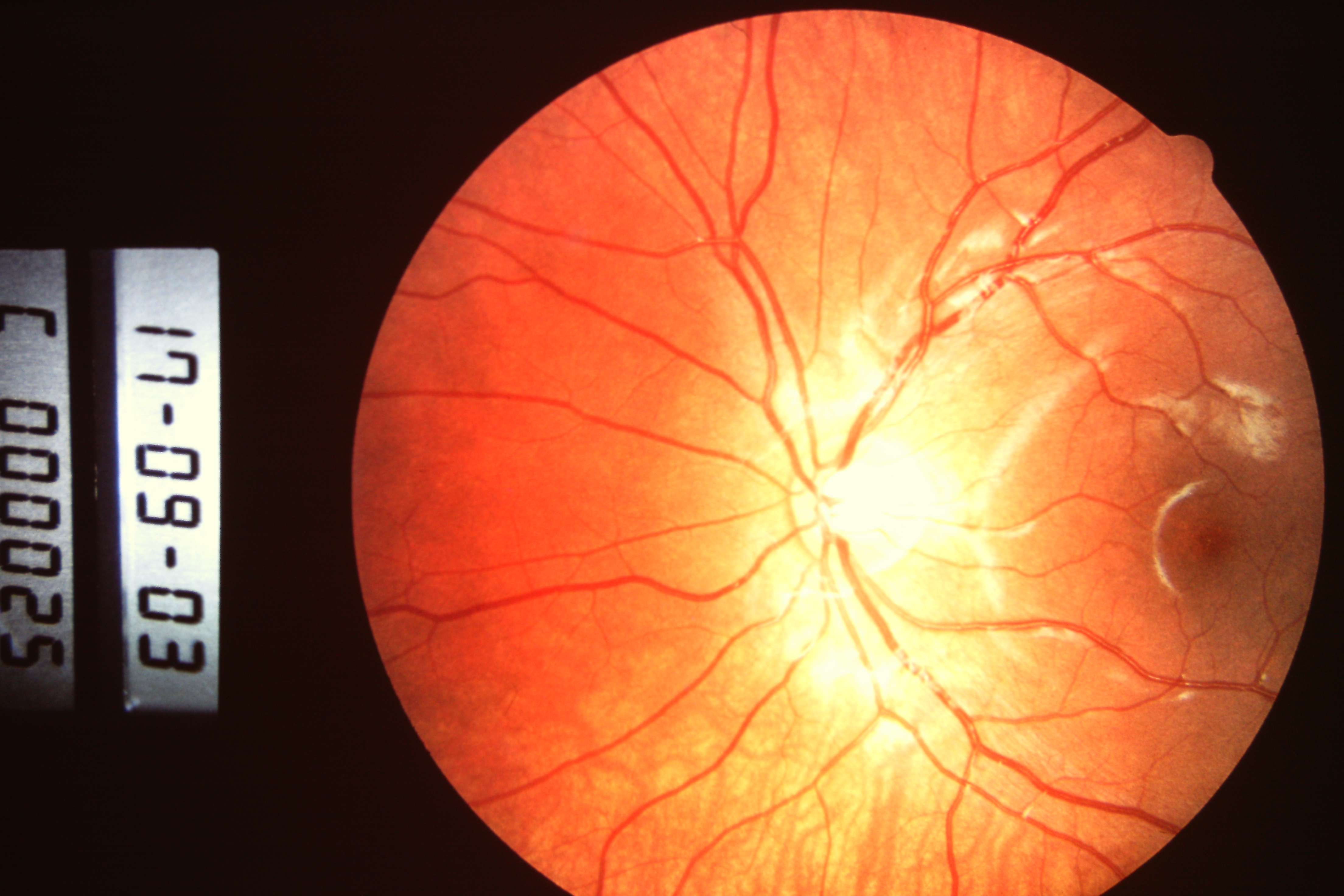

Supplement: Supplementary file 12 — Source Data for Figure 6 [file EMMM-14-e15809-s004.zip › Figure 6 Source Data/Figure 6 source data images/Fig6A BA PICT0049.JPG]

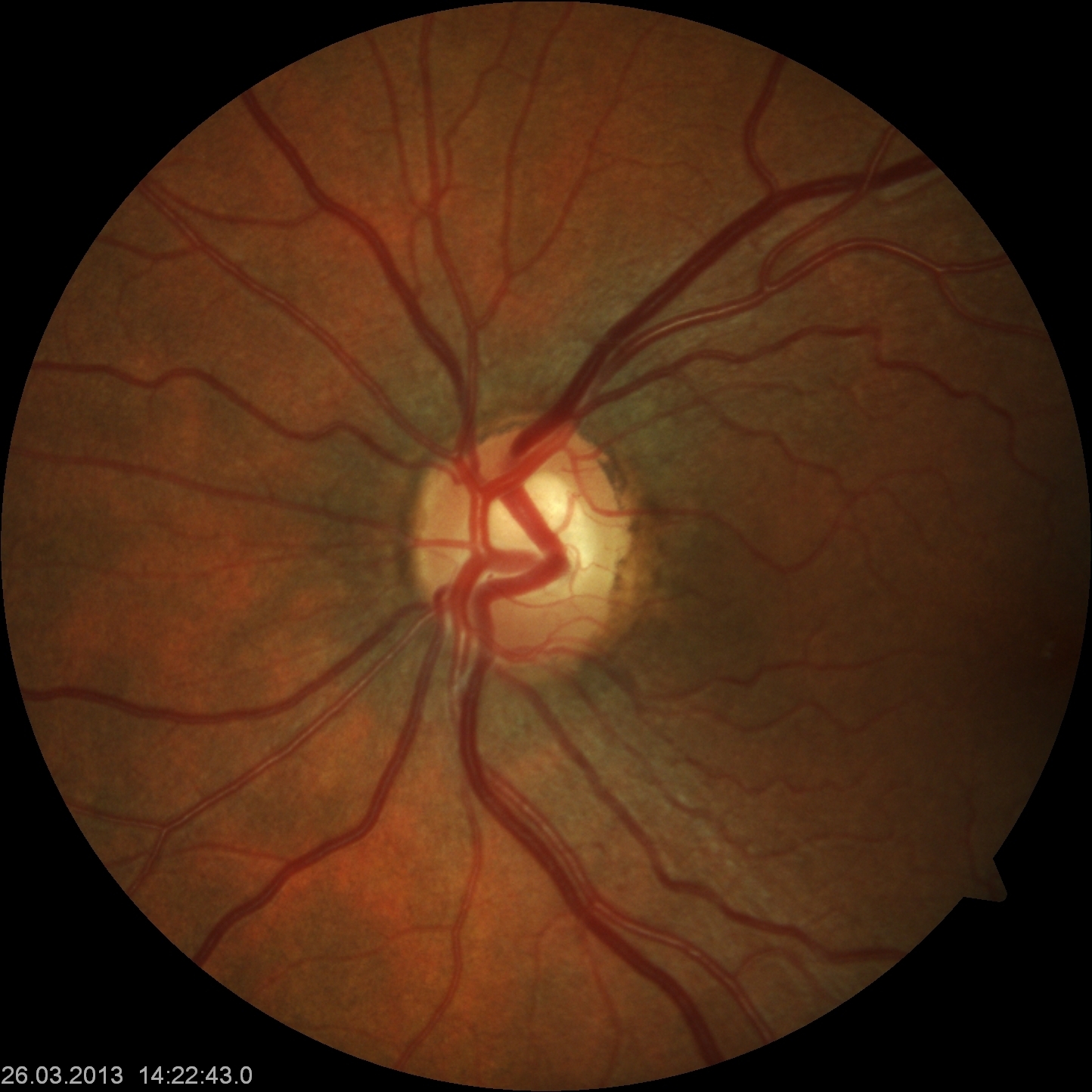

Supplement: Supplementary file 12 — Source Data for Figure 6 [file EMMM-14-e15809-s004.zip › Figure 6 Source Data/Figure 6 source data images/Fig6A CADASIL 5 OS.jpg]

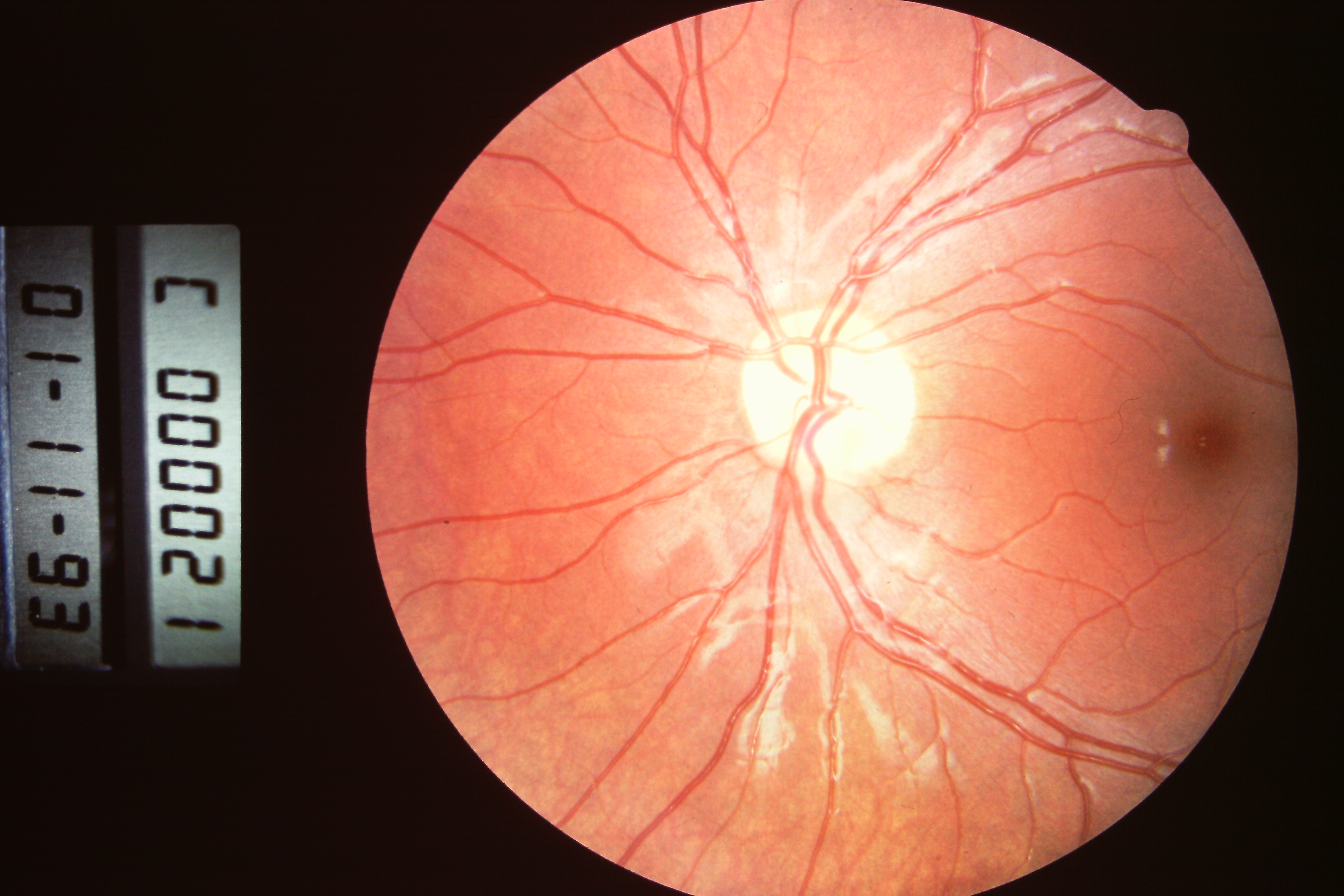

Supplement: Supplementary file 12 — Source Data for Figure 6 [file EMMM-14-e15809-s004.zip › Figure 6 Source Data/Figure 6 source data images/Fig6A pediatric CTRL PICT0174.JPG]
