# Supplementary material for: Sex differences and risk factors for bleeding in Alagille syndrome
Source: EMBO Mol Med. 2022 Nov 8;14(12):e15809. doi: 10.15252/emmm.202215809 (PMC9728057; doi:10.15252/emmm.202215809)
Supplement: Supplementary file 13 — PDF+ [file EMMM-14-e15809-s014.pdf]

Article

SOURCE DATA TRANSPARENT PROCESS OPEN ACCESS

EMBO Molecular Medicine

Sex differences and risk factors for bleeding in Alagille syndrome

Simona Hankeova[1,2], Noemi Van Hul[1], Jakub Laznovsky[3], Elisabeth Verboven[1], Katrin Mangold[1], Naomi Hensens[1,4], Csaba Adori[5], Elvira Verhoef[1,4], Tomas Zikmund[3], Feven Dawit[6], Michaela Kavkova[3], Jakub Salplachta[3], Marika Sjöqvist[1], Bengt R Johansson[7], Mohamed G Hassan[8,9], Linda Fredriksson[10], Karsten Baumgärtel[11], Vitezslav Bryja[2], Urban Lendahl[1], Andrew Jheon[8], Florian Alten[12], Kristina Teär Fahnehjelm[13,14], Björn Fischler[6], Jozef Kaiser[3] & Emma R Andersson[1,*]

Abstract

Spontaneous bleeds are a leading cause of death in the pediatric *JAG1*-related liver disease Alagille syndrome (ALGS). We asked whether there are sex differences in bleeding events in patients, whether *Jag1*$^{Ndr/Ndr}$ mice display bleeds or vascular defects, and whether discovered vascular pathology can be confirmed in patients non-invasively. We performed a systematic review of patients with ALGS and vascular events following PRISMA guidelines, in the context of patient sex, and found significantly more girls than boys reported with spontaneous intracranial hemorrhage. We investigated vascular development, homeostasis, and bleeding in *Jag1*$^{Ndr/Ndr}$ mice, using retina as a model. *Jag1*$^{Ndr/Ndr}$ mice displayed sporadic brain bleeds, a thin skull, tortuous blood vessels, sparse arterial smooth muscle cell coverage in multiple organs, which could be aggravated by hypertension, and sex-specific venous defects. Importantly, we demonstrated that retinographs from patients display similar characteristics with significantly increased vascular tortuosity. In conclusion, there are clinically important sex differences in vascular disease in ALGS, and retinography allows non-invasive vascular analysis in patients. Finally, *Jag1*$^{Ndr/Ndr}$ mice represent a new model for vascular compromise in ALGS.

Keywords Alagille syndrome; Bleeding; Jagged1; Notch; Vasculature
Subject Categories Cardiovascular System; Vascular Biology & Angiogenesis

DOI 10.15252/emmm.202215809 | Received 1 February 2022 | Revised 27 September 2022 | Accepted 5 October 2022 | Published online 8 November 2022
EMBO Mol Med (2022) 14: e15809

Introduction

Alagille syndrome (ALGS) is a pediatric disorder characterized by liver and heart defects, vertebral abnormalities, distinctive facial features, and posterior embryotoxon (Alagille *et al*, 1975; Emerick *et al*, 1999). However, up to 25% of deaths in these patients are attributed to intracranial hemorrhage (Emerick *et al*, 1999; Quiros-Tejeira *et al*, 1999; Kamath *et al*, 2004). Vascular defects in ALGS represent a significant burden of the disease (Hoffenberg *et al*, 1995; Emerick *et al*, 1999; Lykavieris *et al*, 2003; Kamath *et al*, 2004) and lead to transplant-associated complications (Kamath *et al*, 2004). It is currently not well understood why some patients experience spontaneous bleeds. No animal model has been reported for ALGS bleeding, and risk factors for bleeding have not been systematically addressed. Furthermore, vascular disease is currently assessed in patients using computed tomography (CT) or magnetic resonance imaging which necessitates exposure to radiation and can require sedation, making analysis in pediatric patients medically and ethically challenging.

Alagille syndrome is caused by mutations in the Notch ligand *JAGGED1* or the receptor *NOTCH2* (Li *et al*, 1997; Oda *et al*, 1997;

1 Department of Cell and Molecular Biology, Karolinska Institutet, Stockholm, Sweden
2 Department of Experimental Biology, Masaryk University, Brno, Czech Republic
3 CEITEC – Central European Institute of Technology, Brno University of Technology, Brno, Czech Republic
4 University of Applied Sciences Utrecht, Utrecht, The Netherlands
5 Department of Neuroscience, Karolinska Institutet, Stockholm, Sweden
6 Department of Pediatrics, Clinical Science, Intervention and Technology (CLINTEC), Karolinska Institutet and Karolinska University Hospital, Huddinge, Sweden
7 EM Unit, Institute of Biomedicine, University of Gothenburg, Gothenburg, Sweden
8 University of San Francisco, San Francisco, CA, USA
9 Department of Orthodontics, Faculty of Dentistry, Assiut University, Assiut, Egypt
10 Department of Medical Biochemistry and Biophysics, Karolinska Institutet, Stockholm, Sweden
11 Travere Therapeutics, San Diego, CA, USA
12 Department of Ophthalmology, University of Muenster Medical Center, Münster, Germany
13 Department of Pediatric Ophthalmology, Strabismus, Electrophysiology and Ocular Oncology, St. Erik Eye Hospital, Karolinska Institutet, Stockholm, Sweden
14 Department of Clinical Neuroscience, Karolinska Institutet, Stockholm, Sweden
*Corresponding author. Tel: +46852487360; E-mail: emma.andersson@ki.se

© 2022 The Authors. Published under the terms of the CC BY 4.0 license.

McDaniell *et al*, 2006). Notch signaling is a major regulator of blood vessel development, with key roles in blood vessel formation and maturation (Benedito *et al*, 2009; Phng & Gerhardt, 2009; Henshall *et al*, 2015). Notch mutations are the cause of at least two congenital diseases with vascular involvement: Cerebral Autosomal Dominant Arteriopathy with Subcortical Infarcts and Leukoencephalopathy (CADASIL, mutations in *NOTCH3*; Joutel *et al*, 1996) and Adams Oliver syndrome (mutations in *NOTCH1*, *DLL4* or *RBPJk*, for review see Mašek & Andersson (2017)). It has been suggested that ALGS should also be considered a vascular disorder (Crosnier *et al*, 2000; Lykavieris *et al*, 2003; Kamath *et al*, 2004; Mašek & Andersson, 2017). The association of Notch mutations with congenital vascular disorders underscores the need for appropriate animal models of Notch-related vascular disease, in which to test potential therapies. The recently reported $Jag1^{Ndr/Ndr}$ mouse model for ALGS recapitulates cholestatic liver disease in postnatal pups (Andersson *et al*, 2018) with spontaneous liver regeneration in adults (Hankeova *et al*, 2021), as well as cardiac phenotypes and ocular anomalies (Andersson *et al*, 2018). This mouse model could thus provide an important tool for preclinical studies.

There are many possible bleeding risk factors, that could modify risk in patients with ALGS, but these have not yet been systematically addressed in patients nor in mouse models. Chronic cholestatic liver disease is associated with coagulopathy (Tiede *et al*, 2022), although patients have explicitly been reported with bleeds in the absence of coagulopathy (O'Connell *et al*, 2012; Fiorda-Diaz *et al*, 2017). Bone integrity, and skull in particular, is also compromised in some patients with ALGS (Hoffenberg *et al*, 1995; Volz *et al*, 2015), which could increase the risk of intracranial bleeds. Furthermore, although no sex differences have been reported in ALGS, female sex is a risk factor for prevalence of intracranial aneurysms, aneurysm growth, and subarachnoid hemorrhage (Fuentes *et al*, 2022). Finally, high blood pressure increases the risk of aneurysmal subarachnoid hemorrhage (Vlak *et al*, 2013), but it is not known whether blood pressure is affected in ALGS, nor how this may interact with potential vascular defects. There are thus multiple relevant risk factors for bleeding risk that should be investigated in patients and/or animal models to understand the etiology of bleeding in patients.

Using a systematic review following the PRISMA guidelines, we found that spontaneous intracranial bleeds are significantly more often reported in girls than boys with ALG. Our analysis of the ALGS mouse model ($Jag1^{Ndr/Ndr}$ mice) showed reduced postnatal survival and sporadic spontaneous bleeding, in the brain and in other organs. Risk factor analysis revealed no evidence of coagulopathy in the cholestatic $Jag1^{Ndr/Ndr}$ mice. Instead, $Jag1^{Ndr/Ndr}$ mice had thinner skulls and vascular defects, with the latter being significantly exacerbated by hypertension. Interestingly, female sex specifically aggravated a reduction in veins and increase in venous tortuosity in $Jag1^{Ndr/Ndr}$ mice. $Jag1^{Ndr/Ndr}$ mice thus represent the first reported model for translational research for bleeding in ALGS. Vascular tortuosity, discovered in the ALGS mouse model, was significantly increased in retinographs from pediatric patients with ALGS, providing a new method for non-invasive assessment of vascular health in ALGS. In sum, drawing on patient and animal data, this study provides evidence for multiple risk factors modulating vascular health in ALGS, including hypertension, frail bones, and sex, and demonstrates that retinography could be further investigated as a clinical tool for monitoring patient vascular health.

Results

Patients with Alagille syndrome exhibit sporadic spontaneous and provoked hemorrhages, with sex differences

Sex is not thought to impact the prevalence of vascular defects or intracranial bleeds in ALGS (Kamath *et al*, 2004; Emerick *et al*, 2005; Carpenter *et al*, 2018). However, sex-based differences in cardiovascular disease, stroke, and intracranial bleeds in the general population have been reported (Appelros & Åsberg, 2020; Zuurbier *et al*, 2022). To test if sex has an impact on vascular abnormalities in patients with ALGS we performed a systematic review, adhering to the PRISMA guidelines (Page *et al*, 2021), and assessed the full text of 771 publications. We identified 107 reports with 172 patients with ALGS, sex data available, and vascular structural defects or vascular events (Fig 1A; Appendix Fig S1 and Appendix Table S1, for search terms and strategy see Appendix Supplementary Methods). There were no sex differences in the most frequently reported structural abnormalities: stenoses, collateral vessels/occlusions or aneurysms (Appendix Fig S1A) nor in the most frequently reported functional events: overall bleeding events, surgery-related vascular complications or ischemic events (Appendix Fig S1B).

Because intracranial bleeding causes more deaths than liver disease in ALGS (Emerick *et al*, 1999), we analyzed these events in greater detail (Table 1). Intracranial bleeds were the consequence of minor head trauma often associated with a paper-thin skull, coagulopathy induced by liver cholestasis, or were spontaneous (Fig 1B and Table 1). Patients with minor head trauma or coagulopathy exhibited intracranial bleeding at a young age, on average 5.9 years for males and 8.3 years for females, and there were no sex differences in trauma- or coagulopathy-associated intracranial bleeds (Table 1). In contrast, there were significantly more spontaneous brain hemorrhages in females than males (10 vs. 1, Fig 1B and Table 1, two-sided binominal exact test $P = 0.0117$). Spontaneous brain hemorrhages often resulted from a ruptured aneurysm and occurred in older patients, specifically at 13 years for the one male and on average 16.5 years in the 10 females (age range: 4-30 years).

In sum, this analysis systematically aggregates all available bleeding and vascular data for patients with ALGS and shows that female sex is associated with spontaneous brain hemorrhage, often caused by a ruptured aneurysm (Fig 1B and Table 1).

Cholestatic $Jag1^{Ndr/Ndr}$ mice exhibit normal coagulation but a thinner skull and sporadic spontaneous and provoked hemorrhages

To investigate whether the $Jag1^{Ndr/Ndr}$ mouse model for ALGS liver disease also presents risk factors for bleeding, we analyzed parameters associated with bleeding events in cholestasis in general or in ALGS specifically, including coagulopathy and bone frailty. Forty-six $Jag1^{Ndr/Ndr}$ pups were monitored from postnatal day 0 (P0) to P10. Fourteen $Jag1^{Ndr/Ndr}$ pups died (30.4%), most of which were cannibalized by the mother, but one displayed an obvious brain bleed (also reported further below; Fig 2A). At P10, surviving $Jag1^{Ndr/Ndr}$ pups were jaundiced and total bilirubin was significantly increased, as previously reported (Andersson *et al*, 2018; Fig 2B). Despite cholestasis, coagulation activity was normal with wild type levels of Thrombin–Antithrombin complexes in blood plasma

 © 2022 The Authors

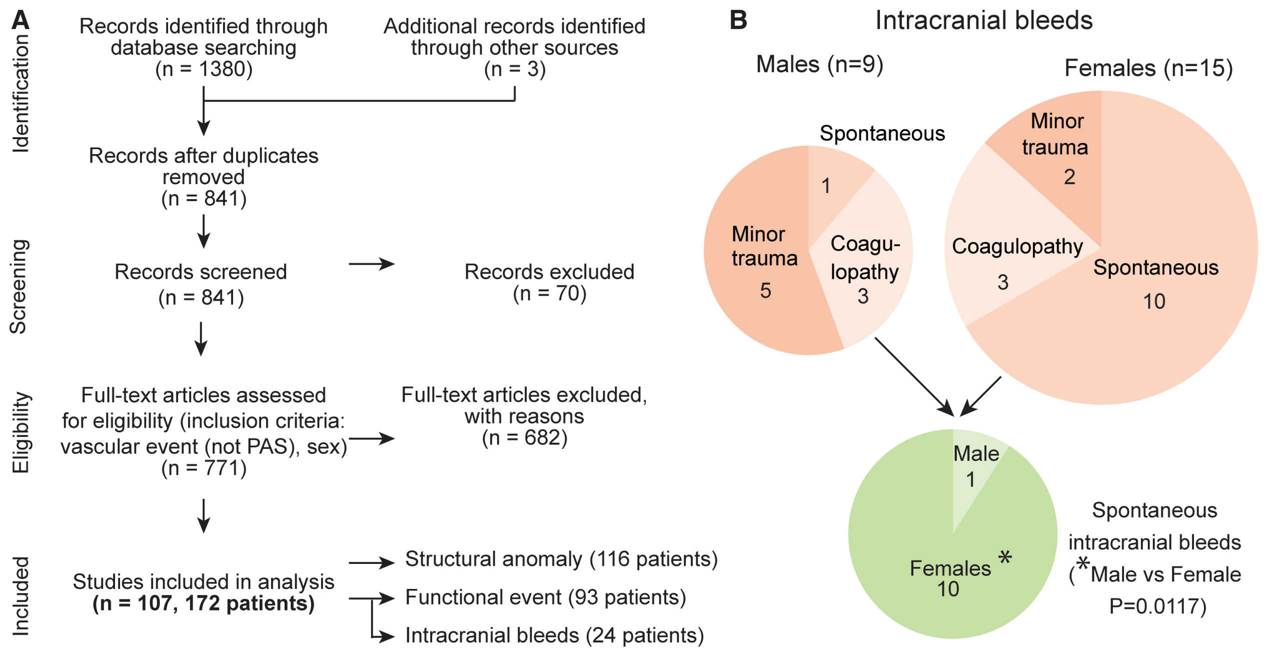

Figure 1. Patients with Alagille syndrome exhibit sporadic spontaneous and provoked hemorrhages, with sex differences.

A Systematic review search strategy following PRISMA guidelines. For detailed flow chart see Appendix Supplementary Methods. ALGS, Alagille syndrome; PAS, pulmonary artery stenosis.

B Schematic depicting intracranial bleeds ($n = 24$, Binominal test in 9 males vs 15 females, ns, $P = 0.2295$). They were reported as a consequence of either minor head trauma or coagulopathy, or they were spontaneous (pie charts denote reported patient numbers). Pie chart depicting the number of spontaneous intracranial bleeds (male $n = 1$, females $n = 10$, Binominal test *$P = 0.0117$). For details of intracranial bleeds and references, see Table 1.

Source data are available online for this figure.

(Fig 2C), fibrinogen in serum (Fig 2D), international normalized ratio (INR) in complete blood (Fig 2E), and tail bleeding time (Fig 2F). Analysis of P30 $Jag1^{Ndr/Ndr}$ skulls using micro CT (µCT) revealed bony protrusions at the intersection of the parietal, temporal, and occipital bones in $Jag1^{Ndr/Ndr}$ mice (Fig EV1A, blue arrows), and thinner cranial bones in $Jag1^{Ndr/Ndr}$ mice than in wild type mice which often consisted of a single layer of compact bone (Figs 2G–I and EV1B–E). $Jag1^{Ndr/Ndr}$ pups are strikingly smaller than wild type animals (Andersson *et al*, 2018) and, accordingly, the $Jag1^{Ndr/Ndr}$ adult skull lengths were shorter (Fig EV1F, ~ 94% of the wild type skull lengths for both males and females). However, the volume of the $Jag1^{Ndr/Ndr}$ cranial bone full thickness (Fig 2H, ~ 78.4% of wild type volume for males and 82.8% for females) and the compact bone (Fig 2I, ~ 77.9% of wild type volume for males and 81.5% for females) were both disproportionately and significantly lower than wild type skull volumes. We segmented out the temporal bone, which was reported as frail in patients with ALGS (Hoffenberg *et al*, 1995; Petaros *et al*, 2015), and the temporal bone volume was also significantly decreased in $Jag1^{Ndr/Ndr}$ mice (Fig EV1G and H).

To test vascular permeability and integrity we used tracers and resin injections (Hankeova *et al*, 2021). We used the Evans blue assay as a high molecular weight tracer (67 kDa when bound to serum albumin) and fluorescent tracers for low molecular weight tracers (1 kDa cadaverin and 3 kDa dextran) to assess permeability of different molecular sizes. There were no differences in vessel permeability, as assessed by Evans blue in adult $Jag1^{Ndr/Ndr}$ kidneys, hearts, or livers (Fig EV2A–C). To address if blood vessel barrier function is selectively affected for smaller sized molecules, we injected P30 mice with a mix of 3 kDa Dextran-FITC and 1 kDa Cadaverin-555. Despite adjusting injected volumes to the weight of each animal, the fluorescence signal was significantly higher in plasma of $Jag1^{Ndr/Ndr}$ mice (Fig EV2D and E), which could reflect autofluorescence of bilirubin itself in the cholestatic mice (Chen, 1973). Fluorescence signal was also significantly increased in the kidneys of $Jag1^{Ndr/Ndr}$ mice, which similarly could reflect either permeability of the small fluorescent tracers or of fluorescent bilirubin in cholestatic mice (Fig EV2F and G). In resin injection experiments, a synthetic resin is injected into blood vessels, which can provoke blood vessel rupture due to injection pressure. Extravasated resin reports the occurrence of vessel rupture as well as the volume. Two of six $Jag1^{Ndr/Ndr}$ livers (both from females) had 40x more resin outside of the portal vein than the average of control animals (leaks pseudo-colored red, Fig 2J and K) in these provoked blood vessel ruptures.

To investigate spontaneous vascular accidents in the nervous system, we next examined the brain and retina. We did not detect any changes in the blood–brain barrier permeability of $Jag1^{Ndr/Ndr}$ mice (Figs 2L and EV2H and I). Eighty-three $Jag1^{Ndr/Ndr}$ pups were monitored daily from birth until P10 (including the 46 described above in Fig 2A) and macroscopically obvious brain hemorrhages occurred in two $Jag1^{Ndr/Ndr}$ pups (2.41%) but were not observed in $Jag1^{+/+}$ or $Jag1^{+/Ndr}$ mice (Figs 2M and EV2J, blue arrowheads

Table 1. Overview of patients with Alagille syndrome reported with vascular abnormality.

Sex	Age (years)	IC event	Coagulation and bilirubin data	Provoked or Sporadic	Ref	Patient # (as in Fig 1 Source data)
Males	Neonatal	Subdural hematoma after birth trauma.	NR	Minor trauma	Emerick *et al* (2005)	107
	61 days	Intracranial hemorrhage. Recovered.	Vitamin K deficiency (cholestatic coagulopathy)	Coagulopathy	Matsuura *et al* (2013)	131
	22 months	Epidural hematoma, and parenchymal and intraventricular bleeds, after falling from a chair.	Normal coagulation factors	Minor trauma	Kazi *et al* (2018)	124
	2	Epidural hematoma after minor accidental head trauma. Fatal.	NR	Minor trauma	Hoffenberg *et al* (1995)	120
	2.3	Large epidural hematoma due to minor head trauma. Paper-thin cranial bones.	NR	Minor trauma	Hoffenberg *et al* (1995)	121
	6	Intracranial bleed after liver transplant. Fatal.	Mild disseminated intravascular coagulation	Coagulopathy	Emerick *et al* (1999, 2005)	108
	12	Hematoma at age 12 with right parietal fracture but no history of trauma. Stroke at age 18.	NR	Probably minor trauma	Quiros-Tejeira *et al* (1999)	139
	13	Subarachnoid hemorrhage.	NR	Spontaneous	Santamaria *et al* (2016)	152
	23	First event: ruptured basilar tip aneurysm.<br>Second event unclear: red blood cells in cerebrospinal fluid. Aneurysm of posterior communicating artery present but no evidence of rupture.<br>Aneurysm clipped and patient recovered.	At second event: 16.1 s Prothrombin 40.6 s partial thrompboplastin total bilirubin 8.6 mg/dl	Coagulopathy	Cowan *et al* (2004)	106
Females	10 weeks	Subdural hematoma, posterior fossa and around tentorium.	Raised prothrombin time. Raised activated partial prothrombin time. Thrombin and fibrinogen normal. Conjugated hyperbilirubinemia	Coagulopathy	Vorstman *et al* (2003)	69
	5 months	Intracerebral hemorrhage.<br>Fatal.<br>Multiple large, thin-walled vessels at autopsy.	Hemoglobin 5.9 g/dl. Prothrombin time 20 s (control of 15), Total bilirubin 8.8 mg/dl, direct 5.2 mg/dl	Coagulopathy	Agrawal *et al* (2015)	7
	22 months	Evidence of old intracerebral hemorrhage on CT scan.	NR for time of hemorrhage	Spontaneous	Rachmel *et al* (1989)	49
	2	Intracerebral hemorrhage.<br>Fatal.<br>Multiple large, thin-walled vessels at autopsy.	Normal coagulation values	Spontaneous	Hoffenberg *et al* (1995)	34
	4	Epidural hematoma after fall.<br>Fatal.<br>At autopsy: thinning of arterial walls with apoptotic muscle cell layer and thin cranial bones, esp temporal bones.	NR	Minor trauma	Petaros *et al* (2015)	46
	4	MCA territory stroke and right frontal stroke after cardiac procedure.<br>Moyamoya syndrome.<br>EDAS (encephaloduroarteriosynangiosis) for revascularization.<br>Fatal left thalamic hemorrhage 2 years after EDAS.	NR	Spontaneous	Baird *et al* (2015)	11
	9	Subarachnoid hemorrhage after minor head trauma.	NR	Minor trauma	Emerick *et al* (2005)	20

© 2022 The Authors

Table 1 (continued)

Sex	Age (years)	IC event	Coagulation and bilirubin data	Provoked or Sporadic	Ref	Patient # (as in Fig 1 Source data)
	17	Subarachnoid hemorrhage from superior cerebellar artery aneurysm. Aneurysms coiled and patient recovered.	"Laboratory testing was insignificant other than urea of 8.2 and deranged liver function tests (. . .)"	Spontaneous	O'Connell *et al* (2012)	44
	17	First event: intracranial epidural hematoma. Second event: Subarachnoid hemorrhage from communicating artery aneurysm. Recovered.	"Laboratory tests were unremarkable"	Spontaneous	Fiorda-Diaz *et al* (2017)	28
	20	Intracranial hemorrhage due to ruptured middle cerebral artery aneurysm. Fatal.	NR	Spontaneous	Emerick *et al* (2005)	21
	21	Catastrophic subarachnoid hemorrhage and intraparenchymal hematoma. Possible basilar artery aneurysm rupture. Fatal.	NR	Spontaneous	Tumialán *et al* (2006)	65
	25	Subarachnoid hemorrhage from large saccular ruptured terminal basilar artery aneurysm. Fatal.	NR. Autopsy	Spontaneous	Doberentz *et al* (2015)	18
	28	Cerebral and ocular hemorrhage. Urgent orthotopic liver transplant, recovered.	INR 3.18<br>Hemoglobin 5.9 g/dl<br>Platelets 90,000/mm^3<br>Total bilirubin 63 mg/dl	Coagulopathy	Frongillo *et al* (2015)	30
	28	Subarachnoid hemorrhage from vertebrobasilar junction aneurysm. Stent exclusion of aneurysm, patient recovered.	Total bilirubin 1.3 mg/dl<br>Alkaline phosphatase 444 units/l [normal 40–125 units/l]	Spontaneous	Gaba *et al* (2008)	31
	30	Subarachnoid hemorrhage due to a ruptured 8-mm aneurysm of the right supraclinoid internal carotid artery. Right parietal subdural hematoma. Surgical clipping of aneurysm, patient recovered.	NR	Spontaneous	Schlosser *et al* (2004)	59

Summary of patients identified in Systematic Review, with ALGS and intracranial bleeding, including type of bleed, cause or associated insult, and sex data. These data are the basis of Fig 1B. IC, intracranial; NR, not reported.

indicate bleeds in head region, sex n.d.). Surviving $Jag1^{Ndr/Ndr}$ pups sporadically displayed retinal hemorrhage (Fig 2N, sex n.d.) or leaky retinal arterioles (Fig 2O, sex n.d.).

In summary, our data show that at least 2–3% of $Jag1^{Ndr/Ndr}$ mice exhibit spontaneous lethal central nervous system bleeds within 10 days of birth. $Jag1^{Ndr/Ndr}$ mice recapitulate spontaneous and rare bleeds in different organs, in the absence of coagulation defects. As described in some patients with ALGS and intracranial hemorrhage (Hoffenberg *et al*, 1995; Petaros *et al*, 2015, Table 1), thinner intracranial bones in $Jag1^{Ndr/Ndr}$ mice may also contribute to nervous system bleeds.

$Jag1^{Ndr/Ndr}$ mice display vascular guidance defects, with fewer and more tortuous blood vessels

Abnormal blood vessel growth or patterning can cause changes in blood flow leading to vessel occlusion (Qiao *et al*, 2019), changes in shear stress (Secomb, 2016) and changes to the vessel wall resulting in blood vessel tortuosity/dolichoectasia associated with vascular lesions and stroke (Han, 2012). To analyze the impact of abnormal JAG1 signaling on blood vessel growth and patterning we investigated retinal vessels from P5 until adulthood. $Jag1^{Ndr/Ndr}$ mice displayed abnormal vascular development phenocopying the *Jag1* endothelial cell (EC) knockout (Benedito *et al*, 2009). Retinal angiogenesis takes place postnatally during the first 3 weeks after birth (Stahl *et al*, 2010; Fig EV3A). During these stages, the $Jag1^{Ndr/Ndr}$ vasculature displayed delayed outgrowth (Fig EV3B and C) with abnormal tip cell morphology (Fig EV3D–F). Primary vascular plexus remodeling was defective during the first 15 postnatal days in $Jag1^{Ndr/Ndr}$ mice (Fig EV3G–M). The delay in vascular growth and remodeling was accompanied by decreased EC proliferation (Fig EV3N) and DLL4 upregulation in the vascular front (Fig EV3O and P).

Next, we quantified the occurrence of arteriovenous crossings, which are pathological in mouse retina (Martin *et al*, 2020), and found an average of two arteriole/venule crossings per $Jag1^{Ndr/Ndr}$ retina in both males and females (Fig 3A and B). $Jag1^{Ndr/Ndr}$ males and females had significantly fewer arterioles (Fig 3C) compared to same sex $Jag1^{CTRL}$ mice and $Jag1^{Ndr/Ndr}$ females had fewer venules compared to both $Jag1^{Ndr/Ndr}$ males and $Jag1^{CTRL}$ females (Fig 3D, Two-way ANOVA shows a significant interaction between sex and genotype, Fig 3 Source Data). Arterial tortuosity was increased in

three of six *Jag1*$^{Ndr/Ndr}$ mice at P30 and in one of six *Jag1*$^{Ndr/Ndr}$ mice at 1 year (Fig 3E) independent of sex (Fig 3F) but the overall differences were not statistically significant. In contrast, venous tortuosity was significantly increased in *Jag1*$^{Ndr/Ndr}$ mice at P30 compared to *Jag1*$^{+/+}$ mice (Fig 3G). Genotype and sex showed a significant interaction and impact on venous tortuosity, with the greatest increase in female *Jag1*$^{Ndr/Ndr}$ mice (Fig 3H, Two-way ANOVA shows a significant interaction between sex and genotype, Fig 3 Source Data). We further analyzed the tortuosity and branching of the middle cerebral artery (MCA; Fig 3I), which has been reported to be affected in patients with ALGS (Emerick *et al*, 1999; Woolfenden *et al*, 1999; Rocha *et al*, 2012). MCA tortuosity and branching were abnormal in two of six *Jag1*$^{Ndr/Ndr}$ mice (Movies EV1 and EV2), but the differences were not statistically significant at the population level (Fig 3J–L). Together, our data demonstrate patterning defects in major arterioles and venules of *Jag1*$^{Ndr/Ndr}$ mice, with sex-specific differences in the numbers of venules and venous tortuosity.

Jag1$^{Ndr/Ndr}$ mice display sparse arteriole vascular smooth muscle cell coverage that is exacerbated upon hypertension

Blood vessels are composed of two principal cell types: ECs and mural cells, which include VSMCs and pericytes. The foremost Notch-related vascular disorder, CADASIL, is characterized by poor arterial VSMC coverage of arteries (Joutel *et al*, 1996). We therefore

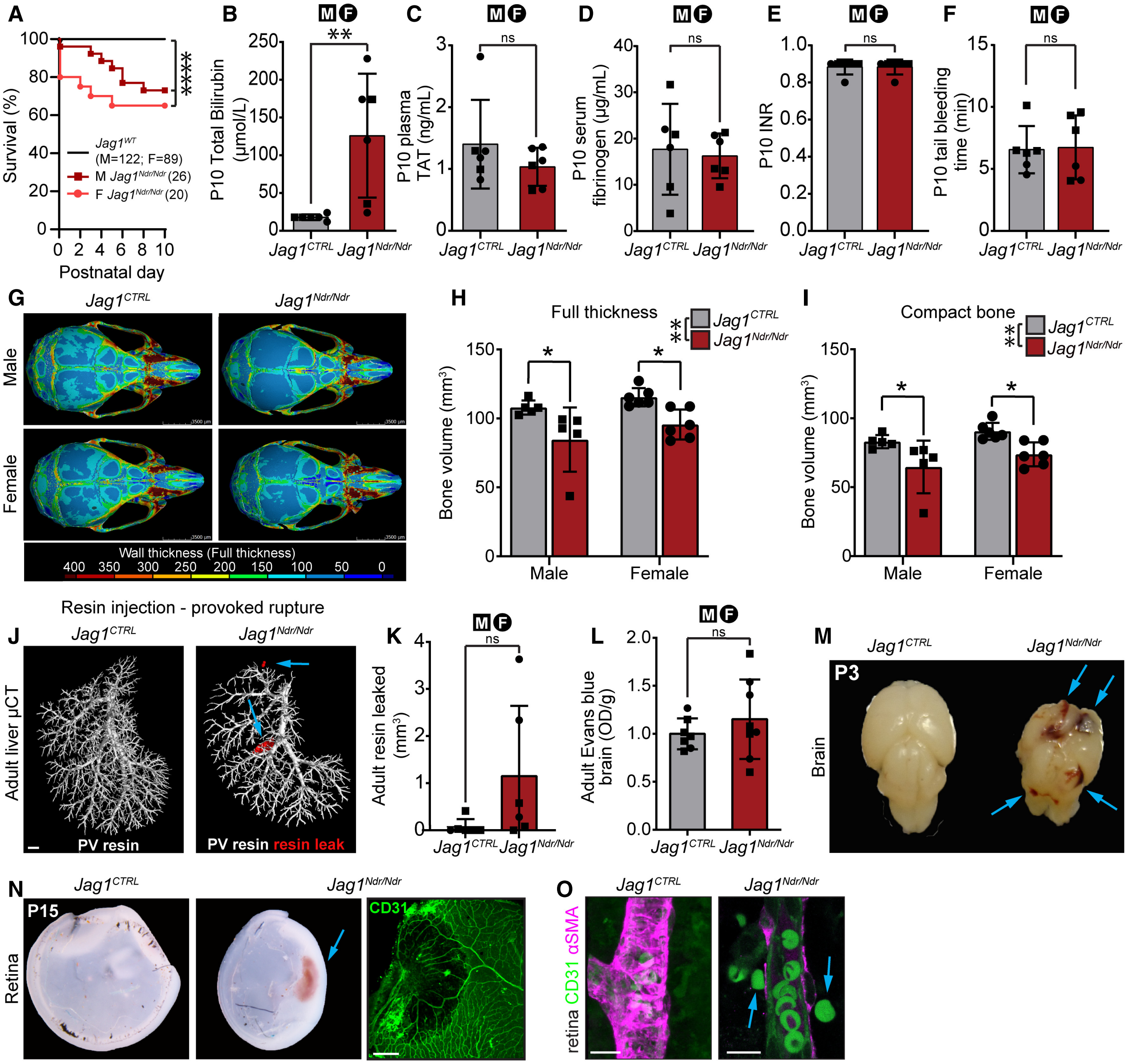

Figure 2.

© 2022 The Authors

Figure 2. Cholestatic *Jag1*$^{Ndr/Ndr}$ mice exhibit normal coagulation but a thinner skull and sporadic spontaneous and provoked hemorrhages.

A Pup survival analysis between P0 and P10, by sex. Each dot represents the absolute percent of remaining animals per group (*Jag1*^{CTRL} $n = 211$, male *Jag1*$^{Ndr/Ndr}$ $n = 26$, female *Jag1*$^{Ndr/Ndr}$ $n = 20$, log-rank test, ****$P < 0.0001$).

B P10 plasma total bilirubin levels ($n = 6$ per group, unpaired *t*-test, **$P = 0.0091$).

C P10 plasma thrombin antithrombin levels ($n = 6$ per group, unpaired *t*-test, ns, $P = 0.2788$).

D P10 serum fibrinogen levels ($n = 6$ per group, unpaired *t*-test, ns, $P = 0.7585$).

E P10 whole blood prothrombin time (INR; $n = 6$ per group, unpaired *t*-test, ns, $P > 0.9999$).

F P10 tail bleeding time ($n = 6$ per group, unpaired *t*-test, ns, $P = 0.8916$).

G–I µCT of P30 skulls. (G) Color map depicting wall thickness. Scale bar 3.5 mm. (H) Skull full thickness volume (n = 5-6 per group, two-way ANOVA. Interaction $P = 0.7595$; Sex $P = 0.1164$, Genotype **$P = 0.0012$. Šídák's multiple comparisons test: Male *Jag1*$^{+/+}$ vs *Jag1*$^{Ndr/Ndr}$ *$P = 0.0227$; Female *Jag1*$^{+/+}$ vs *Jag1*$^{Ndr/Ndr}$ *$P = 0.034$), (I) Compact bone total volume (n = 5-6 per group, two-way ANOVA. Interaction $P = 0.8631$; Sex $P = 0.0901$, Genotype **$P = 0.0014$. Šídák's multiple comparisons test: Male *Jag1*$^{+/+}$ vs *Jag1*$^{Ndr/Ndr}$ *$P = 0.0305$; Female *Jag1*$^{+/+}$ vs *Jag1*$^{Ndr/Ndr}$ *$P = 0.0307$).

J, K (J) Provoked vascular accidents induced by portal vein resin injection, with (K) resin leakage quantification outside of blood vessels (red) in adult mice ($n = 6$ per group, unpaired *t*-test, ns, $P = 0.1088$). Scale bar 1 mm.

L Adult brain Evans blue assay (*Jag1*^{CTRL} $n = 7$, *Jag1*$^{Ndr/Ndr}$ $n = 8$, unpaired *t*-test, ns, $P = 0.3807$).

M Hemorrhagic P3 *Jag1*$^{Ndr/Ndr}$ brain (n = 2/83 *Jag1*$^{Ndr/Ndr}$ mice, sex n.d.).

N Hemorrhagic P15 *Jag1*$^{Ndr/Ndr}$ retina. Scale bar 20 µm. Sex n.d.

O Red blood cells (green outside P10 *Jag1*$^{Ndr/Ndr}$ retinal arteriole. Scale bar 10 µm.

Data information: Bar graphs depict mean values ± standard deviation, each dot represents one biological replicate. Circles represent females, squares represent males. For details/results of statistical analyses, please see source data. µCT, micro computed tomography; n.d., not determined; OD, optical density; TAT, thrombin antithrombin; P(X), postnatal day X.

Source data are available online for this figure.

investigated VSMCs and pericytes (a cell required for blood–brain/retina barrier integrity; Armulik *et al*, 2010) in *Jag1*$^{Ndr/Ndr}$ mice. In adult *Jag1*$^{Ndr/Ndr}$ retinas, CD13+ pericyte coverage was similar to that in *Jag1*^{CTRL} retinas for both male and female mice (Fig EV4A and B). We therefore focused on VSMC development and homeostasis. At P10, α-smooth muscle actin positive (αSMA+) VSMC morphology in *Jag1*$^{Ndr/Ndr}$ retinas was less mature with some cell bodies oriented parallel to the blood vessel axis rather than perpendicular (Fig 4A top panel, yellow arrows, magnified in inset). At P15, *Jag1*$^{Ndr/Ndr}$ VSMC arteriole coverage was incomplete and parallel-oriented VSMCs were still present (Fig 4B yellow arrows, boxed region). At P30, *Jag1*$^{Ndr/Ndr}$ arteriolar VSMCs exhibited a more mature morphology with mostly perpendicular VSMCs, but with sparse coverage and occasional parallel orientation (Fig 4C yellow arrows). Adult *Jag1*$^{Ndr/Ndr}$ mice (at 3–6 months) displayed sparse VSMC arteriole coverage, stenoses, and sporadically larger gaps (Fig 4D, stenosis green arrowheads). By 1 year of age, some *Jag1*$^{Ndr/Ndr}$ arteriolar VSMCs degenerated, resulting in αSMA-negative areas (Fig 4E white arrowheads, F, and G). αSMA-negative gaps increased in frequency with age in *Jag1*$^{Ndr/Ndr}$ mice, with 50% of mice displaying gaps at 1 year of age (Fig 4F), and the number of gaps per retina also increasing with age (Fig 4G). αSMA-negative areas were sometimes associated with aneurysms in *Jag1*$^{Ndr/Ndr}$ arterioles (Fig 4E white arrows). Transmission electron microscopy of retinal blood vessels confirmed sparse and thin VSMCs in *Jag1*$^{Ndr/Ndr}$ arterioles, which were not in contact with neighboring VSMCs (Fig 4H blue arrowheads, VSMCs pseudo-colored magenta and ECs green). *Jag1*$^{Ndr/Ndr}$ coronary artery VSMCs displayed similar gaps (Fig EV4C, white arrowheads), indicating that this VSMC defect is not specific to the nervous system.

VSMCs are diverse and can perform both contractile and synthetic functions (Rensen *et al*, 2007). To determine whether *Jag1*$^{Ndr/Ndr}$ VSMCs differ in the expression of contractile (αSMA, SM22, MYH11) and synthetic (VIM, COLI) markers, we evaluated their expression in VSMCs and in the αSMA-negative gaps. Arterial αSMA and SM22 were increased in *Jag1*$^{Ndr/Ndr}$ mice, while levels of VIM were unaffected (Fig EV4D and E). The contractile VSMC markers in *Jag1*$^{Ndr/Ndr}$ mice were downregulated 80–90% in gaps between αSMA+ VSMC, while COLI was decreased by 67% and VIM was decreased by 30% (Fig EV4D, F, and G). Furthermore, the changes in *Jag1*$^{Ndr/Ndr}$ VSMC coverage led to reactivity in the parenchyma as evidenced by an increase in astrocyte density surrounding *Jag1*$^{Ndr/Ndr}$ arterioles (Fig EV4H and J), and the presence of numerous reactive bundles (Fig EV4H, white arrowheads). The astrocytes surrounding venules were similar in *Jag1*^{CTRL} and *Jag1*$^{Ndr/Ndr}$ retinas (Fig EV4I and K). Finally, we tested whether *Jag1*$^{\Delta Dsl/+}$ mice, an alternative model for ALGS (Thakurdas *et al*, 2016) displayed similar phenotypes. Analysis of *Jag1*$^{\Delta Dsl/+}$mice revealed, like *Jag1*$^{Ndr/Ndr}$ mice, a reduction in the number of major arterioles and venules (Appendix Fig S2A and B) and increased number of arteriovenous crossings (Appendix Fig S2C and D). Interestingly, unlike *Jag1*$^{Ndr/Ndr}$ mice, at 1 year of age they did not display severe sparse VSMC coverage, nor a significant difference in αSMA fluorescence intensity coverage of arterioles, nor a statistically significant increase in GFAP+ reactive astrocyte coverage (Appendix Fig S2E–G). *Jag1*$^{Ndr/Ndr}$ mice thus exhibit a more severe phenotype than *Jag1*$^{\Delta Dsl/+}$ mice.

We next investigated whether apoptosis contributes to the generation of αSMA-negative regions in 1-year-old *Jag1*$^{Ndr/Ndr}$ mice (Fig 4I and J) since VSMC apoptosis is increased in *Notch3* mutant mice (Henshall *et al*, 2015). At this stage, the number of first-generation branch points was normal in *Jag1*$^{Ndr/Ndr}$ arterioles (Fig EV4L). Surprisingly, *Jag1*$^{Ndr/Ndr}$ arterioles displayed fewer cCasp3+ ECs at branching points (Fig EV4M) but significantly more cCasp3+ along the arteriole length, often associated with αSMA-negative areas (Fig 4J, note the aneurysm formation in the αSMA-negative area). To determine whether the sparse retinal VSMC coverage was representative of brain vasculature we imaged MCA VSMC coverage in P10 mice. The *Jag1*$^{Ndr/Ndr}$ arterial VSMC showed fewer VSMCs per area (Fig 4K and L), confirming that a paucity of VSMCs is present both in neural retina and in brain.

Next, we examined whether high blood pressure, a major risk factor for subarachnoid hemorrhage (O'Donnell *et al*, 2010; Han, 2012), affected vascular health in *Jag1*$^{Ndr/Ndr}$ mice. We treated

© 2022 The Authors

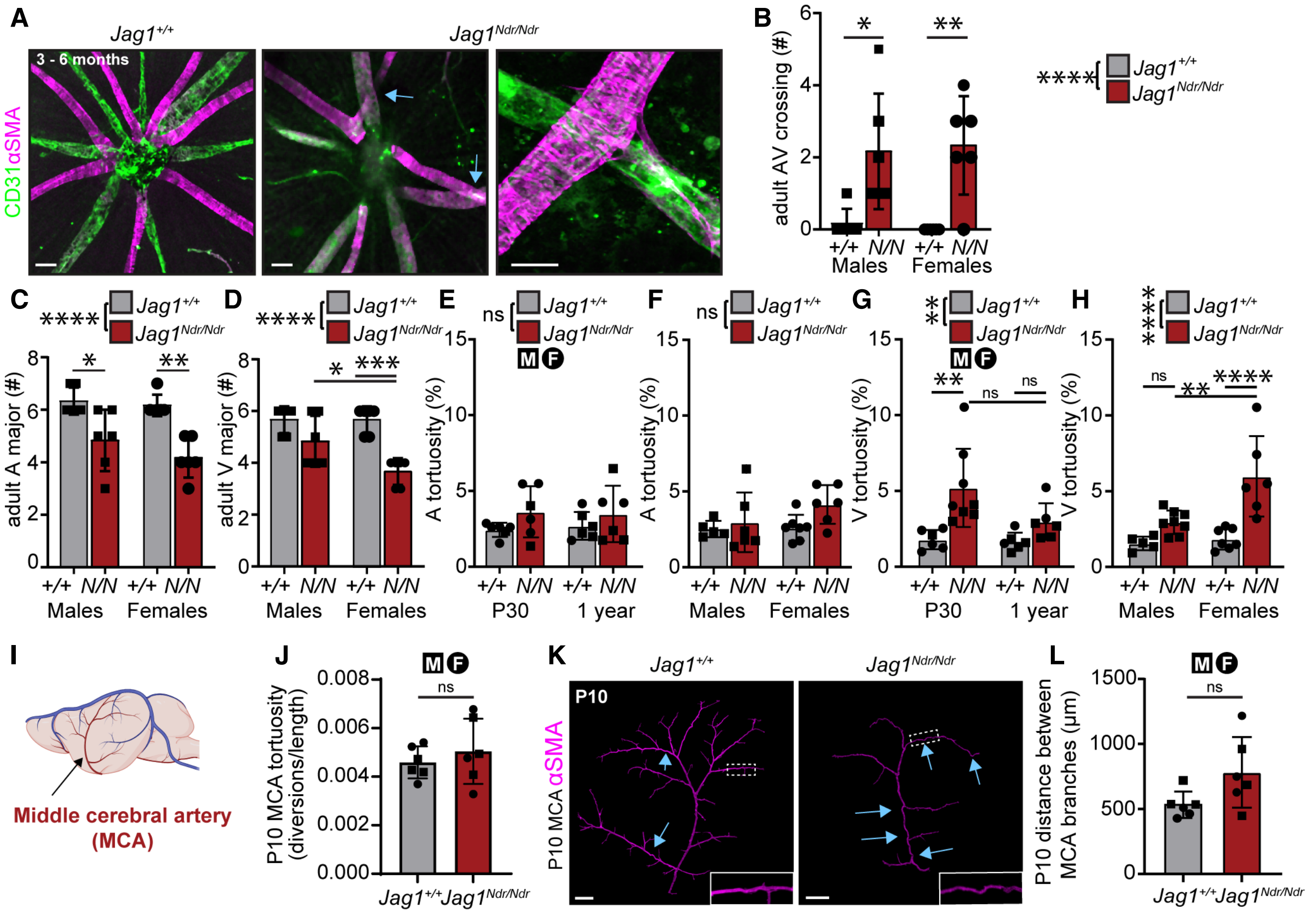

Figure 3. $Jag1^{Ndr/Ndr}$ mice display vascular guidance defects, with fewer and more tortuous blood vessels.

A, B (A) Radial arrangement of arterioles (magenta) and venules (green) from the optic nerve. Blue arrows label arteriovenous crossings. Magnification of crossing shows compression/narrowing of the underlying vein. Scale bar left and middle panels 50 µm, and right panel 20 µm. (B) Arteriovenous crossings number per retina ($n = 6$ per group, two-way ANOVA with Tukey's multiple comparisons test. Interaction $P = 0.7074$, Sex $P \geq 0.9999$, Genotype $^{****}P \leq 0.0001$, Tukey's multiple comparisons test: Male:$Jag1^{+/+}$ vs. Male:$Jag1^{Ndr/Ndr}$ $^{*}P = 0.0201$; Female:$Jag1^{+/+}$ vs. Female:$Jag1^{Ndr/Ndr}$ $^{**}P = 0.0061$).

C, D (C) Number of major arterioles ($n = 6$ per group, two-way ANOVA with Tukey's multiple comparisons test. Interaction $P = 0.4353$, Sex $P = 0.1995$, Genotype $^{****}P \leq 0.0001$, Tukey's multiple comparisons test: Male:$Jag1^{+/+}$ vs. Male:$Jag1^{Ndr/Ndr}$ $^{*}P = 0.0146$; Female:$Jag1^{+/+}$ vs. Female:$Jag1^{Ndr/Ndr}$ $^{**}P = 0.0011$) and (D) venules ($n = 6$ per group, two-way ANOVA with Tukey's multiple comparisons test. Interaction $P = 0.044$, Sex $P = 0.044$, Genotype $^{****}P \leq 0.0001$, Tukey's multiple comparisons test: Male: $Jag1^{Ndr/Ndr}$ vs. Female:$Jag1^{Ndr/Ndr}$ $^{*}P = 0.0302$; Female:$Jag1^{+/+}$ vs. Female:$Jag1^{Ndr/Ndr}$ $^{***}P = 0.0002$).

E, F (E) Arterial tortuosity at P30 and 1 year, irrespective of sex ($n = 6$ per group, two-way ANOVA, not significant). (F) Arterial tortuosity in male and female mice, irrespective of age ($n = 5$-7, two-way ANOVA, not significant).

G, H (G) Venous tortuosity at P30 and 1 year, irrespective of sex ($n = 6$-8 per group, two-way ANOVA with Tukey's multiple comparisons test. Interaction $P = 0.1166$, Age $P = 0.0787$, Genotype $^{**}P = 0.0013$. Tukey's multiple comparisons test: P30:$Jag1^{+/+}$ vs. P30:$Jag1^{Ndr/Ndr}$ $^{**}P = 0.004$; P30:$Jag1^{Ndr/Ndr}$ vs. 1 year:$Jag1^{Ndr/Ndr}$ $P = 0.0802$; 1 year:$Jag1^{+/+}$ vs. 1 year:$Jag1^{Ndr/Ndr}$ $P = 0.5066$). (H) Venous tortuosity in male and female mice, irrespective of age ($n = 5$-7, two-way ANOVA with Tukey's multiple comparisons test. Interaction $^{*}P = 0.0223$, Sex $^{**}P = 0.007$, Genotype $^{***}P \leq 0.0001$. Tukey's multiple comparisons test: Male:$Jag1^{+/+}$ vs. Male:$Jag1^{Ndr/Ndr}$ $P = 0.3247$, Male:$Jag1^{Ndr/Ndr}$ vs. Female:$Jag1^{Ndr/Ndr}$ $^{**}P = 0.0031$, Female:$Jag1^{+/+}$ vs. Female:$Jag1^{Ndr/Ndr}$ $^{****}P = 0.0001$).

I–L (I) Schematic of middle cerebral artery (MCA) in mouse brain (Image from Biorender). (J) Tortuosity of MCA, as assessed by number of diversions per length ($n = 6$, t-test ns). (K) Visualization of MCA with alpha smooth muscle cell actin (αSMA) showed stereotype vasculature in 6 of 6 $Jag1^{+/+}$ animals, but 2 of 6 $Jag1^{Ndr/Ndr}$ animals showed highly divergent MCA architecture (blue arrows). Scale bar 500 µm. (L) Distances between arterial branches in MCA at P10 (unpaired t-test, not significant).

Data information: Bar graphs depict mean values ± standard deviation, each dot represents one biological replicate. Circles represent females, squares represent males. For details/results of statistical analyses, please see source data. A, arteriole; AV, arteriovenous; αSMA, alpha smooth muscle actin; F, female; M, male; P(X), postnatal day X; V, venule.

Source data are available online for this figure.

$Jag1^{Ndr/Ndr}$ mice with Angiotensin II (AngII), a VSMC vasoconstrictor, for 2 weeks (experimental set up Fig 4M). The baseline mean blood pressure was significantly lower in $Jag1^{Ndr/Ndr}$ mice (Fig 4N). Hypertension is reported as systolic blood pressure above 140 mmHg (Mills *et al*, 2016; Fig 4O, dotted line). Due to low $Jag1^{Ndr/Ndr}$ blood pressure, we considered mice that responded with ≥ 20% increase in the systolic blood pressure as hypertensive (Fig 4O). To evaluate whether hypertension affected blood vessel

 © 2022 The Authors

permeability in $Jag1^{Ndr/Ndr}$ mice, we injected the mice with Evans blue. The amount of Evans blue extracted from internal organs and brain was similar in AngII-treated $Jag1^{CTRL}$ and $Jag1^{Ndr/Ndr}$ mice (Figs 4P and EV4N). However, macroscopic evaluation of the brain revealed that one of five AngII-treated $Jag1^{Ndr/Ndr}$ mice (a male) displayed Evans blue leakage outside of the intracranial vessels (Fig 4Q). Finally, we examined whether hypertension affected VSMCs. Arteriolar VSMCs in AngII-treated adult $Jag1^{Ndr/Ndr}$ retinas

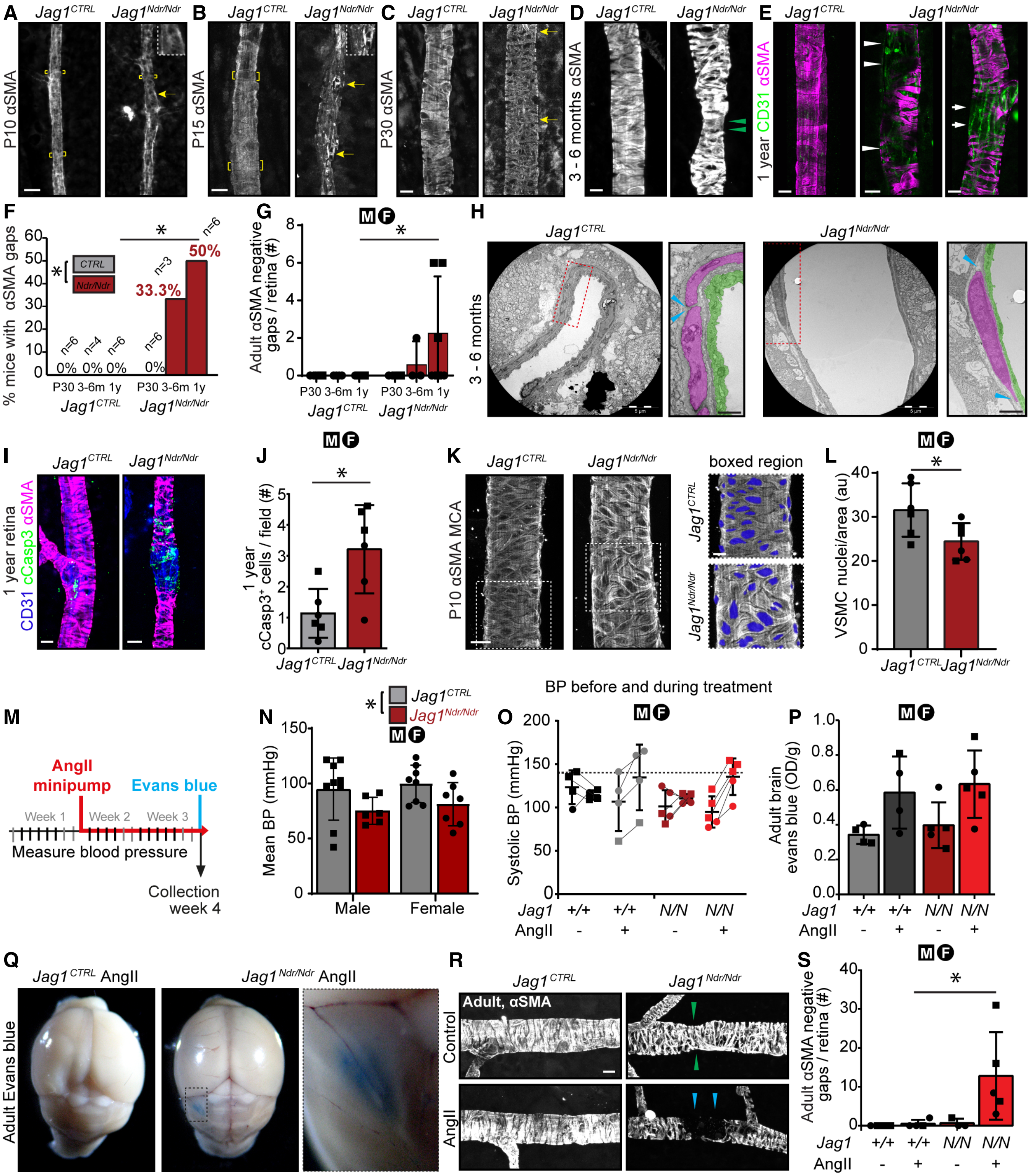

Figure 4.

◀ **Figure 4.** ***Jag1***$^{Ndr/Ndr}$ **mice display sparse arteriole vascular smooth muscle cell coverage that is exacerbated upon hypertension.**

A–E αSMA coverage of (A) P10, (B) P15, (C) P30, (D) 3–6-month-old and (E) 1-year-old retinal arterioles. Brackets in A, B denote perpendicular VSMCs around arteriole. Yellow arrows in A, B, C. indicate VSMCs with an orientation parallel to the blood vessel. Green arrowheads in D denote stenosis. White arrowheads in E label αSMA-negative gaps and white arrows an aneurysm. Cropped images were placed on black background for esthetic purposes. Scale bars 10 μm.

F, G (F) Incidence of α SMA negative gaps on arteries by age (Two-way ANOVA with Šídák's multiple comparisons test). (G) Number of α SMA negative gaps per retina and mouse by age (Two-way ANOVA with Šídák's multiple comparisons test. Dots represent biological replicates/individual retinas/mice. Two-way ANOVA not significant. Šídák's multiple comparisons test: 1 year *Jag1*$^{+/+}$ vs. 1 year *Jag1*$^{Ndr/Ndr}$ $^{*}P = 0.0189$).

H Transmission electron microscopy of retinal arterioles. VSMCs color-coded in magenta, EC in green. Scale bar 5 μm, boxed region 1 μm. Blue arrowheads denote the edges of VSMCs.

I, J (I) One-year-old retinal arteriolar cCasp3+ apoptotic cells. Scale bar 10 μm. (J) cCasp3+ cells per field ($n = 6$ per group, unpaired t-test, $^{*}P = 0.0109$).

K, L (K) Left panel aSMA coverage of MCA of P10 mice. Scale bar 20 μm. Right panel magnification of area where number of nuclei was counted. Nuclei were color-coded blue. Scale bar 10 μm. (L) MCA vascular smooth muscle cell nuclei quantification ($n = 6$ per group, unpaired t-test, $^{*}P = 0.0421$).

M–S (M) Experimental set up for AngII treatment. (N) Blood pressure (BP) before treatment in male and female *Jag1*^{CTRL} and *Jag1*$^{Ndr/Ndr}$ mice. Each dot represents one animal/one biological replicate. (Two-way ANOVA Interaction $P = 0.9407$, Sex $P = 0.4957$, Genotype $^{*}P = 0.0207$. Šídák's multiple comparisons test not significant). (O) Blood pressure increase in *Jag1*^{CTRL} and *Jag1*$^{Ndr/Ndr}$ mice treated with vehicle or AngII for 2 weeks (*Jag1*$^{+/+}$ ctrl $n = 4$, *Jag1*$^{+/+}$ AngII $n = 4$, *Jag1*$^{Ndr/Ndr}$ ctrl $n = 5$, *Jag1*$^{Ndr/Ndr}$ AngII $n = 5$, two-way ANOVA, with Šídák's multiple comparisons). (P) Detection of Evans blue leakage in brain in *Jag1*^{CTRL} and *Jag1*$^{Ndr/Ndr}$ mice with or without AngII treatment. (*Jag1*$^{+/+}$ ctrl $n = 4$, *Jag1*$^{+/+}$ AngII $n = 4$, *Jag1*$^{Ndr/Ndr}$ ctrl $n = 5$, *Jag1*$^{Ndr/Ndr}$ AngII $n = 5$, two-way ANOVA, with Šídák's multiple comparison. Interaction $P = 0.9687$, Treatment $^{**}P = 0.007$, Genotype $P = 0.5058$). (Q) One *Jag1*$^{Ndr/Ndr}$ mouse (male) displayed a macroscopic EB leakage, upon AngII treatment. (R) Retinal arteriolar αSMA coverage in vehicle or AngII-treated mice. Green arrowheads denote stenosis, blue arrowheads label αSMA-negative gap. Scale bar 10 μm. (S) αSMA-negative gaps in arterioles per retina (*Jag1*$^{+/+}$ ctrl $n = 4$, *Jag1*$^{+/+}$ AngII $n = 4$, *Jag1*$^{Ndr/Ndr}$ ctrl n = 3-5, *Jag1*$^{Ndr/Ndr}$ AngII $n = 5$, two-way ANOVA, with Šídák's multiple comparison. Interaction $P = 0.1054$, treatment $P = 0.0815$; Genotype $P = 0.0747$. Šíák's multiple comparisons test: AngII-treated *Jag1*$^{+/+}$ vs. AngII-treated *Jag1*$^{Ndr/Ndr}$ $^{*}P = 0.0315$).

Data information: AngII, AngiotensinII; αSMA, alpha smooth muscle actin; BP, blood pressure; cCasp3, cleaved caspase 3; EB, Evans blue; MCA, middle cerebral artery; OD, optical density, P(X), postnatal day X; VSMC, vascular smooth muscle cell. Bar graphs depict mean values ± standard deviation, each dot represents one biological replicate. Circles represent females, squares represent males. For details/results of statistical analyses, please see source data.

Source data are available online for this figure.

were absent in large patches (Fig 4R, blue arrowheads), resulting in a significant increase in αSMA-negative gaps at this relatively young age (Fig 4S). In summary, our data show that VSMC homeostasis is compromised in *Jag1*$^{Ndr/Ndr}$ mice, leading to gaps in VSMC coverage, which are exacerbated by aging and/or upon hypertension. Hypertension was associated with intracranial vessel leakage in one of five *Jag1*$^{Ndr/Ndr}$ mice.

Capillary homeostasis is compromised and retinal ganglion cells break down in *Jag1*$^{Ndr/Ndr}$ retinas

Blood vessel homeostasis is crucial for the integrity of the blood-brain/retina barrier, and tissue health, and could be a factor contributing to bleeding in ALGS. We studied all three retinal vascular plexuses in P30, 3–6 month (adult) and 12-month-old mice (Figs 5A and E, and EV5A). While the superficial capillary plexus (SCP) was equally branched in both *Jag1*^{CTRL} and *Jag1*$^{Ndr/Ndr}$ retinas across stages (Figs 5A, B, E, and F, and EV5A and B), the P30 and adult *Jag1*$^{Ndr/Ndr}$ intermediate capillary plexus (ICP) was less vascularized (Fig 5C and G) with significantly fewer branching points (Fig 5D and H), and the phenotype was more severe in adult mice than at P30 (e.g., at P30 the *Jag1*$^{Ndr/Ndr}$ ICP length is 74% of wild type, while at 3-6 months it is 66% of wild type). In 1-year-old mice the ICP was equally branched and vascularized (Fig EV5C and D) in *Jag1*^{CTRL} and *Jag1*$^{Ndr/Ndr}$ retinas. The vascularization of the P30 *Jag1*$^{Ndr/Ndr}$ retina was similar to adult *Jag1*^{CTRL}, while the vascularization of adult *Jag1*$^{Ndr/Ndr}$ retina was similar to the 1-year-old *Jag1*^{CTRL} retina with diminished branching and vascular density in ICP, suggesting accelerated aging in *Jag1*$^{Ndr/Ndr}$ retina. In order to determine whether the differences in ICP density were a result of capillary regression, we stained for Collagen IV (COLIV; Fig 5I) and measured the lengths of empty COLIV sleeves. Empty COLIV sleeves per area were increased in the ICP of two female *Jag1*$^{Ndr/Ndr}$ mice (Fig 5J). We further quantified the number of vertical sprouts between SCP and ICP and ICP and the deep capillary plexus (DCP) in adult (Fig 5K and L) and 1-year-old (Fig 5K and M) mice. There were significantly fewer ICP-DCP vertical sprouts adult *Jag1*$^{Ndr/Ndr}$ retina (Fig 5L). In contrast, the number of vertical sprouts was similar between animals in 1-year-old mice (Fig 5M). Nevertheless, vascular defects were aggravated in 1-year-old *Jag1*$^{Ndr/Ndr}$ retina as evidenced by a discontinuous ICP (Fig 5K, bottom panel, white arrowheads).

We next asked whether vascular defects in retina had an impact on neural tissue, since optic disc drusen has been reported in patients with ALGS (Hingorani *et al*, 1999; Narula *et al*, 2006). Retinal ganglion cells (RGCs; Sanes & Masland, 2015) transmit signals from photoreceptors to the brain via the optic nerve. Approximately 90% of patients with ALGS display optic nerve anomalies (El-Koofy *et al*, 2011). RGCs are sensitive to hypoxia (Kergoat *et al*, 2006), and can therefore be affected by the vascular defects identified here. To evaluate if the changes in retinal vasculature had neurological consequences, we investigated neurofilament-positive (NF+) RGC axons. The RGC axons appeared healthy in *Jag1*$^{Ndr/Ndr}$ retinas at P10 (Fig 5N and P). However, at P40 the RGC axons in *Jag1*$^{Ndr/Ndr}$ mice were reduced in number (Fig 5O) and covered 40% less area (Fig 5P). In sum, the *Jag1*$^{Ndr/Ndr}$ adult vasculature aged prematurely, marked by retracting retinal capillaries resulting in a reduced and discontinuous vascular network. The onset of vascular degeneration was associated with RGC degradation in *Jag1*$^{Ndr/Ndr}$ mice.

Non-invasive retinography reveals significant vascular defects in patients with Alagille syndrome

Abnormal retinal vasculature has been noted in patients with ALGS (Hingorani *et al*, 1999; Kim & Fulton, 2007; Fahnehjelm *et al*, 2011), but it has not been systematically analyzed nor compared to animal models, or patients with CADASIL. Since *Notch3*

 © 2022 The Authors

knockout mice (Henshall *et al*, 2015), and mice bearing CADASIL loss-of-function—NOTCH3^{C455R} (Machuca-Parra *et al*, 2017) or a signaling-competent CADASIL-NOTCH3^{R90C} (Ruchoux *et al*, 2003) display sparse VSMCs or gaps similar to that identified in *Jag1*$^{Ndr/Ndr}$ mice (Fig 4A–E), and mice bearing CADASIL-mutant NOTCH3^{R1031C} similarly exhibit VSMC thinning (Arboleda-

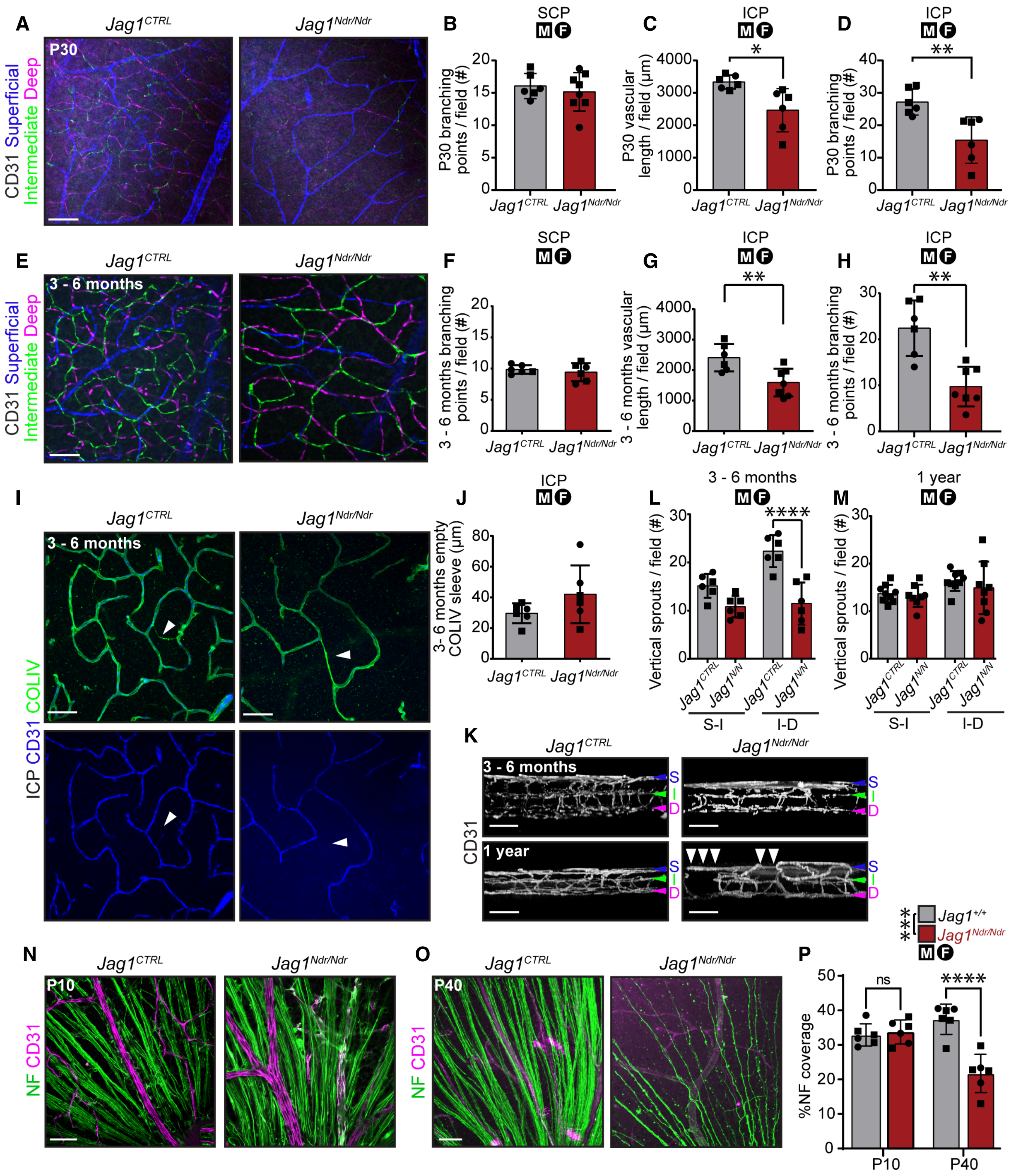

Figure 5.

Figure 5. Capillary homeostasis is compromised, and retinal ganglion cells break down in $Jag1^{Ndr/Ndr}$ retinas.

A–D (A) P30 retina, three CD31+ capillary layers pseudo-colored for superficial, intermediate and deep layers. Scale bar 50 μm. (B) P30 SCP branching point number (*n* = 6-8 per group, unpaired *t*-test, ns, *P* = 0.5346). (C) P30 ICP vascular length (*n* = 6 per group, unpaired *t*-test, **P* = 0.0124). (D) P30 ICP branching point number (*n* = 6 per group, unpaired *t*-test, ***P* = 0.0057).

E–H (E) 3–6-month-old retina, three pseudo-colored CD31+ capillary layers. Scale bar 50 μm. (F) Adult SCP branching point number (*n* = 6 per group, unpaired *t*-test, ns, *P* = 0.5365). (G) Adult ICP vascular length (*n* = 6-7 per group, unpaired *t*-test, ***P* = 0.0078). (H) Adult ICP branching point number (*n* = 6-7 per group, unpaired *t*-test, ***P* = 0.001).

I, J (I) ICP COLIV and CD31 (arrowhead labels COLIV+ CD31- capillary). Scale bar 50 μm. (J) Empty COLIV sleeve length in ICP per field (*n* = 6 per group, unpaired *t*-test, ns, *P* = 0.1583).

K–M (K) Retinal capillary plexuses in 3–6-month-old (top panels) and 1-year-old mice (bottom panels). White arrowheads label discontinuous ICP. Blue arrowhead labels SCP, green ICP, and magenta DCP. (L-M) Vertical sprout number between SCP (S) and ICP (I) and ICP (I) and DCP (D) in (L) adult (*n* = 6 per group, two-way ANOVA, genotype ****P* = 0.0004, Šídák's multiple comparisons test S-I ns, *P* = 0.0592, (I–D) *****P* < 0.0001) and (M) 1-year-old mice (*n* = 8 per group, two-way ANOVA, genotype ns, *P* = 0.4507, Šídák's multiple comparisons test S-I ns, *P* = 0.9483, (I–D) ns, *P* = 0.6046).

N–P (N) P10 and (O) P40 neurofilament (NF) and CD31 staining. Scale bar 50 μm. (P) % area positive for NF in P10 and P40 mice (*n* = 6 per group, two-way ANOVA: Interaction ****P* = 0.0001; Age **P* = 0.0428; Genotype ****P* = 0.0004. Šídák's multiple comparisons test: P10 $Jag1^{+/+}$ vs. P10 $Jag1^{Ndr/Ndr}$ *P* = 0.9246; P40 $Jag1^{+/+}$ vs. P40 $Jag1^{Ndr/Ndr}$ *****P* ≤ 0.0001).

Data information: COLIV, collagen IV; D or DCP, deep capillary plexus; I or ICP, intermediate capillary plexus; NF, neurofilament; S or SCP, superficial capillary plexus; P(X), postnatal day X. Bar graphs depict mean values ± standard deviation, each dot represents one biological replicate. Circles represent females, squares represent males. For details/results of statistical analyses, please see source data.
Source data are available online for this figure.

Velasquez *et al*, 2011), we considered it of interest to include CADASIL patients in this analysis.

We first analyzed retinal vasculature in a previously reported cohort of CADASIL patients (Alten *et al*, 2014; Nelis *et al*, 2018). There were no significant differences in the numbers of arteriovenous crossings (Fig 6A and B), major arterioles or venules (Fig 6C and D) nor in arterial or venous tortuosity (Fig 6E and F) in patients with CADASIL compared to age-matched control patients. Venous tortuosity was mildly increased in some patients with CADASIL, but the degree of tortuosity did not correlate with Fazekas score (a measure of white matter T2 hyperintense lesions, Appendix Fig S3A). While arterial tortuosity does not change with age in healthy humans (Appendix Fig S3B), venous tortuosity correlated with age in healthy humans (Appendix Fig S3C), but not in patients with CADASIL.

We analyzed retinal fundus photographs from pediatric patients with ALGS, biliary atresia (BA, as cholestatic controls), and age-matched healthy control patients (Fahnehjelm *et al*, 1999, 2011; Fig 6A). Similar to the $Jag1^{Ndr/Ndr}$ mice (Fig 3A and B), arteriovenous crossings were significantly increased in patients with ALGS compared to the control group (Fig 6G). The number of major arterioles and venules were significantly lower in patients with ALGS compared to controls, but similar to patients with BA (Fig 6H and I). Arteriole tortuosity was significantly increased in patients with ALGS compared to controls (Fig 6J), and venule tortuosity was significantly increased in patients with ALGS compared to both patients with BA and healthy controls (Fig 6K). As expected in this small cohort, no patients in this group experienced intracranial bleeds (Table EV1).

Altogether, our data demonstrate that patients with ALGS have vascular abnormalities that can be visualized and quantified non-invasively. Specifically, patients with ALGS have more arteriovenous crossings, fewer major arterioles and venules, and increased vascular tortuosity. Increased venous tortuosity is highly penetrant in patients with ALGS (identified in five of six patients). Importantly, $Jag1^{Ndr/Ndr}$ mice predicted these phenotypes with fewer major blood vessels, more arteriovenous crossings, and increased vascular tortuosity.

Discussion

In this study, we investigated risk factors that contribute to bleeds in patients with ALGS. Using a systematic review and $Jag1^{Ndr/Ndr}$ mice, we identified female sex, hypertension, reduced skull thickness, premature vascular aging, and increased venous tortuosity as risk factors negatively impacting JAG1-deficient vasculature. We show that $Jag1^{Ndr/Ndr}$ mice are a faithful model recapitulating vascular disease in patients with ALGS. Of clinical relevance, vascular anomalies identified in mice can be detected non-invasively in patients using retinography.

Our systematic review is the first report that methodically maps sex differences in ALGS vascular disease. We identified that spontaneous intracranial bleeding was reported more often in female than male patients. The majority of bleeds were subarachnoid hemorrhages (SAH) which, in the general population, are more common in men between ages 25 and 45, and in women between ages 55 and 85 (de Rooij *et al*, 2007). The greater risk of SAH in women over 55 has been attributed to hormonal differences after menopause, but hormone replacement therapy has yielded mixed results (Mhurchu *et al*, 2001; Qureshi *et al*, 2016). The patients summarized in the systematic review with SAH included one 13-year-old male and 10 females between 9 and 30 years old. Patients with ALGS may also present with hormonal differences which are as yet poorly characterized: puberty is delayed in some patients with ALGS, and some are non-responsive to growth hormone (Alagille *et al*, 1987; Bucuvalas *et al*, 1993). Whether sex differences in vascular pathology are a result of hormonal differences, and whether hormones are associated with the risk of SAH in this pediatric population, is thus worth further investigation.

This study shows that $Jag1^{Ndr/Ndr}$ mice exhibit a spectrum of vascular phenotypes that differ both in frequency (highly penetrant, sex-dependent, and sporadic) and severity. An example of a highly penetrant vascular phenotype is the aberrant arteriovenous crossings which present in 11 of 12 $Jag1^{Ndr/Ndr}$ mice (Fig 3B). Female $Jag1^{Ndr/Ndr}$ mice displayed more statistically significant vascular defects, including a greater reduction in veins and increased venous tortuosity (Fig 3D and H). But interestingly, we more often detected a high number of αSMA gaps (Fig 4G, J, and S) and apoptotic

 © 2022 The Authors

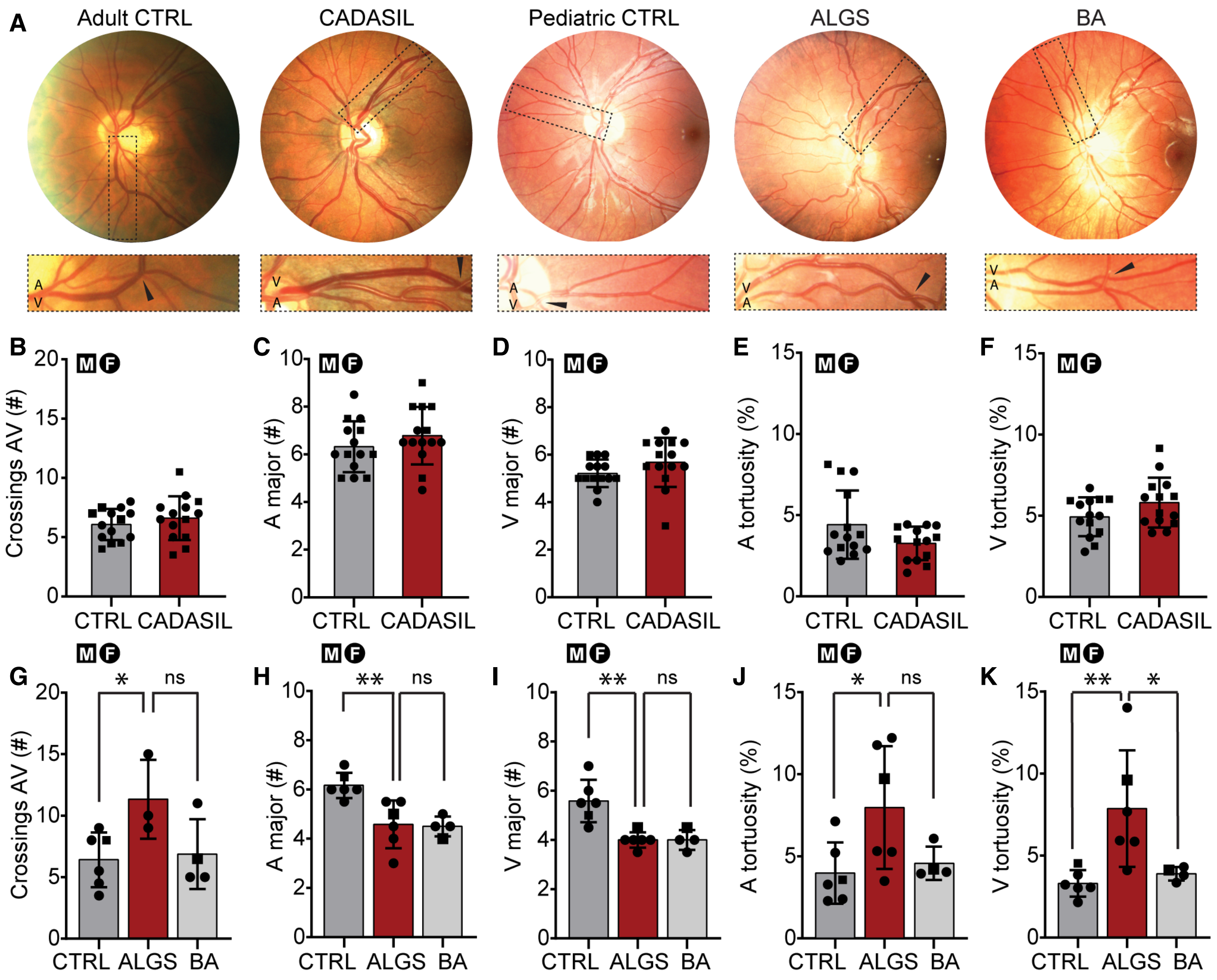

Figure 6. Non-invasive retinography reveals significant vascular defects in patients with Alagille syndrome.

A Representative retinographs from a healthy adult, patient with CADASIL, healthy pediatric individual, patient with ALGS, and patient with BA. Boxed regions and black arrowheads magnify arteriovenous crossing.

B–F (B) Arteriovenous crossings per retina ($n = 14$ per group, unpaired t-test, ns, $P = 0.3854$). (C) Major arterioles ($n = 14$ per group, unpaired t-test, ns, $P = 0.2902$) and (D) venules ($n = 14$ per group, unpaired t-test, ns, $P = 0.1536$). (E) Arterial ($n = 14$ per group, unpaired t-test, ns, $P = 0.0759$) and (F) venous tortuosity ($n = 14$ per group, unpaired t-test, ns, $P = 0.1050$).

G–K (G) Arteriovenous crossings per retina (CTRL $n = 5$, ALGS $n = 3$, BA $n = 4$, one-way ANOVA, ns, $P = 0.0608$, Šídák's multiple comparisons ALGS vs. CTRL *$P = 0.0492$, ALGS vs. BA ns, $P = 0.0999$). (H) Major arterioles (CTRL $n = 5$, ALGS $n = 5$, BA $n = 4$, one-way ANOVA, **$P = 0.0026$, Šídák's multiple comparisons ALGS vs. CTRL **$P = 0.0039$, ALGS vs. BA ns, $P = 0.9799$) and (I) venules (CTRL $n = 5$, ALGS $n = 5$, BA $n = 4$, one-way ANOVA, ***$P = 0.0008$, Šídák's multiple comparisons ALGS vs. CTRL **$P = 0.0011$, ALGS vs. BA ns, $P > 0.9999$). (J) Arterial (CTRL $n = 5$, ALGS $n = 5$, BA $n = 4$, one-way ANOVA, *$P = 0.0486$, Šídák's multiple comparisons ALGS vs. CTRL *$P = 0.0392$, ALGS vs. BA ns, $P = 0.1204$) and (K) venous tortuosity (CTRL $n = 5$, ALGS $n = 5$, BA $n = 4$, one-way ANOVA, **$P = 0.009$, Šídák's multiple comparisons ALGS vs. CTRL **$P = 0.0076$, ALGS vs. BA *$P = 0.0327$).

Data information: Bar graphs depict mean values ± standard deviation, each dot represents one biological replicate. Circles represent females, squares represent males. For details/results of statistical analyses, please see source data. A, arteriole; ALGS, Alagille syndrome; AV, arteriovenous; BA, biliary atresia; CADASIL, cerebral autosomal dominant arteriopathy with subcortical infarcts and leukoencephalopathy; CTRL, control; V, venule.
Source data are available online for this figure.

VSMCs in males. To facilitate an overview, all sex data are summarized in Appendix Table S2. Sporadic events included two $Jag1^{Ndr/Ndr}$ female mice with large provoked bleeds in liver (Fig 2J and K) and one macroscopic brain bleed in AngII-treated $Jag1^{Ndr/Ndr}$ male (Fig 4G). Analyzing vascular events is also complicated by the fact that the mice with the gravest vascular defects are likely to die earlier, and mice surviving to 1 year of age may be enriched for the healthiest $Jag1^{Ndr/Ndr}$ mice. There are thus both statistically significant highly penetrant and sex-specific differences in *Jag1*-abrogated vasculature, as well as sporadic events in both sexes. Which phenotype, or phenotype severity, correlates with bleeding events would be important to test in future studies.

© 2022 The Authors

Vascular health was compromised by hypertension in $Jag1^{Ndr/Ndr}$ mice. Two weeks of hypertension in $Jag1^{Ndr/Ndr}$ mice caused rapid degeneration of VSMCs, forming αSMA-negative gaps in the VSMC layer (Fig 4R and S). Hypertension, a well-established risk factor for subarachnoid hemorrhage (Ruigrok *et al*, 2001), is most prevalent in men in early adulthood, but is more common in women from middle age (Wang *et al*, 2022). However, $Jag1^{Ndr/Ndr}$ mice had lower blood pressure than controls, irrespective of sex. This could be due to the cardiac defects present in $Jag1^{Ndr/Ndr}$ mice which include atrial and ventricular septation defects (Andersson *et al*, 2018). Hypertension was reported in several patients with ALGS, often related to renal artery stenosis (Berard *et al*, 1998; Emerick *et al*, 2005; Santamaria *et al*, 2016) and associated with visceral artery aneurysm (Sanada *et al*, 2019). Hypertension could thus be a risk factor for patients with ALGS. Future studies should, therefore, aim to systematically determine whether VSMC compromise in patients with ALGS interacts with elevated blood pressure.

Notch signaling is crucial for arterial specification during embryogenesis and arterial VSMC maintenance throughout life (High *et al*, 2008; Henshall *et al*, 2015). The VSMC paucity and apoptotic VSMCs in the $Jag1^{Ndr/Ndr}$ mice are similar to that seen in *Notch3* mutant mice and CADASIL (Joutel *et al*, 1996; Henshall *et al*, 2015; Machuca-Parra *et al*, 2017). Although multiple consequences of *NOTCH3* mutations have been described in CADASIL (most often toxic gain of function; Xiromerisiou *et al*, 2020), it has also been proposed that reduced NOTCH3 signaling can contribute to the VSMC phenotype in CADASIL (and indeed, a CADASIL-like patient was reported with homozygous *NOTCH3* loss of function and paucity of VSMCs but no granular osmiophilic material (GOMs); Pippucci *et al*, 2015). Whether the CADASIL-like VSMC pathology in $Jag1^{Ndr/Ndr}$ mice entails a risk for CADASIL-like vascular dementia in patients with ALGS is an important future avenue of research. No GOM, a hallmark of CADASIL, was seen in the $Jag1^{Ndr/Ndr}$ mice. The retinal vascular abnormalities detected in ALGS retinographs were not present in CADASIL, suggesting vascular tortuosity (identified in both ALGS and the mouse model) is specific to ALGS. ALGS thus mimics some aspects of CADASIL, such as VSMC paucity, but also exhibits unique vascular tortuosity. In $Jag1^{Ndr/Ndr}$ mice, VSMC paucity was present in cardiac and neural arterioles in retina and brain (Figs 4C–I, K, and L, and EV4C). Weak VSMC coverage has been reported in a ruptured intracranial aneurysm in a patient with ALGS (Doberentz *et al*, 2015), and our data further corroborate that VSMC pathology may be of concern in ALGS. Further investigation of the similarities and differences between *Jag1* and *Notch3* compromised mice should help elucidate which phenotypes are regulated by Jag1-Notch3 mechanisms and could be amendable to similar therapeutic approaches.

Retinal vessel tortuosity, observed both in patients with ALGS and $Jag1^{Ndr/Ndr}$ mice, is an indicator of vessel wall dysfunction (Rim *et al*, 2020) and is associated with ischemic stroke (Ong *et al*, 2013; Hughes *et al*, 2016). Furthermore, retinal vascular changes are correlated with cerebral small vessel disease (Kwa *et al*, 2002) and with other abnormal vessels in the body (Kim & Fulton, 2007). Although we focused on bleeding events in this report, it is worth noting that ischemic events have also been reported in patients with ALGS (Appendix Fig S1B and Appendix Table S1). Hence non-invasive retinal vascular imaging could serve as a screening method to investigate vascular compromise in patients with ALGS. Retinographs from patients with BA and ALGS displayed reduction in the number of veins, while venous tortuosity was specific to patients with ALGS, indicating that this is not related to cholestasis. Retinal tortuosity in patients with ALGS could, therefore, be further investigated as a correlate for neurovascular dolichoectasia (Carpenter *et al*, 2018) or with tortuous vessels in lung (Dieulafoy lesions; Sheth *et al*, 2018; Lentz *et al*, 2016), which are linked to pulmonary hemorrhage. The inclusion of patients with other cholestatic disorders in this type of study will be important to determine which symptoms are related to cholestasis, and which are specific to ALGS.

Aging is a major risk factor contributing to the development of cardiovascular disease and is partly attributed to vessel wall changes leading to EC dysfunction (Seals *et al*, 2011) and loss of contractile VSMCs (Reagan *et al*, 2018). Retinal vascular health was severely affected in adult $Jag1^{Ndr/Ndr}$ mice as shown by decreased vascular density, sparse VSMC coverage, and degeneration of retinal ganglion cells. Reduced retinal vessel branching is also present in patients with ALGS (Hingorani *et al*, 1999) with fewer major arterioles and venules at the optic disc border. Premature vascular aging in patients with ALGS could also explain early-onset vascular bleeding. In the general population, ruptured intracranial aneurysms occur between 56 and 58 years of age (Wang *et al*, 2018), whereas the age range for intracranial hemorrhage in patients with ALGS was 2 months to 30 years of age (Table 1). In sum, aging is a well-established risk factor for many diseases and potentially accelerated vascular aging could be considered in long-term care of patients with ALGS.

There are several limitations to this report. One is the low number of patients, a major challenge in the study of rare disease, included in the systematic review (172 patients with ALGS and sex data, but only 10 females and 1 male with sporadic intracranial bleeding) and in the retinograph analysis (ALGS $n = 6$). Another limitation of the study is that we were unable to identify the exact origin and cause of spontaneous central nervous system bleedings or to test whether retinal vasculopathy correlates with bleeding events, in the limited numbers of mice with bleeds and tissue suitable for analysis. Of note, $Jag1^{Ndr/Ndr}$ mice that died between birth and P10 were often cannibalized by the mother and thus not possible to analyze for bleeding events or vasculopathy. Experiments aimed at specifically collecting large cohorts of mice with and without bleeds at matched stages would thus allow for testing correlations. Furthermore, future well-powered large-scale studies in humans, for example, via the Global Alagille Alliance (Vandriel *et al*, 2022; https://www.galastudy.com/), could assess whether sporadic intracranial bleeds are indeed over-represented among adult females with ALGS; and test whether patient retinography can serve as a biomarker for vascular health in patients.

Materials and Methods

Statistical analysis

Statistical analyses of differences between $Jag1^{CTRL}$ and $Jag1^{Ndr/Ndr}$ or $Jag1^{\Delta Dsl/+}$ animals was evaluated using two-sided unequal variance *t*-test or Mann–Whitney test. When more than two conditions were compared, a one-way ANOVA was used, and when two grouping variables were being analyzed (e.g., genotype and another

© 2022 The Authors

variable), a two-way ANOVA combined with multiple comparisons test was used, as appropriate and as described in figure legends and Source data. Pearson correlation was used for correlation analysis. For analysis of the systematic review, two-sided binominal exact test was used to test whether the observed proportions of male and female patients, reported with given vascular or bleeding events, was significantly different from the expected proportions (1:1). *P*-value was considered statistically significant if $P < 0.05$ (*$P < 0.05$, **$P < 0.01$, ***$P < 0.0001$). Specific *P*-values are listed in figure legends and Source data. Sample size was calculated using the Resource equation method.

Study approval

Animal experiments were performed in accordance with local rules and regulations and were approved by Stockholms Norra Djurförsöksetiska nämnd (Stockholm animal research ethics board, ethics approval numbers: N50/14, N61/16, N5253/19, N2987/20, N14960/20). All procedures conform to the guidelines from Directive 2010/63/EU of the European Parliament. $Jag1^{\Delta Dsl/+}$ mouse experiments were performed in accordance with local rules and regulations and were approved by Charles River Laboratories institutional animal care and use committee (IACUC). All actions conform to the federal regulations for care of laboratory animals.

Data collection from pediatric patients and analysis was performed under the 335/00, and 2019-00202 ethical permits approved by Regional ethics review board in Stockholm. The patients included in this study and/or their guardians gave written informed consent, and were previously reported in part (Fahnehjelm *et al*, 1999, 2011). The medical history from patients with ALGS or BA, whose retina fundus photographs were analyzed here, follows the decision 2017/1394-31 by the Regional ethics review board in Stockholm. Permission was given to retrospectively collect data from charts of patients with chronic cholestatic disease without additional consent from patients. All investigation conforms to the principles outlined in the WMA Declaration of Helsinki and the Department of Health and Human Services Belmont Report.

The data collection and analysis from patients with CADASIL and adult control patients were approved by the IRB of the Ärztekammer Westfalen-Lippe and University of Münster (2015-402-f-S). All subjects gave written informed consent.

Mouse maintenance and breeding

The Nodder mice ($Jag1^{+/Ndr}$ colony C3H background EM strain #13207, wild type C57bl6 JAX strain #000664) were maintained in a mixed C3H/C57bl6 genetic background as reported previously (Andersson *et al*, 2018). Nodder mice were genotyped for the *Ndr* allele and sex by Transnetyx® (USA). Mice were housed in cages with enrichment and maintained on a standard day-night (12 h) cycle, with ad libitum access to food (standard chow SDS RM3 or SDS CRM, Special Diet Services) and water. Experiments generally include mice of both male and female sex. Experiments in which the results suggested sex differences were expanded with additional mice of each sex to determine whether sex differences were present. $Jag1^{+/+}$ and $Jag1^{Ndr/+}$ mice display wild type vasculature, and $Jag1^{Ndr/+}$ mice have not displayed spontaneous bleeds, and are thus both used as controls ($Jag1^{CTRL}$). The use of each genotype is specified in each section below. For organ collection, mice between P0 – P10 were sacrificed by decapitation and mice > P10 by CO_2 inhalation.

The $Jag1^{\Delta Dsl/+}$ mice (JAX strain #010616) on C57bl6/129S1 background were backcrossed to C57bl6 genetic background. SNP analysis confirmed 99.7% C57bl6 background. $Jag1^{\Delta Dsl/+}$ mice were genotyped for both alleles by Charles River Laboratories. Mice were group housed in cages, maintained on 14:10 light cycle, with ad libitum access to food (standard chow 5L79, specifically formulated by Lab Diet for Charles River) and water.

Patient samples

Color photographs of the ocular fundi of patients with ALGS, BA and their age-matched controls were obtained after pupil dilatation using fundus cameras Canon EOS-1 Kodak Professional DCS 520C (Canon, Rochester, New York, USA) or Canon CRDG non-mydriatic retinal camera (Canon, Tokyo, Japan). Only correctly focused photographs were used for the analysis. Seven healthy pediatric control patients (median age 8 years, range 7–8 years; 1 male, 6 females), six patients with ALGS (median age 7.5 years, range 2–11 years; 1 male, 5 females), four patients with BA (median age 10.5 years, range 8–16 years; 1 male, 3 females) were included. Color fundus photography of patients with CADASIL and their aged-matched controls was performed using Visucam (Carl Zeiss Meditech, Germany). Data from these patients have been published in part in previous studies (Alten *et al*, 2014; Nelis *et al*, 2018). Fourteen healthy adult control patients (median age 48 years old, range 23–61 years; 5 males, 9 females) and 14 patients with CADASIL (median age 51 year, range 27–59 years; 6 males, 8 females) were included.

Blinding and randomization statement

The experimenter was not blinded when selecting and sacrificing animals for an experiment. No randomization procedure was used to allocate animals into groups. The experimenter was blinded when processing samples for analysis, during data acquisition and data analysis.

Immunofluorescence staining of retina

Eyes were fixed with 3.8% Formaldehyde solution (Sigma-Aldrich, cat. #F1635) overnight and whole retinas were dissected out, blocked and permeabilized in phosphate-buffered saline (PBS) containing 1% Bovine serum albumin (Sigma-Aldrich, cat. # A2153) with 0.3% TritonX-100 (Sigma-Aldrich, cat. #T8787). Primary and secondary antibodies were diluted in blocking solution. PBS: blocking solution (1:1) was used as a washing buffer. Each step was performed at 4°C, overnight. Retinas were flat-mounted in Vectashield (cat. no. H-1000, Vector Laboratories). Primary antibodies used: rat anti-mouse CD31 (RRID: AB_394816, cat. #553370, BD Biosciences, 1:100), rabbit anti-ERG (RRID: AB_2630401, cat. #ab92513, Abcam, 1:200), goat anti-DLL4 (RRID: AB_354770, cat. #AF1389, RnD systems, dilution 1:2,000), goat anti-CD13 (RRID: AB_2227288, cat. #AF2335, RnD Systems, 1:200), goat anti-Jagged1 (RRID: AB_260348, cat. #J4127, Sigma-Aldrich, 1:500), mouse anti-human α-smooth muscle actin (Cy3 conjugated, RRID: AB_476856, cat. #C6198, Sigma Aldrich, 1:500; FITC conjugated, RRID: AB_476977, cat. #F3777, Sigma Aldrich, 1:500), rabbit anti-Myosin heavy chain

© 2022 The Authors

11 (RRID: AB_2147146, cat. #ab53219, Abcam, 1:200), goat anti-Collagen I (RRID: AB_2753206, cat. #1310-01, Southern Biotech, 1:200), mouse anti-vimentin (RRID: AB_628437, cat. #sc6360, Santa Cruz, 1:200), rabbit anti-Transgelin (RRID: AB_443021, cat. #ab14106, Abcam, 1:200), rabbit anti-HISTONE H3 (phospho S10; RRID: AB_304763, cat. #ab5176, Abcam, 1:500), mouse anti-GFAP (Cy3 conjugated, RRID: AB_11213580, cat. # MAB3402C3, Millipore, 1:500), rabbit anti-Cleaved CASPASE3 (RRID: AB_2341188, cat. #9661 (D175), Cell Signaling, 1:700), rabbit anti-COLLAGEN type IV (RRID: AB_2276457, cat. # AB756P, Millipore, 1:500), mouse anti-Neurofilament (RRID: AB_528399, cat. #RT97, DSHB, 1:200) nuclei were labeled with DAPI (cat. #D9542, Sigma Aldrich, 1:1,000). Images were captured using LSM 510 META, LSM 880, LSM980 (Carl Zeiss AG) microscopes and processed in Image J (NIH), and/or Adobe image suite software (Adobe Inc). Any image modifications were applied identically to images being compared.

Vascular outgrowth analysis

The distance from the optic nerve to the periphery of a retina was measured in ImageJ (NIH) using the straight line tool. Distance was measured on tile-scanned images (10x or 20x objective) of whole retina starting from the optic nerve. Six measurements, regularly radially spaced at approximately 60-degree intervals, were performed per retina from 6 $Jag1^{+/+}$ and 6 $Jag1^{Ndr/Ndr}$ animals at P5.

Tip cells and filopodia analysis

Tip cells were defined as $CD31^{+}ERG^{+}$ cells with extended filopodia at the vascular front. The number of tips per tip cell was quantified as the number of tips with filopodia bundles (single membrane protrusions coming out of a tip cell) extending in a single direction, divided by the number of tip cells. Filopodia was defined as single hair-like membrane protrusions extending from a tip cell. All quantifications were counted manually in Image J, in 40× images, 4 images per animal, in 6 $Jag1^{+/+}$ and 6 $Jag1^{Ndr/Ndr}$ mice at P5.

Quantification of vascular density, branching point, and number of ERG+ endothelial cells

The number of branching points per field (40× objective) was manually quantified in the middle of the outgrowing vascular plexus (at P5, P10, P15 halfway between an arteriole and venule). The quantification of branching points was performed in 3–6 images per animal, in 6 $Jag1^{+/+}$ and 6 $Jag1^{Ndr/Ndr}$ mice at P5 and P15, 4 $Jag1^{+/+}$, 2 $Jag1^{Ndr/+}$ and 6 $Jag1^{Ndr/Ndr}$ mice at P10. In each 40x field the number of ERG^{+} nuclei were manually quantified in 3–5 images per animal, in 6 $Jag1^{+/+}$ and 6 $Jag1^{Ndr/Ndr}$ mice at P5 and P15, 4 $Jag1^{+/+}$, 2 $Jag1^{Ndr/+}$, and 6 $Jag1^{Ndr/Ndr}$ mice at P10. In the same field, the CD31+ vascular length was manually measured using the segmented line tool in ImageJ. The number of ERG+ endothelial cells was divided by vascular length.

Phosphorylated Histone3 proliferation analysis

Whole retina tile-scans (objective 10x) were taken from 4 $Jag1^{+/+}$ and 4 $Jag1^{Ndr/Ndr}$ mice at P5. Phospho-Histone H3+ (PH3+) CD31+ cells were manually counted in a field within a 45° wedge originating at the optic nerve, in zones of radius 100 μm, in 12 zones altogether. The average number of PH3+ CD31+ per zone was normalized to the zone area (μm^2) and multiplied by 100 (per 100 μm^2 area).

Cleaved Caspase 3

The number of cleaved caspase3 (cl. CASP3)-positive cells was quantified manually along arterioles in aged mice (> 1 year old) in 7–13 stacks per mouse, in 5 $Jag1^{+/+}$, 1 $Jag1^{Ndr/+}$ and 6 $Jag1^{Ndr/Ndr}$ mice. The arterioles were imaged in confocal z-stacks and inspected for cl. CASP3+ cells throughout the stacks. The number of first-generation arteriole branching points per image (field), the number of cl. CASP3+ cells at a branching point and the number of cl. CASP3+ outside of a branching point were manually quantified.

Arterial and venous segregation

In murine retinas, the number of major arterioles and venules originating at the optic nerve, and the number of arteriovenous crossings, were quantified in whole mount retinas stained for CD31 and αSMA. 6 male and 6 female $Jag1^{+/+}$ and 6 male and 6 female $Jag1^{Ndr/Ndr}$ adults and 6 $Jag1^{+/+}$ and 6 $Jag1^{\Delta Dsl/+}$ 1-year-old mice were included for quantification of arterioles and venules. Arteriole/arteriole and arteriole/venule crossings were counted and grouped together. No venule/venule crossings were observed. Altogether 6 male and 6 female $Jag1^{+/+}$ mice and 6 male and 6 female $Jag1^{Ndr/Ndr}$ mice were included for quantification of aberrant crossings in adult mice (3–6 months).

In human retinas, the number of major arterioles and venules were quantified at the border of the optic nerve head from fundus photographs in which the optic nerve head was the center of the image. The venules were recognized by their darker color. The number of arteriovenous crossings was counted. No distinction was made between arteriovenous crossing and nicking. Patients with ALGS n = 3, BA n = 4, pediatric control n = 6, CADASIL n = 14, adult control n = 14.

iDISCO+ immunostaining of mouse brain

iDISCO+ immunostaining and clearing of mouse brain samples were performed as described earlier (Renier *et al*, 2016). Briefly, mice were anesthetized with an overdose of isoflurane and intracardially perfused with PBS (pH 7.4; Ambion). Brains were postfixed in modified Zamboni fixative (4% paraformaldehyde and 0.2% picric acid, diluted in phosphate buffer, pH 7.4) at 4°C for 72 h and stored in PBS, containing 0.1% sodium azide, pending analysis. After being washed in PBS three times and dehydrated in rinsing methanol/water series (20%–40%–60%–80%–100%–100%), 1 h each, the samples were bleached with 5% hydrogen peroxide in 100% methanol overnight at 4°C. They were then rehydrated in downgrading series of methanol/water (80%–60%–40%–20%-PBS), incubated in permeabilization solution (0.2% Triton X-100/20% dimethyl sulfoxide (DMSO)/0.3 M glycine in 0.01 M PBS + 0.02% sodium azide) for 3 days and then in blocking solution (0.2%Triton X-100/10% DMSO/6% normal donkey serum in 0.01 M PBS + 0.02% sodium azide) for 2 days, both at 37°C. The samples were incubated with primary antibodies for alpha smooth muscle actin (αSMA; Cy3 conjugated, cat. #C6198, Sigma Aldrich, 1:500) and glucose transporter

 © 2022 The Authors

1 (GLUT1; cat. #2956779, Millipore, 1:500) for 5 days at 37°C [antibody diluent: 0.2% Tween-20/heparin (10 mg/ml)/5% DMSO/3% normal donkey serum in PBS + 0.02% sodium azide]. After overnight washing, the brains were incubated in secondary antibodies (Donkey anti-Rabbit IgG – Alexa Fluor 647; cat. #A31571, Thermo Fisher, 1:500) for 6 days at 37°C [diluent: 0.2% Tween-20/heparin (10 mg/ml)/3% normal donkey serum in PBS + 0.02% sodium azide]. Finally, the brains were cleared after dehydration in rinsing methanol/water series (see above), by incubation in 66% dichloromethane/33% methanol for 3 h and twice in 100% dichloromethane for 30 min. The cleared brains were transferred to 100% dibenzyl ether for long-term storage.

Light sheet fluorescence microscopy and 3D image reconstruction

Cleared brains were imaged with a light sheet microscope (Ultramicroscope II, Lavision Biotec, Bielefeld, Germany) and the ImSpector 347 software. All images were acquired using a 2x objective lens with additional 0.63x zoom body, an NA of 0.6 (XLPLN10XSVMP, Olympus), and a sCMOS camera (Hamamatsu ORCA-Flash4.0). Excitation laser lines of 561 and 647 nm were used. The light-sheet microscopy images were recorded with an xy resolution of 3.78 μm and a z-step size of 3.4 μm.

The serials of 16-bit uncompressed tif images were converted to an IMS file using the Imaris File Converter (Bitplane, UK), and the 3D vision of acquisitions was reconstructed using Imaris 9.9.0 software (Bitplane, UK). Quantifications and measurements such as distance between main branching points were obtained using semi-automatic filament tracing (autopath) on original images in Imaris. For visualization of the MCA, surfaces were applied to remove signal outside of the MCA and snapshot images (1,200 dpi) were taken from the actual light sheet scans (Imaris).

Vessel tortuosity

Retinal blood vessel tortuosity was quantified by manually tracing the curved length of major arterioles and venules from the optic nerve toward the periphery in murine whole retina (20x objective) or human retina fundus photographs, in Adobe Illustrator or ImageJ. In murine retinas, only the main branches were traced for analysis. In human retinas, only branches that extended to the periphery (edge of the image) were included for analysis. The chord length was measured as a straight line connecting the start point to the end point of a vessel. Tortuosity was calculated by dividing the curved length by the chord length and expressed as a percentage increase over 100% (100% = straight). The analysis was performed on whole retinas from one eye from 6 *Jag1*$^{+/+}$ and 8 (6 for arterioles) *Jag1*$^{Ndr/Ndr}$ P30 mice, 6 *Jag1*$^{+/+}$ and 6 *Jag1*$^{Ndr/Ndr}$ 1-year-old mice and all the patients included in the study (both eyes).

MCA tortuosity was quantified by the number of MCA diversion points, normalized to its length. The length of the main MCA vessel was obtained by creating a filament with the semi-automatic autopath function in Imaris on the original images. Diversions of the expected path of the curved vessel were identified as diversions from the expected continuous path of a vessel, and manually counted. The analysis was performed in 6 *Jag1*$^{+/+}$ and 6 *Jag1*$^{Ndr/Ndr}$ P10 mice.

Vertical sprouting analysis

To address the overall number of sprouts connecting SCP to ICP, and DCP to ICP, a single image from a z-stack halfway between SCP to ICP and DCP to ICP was analyzed. A sprout in this image appears as a single dot. One to three Z-stack images (40× objective) taken in the middle of a retina between arteriole and venule was analyzed per animal. Only samples in which CD31 antibody penetrated to all three vascular layers were included in the analysis. 6 *Jag1*$^{+/+}$ and 6 *Jag1*$^{Ndr/Ndr}$ adult mice (3–6 months) and 9 *Jag1*$^{+/+}$ and 8 *Jag1*$^{Ndr/Ndr}$ aged animals (1 year) were included.

Superficial and intermediate capillary plexus integrity

The number of SCP and ICP branching points and ICP vascular length was quantified in z-projected images (ImageJ) of the SCP or ICP (40x objective) taken in the middle of the retina between an arteriole and a venule. Only samples in which CD31 antibody penetrated to all three vascular layers were included in the analysis. Three to four images from 6 *Jag1*$^{+/+}$ and 6 *Jag1*$^{Ndr/Ndr}$ P30, 4 images from 6 *Jag1*$^{+/+}$ and 7 *Jag1*$^{Ndr/Ndr}$ adult animals, and two to five images from 6 *Jag1*$^{+/+}$ 6 *Jag1*$^{Ndr/Ndr}$ 1-year-old animals were analyzed. In the same field, the CD31+ vascular length was measured by segmented line tool in ImageJ. In the intermediate capillary plexus in 6 *Jag1*$^{+/+}$ and 6 *Jag1*$^{Ndr/Ndr}$ adult animals (3–6 months), the length of empty CollagenIV sleeves was measured using the segmented line tool in ImageJ.

Neurofilament quantification

Neurofilament coverage was quantified in ImageJ and calculated as % of area covered by neurofilaments of the total area (100%). Neurofilament area was measured using the following script in ImageJ:

```
run("Measure");
run("8-bit");
run("Median...", "radius = 2");
setAutoThreshold("Mean dark");
//run("Threshold...");
//setThreshold(6, 255);
setOption("BlackBackground", false);
run("Convert to Mask");
run("Watershed");
run("Analyze Particles...");
Total area was measured with "Measure".
```

Only samples in which red blood cell autofluorescence did not significantly interfere with fluorescent intensity quantification were included in the analysis. The measurement was performed on 3 to 6 images from 6 *Jag1*$^{+/+}$ and 6 *Jag1*$^{Ndr/Ndr}$ P10 and 2 to 4 images from 6 *Jag1*$^{+/+}$ and 6 *Jag1*$^{Ndr/Ndr}$ P40 animals.

Fluorescence intensity

For GFAP and αSMA intensities, the blood vessel of interest was outlined using the lasso tool. The intensity was assessed by the histogram function for individual channels in Adobe Photoshop. GFAP intensity was quantified in adults from 3 images from 4 *Jag1*$^{+/+}$ and

© 2022 The Authors

4 $Jag1^{Ndr/Ndr}$ mice. GFAP and αSMA intensities were quantified in 5 to 6 images from 6 $Jag1^{+/+}$ and $Jag1^{\Delta Dsl/+}$ 1-year-old mice.

For synthetic VSMC analysis, αSMA, SM22, MYH11, VIM, COLI intensities were quantified in ImageJ following this script:

```
run("Duplicate...", "title=MAX_1977-1wtp5vegfr340x1-1.lsm duplicate channels=1");
run("Median...", "radius = 5");
setAutoThreshold("Huang dark");
//run("Threshold...");
setOption("BlackBackground", false);
run("Convert to Mask");
run("Watershed");
//run("Threshold...");
run("Analyze Particles...", "size=200-Infinity pixel add");
roiManager("Show All with labels");
roiManager("Show All");
run("Set Measurements...", "area mean display redirect=None decimal=3");
close();
setSlice(2);
roiManager("Measure");
setSlice(3);
roiManager("Measure");
setSlice(1);
roiManager("Measure");
```

The region of interest for each channel was based on αSMA+ cells. The αSMA- areas were manually outlined and the fluorescence intensity was measured for all channels. The fluorescent intensity of αSMA- areas for each channel was normalized to the average intensity of αSMA+ cells (100% intensity) per image. Only samples in which red blood cells autofluorescence did not significantly interfered with fluorescent intensity quantification were included in the analysis. The quantification was performed in 4 images from 5 $Jag1^{Ndr/Ndr}$ (αSMA, SM22, VIM) and 6 $Jag1^{Ndr/Ndr}$ (αSMA, MYH11, COLI) 3-month-old to 1-year-old mice.

ASMA coverage

The number of gaps in αSMA retinal arteriole coverage was quantified manually. An αSMA gap was considered a gap between two VSMCs that was greater than one cell width (~ > 10 μm). The total number of gaps per retina (from one eye) was counted. The number of gaps was quantified in 6 $Jag1^{+/+}$and 6 $Jag1^{Ndr/Ndr}$ mice at P30 and 1-year-old and 3 $Jag1^{+/+}$ and 3 $Jag1^{Ndr/Ndr}$ 3–6-month-old mice.

In Angiotensin II experiment, the number of gaps was quantified in 4 $Jag1^{+/+}$, 3 $Jag1^{Ndr/Ndr}$, 4 $Jag1^{+/+}$ treated with Angiotensin II and 5 $Jag1^{Ndr/Ndr}$ mice treated with Angiotensin II.

The MCA αSMA coverage was assessed in 6 $Jag1^{+/+}$ and 6 $Jag1^{Ndr/Ndr}$ P10 mice by counting the αSMA nuclei. An αSMA nucleus was determined by lack of αSMA staining in the nucleus region. The number of αSMA nuclei was counted in three 100-pixel long regions per one MCA image per animal.

Image processing

Capillary plexus visualization was accomplished by splitting z-stack images into 3 equally sized z-stacks per animal, color coding each plexus and merging the 3 images. Due to different sizes of $Jag1^{+/+}$ and $Jag1^{Ndr/Ndr}$ retinas, stack sizes were different among animals. Some images (Figs 3N and 4A, and 6E; Appendix Fig S3A) were further processed in ImageJ by median filter (radius = 1). Retina whole vasculature side images were further processed in Volocity in which high opacity and noise reduction filters were included. To remove unspecific signal (always the pair of CTRL-NDR) some images were processed in ImageJ by median filter (radius 0.5–2), see Source data for comparison.

Liver resin casting

Adult liver portal vein vasculature was injected with synthetic resin MICROFIL® and scanned with micro computed tomography as previously described (Hankeova *et al*, 2021).

Micro-CT liver rupture analysis

Following the micro-CT measurements, the reconstructed data was imported and analyzed in VG Studio MAX software (Volume Graphics GmbH, https://www.volumegraphics.com). The Microfil-filled structures were separated from the background by global thresholding creating a main region of interest (ROI), then the areas of Microfil leaked from ruptured vessels were manually selected into separate ROIs. The volume of each ROI of leaked Microfil was calculated by the software. The analysis was performed in 4 $Jag1^{+/+}$, 2 $Jag1^{+/Ndr}$ and 6 $Jag1^{Ndr/Ndr}$ samples (analyses of portal vein and biliary architecture, but not vascular leakage data, for 3 $Jag1^{+/+}$ and 3 $Jag1^{Ndr/Ndr}$ samples of these casts were previously published in Hankeova *et al* (2021)).

Skull micro-CT

Micro-CT analysis was performed on 23 P30 skulls, 12 $Jag1^{+/+}$ and 11 $Jag1^{Ndr/Ndr}$ mice using a GE Phoenix v|tome|x L 240 (GE Sensing & Inspection Technologies GmbH, Germany) micro-CT scanner. The system is equipped with a 180 kV/15 W maximum power nanofocus X-ray tube and a flat panel detector with 4,000 × 4,000 pixel count and a pixel size of 100 × 100 μm. Acquisition parameters: voltage 80 kV, current 210 μA, integration time of 500 ms and 12.5 μm voxel size.

Skull thickness, length, and volume analysis

The skull thickness and volume analyses were conducted in VG Studio MAX 3.4 (Volume Graphics GmbH, Germany). A sample was registered within the coordinate system, and the skull surface was determined. Mandibula, all teeth, and remaining vertebrae were segmented out to maintain only skull. The length of the skull was measured from the occipital bone to the nasal bone from a dorsal view. The volume was calculated by multiplying the number of voxels determined from the skull surface by the volume of one voxel. The skull thickness was measured by utilizing a ray, searching for the opposite surface for each point on the skull surface. The length of the ray between surfaces expresses the actual skull thickness, and rays' lengths are depicted as a color-coded thickness map on each skull. The thickness of the skulls was measured in two different ways, first the full thickness of the skull and second taking into

© 2022 The Authors

account the trabeculation of the spongy bone. The spongy bone segmentation was conducted using a custom-written MATLAB pipeline (deposited in GitHub, R2017a, The MathWorks Inc, Natick, MA). The extracted spongy bone was fused with the extracted compact bone to create a full-thickness bone, which was consequently analyzed in the same way in the VG studio. Subsequently, the right temporal bone was manually segmented, with emphasis on the removal of overlapping surrounding bones. Temporal bone thickness was measured by the identical methodology as the whole skull. The analysis was performed in 6 *Jag1*$^{+/+}$ females, 6 *Jag1*$^{+/+}$ males, 6 *Jag1*$^{Ndr/Ndr}$ females and 5 *Jag1*$^{Ndr/Ndr}$ males.

Vessel permeability assay

Mice (3–6 months old) were injected with 200 µl 0.5% Evans blue (cat. #E2153, Sigma-Aldrich) via the tail vein. The dye was allowed to circulate for 17 h. Mice were anesthetized by CO_2 inhalation. Mice were either transcardially perfused with Hanks buffered salt solution (HBSS), at a perfusion rate of 5 ml/min for 3 min, or organs were immediately collected. Internal organs were dissected out, weighed, and placed in matched volumes of Formamide solution (cat. # 15515-026, Invitrogen) for 17 h at 56°C. Formamide solution containing Evans blue extracted from the organs was measured for absorbance at 610 nm in a VersaMax™ microplate reader (Molecular Devices Versa Max microplate reader). The amount of Evans blue measured was divided by the organ weight, and data were normalized to control conditions for either perfused or unperfused conditions, as appropriate. Brain vasculature was inspected under the stereomicroscope Stemi 305 (Carl Zeiss Microscopy) and photographed with a camera (Cannon, PowerShot S3 IS). The analysis is based on 5 *Jag1*$^{+/+}$, 2 *Jag1*$^{+/Ndr}$ and 8 *Jag1*$^{Ndr/Ndr}$ mice.

P30 mice were injected via tail vein with 100 µl PBS (*Jag1*$^{+/+}$, n = 3 males and n = 3 females) or with a tracer cocktail of Dextran-3 kDa-FITC (10 µg/g body weight, D3306, Invitrogen) and Cadaverin-1 kDa-555 (6 µg/g body weight, A30677, Invitrogen), (*Jag1*$^{+/+}$ n = 6 males and n = 6 females; *Jag1*$^{Ndr/Ndr}$ n = 6 males and n = 6 females). After 4 h of circulation, mice were anesthetized, and blood was collected by intracardiac puncture followed by perfusion with PBS for 3 min. Kidneys and brains were collected in PBS and *in situ* detection of fluorophore-conjugated tracers was done by imaging using Stereomicroscope Stemi 305 (Carl Zeiss Microscopy) equipped with a LabCam adaptor (iDu Optics) and iPhoneX.

Kidneys and brain were weighed and homogenized (Precellys24, Bertin technologies) in 300 µl of 1% Triton-PBS. The lysates were then centrifuged at 13,000 rpm at 4°C for 30 min and once more for 15 min. Plasma (100 µl, diluted ½ with PBS), retrieved using lithium-heparin tubes (Sarstedt, 20.1345) and supernatants (100 µl, undiluted) were used to quantify relative fluorescence values normalized to organ weights (POLARstar Omega plate reader, Labvision).

Whole organ hemorrhage analysis and imaging

Freshly dissected brains and retinas were inspected for the presence of hemorrhages under a stereomicroscope Stemi 305 (Carl Zeiss Microscopy). Whole organs or tissue images were taken with a stereomicroscope Stemi 305 (Carl Zeiss Microscopy) combined with Canon camera (Cannon, PowerShot S3 IS or Iphone6). The background around the tissue (brain, retina) was covered by a solid black color in Adobe Photoshop for esthetic purposes.

Survival analysis

Newborn pups were observed, and live/deceased mice were counted daily during the first 10 days after birth. The pups were biopsied, tattooed, and genotyped. 211 *Jag1*$^{+/+}$ and 46 *Jag1*$^{Ndr/Ndr}$ were included in the analysis, 14 *Jag1*$^{Ndr/Ndr}$ pups died during the first 10 days.

Electron microscopy

Animals were anesthetized by CO_2 inhalation and transcardially perfused with HBSS and electron microscopy fixative (2% formaldehyde, 2.5% glutaraldehyde and 0.02% sodium azide in 0.05 M sodium cacodylate buffer, pH 7.2) as previously described (Henshall *et al*, 2015). For retina and heart analysis 6 *Jag1*$^{+/+}$ and 6 *Jag1*$^{Ndr/Ndr}$ mice were used. Images were further processed in Adobe Photoshop to pseudocolor cell types.

Quantitative real time qPCR

RNA was isolated from 6 *Jag1*$^{+/+}$ and 6 *Jag1*$^{Ndr/Ndr}$ P5 pups. mRNA was extracted using the RNeasy Mini Kit (QIAGEN, cat. #74104), including on-column DNase I digestion (QIAGEN, cat. #79254). Complementary DNA was synthesized from 1 µg total RNA using the Thermo Scientific™ Maxima™ First Strand cDNA Synthesis Kit for RT-qPCR (Thermo Scientific, cat. #K1641) according to the manufacturer instructions. Quantitative real-time PCR (qPCR) was performed as described (Jin *et al*, 2008). Primers used for qPCR are listed below. mRNA levels are normalized to the average housekeeping RNA levels of 18 S and β-actin.

Gene	Forward primer	Reverse primer	Product length (bp)
18 S	gtggagcgatttgtctggtt	cgctgagccagtcagtgtag	200
β-actin	gacaggatgcagaaggagat	ttgctgatccacatctgctg	146
Dll4	tttgcccagactccatcctcacag	ttccccccatctcccttattgg	356

Blood pressure measurements

Blood pressure was measured using the CODA® High Throughput (Kent Scientific) tail cuff system. The animals were awake, on a heating pad, and in a restraining device, during the experiment. The animals were accustomed to the tail cuff for 5 min before data recording. Tail blood pressure was assessed between 32 and 34°C. The systolic, diastolic, and mean blood pressure were recorded. The mean baseline blood pressure was calculated as an average from 5 recordings on 5 days. In the Angiotensin II experiment, the mean blood pressure is the average of nine recordings. The increase in blood pressure was calculated by comparing the average mean blood pressure before Angiotensin II treatment (100%) and during 2 weeks of Angiotensin II treatment.

© 2022 The Authors

Angiotensin II treatment

Adult mice (3–7.5 months) were treated with Angiotensin II (cat. #A6402, Sigma-Aldrich) for 2 weeks. Angiotensin II was delivered via osmotic mini pumps (Alzet®, 2002 model) with an infusion rate of 0.025 μg/g/h. The animals were anesthetized by Isoflurane inhalation (~ 2%) and IP injected with 200 μl Rimadyl (0.5 mg/ml) prior to the surgery to relieve pain. The osmotic mini pumps were implanted subcutaneously, and the incision was sealed with surgical glue. Animals in the experiment were weighed daily and the blood pressure was measured to verify the effect of Angiotensin II treatment. 2 of 11 $Jag1^{+/+}$ mice, and 1 of 8 $Jag1^{Ndr/Ndr}$ mice demonstrated a strong reaction to Angiotensin II treatment (weight loss > 20% and piloerection) and had to be sacrificed. 5 $Jag1^{+/+}$ and 2 $Jag1^{Ndr/Ndr}$ mice had weak or no response to Angiotensin II (no blood pressure increase) and were therefore excluded from the analysis. Animals were given extra "porridge" during the treatment to avoid dehydration. Control animals were implanted with mini-pumps loaded with Dulbecco's phosphate buffer saline (DPBS), in total 4 $Jag1^{+/+}$ and 5 $Jag1^{Ndr/Ndr}$ mice.

Plasma and serum collection

Blood from P10 mice was collected from the trunk after decapitation. To obtain blood plasma the blood was mixed with 10 μl of Heparin (LEO Pharma) and kept cold throughout handling. To obtain serum the blood was collected in a tube and allowed to coagulate at room temperature. Afterwards, the blood-Heparin mix was centrifuged at 3,000 *g* for 15 min at 4°C (plasma) or blood was centrifuged at 3,000 *g* for 15 min at room temperature (serum). Plasma or serum was carefully removed from the top layer, placed in a new tube, and frozen (−20°C short term (max 2 weeks), −80°C long-term storage). Blood plasma was collected from 6 $Jag1^{+/+}$ and 6 $Jag1^{Ndr/Ndr}$ mice and serum were collected from 6 $Jag1^{+/+}$ and 6 $Jag1^{Ndr/Ndr}$ mice.

Coagulation assays

Thrombin–Antithrombin (TAT) complexes were measured in blood plasma by ELISA using a commercial kit (cat. #ab137994, Abcam). The assay was performed according to manufacturer's instructions. The chromogen substrate was incubated for 20 min and the absorbance was read at 450 nm using VersaMax™ microplate reader. The TAT ELISA was performed on P10 plasma (6 $Jag1^{+/+}$ and 6 $Jag1^{Ndr/Ndr}$ mice). Fibrinogen was measured in P10 serum (6 $Jag1^{+/+}$ and 6 $Jag1^{Ndr/Ndr}$ mice) using a commercial kit (cat. #ab213478, Abcam), according to manufacturer's instructions. The chromogen substrate was incubated for 15 min and the absorbance was read at 450 nm using VersaMax™ microplate reader. INR was measured in a drop of fresh whole blood (6 $Jag1^{+/+}$ and 6 $Jag1^{Ndr/Ndr}$ mice at P10) with a commercially available point of care coagumeter (CoaguChek® XS, Roche). Tail bleeding time was assessed in 6 $Jag1^{+/+}$ and 6 $Jag1^{Ndr/Ndr}$ mice at P10 in anesthetized (Isoflurane inhalation) pups by severing the tip of the tail (2 mm from the tip) and gently wiping the tail on a tissue paper without squeezing the tail. The time was recorded from the moment the tail was cut. The tail was wiped every 20 s to observe the bleeding. The bleeding was considered stopped when no bleeding reappeared after 1 min.

The paper explained

Problem
Vascular abnormalities, especially intracranial bleeds, are a major cause of death in patients with Alagille syndrome. Coagulopathy is associated with cholestatic liver diseases, but is not present in all patients with Alagille syndrome who experience intracranial bleeding. Hence, risk factors contributing to vascular anomalies and bleeding events remain largely unknown. Furthermore, there is no reported suitable animal model to investigate the vascular bleeds in Alagille syndrome, that is representative of Alagille syndrome heterogeneity and multisystem presentation.

Results
We performed a systematic review studying patients with Alagille syndrome and vascular anomalies, which revealed that girls were reported 10 times more often with spontaneous intracranial bleeds than boys. We further established that the $Jag1^{Ndr/Ndr}$ mouse model recapitulates sporadic brain hemorrhages and female mutant mice showed strongly affected veins compared to males. Finally, we show that vascular defects discovered in the $Jag1^{Ndr/Ndr}$ mouse model can also be identified in patients with Alagille syndrome non-invasively, in patient retinographs. We identified female sex, hypertension, and aging as risk factors further aggravating vascular disease in Alagille syndrome.

Impact
Our data provide the first evidence of sexual dimorphism in patients with Alagille syndrome. Our findings suggest that young girls with Alagille syndrome should be monitored with higher frequency for the presence of an aneurysm. Furthermore, hypertensive patients with Alagille syndrome may be at increased risk of developing vascular disease associated with vascular smooth muscle cells loss. Importantly, the $Jag1^{Ndr/Ndr}$ mouse model could serve as a tool for testing potential therapies for patients with Alagille syndrome. Finally, retinography should be further investigated as a tool for non-invasive vascular investigation in this pediatric population.

Liver enzyme analysis

Liver enzymes (including total bilirubin) were analyzed from blood plasma of 6 $Jag1^{+/+}$ and 6 $Jag1^{Ndr/Ndr}$ mice P10 mice diluted six fold with DPBS, using mammalian liver profile (Abaxis, PN 500-1040) and VetScan2 system (Abaxis Inc).

Systematic review

The systematic review was performed according to the PRISMA guidelines. For details on search strategies and PRISMA checklist, please see the Appendix Supplementary Methods.

Data availability

https://github.com/jakublaznovsky/Spongy-bone-segmentation.

Expanded View for this article is available online.

Acknowledgements

We thank Bettina Semsch, Soniya Savant, Katarina Tiklova, Liqun He, Christer Betsholtz, Tomasz Krzywkowski, Mats Nilsson, Rickard Sandberg, Gösta Winberg, Jianshe Wang, Liting Li and Daniel Oliveira for their help and

© 2022 The Authors

contribution to the manuscript. We thank the Biomedicum Imaging Core for their input on image analysis. We thank the Karolinska Mouse Models facility for their help with blood pressure measurements. The monoclonal antibody NF-RT97 developed by Wood, J. obtained from the Developmental Studies Hybridoma Bank, created by the NICHD of the NIH and maintained at The University of Iowa, Department of Biology, Iowa City, IA 52242. This works was supported by Center of Innovative Medicine (CIMED) Grant (2-538/2014-29; ERA); Ragnar Söderberg Foundation (Swedish Foundations' Starting Grant, S11/20; ERA); Swedish Research Council (2019-01350; ERA); EASL Daniel Alagille Award (ERA); Heart and Lung Foundation (20170723) ERA; KI Funding (2-2110/2019-7, 2-560/2015-280) ERA; Alex and Eva Wallström Foundation (ERA); Wera Ekströms Stiftelse (SH); "KI-MU" program co-financed from European Social Fund and the state budget of the Czech Republic (CZ.1.07/2.3.00/20.0180; VB); Czech Science Foundation project Expro (GX19-28347X; VB); CzechNanoLab Research Infrastructure supported by MEYS CR (LM2018110; JK).

Author contributions

Simona Hankeova: Conceptualization; data curation; formal analysis; investigation; visualization; methodology; writing – original draft. **Noémi KM Van Hul:** Data curation; formal analysis; investigation; visualization; methodology; writing – review and editing. **Jakub Laznovsky:** Formal analysis; investigation; visualization; methodology; writing – review and editing. **Elisabeth Verboven:** Formal analysis; investigation; visualization; methodology; writing – review and editing. **Katrin Mangold:** Formal analysis; investigation. **Naomi Hensens:** Formal analysis; investigation; visualization. **Csaba Adori:** Investigation; methodology. **Elvira Verhoef:** Formal analysis; investigation; visualization. **Tomas Zikmund:** Conceptualization; supervision. **Feven Dawit:** Investigation. **Michaela Kavkova:** Formal analysis; investigation; writing – review and editing. **Jakub Salplachta:** Formal analysis; investigation; visualization; writing – review and editing. **Marika Sjoqvist:** Investigation. **Bengt R Johansson:** Visualization; methodology. **Mohamed G Hassan:** Visualization. **Linda Fredriksson:** Resources; writing – review and editing. **Karsten Baumgartel:** Resources; writing – review and editing. **Vitezslav Bryja:** Funding acquisition; writing – review and editing. **Urban Lendahl:** Funding acquisition; writing – review and editing. **Andrew Jheon:** Investigation. **Florian Alten:** Resources; writing – review and editing. **Kristina, Teär Fahnehjelm:** Resources; writing – review and editing. **Björn Fischler:** Resources; investigation; writing – review and editing. **Jozef Kaiser:** Conceptualization; supervision; funding acquisition; writing – review and editing. **Emma Andersson:** Conceptualization; resources; formal analysis; supervision; funding acquisition; investigation; visualization; methodology; writing – original draft.

Disclosure and competing interests statement

One project in ERA lab is funded by modeRNA, unrelated to this manuscript (no personal remuneration). Travere funded analysis of *Jag1*$^{\Delta DSL/+}$ mice. UL holds a research grant from Merck Healthcare KGaA, but no personal remuneration, unrelated to this manuscript.

For more information

https://www.omim.org/entry/118450
https://anderssonlab.com/
https://alagille.org/

References

Agrawal S, Chennuri V, Agrawal P (2015) Genetic diagnosis in an Indian child with Alagille syndrome. *Indian J Pediatr* 82: 653–654

Alagille D, Odièvre M, Gautier M, Dommergues JP (1975) Hepatic ductular hypoplasia associated with characteristic facies, vertebral malformations, retarded physical, mental, and sexual development, and cardiac murmur. *J Pediatr* 86: 63–71

Alagille D, Estrada A, Hadchouel M, Gautler M, Odièvre M, Dommergues JP (1987) Syndromic paucity of interlobular bile ducts (Alagille syndrome or arteriohepatic dysplasia): review of 80 cases. *J Pediatr* 110: 195–200

Alten F, Motte J, Ewering C, Osada N, Clemens CR, Kadas EM, Eter N, Paul F, Marziniak M (2014) Multimodal retinal vessel analysis in CADASIL patients. *PLoS One* 9: e112311

Andersson ER, Chivukula IV, Hankeova S, Sjöqvist M, Tsoi YL, Ramsköld D, Masek J, Elmansuri A, Hoogendoorn A, Vazquez E *et al* (2018) Mouse model of Alagille syndrome and mechanisms of jagged1 missense mutations. *Gastroenterology* 154: 1080–1095

Appelros P, Åsberg S (2020) Sex differences in stroke. *Handb Clin Neurol* 175: 299–312

Arboleda-Velasquez JF, Manent J, Lee JH, Tikka S, Ospina C, Vanderburg CR, Frosch MP, Rodríguez-Falcón M, Villen J, Gygi S *et al* (2011) Hypomorphic Notch 3 alleles link Notch signaling to ischemic cerebral small-vessel disease. *Proc Natl Acad Sci U S A* 108: E128

Armulik A, Genove G, Mäe M, Nisancioglu MH, Wallgard E, Niaudet C, He L, Norlin J, Lindblom P, Strittmatter K *et al* (2010) Pericytes regulate the blood–brain barrier. *Nature* 468: 557–561

Baird LC, Smith ER, Ichord R, Piccoli DA, Bernard TJ, Spinner NB, Scott RM, Kamath BM (2015) Moyamoya syndrome associated with Alagille syndrome: outcome after surgical revascularization. *J Pediatr* 166: 470–473

Benedito R, Roca C, Sörensen I, Adams S, Gossler A, Fruttiger M, Adams RH (2009) The notch ligands Dll4 and Jagged1 have opposing effects on angiogenesis. *Cell* 137: 1124–1135

Berard E, Sarles J, Triolo V, Gagnadoux MF, Wernert F, Hadchouel M, Niaudet P (1998) Renovascular hypertension and vascular anomalies in Alagille syndrome. *Pediatr Nephrol* 12: 121–124

Bucuvalas JC, Horn JA, Carlsson L, Balistreri WF, Chernausek SD (1993) Growth hormone insensitivity associated with elevated circulating growth hormone-binding protein in children with Alagille syndrome and short stature. *J Clin Endocrinol Metab* 76: 1477–1482

Carpenter CD, Linscott LL, Leach JL, Vadivelu S, Abruzzo T (2018) Spectrum of cerebral arterial and venous abnormalities in Alagille syndrome. *Pediatr Radiol* 48: 602–608

Chen RF (1973) The fluorescence of bilirubin-albumin complexes. In *Fluorescence techniques in cell biology*, AA Thaer, M Sernetz (eds), pp 273–282. Berlin, Heidelberg: Springer Berlin Heidelberg

Cowan JA, Barkhoudarian G, Yang LJS, Thompson BG (2004) Progression of a posterior communicating artery infundibulum into an aneurysm in a patient with Alagille syndrome. Case report. *J Neurosurg* 101: 694–696

Crosnier C, Attié-Bitach T, Encha-Razavi F, Audollent S, Soudy F, Hadchouel M, Meunier-Rotival M, Vekemans M (2000) JAGGED1 gene expression during human embryogenesis elucidates the wide phenotypic spectrum of Alagille syndrome. *Hepatology* 32: 574–581

Doberentz E, Kuchelmeister K, Madea B (2015) Subarachnoid hemorrhage due to aneurysm rupture in a young woman with Alagille syndrome - a rare cause of sudden death. *Leg Med* 17: 309–312

El-Koofy NM, El-Mahdy R, Fahmy ME, El-Hennawy A, Farag MY, El-Karaksy HM (2011) Alagille syndrome: clinical and ocular pathognomonic features. *Eur J Ophthalmol* 21: 199–206

Emerick KM, Rand EB, Goldmuntz E, Krantz ID, Spinner NB, Piccoli DA (1999) Features of Alagille syndrome in 92 patients: frequency and relation to prognosis. *Hepatology* 29: 822–829

© 2022 The Authors

Emerick KM, Krantz ID, Kamath BM, Darling C, Burrowes DM, Spinner NB, Whitington PF, Piccoli DA (2005) Intracranial vascular abnormalities in patients with Alagille syndrome. *J Pediatr Gastroenterol Nutr* 41: 99 – 107

Fahnehjelm KT, Wide K, Ygge J, Hellström A, Tomson T, Winbladh B, Strömland K (1999) Visual and ocular outcome in children after prenatal exposure to antiepileptic drugs. *Acta Ophthalmol Scand* 77: 530 – 535

Fahnehjelm KT, Fischler B, Martin L, Nemeth A (2011) Occurrence and pattern of ocular disease in children with cholestatic disorders. *Acta Ophthalmol* 89: 143 – 150

Fiorda-Diaz J, Shabsigh M, Dimitrova G, Soghomonyan S, Sandhu G (2017) Perioperative management of subarachnoid hemorrhage in a patient with alagille syndrome and unrepaired tetralogy of fallot: case report. *Front Surg* 4: 72

Frongillo F, Bianco G, Silvestrini N, Lirosi MC, Sanchez AM, Nure E, Gaspari R, Avolio AW, Sganga G, Agnes S (2015) Acute liver failure in an adult, a rare complication of Alagille syndrome: case report and brief review. *Transplant Proc* 47: 2179 – 2181

Fuentes AM, Du X, Pandey DK, Ansari SA, Amin-hanjani S (2022) Abstract WP149: Mechanical thrombectomy for ischemic stroke: evaluating outcome associations with race. *Stroke* 53: AWP149

Gaba RC, Shah RP, Muskovitz AA, Guzman G, Michals EA (2008) Synchronous moyamoya syndrome and ruptured cerebral aneurysm in Alagille syndrome. *J Clin Neurosci* 15: 1395 – 1398

Han HC (2012) Twisted blood vessels: Symptoms, etiology and biomechanical mechanisms. *J Vasc Res* 49: 185 – 197

Hankeova S, Salplachta J, Zikmund T, Kavkova M, Hul V, Brinek A, Smekalova V, Laznovsky J, Lendahl U, Ellis E *et al* (2021) DUCT reveals architectural mechanisms contributing to bile duct recovery in a mouse model for Alagille syndrome. *eLife* 10: e60916

Henshall TL, Keller A, He L, Johansson BR, Wallgard E, Raschperger E, Andaloussi Mae M, Jin S, Betsholtz C, Lendahl U (2015) Notch3 is necessary for blood vessel integrity in the central nervous system. *Arterioscler Thromb Vasc Biol* 35: 409 – 420

High FA, Lu MM, Pear WS, Loomes KM, Kaestner KH, Epstein JA (2008) Endothelial expression of the Notch ligand Jagged1 is required for vascular smooth muscle development. *Proc Natl Acad Sci U S A* 105: 1955 – 1959

Hingorani M, Nischal KK, Davies A, Bentley C, Vivian A, Baker AJ, Mieli-Vergani G, Bird AC, Aclimandos WA (1999) Ocular abnormalities in Alagille syndrome. *Ophthalmology* 106: 330 – 337

Hoffenberg EJ, Narkewicz MR, Sondheimer JM, Smith DJ, Silverman A, Sokol RJ (1995) Outcome of syndromic paucity of interlobular bile ducts (Alagille syndrome) with onset of cholestasis in infancy. *J Pediatr* 127: 220 – 224

Hughes AD, Falaschetti E, Witt N, Wijetunge S, Thom SAMG, Tillin T, Aldington SJ, Chaturvedi N (2016) Association of retinopathy and retinal microvascular abnormalities with stroke and cerebrovascular disease. *Stroke* 47: 2862 – 2864

Jin S, Hansson EM, Tikka S, Lanner F, Sahlgren C, Farnebo F, Baumann M, Kalimo H, Lendahl U (2008) Notch signaling regulates platelet-derived growth factor receptor-β expression in vascular smooth muscle cells. *Circ Res* 102: 1483 – 1491

Joutel A, Corpechot C, Ducros A, Vahedi K, Chabriat H, Mouton P, Alamowitch S, Domenga V, Cécillion M, Marechal E *et al* (1996) Notch3 mutations in CADASIL, a hereditary adult-onset condition causing stroke and dementia. *Nature* 383: 707 – 710

Kamath BM, Spinner NB, Emerick KM, Chudley AE, Booth C, Piccoli DA, Krantz ID (2004) Vascular anomalies in Alagille syndrome: a significant cause of morbidity and mortality. *Circulation* 109: 1354 – 1358

Kazi R, Fakhoury J, Beck R (2018) A case of recurrent intracranial bleeds in the setting of Alagille syndrome. *Pediatrics* 142: 52

Kergoat H, Herard M-E, Lemay M (2006) RGC sensitivity to mild systemic hypoxia. *Invest Ophthalmol Vis Sci* 47: 5423 – 5427

Kim BJ, Fulton AB (2007) The genetics and ocular findings of Alagille syndrome. *Semin Ophthalmol* 22: 205 – 210

Kwa VIH, van der Sande JJ, Stam J, Tijmes N, Vrooland JL, Amsterdam Vascular Medicine Group (2002) Retinal arterial changes correlate with cerebral small-vessel disease. *Neurology* 59: 1536 – 1540

Lentz R, Merrick C, Rickman O, Maldonado F (2016) Endobronchial Nd:YAP laser ablation of a bleeding dieulafoy lesion of the bronchus. *Chest* 150: 989A

Li L, Krantz ID, Deng Y, Genin A, Banta AB, Collins CC, Qi M, Trask BJ, Kuo WL, Cochran J *et al* (1997) Alagille syndrome is caused by mutations in human Jagged1, which encodes a ligand for Notch1. *Nat Genet* 16: 243 – 251

Lykavieris P, Crosnier C, Trichet C, Meunier-Rotival M, Hadchouel M (2003) Bleeding tendency in children with Alagille syndrome. *Pediatrics* 111: 167 – 170

Machuca-Parra AI, Bigger-Allen AA, Sanchez A v, Boutabla A, Cardona-Vélez J, Amarnani D, Saint-Geniez M, Siebel CW, Kim LA, D'Amore PA *et al* (2017) Therapeutic antibody targeting of Notch3 signaling prevents mural cell loss in CADASIL. *J Exp Med* 214: 282

Martin G, Wefelmeyer KL, Bucher F, Schlunck G, Agostini HT (2020) Arteriovenous crossing in retinal vessels of mice, rats, and pigs. *Mol Vis* 26: 731 – 741

Mašek J, Andersson ER (2017) The developmental biology of genetic Notch disorders. *Development* 144: 1743 – 1763

Matsuura G, Higashimoto Y, Iwai J, Murayama K, Horie H, Suzuki T (2013) A case of Alagille syndrome with hepatic-duct obliteration presenting intracranial hemorrhage. *Chiba Med J* 89: 181 – 185

McDaniell R, Warthen DM, Sanchez-Lara PA, Pai A, Krantz ID, Piccoli DA, Spinner NB (2006) NOTCH2 mutations cause Alagille syndrome, a heterogeneous disorder of the notch signaling pathway. *Am J Hum Genet* 79: 169 – 173

Mhurchu CN, Anderson C, Jamrozik K, Hankey G, Dunbabin D (2001) Hormonal factors and risk of aneurysmal subarachnoid hemorrhage: an international population-based, case–control study cliona. *Stroke* 32: 606 – 612

Mills KT, Bundy JD, Kelly TN, Reed JE, Kearney PM, Reynolds K, Chen J, He J (2016) Global disparities of hypertension prevalence and control: a systematic analysis of population-based studies from 90 countries. *Circulation* 134: 441 – 450

Narula P, Gifford J, Steggall MA, Lloyd C, Van Mourik IDM, Mckiernan PJ, Willshaw HE, Kelly D (2006) Visual loss and idiopathic intracranial hypertension in children with Alagille syndrome. *J Pediatr Gastroenterol Nutr* 43: 348 – 352

Nelis P, Kleffner I, Burg MC, Clemens CR, Alnawaiseh M, Motte J, Marziniak M, Eter N, Alten F (2018) OCT-angiography reveals reduced vessel density in the deep retinal plexus of CADASIL patients. *Sci Rep* 8: 1 – 7

O'Connell D, Kaliaperumal C, Fanning N, Wyse G, Kaar G (2012) Superior cerebellar aneurysm causing subarachnoid haemorrhage in a 17-year-old with alagille syndrome. *Br J Neurosurg* 26: 287 – 289

Oda T, Elkahloun AG, Pike BL, Okajima K, Krantz ID, Genin A, Piccoli DA, Meltzer PS, Spinner NB, Collins FS *et al* (1997) Mutations in the human Jagged1 gene are responsible for Alagille syndrome. *Nat Genet* 16: 235 – 242

O'Donnell MJ, Xavier D, Liu L, Zhang H, Chin SL, Rao-Melacini P, Rangarajan S, Islam S, Pais P, McQueen MJ *et al* (2010) Risk factors for ischaemic and intracerebral haemorrhagic stroke in 22 countries (the INTERSTROKE study): a case–control study. *Lancet* 376: 112 – 123

Ong YT, de Silva DA, Cheung CY, Chang HM, Chen CP, Wong MC, Wong TY, Ikram MK (2013) Microvascular structure and network in the retina of patients with ischemic stroke. *Stroke* 44: 2121 – 2127

© 2022 The Authors

Page MJ, McKenzie JE, Bossuyt PM, Boutron I, Hoffmann TC, Mulrow CD, Shamseer L, Tetzlaff JM, Akl EA, Brennan SE *et al* (2021) The PRISMA 2020 statement: an updated guideline for reporting systematic reviews. *Syst Rev* 10: 89

Petaros A, Miletic D, Stifter S, Slaus M, Stemberga V (2015) Alagille syndrome case report: implications for forensic pathology and anthropology. *Int J Legal Med* 129: 543 – 549

Phng LK, Gerhardt H (2009) Angiogenesis: a team effort coordinated by notch. *Dev Cell* 16: 196 – 208

Pippucci T, Maresca A, Magini P, Cenacchi G, Palombo F, Papa V, Incensi A, Gasparre G, Lucia M, Preziuso C *et al* (2015) Homozygous NOTCH 3 null mutation and impaired NOTCH 3 signaling in recessive early-onset arteriopathy and cavitating leukoencephalopathy. *EMBO Mol Med* 7: 848 – 858

Qiao CZ, Richter GT, Pan WJ, Jin YB, Lin XX (2019) Extracranial arteriovenous malformations: from bedside to bench. *Mutagenesis* 34: 299 – 306

Quiros-Tejeira RE, Ament ME, Heyman MB, Martin MG, Rosenthal P, Hall TR, McDiarmid SV, Vargas JH (1999) Variable morbidity in alagille syndrome: a review of 43 cases. *J Pediatr Gastroenterol Nutr* 29: 431 – 437

Qureshi AI, Malik AA, Saeed O, Defillo A, Sherr GT, Suri MFK (2016) Hormone replacement therapy and the risk of subarachnoid hemorrhage in postmenopausal women. *J Neurosurg* 124: 45 – 50

Rachmel A, Zeharia A, Neuman-Levin M, Weitz R, Shamir R, Dinari G (1989) Alagille syndrome associated with Moyamoya disease. *Am J Med Genet* 33: 89 – 91

Reagan AM, Gu X, Paudel S, Ashpole NM, Zalles M, Sonntag WE, Ungvari Z, Csiszar A, Otalora L, Freeman WM *et al* (2018) Age-related focal loss of contractile vascular smooth muscle cells in retinal arterioles is accelerated by caveolin-1 deficiency. *Neurobiol Aging* 71: 1 – 12

Renier N, Adams EL, Kirst C, Wu Z, Azevedo R, Kohl J, Autry AE, Kadiri L, Umadevi Venkataraju K, Zhou Y *et al* (2016) Mapping of brain activity by automated volume analysis of immediate early genes. *Cell* 165: 1789 – 1802

Rensen SSM, Doevendans PAFM, van Eys GJJM (2007) Regulation and characteristics of vascular smooth muscle cell phenotypic diversity. *Neth Heart J* 15: 100 – 108

Rim TH, Teo AWJ, Yang HHS, Cheung CY, Wong TY (2020) Retinal vascular signs and cerebrovascular diseases. *J Neuroophthalmol* 40: 44 – 59

Rocha R, Soro MI, Leitão A, Luís M, Md S, Leão MM (2012) Moyamoya vascular pattern in Alagille syndrome. *Pediatr Neurol* 47: 125 – 128

de Rooij NK, Linn FHH, van der Plas JA, Algra A, Rinkel GJE (2007) Incidence of subarachnoid haemorrhage: a systematic review with emphasis on region, age, gender and time trends. *J Neurol Neurosurg Psychiatry* 78: 1365 – 1372

Ruchoux MM, Domenga V, Brulin P, Maciazek J, Limol S, Tournier-Lasserve E, Joutel A (2003) Transgenic mice expressing mutant Notch3 develop vascular alterations characteristic of cerebral autosomal dominant arteriopathy with subcortical infarcts and leukoencephalopathy. *Am J Pathol* 162: 329 – 342

Ruigrok YM, Buskens E, Rinkel GJE (2001) Attributable risk of common and rare determinants of subarachnoid hemorrhage. *Stroke* 32: 1173 – 1175

Sanada Y, Naya I, Katano T, Hirata Y, Yamada N, Okada N, Ihara Y, Onishi Y, Mizuta K (2019) Visceral artery anomalies in patients with Alagille syndrome. *Pediatr Transplant* 23: e13352

Sanes JR, Masland RH (2015) The types of retinal ganglion cells: current status and implications for neuronal classification. *Annu Rev Neurosci* 38: 221 – 246

Santamaria R, Lopez-Aguilera J, Gonzalez De Caldas R, Sanchez-Frias M, Garcia-Rios A, Buendia P, Aljama P (2016) Vascular abnormalities as predominant feature in alagille syndrome patients with a novel mutation in JAG1 gene. *J Hypertens* 34: e180

Schlosser H-G, Kerner T, Woiciechowsky C, Benndorf G (2004) Multiple cerebral aneurysms and subarachnoid hemorrhage in a patient with Alagille syndrome. *AJNR Am J Neuroradiol* 25: 1366 – 1367

Seals DR, Jablonski KL, Donato AJ (2011) Aging and vascular endothelial function in humans. *Clin Sci* 120: 357 – 375

Secomb TW (2016) Hemodynamics. *Compr Physiol* 6: 975 – 1003

Sheth HS, Maldonado F, Lentz RJ (2018) Two cases of Dieulafoy lesions of the bronchus with novel comorbid associations and endobronchial ablative management. *Medicine* 97: e9754

Stahl A, Connor KM, Sapieha P, Chen J, Dennison RJ, Krah NM, Seaward MR, Willett KL, Aderman CM, Guerin KI *et al* (2010) The mouse retina as an angiogenesis model. *Invest Ophthalmol Vis Sci* 51: 2813 – 2826

Thakurdas SM, Lopez MF, Kakuda S, Fernandez-Valdivia R, Zarrin-Khameh N, Haltiwanger RS, Jafar-Nejad H (2016) Jagged1 heterozygosity in mice results in a congenital cholangiopathy which is reversed by concomitant deletion of one copy of Poglut1 (Rumi). *Hepatology* 63: 550 – 565

Tiede A, Zieger B, Lisman T (2022) Acquired bleeding disorders. *Haemophilia* 28: 68 – 76

Tumialán LM, Dhall SS, Tomak PR, Barrow DL (2006) Alagille syndrome and aneurysmal subarachnoid hemorrhage. *Pediatr Neurosurg* 42: 57 – 61

Vandriel SM, Li L-T, She H, Wang J-S, Gilbert MA, Jankowska I, Czubkowski P, Gliwicz-Miedzińska D, Gonzales EM, Jacquemin E *et al* (2022) Natural history of liver disease in a large international cohort of children with Alagille syndrome: results from the GALA study. *Hepatology* https://doi.org/10.1002/hep.32761

Vlak MHM, Rinkel GJE, Greebe P, Greving JP, Algra A (2013) Lifetime risks for aneurysmal subarachnoid haemorrhage: multivariable risk stratification. *J Neurol Neurosurg Psychiatry* 84: 619 – 623

Volz KS, Jacobs AH, Chen HI, Poduri A, McKay AS, Riordan DP, Kofler N, Kitajewski J, Weissman I, Red-Horse K (2015) Pericytes are progenitors for coronary artery smooth muscle. *eLife* 4: e10036

Vorstman EBA, Anslow P, Keeling DM, Haythornthwaite G, Bilolikar H, McShane MT (2003) Brain haemorrhage in five infants with coagulopathy. *Arch Dis Child* 88: 1119 – 1121

Wang G-X, Zhang D, Wang Z-P, Yang L-Q, Yang H, Li W (2018) Risk factors for ruptured intracranial aneurysms. *Indian J Med Res* 147: 51 – 57

Wang X, Carcel C, Woodward M, Schutte AE (2022) Blood pressure and stroke: a review of sex- and ethnic/racial-specific attributes to the epidemiology, pathophysiology, and management of raised blood pressure. *Stroke* 53: 1114 – 1133

Woolfenden AR, Albers GW, Steinberg GK, Hahn JS, Johnston DCC, Farrell K (1999) Moyamoya syndrome in children with Alagille syndrome: additional evidence of a vasculopathy. *Pediatrics* 103: 505 – 508

Xiromerisiou G, Marogianni C, Dadouli K, Zompola C, Georgouli D, Provatas A, Theodorou A, Zervas P, Nikolaidou C, Stergiou S *et al* (2020) Cerebral autosomal dominant arteriopathy with subcortical infarcts and leukoencephalopathy revisited: genotype–phenotype correlations of all published cases. *Neurol Genet* 6: e434

Zuurbier CCM, Molenberg R, Mensing LA, Wermer MJH, Juvela S, Lindgren AE, Jääskeläinen JE, Koivisto T, Yamazaki T, Uyttenboogaart M *et al* (2022) Sex difference and rupture rate of intracranial aneurysms: an individual patient data meta-analysis. *Stroke* 53: 362 – 369

License: This is an open access article under the terms of the Creative Commons Attribution License, which permits use, distribution and reproduction in any medium, provided the original work is properly cited.

Expanded View Figures

Figure EV1. ***Jag1$^{Ndr/Ndr}$* mice display thinner skulls.**

A Micro computed tomography (μCT) of P30 skulls. Blue arrow marks side skull protrusions in *Jag1$^{Ndr/Ndr}$* mice.

B, C (B) Color map displaying skull full thickness. (C) Cross-section of dorsal cranium in fully segmented skull.

D, E (D) Color map displaying cranial compact bone thickness. (E) Cross-section of dorsal cranium of segmented compact bones.

F Skull length from occipital bone to nasal bone (measured in mid line), (n = 5-6 per group, Two-way ANOVA followed by Šídák's multiple comparison test, Interaction P = 0.9309, Sex P = 0.0069, Genotype **P = 0.0071).

G, H (G) Segmented temporal bone. (H) Temporal bone volume (n = 5-6 per group, Two-way ANOVA followed by Šídák's multiple comparison test, Interaction P = 0.9960, Sex P = 0.3691, Genotype *P = 0.0249).

Data information: Bar graphs depict mean values ± standard deviation, each dot represents one biological replicate. Circles represent females, squares represent males. For details/results of statistical analyses, please see source data.
Source data are available online for this figure.

 © 2022 The Authors

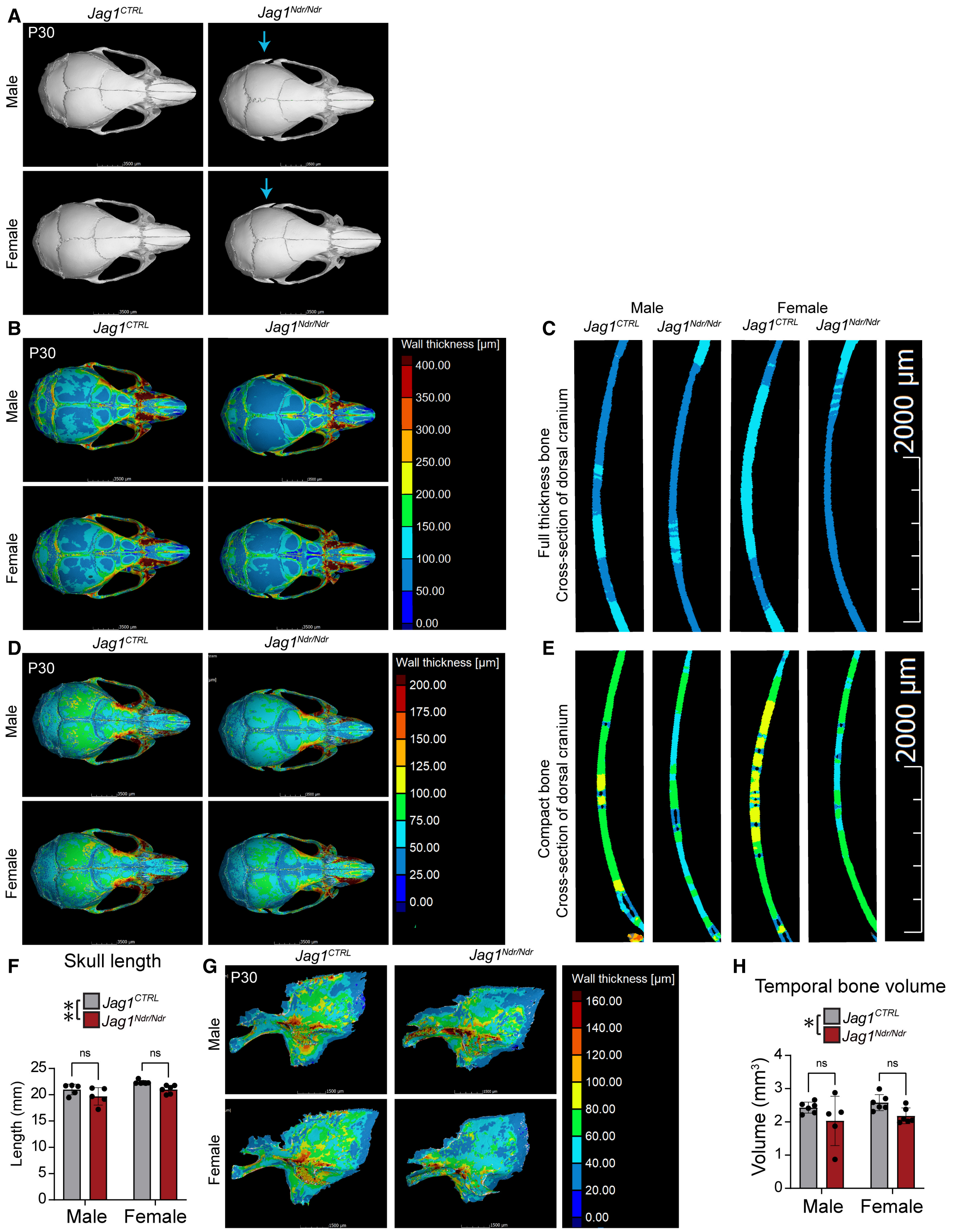
A
Jag1CTRL
Jag1Ndr/Ndr
P30
Male
Female
B
Wall thickness [µm]
400.00
350.00
300.00
250.00
200.00
150.00
100.00
50.00
0.00
C
Male
Female
Full thickness bone
Cross-section of dorsal cranium
2000 µm
D
Wall thickness [µm]
200.00
175.00
150.00
125.00
100.00
75.00
50.00
25.00
0.00
E
Compact bone
Cross-section of dorsal cranium
2000 µm
F
Skull length
Length (mm)
ns
Male
Female
G
Wall thickness [µm]
160.00
140.00
120.00
100.00
80.00
60.00
40.00
20.00
0.00
H
Temporal bone volume
Volume (mm3)
ns
Male
Female

Figure EV1.

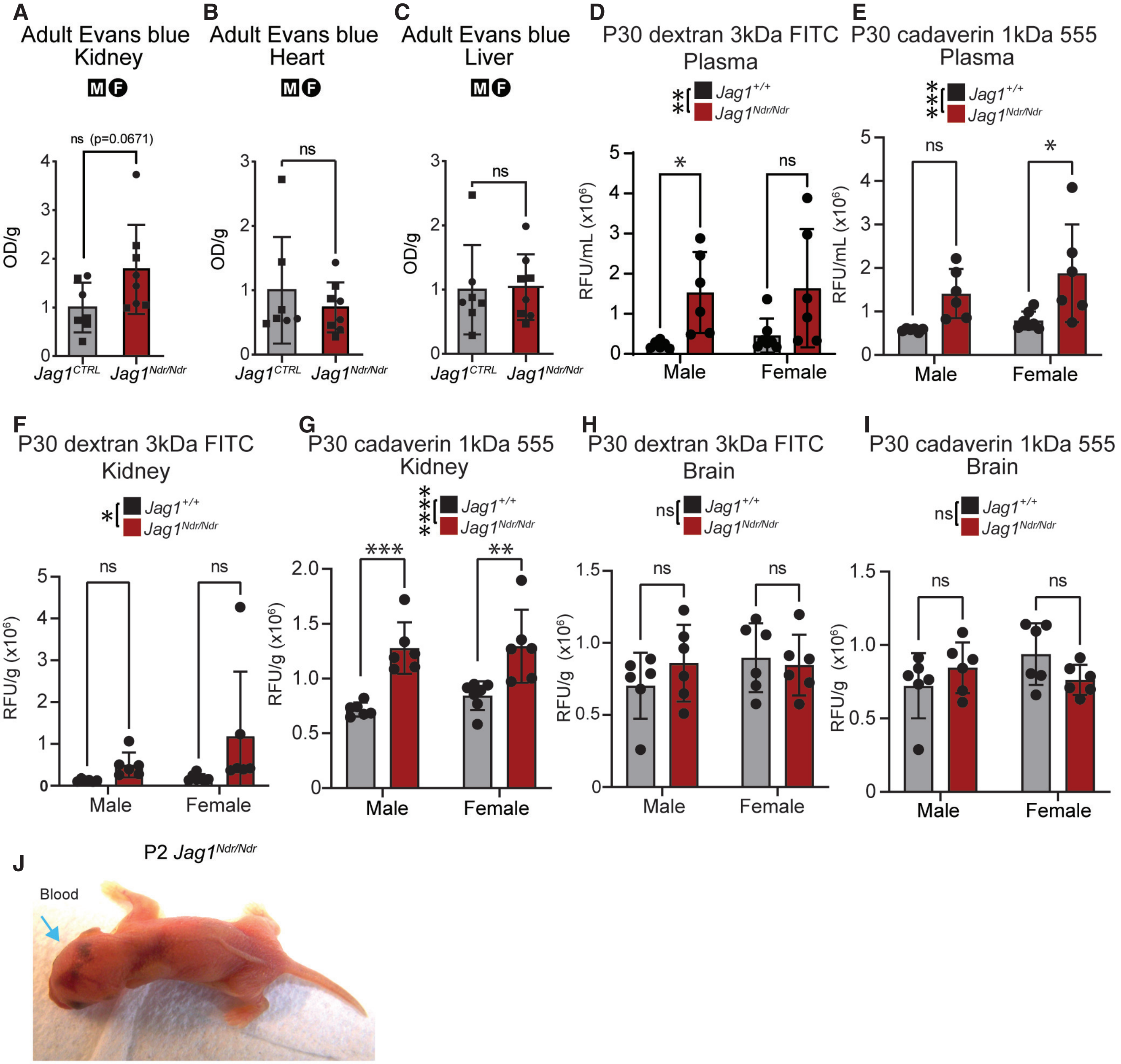

Figure EV2. ***Jag1*$^{Ndr/Ndr}$ mice exhibit selective renal vascular permeability.**

A–C Relative vascular leakage as assessed by Evans blue extracted from adult (A) kidney ($P = 0.0671$, ns), (B) heart ($P = 0.4316$, ns), and (C) liver ($P = 0.8988$, ns) of *Jag1*^{CTRL} and *Jag1*$^{Ndr/Ndr}$ adult mice (n = 7-8 per group, Unpaired t-test).

D, E Relative fluorescence in P30 plasma (D) of 3 kDa Dextran FITC (Two-way ANOVA on biological replicates/individual mice, followed by Šídák's multiple comparison test; Interaction $P = 0.08663$, Sex $P = 0.6541$, Genotype $^{**}P = 0.0025$) or (E) 1 kDa Cadaverin 555 (Two-way ANOVA on biological replicates/individual mice followed by Šídák's multiple comparison test; Interaction $P = 0.625$, Sex $P = 0.186$, Genotype $^{***}P = 0.0009$).

F, G Relative vascular permeability in P30 kidney assessed by relative fluorescence of (F) 3 kDa Dextran FITC (Two-way ANOVA on biological replicates/individual mice, followed by Šídák's multiple comparison test; Interaction $P = 0.3296$, Sex $P = 0.2374$, Genotype $^{*}P = 0.0384$) (G) or 1 kDa Cadaverin 555 (Two-way ANOVA on biological replicates/individual mice, followed by Šídák's multiple comparison test; Interaction $P = 0.5308$, Sex $P = 0.4112$, Genotype $^{****}P < 0.0001$).

H, I Relative vascular permeability in P30 brain assessed by relative fluorescence of (H) 3 kDa Dextran FITC (Two-way ANOVA on biological replicates/individual mice, followed by Šídák's multiple comparison test; Interaction $P = 0.2942$, Sex $P = 0.3624$, Genotype $P = 0.591$) or (I) 1 kDa Cadaverin 555 (Two-way ANOVA on biological replicates/individual mice, followed by Šídák's multiple comparison test; Interaction $P = 0.0596$, Sex $P = 0.3806$, Genotype $P = 0.7301$).

J Hemorrhages in brain and body of one *Jag1*$^{Ndr/Ndr}$ pup at P2, corresponding to brain data in Fig 2M, brain dissected out the next day at P3

Data information: Bar graphs depict mean values ± standard deviation, each dot represents one biological replicate. Circles represent females, squares represent males (A-C). For details/results of statistical analyses, please see source data.
Source data are available online for this figure.

© 2022 The Authors

Figure EV3. Delayed retinal vascular outgrowth and remodeling in *Jag1*$^{Ndr/Ndr}$ mice.

A Schematic depicting retinal angiogenesis between P0 and P15. S, superficial; I, intermediate; D, deep capillary plexus.

B–F (B) P5 retinal vascular outgrowth, (C) quantified (*n* = 6 per group, unpaired *t*-test, *P = 0.0388). Scale bar 100 μm. (D) P5 vascular front with tip cells (boxed region). White arrowhead points to ERG+ tip cell nucleus, (E) quantified (*n* = 6, unpaired *t*-test, P = 0.1281, ns), blue arrowheads point to tips (bundles of filopodia) of tip cell (F) quantified (*n* = 6, unpaired *t*-test, **P = 0.0059). Scale bar 20 μm.

G–M Retinal vasculature at (G) P5, (H) P10, (I) P15. Scale bar (G) 50 μm, (H, I) 20 μm. Retinal blood vessel remodeling quantification at P5, P10, and P15 (J) vascular length per field (Two-way ANOVA on biological replicates/individual mice. Interaction ****$P < 0.0001$, Age ***P = 0.0006, Genotype P = 0.2023. Šídák's multiple comparison test: P5 *Jag1*^{CTRL} vs. *Jag1*$^{Ndr/Ndr}$ ****$P < 0.0001$; P15 *Jag1*^{CTRL} vs. *Jag1*$^{Ndr/Ndr}$ *P = 0.0233), (K) number of ERG+ cells per field (Two-way ANOVA on biological replicates/individual mice. Interaction ****$P < 0.0001$, Age ****$P < 0.0001$, Genotype P = 0.7656. Šídák's multiple comparison test: P5 *Jag1*^{CTRL} vs *Jag1*$^{Ndr/Ndr}$ **P = 0.0013; P15 *Jag1*^{CTRL} vs *Jag1*$^{Ndr/Ndr}$ *P = 0.0217), (L) number of ERG+ cells per vascular length (Two-way ANOVA on biological replicates/individual mice. Interaction P = 0.4617, Age **P = 0.0018, Genotype P = 0.2235), (M) number of branching points per field (*n* = 6 per group, Two-way ANOVA on biological replicates/individual mice. Interaction ****$P < 0.0001$, Age ****$P < 0.0001$, Genotype P = 0.2719. Šídák's multiple comparison test: P5 *Jag1*^{CTRL} vs. *Jag1*$^{Ndr/Ndr}$ ****$P < 0.0001$).

N Immunofluorescence of PH3+ proliferating CD31+ endothelial cells at P5. The dotted line labels the edge of the vascular front. Quantification of the number of proliferating cells per radial zone, normalized to area size at P5 (*n* = 4, Two-way ANOVA on biological replicates/individual mice. Interaction P = 0.3044, Zone ****$P < 0.0001$, Genotype *P = 0.0165). Scale bar 50 μm.

O, P (O) Delta like 4 in P5 vasculature. Scale bar 20 μm. White brackets denote high DLL4 activity. (P) *Dll4* relative mRNA levels in whole retina lysates (*n* = 6, unpaired *t*-test, *P = 0.0461).

Data information: Bar graphs depict mean values ± standard deviation, each dot represents one biological replicate. Circles represent females, squares represent males. For details/results of statistical analyses, please see source data.
Source data are available online for this figure.

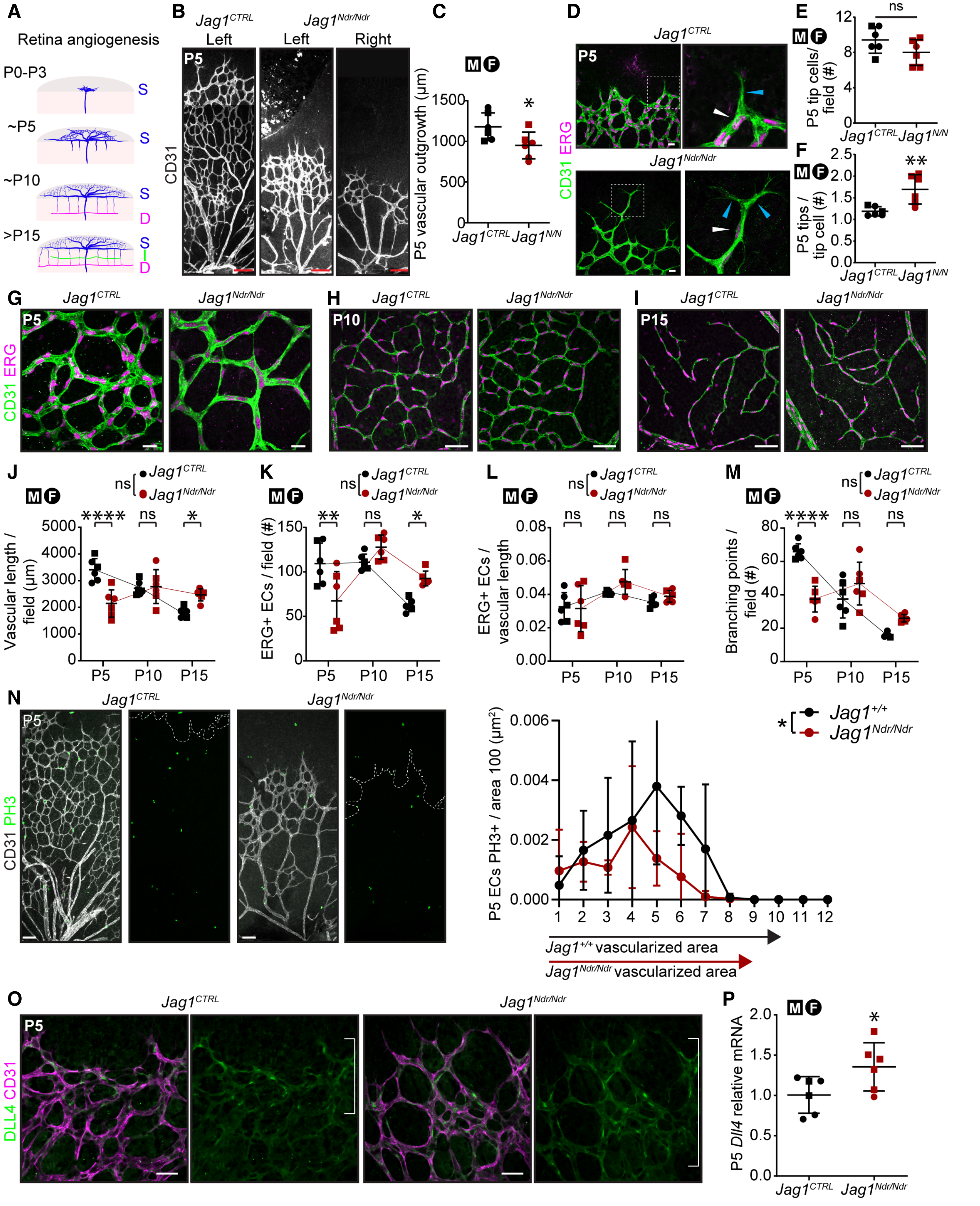

Figure EV3.

© 2022 The Authors

Figure EV4. *Jag1$^{Ndr/Ndr}$* mice display CADASIL-like sparse vascular smooth muscle cell coverage of arteries with an increase in artery-associated reactive astrocytes.

A, B (A) CD13 pericyte coverage of blood vessels was not reduced in adult *Jag1$^{Ndr/Ndr}$* mice. Scale bars 20 μm. (B) Quantification of CD13 intensity per field (*n* = 5-6 per group, Two-way ANOVA followed by Šídák's multiple comparison test, Interaction *P* = 0.0737, Sex *P* = 0.1642, Genotype *P* = 0.3706).

C Transmission electron microscopy of coronary arteries of adult mice. Vascular smooth muscle cells (SMC) are pseudo-colored in magenta and endothelial cells (ECs) in green. White arrowheads label SMC edges and the distances between SMCs. Green arrowhead marks the tight junctions. Scale bars 2 μm.

D, E (D) Staining for contractile (αSMA, SM22) and synthetic (VIM) vascular SMC (E) with quantification (*n* = 6–9 per group, Multiple unpaired *t*-tests, αSMA **P* = 0.0214, SM22 **P* = 0.0302, VIM *P* = 0.6801). Boxed region indicates region with αSMA-negative gap (labeled by white arrowhead). Scale bar 20 μm.

F, G (F) Staining for contractile (αSMA, MYH11) and synthetic (COLI) vascular SMC. Boxed region indicates region with αSMA-negative gap (labeled by white arrowhead). Scale bar 20 μm. (G) Relative decrease in fluorescence intensity of different vascular SMC markers within gap compared to within vascular SMC (set to 100%), (*n* = 5-11 per group, one-way ANOVA *P* < 0.0001, followed by Dunnett's multiple comparisons test: ASMA vs. COLI Adjusted ***P* = 0.0066, ASMA vs. VIM adjusted *****P* < 0.0001).

H–K (H) GFAP+ astrocytes are more prevalent around adult *Jag1$^{Ndr/Ndr}$* arterioles (I) but not veins. White arrowheads label reactive astrocytes. Scale bars 20 μm. (J, K) Quantification of mean GFAP intensity on retinal (J) arterioles (*n* = 4, unpaired *t*-test, ***P* = 0.0052), (K) venules (*n* = 4, unpaired *t*-test, *P* = 0.6547).

L, M (L) Number of arteriolar first-generation branch points in 1-year-old mice (*n* = 6, unpaired *t*-test, *P* = 0.6823). (M) Number of cCasp3+ cells associated with first-generation arteriolar branching point (*n* = 6, unpaired *t*-test, **P* = 0.0153).

N Evans blue extracted from liver, kidney, and heart of mice treated with PBS or Angiotensin II (*n* = 4-5 per group, two-way ANOVA with Subject matching. Interaction *P* = 0.2576, Organ *P* = 0.0011, Genotype & Treatment *P* = 0.2086 Subject ****P* = 0.0004, followed by Tukey's multiple comparison test, *Jag1$^{+/+}$* vs. *Jag1$^{+/+}$* AngII **P* < 0.05).

Data information: Bar graphs depict mean values ± standard deviation, each dot represents one biological replicate. Circles represent females, squares represent males. For details/results of statistical analyses, please see source data. A, arteriole; AngII, Angiotensin II; EC, endothelial cell; SMC, smooth muscle cell; V, venule.
Source data are available online for this figure.

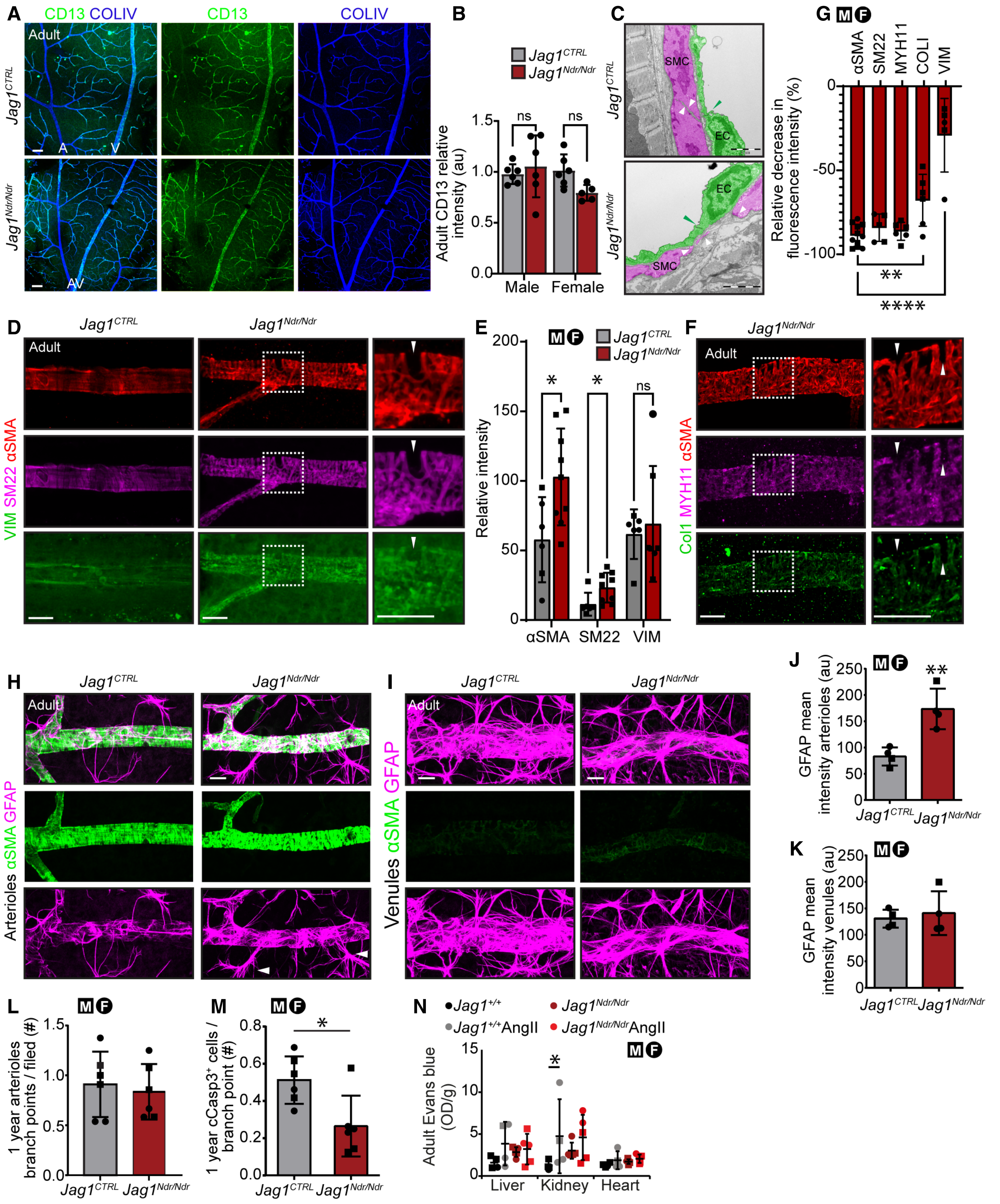

Figure EV4.

 © 2022 The Authors

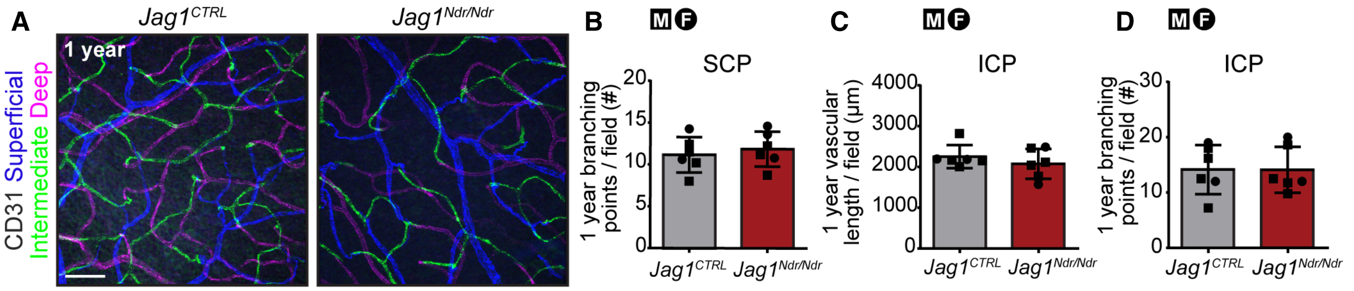

Figure EV5. Aging retina capillary network in 1-year-old mice.

A One-year-old retina three CD31+ capillary layers. Scale bar 50 µm.
B One-year-old SCP branching point number ($n = 6$ per group, unpaired t-test, $P = 0.5730$).
C One-year-old ICP vascular length ($n = 6$ per group, unpaired t-test, $P = 0.3686$).
D One-year-old ICP branching point number ($n = 6$ per group, unpaired t-test, $P = 0.9869$).

Data information: Bar graphs depict mean values ± standard deviation, each dot represents one biological replicate. Circles represent females, squares represent males. For details/results of statistical analyses, please see source data. ICP, intermediate capillary plexus; SCP, superficial capillary plexus.
Source data are available online for this figure.
