# Supplementary material for: Sex differences and risk factors for bleeding in Alagille syndrome
Source: EMBO Mol Med. 2022 Nov 8;14(12):e15809. doi: 10.15252/emmm.202215809 (PMC9728057; doi:10.15252/emmm.202215809)
Supplement: Supplementary file 14 [file EMMM-14-e15809-s009.pdf]

Sex differences and risk factors for bleeding in Alagille syndrome

Simona Hankeova, Noémi van Hul, Jakub Laznovsky, Elisabeth Verboven, Katrin Mangold, Naomi Hensens, Csaba Adori, Elvira Verhoef, Tomas Zikmund, Feven Dawit, Michaela Kavkova, Jakub Salplachta, Marika Sjoqvist, Bengt Johansson, Mohamed Hassan, Linda Fredriksson, Karsten Baumgartel, Vitezslav Bryja, Urban Lendahl, Andrew Jheon, Florian Alten, Kristina Fahnehjelm, Björn Fischler, Jozef Kaiser, and Emma Andersson
DOI: 10.15252/emmm.202215809

Corresponding author: Emma Andersson (Emma.Andersson@ki.se)

Review Timeline:

Transferred from Review Commons :	1st Feb 22
Editorial Decision:	4th Feb 22
Revision Received:	2nd Aug 22
Editorial Decision:	8th Sep 22
Revision Received:	27th Sep 22
Accepted:	5th Oct 22

Editor: Lise Roth

Transaction Report:

This manuscript was transferred to EMBO Molecular Medicine following peer review at Review Commons.

(Note: With the exception of the correction of typographical or spelling errors that could be a source of ambiguity, letters and reports are not edited. Depending on transfer agreements, referee reports obtained elsewhere may or may not be included in this compilation. Referee reports are anonymous unless the Referee chooses to sign their reports.)

Review #1

1. How much time do you estimate the authors will need to complete the suggested revisions:

Estimated time to Complete Revisions (Required)

(Decision Recommendation)

Cannot tell / Not applicable

2. Evidence, reproducibility and clarity:

Evidence, reproducibility and clarity (Required)

In manuscript RC-2021-01147, Hankeova et al. present their findings in regard to the potential risk factors that are associated with intracranial bleeding in patients suffering from a congenital disease called Alagille syndrome (ALGS) and the sexual dimorphism associated with some of ALGS vascular phenotypes. The authors first performed a systematic literature review for case studies of ALGS patients with vascular-related complications with an eye on potential gender-based differences, and found that a significantly higher number of idiopathic intracranial hemorrhage instances was reported in girls compared to boys affected with ALGS. The authors then used an ALGS model previously established by their group (Jag1Ndr/Ndr mutant mice) to further study the bleeding risk factors associated with reduced JAG1 function and their cellular mechanisms. They found that Jag1Ndr/Ndr animals showed an increase in the rate of spontaneous and provoked central nervous system bleeding. Despite cholestasis, the animals did not show abnormal coagulation. However, the mutant animals had thinner skulls and showed fewer and more tortuous blood vessels. One aspect of these vascular defects showed statistically significant sex-specific difference in this mouse model. Moreover, Jag1Ndr/Ndr animals displayed a reduction in the coverage of endothelial cells by vascular smooth muscle cells (VSMCs), a phenotype which was worsened by inducing hypertension in these animals for just two weeks. The authors also uncovered specific vascular abnormalities in the retinas of the mutant mice accompanied by an age-dependent reduction in the coverage of the retina by retinal ganglion cell axons. Finally, analysis of retinal fundus photographs indicated a significant increase in venous tortuosity in patient with ALGS compared to healthy controls and patients with another cholestatic disease.

Intracranial bleeding is a major cause of morbidity and mortality in patients with ALGS. However, the mechanisms and risk factors for these events are not well understood (perhaps other than the sensitivity of these patients to head trauma). Moreover, an animal model for ALGS vascular defects and hemorrhage in the CNS has not been described previously. The current manuscript therefore makes important contributions to the field by providing a link between idiopathic brain hemorrhage in ALGS patients and the patients' gender. Moreover, the vascular abnormalities described in Jag1Ndr/Ndr animals provide a model for future mechanistic and preclinical therapeutic studies on this understudied yet highly important aspects of ALGS. The rapid decline in the VSMC coverage of arteries in response to hypertension reported here in a mouse model of ALGS suggests that blood pressure should

be carefully controlled in patients with ALGS, especially females. Finally, the study offers a new non-invasive way to assess whether a given ALGS patient has vascular abnormalities, and motivates future studies to examine whether retinal vascular abnormalities have a predictive value for the risk of brain hemorrhage in these patients. In addition to these strengths, n my opinion, the paper has two major shortcomings. First, as explained below, the emphasis on the sex-difference of the phenotypes in mice is exaggerated, to the extent that it overshadows the value of establishing a mouse model for ALGS vascular defects for the first time (regardless of sexual dimorphism). Second, there has not been any attempts to provide a molecular mechanisms for the observed phenotypes. However, given the enormous amount of work that has already gone into this manuscript and its potential for future preclinical studies, I don't think mechanistic studies should be considered a requirement for the publication of this work.

Major points

It seems that the only statistically significant sex-specific difference in the mouse analysis is the number of venules in the retina. In contrast, the phenotypes that would potentially increase the risk of bleeding (like skull thickness) are not shown to be sexually dimorphic, and neither is brain bleeding itself (the gender of the 4 animals without bleeding in Figure 4I is not mentioned). In fact, for skull thickness the authors have almost exclusively presented data from males. However, in the Abstract and elsewhere in the manuscript, the way the authors use "sex-specific defects" in a number of sentences implies a much broader sexual dimorphism in the mouse. For example, the sentence "Jag1Ndr/Ndr mice exhibited sex-specific defects with sporadic bleeds, a thin skull, tortuous blood vessels ..." implies that the phenotypes listed after "with" might show a difference between males and females. The title of the paper starts with "Sex differences", but only one phenotype in the ALGS mouse model is sex-specific. The easiest way to address this issue would be to carefully review the text and explicitly mention the only sex-specific phenotype in these instances, followed by other phenotypes that are not sex-specific. The alternative would be to perform statistical analysis separately on males and females for other panels, similar to what is shown in Figure 3B-D. Did the postnatal lethality shown in Figure 2A occur similarly for both males and females? I understand that the focus on sex difference might have arisen after some of the data were generated. However, given the emphasis of the story on this issue, either additional data needs to be added or the claims need to be toned down.

Lines 139-142, description of Figure 2M: Since the Evans blue staining did not show a statistically significant increase in mutants, the authors should remove the sentence "... had mildly increased Evans blue leakage ...".

The way the authors have compared CADASIL and ALGS in the Discussion can be misleading to readers who are not Notch pathway experts. Although both CADASIL mouse models and the ALGS model used in this study show abnormalities in VSMCs, the mechanisms for such abnormalities are likely to be quite different (toxic gain-of-function versus haploinsufficiency). Similarly, talking about Notch3 mutant and CADASIL in the same sentence without further explanation and also using the phrase "defective NOTCH3 in CADASIL" (line 305) would imply to non-experts that loss of NOTCH3 signaling is a major issue in CADASIL. The authors should clarify these issues in the manuscript.

Throughout the manuscript various mouse ages have been used in experiments, between P10 to one year of age. However, it is not clear why those ages were chosen for each experiment.

For example, RGC problems studied in figure 5N and 5O (P10 and P40) are suggested to result from vascular defects. However, the vascular defects in these retinas were analyzed between 3-12 months of age.

Minor comments:

The authors suggest in the Discussion that the vascular tortuosity observed in ALGS arises from defects in ECs not from VSMCs. Given the difference in the disease mechanisms of ALGS and CADASIL and the potential crosstalk between these two cell types, this conclusion is rather speculative. Analyzing the expression of Notch pathway targets in these two cell types might have provided some clue into the cell type responsible for the observed phenotypes.

Please add some text to the Results section to further explain the data shown in Figure S3. In the current version, I think only one short sentence is used to describe this figure.

Lines 165-166, "an effect that was driven by significantly increased tortuosity specifically in female Jag1Ndr/Ndr mice at P30": This cannot be concluded from the presented data in figure 3G, as the statistics was performed for all animals combined, not just the females. Please rephrase.

What is the purple staining in Figure 2P?

Line 52: Alagille syndrome (ALGS) is 'a' pediatric disorder ...

Line 53: 'facies' is used more commonly than 'faces'.

Line 199: Are the authors discussing the aneurysm in 4B? 4G does not show an image.

Line 229: 'Fig. 5J' instead of 'Fig. 6J'.

Line 289: 'mouse' instead of 'moude'.

Line 290: 'venous' instead of 'evenous'.

Line 717: 'Fig. 5' instead of 'Fig. 6'.

3. Significance:

Significance (Required)

Please see above.

Review #2

1. How much time do you estimate the authors will need to complete the suggested revisions:

Estimated time to Complete Revisions (Required)

(Decision Recommendation)

Between 3 and 6 months

2. Evidence, reproducibility and clarity:

Evidence, reproducibility and clarity (Required)

Emma Andersson and colleagues report about Allagille syndrome, a rare congenital disorder affecting blood vessels and bile ducts. The responsible mutations are frequently found in the JAG1 gene, which is a Notch ligand. Some patients have NOTCH2 gene mutations. As such, it is a disorder due to impaired Notch signaling. While the disease has been characterized quite well in recent years there is still little known about sex-specific differences in the phenotype.
Andersson et al., have recently published a very interesting mouse model for Alagille syndrome which contains a Jag1 H268Q missense mutation (Gastroenterology, 2018 Mar;154(4):1080-1095). These mice were called Jag1+/Ndr mice. However, so far mostly the liver phenotype was studied in these mice and not so much the vascular phenotype which most likely plays a major role in the pathogenesis of this disease.

The paper is well written, however sometimes one might have the feeling that it could be worthwhile to split it in two manuscripts. The images are of high quality. Materials and Methods are detailed. Introduction and Discussion are interesting and well balanced.

Otherwise I have only few comments:

1)The number of analyzed Ndr/Ndr mice n=17 is a bit low, especially as some of the defects and brain bleedings occur at rather low frequency e.g. 1/17.

2)Analysis of vascular permeability was only done with Evan's blue. This showed that there is no gross defect. However, this assay is not specific enough to detect slight disturbance of vessel permeability in particular also at the blood brain barrier. The Betsholtz group established similar assays using low molecular weight tracers and it is strongly recommended to extent this investigation.

3)The VSMC phenotype is very interesting and somehow fits to other Notch-related pathologies like CADASIL. It would be very interesting to see if also the cellular phenotype of VSMCs changes in terms of plastic vs. contractile.

4)The authors claim that there are strong sex-specific differences in the Ndr/Ndr mice. Indeed, some of the experiments indicate in this direction. However, a thorough investigation of all phenotypes including bleeding and statistical testing is missing. Moreover, a table to

give an overview about this topic would be helpful.

3. Significance:

Significance (Required)

Here the authors report two major findings: 1) there are substantial sex-specific differences in Alagille patients in terms of idiopathic intracranial hemorrhage, one of the major cause for death in these young patients. This is a very interesting finding. 2) The authors further characterize their Jag1 Ndr mice. Now as homozygous Ndr/Ndr and with much greater and detailed focus on the vascular phenotype. This gives a number of interesting findings which validate this transgenic mouse as an excellent Alagille model system. In addition, the authors report that also in mice they observed some sex-specific differences in the vascular phenotype.
The strengths of the manuscript are obvious: the sex-specific differences are very interesting and will inspire future research. Secondly, the characterization of the Jag1 Ndr/Ndr mice show that this is an excellent Alagille model system.
The weakness of this paper is that we do not learn about any new mechanistic insight. Neither a potential reason for the sex-specific differences not in the vascular pathogenesis.

Review #3

1. How much time do you estimate the authors will need to complete the suggested revisions:

Estimated time to Complete Revisions (Required)

(Decision Recommendation)

Cannot tell / Not applicable

2. Evidence, reproducibility and clarity:

Evidence, reproducibility and clarity (Required)

In this paper, authors aimed at identifying risk factors of cerebral bleeding in the Alagille syndrome. They first performed a literature review of cerebral bleedings in patients with Alagille syndrome (ALGS). They then screened a mouse model of ALGS for cerebral bleeding and vascular defects. They further analyzed the retina of patients with ALGS and or CADASIL, looking for vascular defects. The authors conclude that retinal vessel abnormalities (arteriovenous crossings, reduction in blood vessel numbers and increase vessel tortuosity) are risk factors of cerebral bleeding in ALGS patients that can be detected non-invasively by looking at the retinal fundus.

Unfortunately, this conclusion is not supported by the results. First, characterization of the bleeding phenotype in the ALGS mouse model is extremely rudimentary and abnormalities of cerebral vessels underlying cerebral hemorrhage have not been identified. Second, at no time did the authors show any correlation between arteriovenous crossings, reduction in blood vessel numbers and increase vessel tortuosity in the retina and the occurrence of cerebral bleeding. Third, the observation that these abnormalities are present in the vast majority of mutant mice whereas less than 5% of the mutant mice develop cerebral bleedings belies their conclusion. Another major problem with this paper is that many statements are not supported by experimental data or statistical analysis.

Other major concerns

Abstract and introduction: what is the rationale to look for a gender effect in the occurrence of cerebral bleeding in patients with ALGS? I could not find any explanation in the introduction or in the literature.
Results, first section and Figure 1: The classification of cerebral bleedings is not appropriate and all the most surprising: hematoma and hemorrhages are used in clinics to design the same thing; idiopathic means with an unknown mechanism, therefore a subarachnoid hemorrhage caused by the rupture of an aneurysm cannot be classified as "idiopathic". Conversely, a minor trauma can be a triggering factor but cannot be considered as the cause of a hemorrhage.
Results, second section (lines 113-151) and Figure 2: Characterization of the bleeding phenotype in the ALGS mouse model is extremely rudimentary. Whereas cerebral hemorrhages in patients with ALGS can occur in the adult, only pups from P0 to P10 were analyzed. Screening was limited to a macroscopic analysis of the brain which is unlikely to capture intracerebral hemorrhages and as a result the true prevalence of cerebral bleeding is probably underestimated. More importantly, the origin and the cause of cerebral bleeding were not investigated. Moreover, it is said that Jagged 1 mutant mice exhibit "provoked hemorrhage". I could not find the experimental data supporting this statement. Also, the statement that "thinner skulls in Jag1ndr/ndr mice likely contributed to nervous system bleeds" is not supported by any experimental data.

Results, fourth section, figure 4 and supplementary fig 4. It is said that pericytes in the retinal capillaries were not reduced but supplementary fig 4 does not show any quantification. It is said that VSMC were less mature in mutant mice, please explain. The finding that Jag1ndr/ndr mice display reduced VSMC coverage is not entirely new given that the ndr mutation is an hypomorphic mutation and that mural cell coverage has been reported to be reduced in Jagged1 KO mice (Benedito et al, Cell 2009). The statement that gaps in VSMC coverage are exacerbated by aging are not supported by quantifications. In the AngII experiment (panel H), blood pressure measurements are not shown. By the way, pooling mice with age ranging from 3 to 7.5 months in a mouse model with an age-dependent phenotype seems counterintuitive.
Results, fifth section, Figure 5 (mislabeled figure 6). It is almost counterintuitive to have a reduced density in the ICP and a lower number of vertical sprouts in 3-6 months old mice and a normal density at 1 year. Again, the statement that the RGC axons are healthy in Jag1Ndr/Ndr retinas at P10 is not supported by quantification. Also, the authors show a reduction in the number of RGC axons at P40. Whereas they document VSMC degeneration much later, they claim that the onset of vascular degeneration was associated with RGC

degeneration.

Results, sixth section, figure 6. The rationale to include in the analysis of the retinal fundus CADASIL patients is far from obvious to this Reviewer and does not bring anything. Indeed, whereas ALGS is a developmental disease, CADASIL is a degenerative disease. Moreover, patients with AGLS exhibit cerebral bleeding, whereas cerebral bleeding in patients with CADASIL is extremely rare.

3. Significance:

Significance (Required)

The Alagille syndrome is a developmental disease primarily affecting the liver, caused by hypomorphic or loss of function in the Jagged1 ligand or more rarely in the Notch2 receptor. Nevertheless, cerebral hemorrhage in patients with ALGS is of clinical significance, causing up to 25% of deaths. Because the penetrance of these hemorrhages is incomplete, identifying risk factors would constitute a significant advance.
Unfortunately, retinal vessel abnormalities (arteriovenous crossings, reduction in blood vessel numbers and increase vessel tortuosity) identified in patients or mice with ALGS in this study are unlikely to be risk factors for cerebral hemorrhage for the reasons mentioned in the previous section.

Expertise of this Reviewer encompasses clinical neurology, the physiology and pathology of the cerebroretinal vasculature and the Notch signaling pathway.

Revision Plan

Manuscript number: RC-2021-01147
Corresponding author(s): Emma R Andersson

1. General Statements

We thank the reviewers for their constructive assessment of our work. In general, the comments are high quality, and we believe that addressing these comments will help strengthen the manuscript and provide further important insights. Some of the reviewer feedback refers to allegedly missing statistical analyses or sex information that was actually provided in the manuscript and, below, we include the point-by-point response to highlight which comments require new analyses/new experiments, and which can be addressed by restructuring the manuscript or re-labelling of graphs.

2. Description of the planned revisions

There are 12 main experiments (or sets of experiments) planned to address the reviewer comments. These are first listed here, followed by a point-by-point response to the Reviewers to put these experiments in context.

2.1 **DONE: Quantification of ASMA gaps per age:** To validate our statement that ASMA gaps are an age-dependent phenotype (that worsen with age), we quantified ASMA gaps at P30, 3-6 months and 1 year of age (data in full response below). The analyses confirm that no gaps are present in wild types at any age, nor in Jag1Ndr/Ndr mice at P30. By 1 year, 50% of *Jag1*$^{Ndr/Ndr}$ mice display large gaps in ASMA coverage. These data support our claim that gaps in VSMC coverage are exacerbated by aging.

2.2 **Done: Verification of increased blood pressure in AngII treated-mice:** This analysis is done, and data are presented in the full response below.

2.3 **Quantification of CD13 pericyte coverage:** We quantified CD13 coverage in the mice reported in Supplementary Fig 4 (data in full response below). Because there is a tendency towards reduced coverage in females, we will add 1-3 n per sex and genotype to allow correct statistical testing of these results. **Preliminary data in full response below.**

2.4 **Skull thickness analysis by sex**: Skull analysis by microCT in male and female *Jag1*$^{Ndr/Ndr}$ mice and matching wild types at P30 to determine if genotype interacts with sex to regulate skull thickness. N 4- 6 per sex and genotype).

2.5 **Survival analysis by sex**: Analysis of animal survival per sex to determine whether survival is sex-biased. Currently the data in Fig 2A include 8 *Jag1*$^{Ndr/Ndr}$ males and 8 *Jag1*$^{Ndr/Ndr}$ females. We will add 4 males and females to this dataset and perform survival analyses.

2.6 **Brain vessel permeability assay**: We will assess blood brain barrier permeability using 1kDa Cadaverin 555, 3 kDa Dextran-FITC or -TMR, and/or 10kDa Dextran-FITC at P30 in male and female *Jag1*$^{Ndr/Ndr}$ mice (n of 6 for each). Mice are injected with these fluorescent tracers, which are permitted to circulate. Mice are then sacrificed, and the brain is dissected out. We will image the overall brain fluorescence and/or in brain sections from one half of the brain. The other brain half will be homogenized and fluorescence will be quantified in a fluorescence spectrometer.

2.7 **Correlation of vascular defects and bleeding**: For the mice in the experiment above (brain vessel permeability assay), we will dissect out retinas and analyze retinal architecture (vascular tortuosity and blood vessel number) and mural cell coverage (CD13 and ASMA staining). N of 6 for each, matched to the experiment above.

2.8 **Brain vessel characterization**: We will stain for Glut1, Asma, IgG, ColIV, and/or CD31 at P10 and/or P30 to assess whether intracranial vessels display increased tortuosity, architectural defects, decreased vessel density or reduced VSMC coverage. N of 3 per sex and genotype.

2.9 **Synthetic vs contractile phenotype of VSMCs**: We analyze aorta and retina from male and female *Jag1*$^{Ndr/Ndr}$ P30 mice, using staining for contractile and synthetic VSMCs (for example Vimentin, ColI, Tpm4 as synthetic markers and Sm22, Myh11 as contractile markers).

2.10 **Quantification of retinal ganglion cell (RGC) coverage at P10**: We will quantify RGC coverage at P10 to verify that RGCs are initially normal and degrade by P40 (P40 was quantified and presented in the manuscript).

2.11 **Quantification of capillary bed integrity at P30**: To test whether the capillary bed breakdown occurs before the RGC breakdown at P40, we will analyze the superior, intermediate and deep capillary plexus at P30 (SCP/ICP/DCP). Analyses will be matched to figure 5 (Capillary homeostasis figure) with staining for CD31 and quantification of vascular length and branching points in the SCP and ICP.

2.12 **Novelty and comparison with other models:** We have begun analysis of vasculature in *Jag1*$^{\Delta DSL/+}$ mice, an alternative model for Alagille syndrome which recapitulates aspects of liver disease. Our preliminary analyses show *Jag1*$^{\Delta DSL/+}$mice display a similar phenotype to *Jag1*$^{Ndr/Ndr}$ mice with fewer arteries and veins and display arteriovenous crossings. *Jag1*$^{\Delta DSL/+}$also display overall weaker ASMA staining, but no small or large gaps between VSMCs. The *Jag1*$^{\Delta DSL/+}$mice thus exhibit a milder phenotype than *Jag1*$^{Ndr/Ndr}$ mice. Currently this analysis is based on three circa P30 mice, and two circa one year old mice. We aim to complete the analysis with n=6 at each stage.

In addition to these planned experiments, we will extensively revise the text and generate a summary table for the statistical comparisons, as requested.

Point-by-point response:

Reviewer #1 (Evidence, reproducibility and clarity (Required)):

In manuscript RC-2021-01147, Hankeova et al. present their findings in regard to the potential risk factors that are associated with intracranial bleeding in patients suffering from a congenital disease called Alagille syndrome (ALGS) and the sexual dimorphism associated with some of ALGS vascular phenotypes. The authors first performed a systematic literature review for case studies of ALGS patients with vascular-related complications with an eye on potential gender-based differences, and found that a significantly higher number of idiopathic intracranial hemorrhage instances was reported in girls compared to boys affected with ALGS. The authors then used an ALGS model previously established by their group (Jag1Ndr/Ndr mutant mice) to further study the bleeding risk factors associated with reduced JAG1 function and their cellular mechanisms. They found that Jag1Ndr/Ndr animals showed an increase in the rate of spontaneous and provoked central nervous system bleeding. Despite cholestasis, the animals did not show abnormal coagulation. However, the mutant animals had thinner skulls and showed fewer and more tortuous blood vessels. One aspect of these vascular defects showed statistically significant sex-specific difference in this mouse model. Moreover, Jag1Ndr/Ndr animals displayed a reduction in the coverage of endothelial cells by vascular smooth muscle cells (VSMCs), a phenotype which was worsened by inducing hypertension in these animals for just two weeks. The authors also uncovered specific vascular abnormalities in the retinas of the mutant mice accompanied by an age-dependent reduction in the coverage of the retina by retinal ganglion cell axons. Finally, analysis of retinal fundus photographs indicated a significant increase in venous tortuosity in patient with ALGS compared to healthy controls and patients with another cholestatic disease. Intracranial bleeding is a major cause of morbidity and mortality in patients with ALGS. However, the mechanisms and risk factors for these events are not well understood (perhaps other than the sensitivity of these patients to head trauma). Moreover, an animal model for ALGS vascular defects and hemorrhage in the CNS has not been described previously. The current manuscript therefore makes important contributions to the field by providing a link between idiopathic brain hemorrhage in ALGS patients and the patients' gender. Moreover, the vascular abnormalities described in Jag1Ndr/Ndr animals provide a model for future mechanistic and preclinical therapeutic studies on this understudied yet highly important aspects of ALGS. The rapid decline in the VSMC coverage of arteries in response to hypertension reported here in a mouse model of ALGS suggests that blood pressure should be carefully controlled in patients with ALGS, especially females. Finally, the study offers a new

non-invasive way to assess whether a given ALGS patient has vascular abnormalities, and motivates future studies to examine whether retinal vascular abnormalities have a predictive value for the risk of brain hemorrhage in these patients. In addition to these strengths, in my opinion, the paper has two major shortcomings. First, as explained below, the emphasis on the sex-difference of the phenotypes in mice is exaggerated, to the extent that it overshadows the value of establishing a mouse model for ALGS vascular defects for the first time (regardless of sexual dimorphism). Second, there has not been any attempts to provide a molecular mechanisms for the observed phenotypes. However, given the enormous amount of work that has already gone into this manuscript and its potential for future preclinical studies, I don't think mechanistic studies should be considered a requirement for the publication of this work.

It is a pleasure to read your summary of our work, and we thank you for explicitly identifying strengths and weaknesses of the manuscript. While we consider the sexual dimorphism interesting and important, we agree that this does not need to be the focus of the manuscript and have/will therefore re-orient the manuscript to focus on the establishment of the *Jag1*$^{Ndr/Ndr}$ mice as a mouse model for vascular defects, and that this model predicted observable phenotypes in human retinas. We will, however, also expand upon two experiments to determine whether there is more sexual dimorphism than the two phenotypes we identified (venule number and venous tortuosity). Based on our new results, we would amend the title of the manuscript to reflect the main focus of the research findings.

Major points
1) It seems that the only statistically significant sex-specific difference in the mouse analysis is the number of venules in the retina.

There were two significantly different sex-dimorphic phenotypes in the Jag1Ndr/Ndr mice: number of venules (former Fig 3D), and P30 venous tortuosity (former Fig 3G). Although this was stated in the text and statistical testing was explicitly descried in the figure legend, the figure panel itself did not make clear that P30 venule tortuosity was statistically tested for impact of sex. We will add labelling to this panel to visualize this significant difference. Our preliminary analysis of CD13+ pericyte coverage also suggests a sex-dimorphic phenotype, this will be completed with additional male and female replicates of each genotype.

2) In contrast, the phenotypes that would potentially increase the risk of bleeding (like skull thickness) are not shown to be sexually dimorphic, and neither is brain bleeding itself (the gender of the 4 animals without bleeding in Figure 4I is not mentioned). In fact, for skull thickness the authors have almost exclusively presented data from males. However, in the Abstract and elsewhere in the manuscript, the way the authors use "sex-specific defects" in a number of sentences implies a much broader sexual dimorphism in the mouse. For example,

the sentence "Jag1Ndr/Ndr mice exhibited sex-specific defects with sporadic bleeds, a thin skull, tortuous blood vessels ..." implies that the phenotypes listed after "with" might show a difference between males and females. The title of the paper starts with "Sex differences", but only one phenotype in the ALGS mouse model is sex-specific. The easiest way to address this issue would be to carefully review the text and explicitly mention the only sex-specific phenotype in these instances, followed by other phenotypes that are not sex-specific. The alternative would be to perform statistical analysis separately on males and females for other panels, similar to what is shown in Figure 3B-D.

As we described in the supplemental Materials and Methods, experiments generally include mice of both male and female sex. Experiments in which the results suggested sex differences were expanded with additional mice of each sex to determine whether sex differences were present. The reviewer is absolutely correct that the skull analysis is an exception to this generalization. We will therefore scan and analyze new skulls from male and female wild type and *Jag1*$^{Ndr/Ndr}$ mice (n of 4-6 per sex and genotype). To ensure that the results are comparable we will use the same stage for all mice, which will be P30, as it will not be possible to obtain new mice of 7-8 months of age within 3 months.

The reviewer states that the sex of the animals without bleeding in Fig. 4I are not mentioned, but these are the same as animals and in Fig. 4J and 4K and are explicitly labelled as two females and three males.

As stated above, there were two sex-dimorphic phenotypes in the Jag1Ndr/Ndr mice, and will test whether there are others.

The discovery that females are over-represented when it comes to spontaneous intracranial bleeds in Alagille syndrome is an important and novel sex difference.

We will go through the text and ensure that the conjunction “with” is not used in the fashion the reviewer pointed out, as we agree this is misleading and was not our intention. We will be explicit about which features are dimorphic and which are not.

3) Did the postnatal lethality shown in Figure 2A occur similarly for both males and females?

This is an excellent question, and we thank the reviewer for asking. We initially did not separate the data for this analysis because we considered the sample size underpowered to address survival (dataset is eight males and eight females). We will collect additional survival data from all experiments included in this revision plan to expand this dataset and answer this question. However, the mice also have cardiac defects and severe cholestasis which may negatively impact survival, and confound interpretation.

4) I understand that the focus on sex difference might have arisen after some of the data were generated. However, given the emphasis of the story on this issue, either additional data needs to be added or the claims need to be toned down.

We will include additional data (complete sex- and age-matched sets of skull microCT, expanded survival analysis, increased number of replicates for quantification of CD13 coverage) to determine whether any of these is sexually dimorphic. Based on the results of these analyses, we will stringently edit the text to ensure that there are no overstatements, and that the text reflects the new results.

5) Lines 139-142, description of Figure 2M: Since the Evans blue staining did not show a statistically significant increase in mutants, the authors should remove the sentence "... had mildly increased Evans blue leakage ...".

We will omit this phrase. We will also perform new experiments testing leakage of Dextrans, as described below in a response to Reviewer 2. Based on the results of these experiments, we will modify the text accordingly.

6) The way the authors have compared CADASIL and ALGS in the Discussion can be misleading to readers who are not Notch pathway experts. Although both CADASIL mouse models and the ALGS model used in this study show abnormalities in VSMCs, the mechanisms for such abnormalities are likely to be quite different (toxic gain-of-function versus haploinsufficiency). Similarly, talking about Notch3 mutant and CADASIL in the same sentence without further explanation and also using the phrase "defective NOTCH3 in CADASIL" (line 305) would imply to non-experts that loss of NOTCH3 signaling is a major issue in CADASIL. The authors should clarify these issues in the manuscript.

We used “"defective NOTCH3 in CADASIL" as the most careful way of referring to the NOTCH3 mutations in CADASIL, which defy strict definition. We agree that the current text is insufficient, with language shortcuts that arose due to a character count limit adjustment. These sections will be modified to clarify the difference between the mouse models and patients, toxic gain of function vs loss of function, and developmental vs pathological mechanisms.

7) Throughout the manuscript various mouse ages have been used in experiments, between P10 to one year of age. However, it is not clear why those ages were chosen for each experiment. For example, RGC problems studied in figure 5N and 5O (P10 and P40) are suggested to result from vascular defects. However, the vascular defects in these retinas were analyzed between 3-12 months of age.

We apologize if the choice of stages was unclear and are grateful that this was pointed out so that it can be rectified. In general terms, we have chosen to study developing (P5-P15), adult (3- 6 months) and older mice at the onset of senescence/middle age (one year). The capillary plexus phenotype was investigated in 3-6 month and 1 year old mice since the plexuses are well established and possible to visualize at these stages, and the analysis of the P5-P15 superficial capillary plexus (in Supplementary Fig 3) had shown that the plexus was normal at P10. We realize now that the P10 RGC data was not well explained in the context of the capillary plexus data presented in Supplementary Fig 3. This will be rectified in the revised version. Furthermore, we will stain and analyze new retinas from P30 mice to determine whether the intermediate capillary plexus is well established at this intermediate stage (CD31, ASMA, ColIV staining (n=3 per sex and genotype)).

Minor comments:
8) The authors suggest in the Discussion that the vascular tortuosity observed in ALGS arises from defects in ECs not from VSMCs. Given the difference in the disease mechanisms of ALGS and CADASIL and the potential crosstalk between these two cell types, this conclusion is rather speculative. Analyzing the expression of Notch pathway targets in these two cell types might have provided some clue into the cell type responsible for the observed phenotypes.

Yes, we agree that Notch target gene analysis in these cell types would have been informative. We had therefore performed three scRNA seq experiments to address this question: Smart Seq2 of whole retina, endothelial cell antibody-enriched and neural cell-depleted endothelial cells and sorted VE-Cad tomato-expressing endothelial cells. However, all three experiments yielded too many cells of the wrong cell type, and we were thus unable to address this interesting question. For this reason, we do not address the mechanisms of tortuosity in the Results section, but have reserved this topic for the Discussion, which we think should be a place for speculation. We will clarify in the text that this hypothesis should be addressed with experiments in the future.

9) Please add some text to the Results section to further explain the data shown in Figure S3. In the current version, I think only one short sentence is used to describe this figure.

Indeed, because these data were not novel, and due to character count limits, we had chosen to state only that vascular development in $Jag1^{Ndr/Ndr}$ mice phenocopies the *Jag1* EC knockout. We will modify and expand this text section to explicitly describe the results.

10) Lines 165-166, "an effect that was driven by significantly increased tortuosity specifically in female Jag1Ndr/Ndr mice at P30": This cannot be concluded from the presented data in

figure 3G, as the statistics was performed for all animals combined, not just the females. Please rephrase.

This is a misunderstanding, statistical analysis was done on separate sexes, as described in the figure legend. "(*Jag1*^{CTRL} n=6, *Jag1*$^{Ndr/Ndr}$ n=8, unpaired t test, **P=0.0085, and One-way ANOVA, **P=0.0013, followed by Tukey's multiple comparisons test to test for sex effect, *Jag1*^{CTRL} vs *Jag1*$^{Ndr/Ndr}$ females ***P=0.001, *Jag1*^{CTRL} vs *Jag1*$^{Ndr/Ndr}$ males ns, P=0.2320, *Jag1*$^{Ndr/Ndr}$ females vs *Jag1*$^{Ndr/Ndr}$ males *P=0.0286). This will also be clarified in the figure with explicit labelling of the statistically significantly different *Jag1*$^{Ndr/Ndr}$ males and females, with a star.

11) What is the purple staining in Figure 2P?
Thank you for noting this, a panel label disappeared during formatting. The purple staining is ASMA and will be labelled in the figure.

12) Line 52: Alagille syndrome (ALGS) is 'a' pediatric disorder ...
Line 53: 'facies' is used more commonly than 'faces'.
Line 199: Are the authors discussing the aneurysm in 4B? 4G does not show an image.
Line 229: 'Fig. 5J' instead of 'Fig. 6J'.
Line 289: 'mouse' instead of 'moude'.
Line 290: 'venous' instead of 'evenous'.
Line 717: 'Fig. 5' instead of 'Fig. 6'.

Thank you for the helpful text and figure call-out corrections, very much appreciated.

Reviewer #1 (Significance (Required)):
Please see above.

Reviewer #2 (Evidence, reproducibility and clarity (Required)):

Emma Andersson and colleagues report about Allagille syndrome, a rare congenital disorder affecting blood vessels and bile ducts. The responsible mutations are frequently found in the JAG1 gene, which is a Notch ligand. Some patients have NOTCH2 gene mutations. As such, it is a disorder due to impaired Notch signaling. While the disease has been characterized quite well in recent years there is still little known about sex-specific differences in the phenotype. Andersson et al., have recently published a very interesting mouse model for Alagille syndrome which contains a Jag1 H268Q missense mutation (Gastroenterology, 2018

Mar;154(4):1080-1095). These mice were called Jag1+/Ndr mice. However, so far mostly the liver phenotype was studied in these mice and not so much the vascular phenotype which most likely plays a major role in the pathogenesis of this disease.

We thank the reviewer for referring to our previous work and identifying the novel aspects of this manuscript. Indeed, a model for vascular defects in Alagille syndrome has not yet been described at all.

The paper is well written, however sometimes one might have the feeling that it could be worthwhile to split it in two manuscripts. The images are of high quality. Materials and Methods are detailed. Introduction and Discussion are interesting and well balanced.

We thank the reviewer for the positive comments on the structure of the manuscript and the quality of the images. We agree that it is a large manuscript, and indeed discussed at length whether to prepare one or two manuscripts. We opted for one large manuscript in order to be able to report and integrate the discussion and analysis of both endothelial and vascular smooth muscle cell phenotypes.

Otherwise I have only few comments:

1)The number of analyzed Ndr/Ndr mice n=17 is a bit low, especially as some of the defects and brain bleedings occur at rather low frequency e.g. 1/17.

We apologize for the misunderstanding – the brain bleeds occurred in 2 of 53 *Jag1*$^{Ndr/Ndr}$ mice (Previously stated on page 6: "Fifty-three *Jag1*$^{Ndr/Ndr}$ pups were monitored daily from birth until P10 (including the 16 described above) and macroscopically obvious brain hemorrhages occurred in two *Jag1*$^{Ndr/Ndr}$ pups (…)"). The figure will be re-ordered and this section will be rewritten to clarify the numbers of mice analyzed.

2) Analysis of vascular permeability was only done with Evan's blue. This showed that there is no gross defect. However, this assay is not specific enough to detect slight disturbance of vessel permeability in particular also at the blood brain barrier. The Betsholtz group established similar assays using low molecular weight tracers and it is strongly recommended to extent this investigation.

We agree this would be an important experiment. We will assess blood brain barrier permeability using 1kDa Cadaverin 555, 3 kDa Dextran-FITC or -TMR, and/or 10kDa Dextran-FITC at P30. We have performed pilot experiments to assess delivery and detection. Both 3kDa

and 10kDa are well delivered to wild type mice by tail vein injection and are easily detectable by overall fluorescence in kidney (please see adjacent Fig 1). We measured fluorescence in serum using a fluorescence spectrometer and could distinguish injected and non-injected samples.

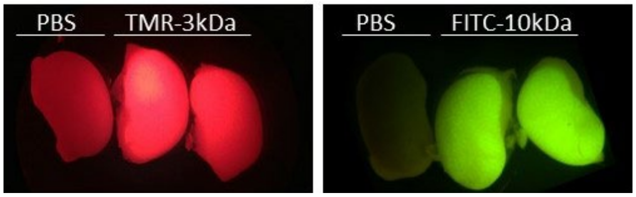

Fig 1. Our pilot experiments show that fluorescent dextrans are detectable in kidney in injected adult wild type mice (as described by others). These fluorescent dextrans (as well as 1kDa Cadaverin 555) will be used to assess vascular leakage in *Jag1*$^{Ndr/Ndr}$ mice.

For these experiments, we will inject wild type and *Jag1*$^{Ndr/Ndr}$ mice at P30 with the fluorescent tracers described above, allow the tracers to circulate and then sacrifice the mice for analysis. We will dissect out the brain and image the overall brain fluorescence and/or in brain sections. We will use one brain half for homogenization and quantification of fluorescence in a fluorescence spectrometer.

3)The VSMC phenotype is very interesting and somehow fits to other Notch-related pathologies like CADASIL. It would be very interesting to see if also the cellular phenotype of VSMCs changes in terms of plastic vs. contractile.

Thank you for the positive words, we agree this would be interesting. We have begun collecting aorta and retina from P30 mice and will analyze expression of contractile and synthetic VSMCs (for example Vimentin, ColI, Tpm4 as synthetic markers and Sm22, Myh11 as contractile markers).

4)The authors claim that there are strong sex-specific differences in the Ndr/Ndr mice. Indeed, some of the experiments indicate in this direction. However, a thorough investigation of all phenotypes including bleeding and statistical testing is missing. Moreover, a table to give an overview about this topic would be helpful.

Statistical testing was done as described in the Materials and Methods: "Experiments generally include mice of both male and female sex. Experiments in which the results suggested sex differences were expanded with additional mice of each sex to determine whether sex differences were present." There were two statistically phenotypes for which sex was a modifier of phenotype: venule number (fewer in *Jag1*$^{Ndr/Ndr}$ females than *Jag1*$^{Ndr/Ndr}$ males) and P30 venule tortuosity (more tortuous in *Jag1*$^{Ndr/Ndr}$ females than *Jag1*$^{Ndr/Ndr}$ males). See also point 10 Reviewer 1 for further explanation. These statistics were reported in the Figure legends, and we appreciate that a summary analysis would be helpful to the reader. We will collect all statistical analyses in one Supplementary Table to provide an easy overview.

Revision Plan

Reviewer #2 (Significance (Required)):

Here the authors report two major findings: 1) there are substantial sex-specific differences in Alagille patients in terms of idiopathic intracranial hemorrhage, one of the major cause for death in these young patients. This is a very interesting finding. 2) The authors further characterize their Jag1 Ndr mice. Now as homozygous Ndr/Ndr and with much greater and detailed focus on the vascular phenotype. This gives a number of interesting findings which validate this transgenic mouse as an excellent Alagille model system. In addition, the authors report that also in mice they observed some sex-specific differences in the vascular phenotype.

The strengths of the manuscript are obvious: the sex-specific differences are very interesting and will inspire future research. Secondly, the characterization of the Jag1 Ndr/Ndr mice show that this is an excellent Alagille model system.

The weakness of this paper is that we do not learn about any new mechanistic insight. Neither a potential reason for the sex-specific differences not in the vascular pathogenesis.

We thank the reviewer for identifying the strengths and weaknesses of the manuscript. We agree that this manuscript focuses on reporting a new mouse model for disease, and we also show that it predicted phenotypes that could be observed in patient retinas non-invasively, which we hope will be clinically useful. As the reviewer also points out in their earlier comments, this manuscript could have been two manuscripts – there is a wealth of new insights, and we think this could be an important model for pre-clinical research.

Revision Plan

Reviewer #3 (Evidence, reproducibility and clarity (Required)):

In this paper, authors aimed at identifying risk factors of cerebral bleeding in the Alagille syndrome. They first performed a literature review of cerebral bleedings in patients with Alagille syndrome (ALGS). They then screened a mouse model of ALGS for cerebral bleeding and vascular defects. They further analyzed the retina of patients with ALGS and or CADASIL, looking for vascular defects. The authors conclude that retinal vessel abnormalities (arteriovenous crossings, reduction in blood vessel numbers and increase vessel tortuosity) are risk factors of cerebral bleeding in ALGS patients that can be detected non-invasively by looking at the retinal fundus.

We thank the reviewer for summarizing our work. By "risk factors" we were referring to skull thickness and coagulopathy, both of which have been implicated in brain hemorrhages in children, or female sex (which we report here as a potential risk factor). The endothelial cell defects and vascular smooth muscle cell defects that we report we consider to be the pathology itself, that may underlie the bleeding, but not technically a "risk factor". As such, the arteriovenous crossings, reduction in blood vessel numbers and increased vessel tortuosity could instead serve as biomarkers (but will have to be studied in larger cohorts including patients with bleeding events).

The literature review was a Systematic Literature Review, following PRISMA guidelines.

We will revise the text to clarify the difference between risk factors and potential biomarkers.

Unfortunately, this conclusion is not supported by the results. First, characterization of the bleeding phenotype in the ALGS mouse model is extremely rudimentary and abnormalities of cerebral vessels underlying cerebral hemorrhage have not been identified.

The neural retina is the state-of-the-art tissue for analysis of vascular development and homeostasis in the nervous system, as it can be flattened and systematically imaged and analyzed. To address whether defects identified in the retina are also present in the brain, we have collected brains from $Jag1^{+/+}$ and $Jag1^{Ndr/Ndr}$ mice to analyse brain vasculature. We will stain for Glut1, Asma, IgG, ColIV, and/or CD31 at P10 and/or at P30 to assess whether intracranial vessels display increased tortuosity, architectural defects, decreased vessel density or reduced VSMC coverage. The protocol takes ca one month and we therefore cannot present preliminary data, but the protocol works well for the liver and has been established in collaboration with Csaba Adori (Adori et al, Science Advances 2021).

Second, at no time did the authors show any correlation between arteriovenous crossings, reduction in blood vessel numbers and increase vessel tortuosity in the retina and the occurrence of cerebral bleeding.

The brain bleeds are observed in dead or dying mice, which would confound any analysis of blood vessels, since these may be degrading due to the death of the animal. Indeed, correlating bleeding events (observed in 4% of *Jag1*$^{Ndr/Ndr}$ mice, a genotype which survives to P10 only 50% of the time, and to adulthood only 25% of the time) would be extremely challenging. This question could be addressed in patients with non-invasive imaging of retina and follow-up of bleeding events, which we intend to pursue after this manuscript is published and such a plan can be justified to funders and ethical boards.

In order to address whether vascular compromise correlates with vascular pathology, we will perform matched analysis of retinal architecture (vascular tortuosity and blood vessel number) and mural cell coverage (CD13 and ASMA) versus dextran leakage in brain (experiment described under Point 2 to Reviewer 2).

Furthermore, this limitation will be acknowledged in the discussion.

Third, the observation that these abnormalities are present in the vast majority of mutant mice whereas less than 5% of the mutant mice develop cerebral bleedings belies their conclusion.

While we were able to identify a gross intracranial bleed in 2 of 53 mice, 75% of *Jag1*$^{Ndr/Ndr}$ mice die between P0 and 3 months (50% of which in the first 10 days, former Fig 2A, and Andersson et al Gastroenterology 2018). Because the mother often cannibalizes the young, and because many organ systems are disrupted in the mouse model (in particular cardiovascular defects and liver defects) it is not possible for us to determine cause of death for the majority of the mice. It is possible that vascular defects contribute to the death of the 75%. On the other hand, we agree that observable brain bleeds represent outliers, and we therefore made a point of describing data points for which there were outlier data in the *Jag1*$^{Ndr/Ndr}$ population that could be meaningful, e.g.

1. frail blood vessels: resin leakage in two of six mice in former Fig 2K, or
2. arterial tortuosity: "Arterial tortuosity was increased in three of six Jag1Ndr/Ndr mice at P30 (Fig 3E) and in one of six Jag1$^{Ndr/Ndr}$ mice at one year (Fig 3F), but the overall differences were not statistically significant."
3. Evans blue leakage: "Three adult Jag1Ndr/Ndr mice (one male, two females) of eight tested, had mildly increased Evan blue leakage in the brain compared to the wild type animals, but on average there was no difference (Fig. 2M)."

Revision Plan

The statement that the abnormalities are present in the majority of the homozygous mice does not take into account the spread of the data and relies instead on the interpretation of the summary statistical analysis. While two groups can be significantly different (for example with regards to venous tortuosity at P30, former Fig 3G), there is a non-negligible spread in the data for the Jag1Ndr/Ndr mice. Jag1+/+ mice exhibit a low venous tortuosity (range 1-2.5%) while Jag1Ndr/Ndr mice range from 3% - 10.5%. It is unlikely that 3% is biologically meaningfully different from 2.5%, but the one Jag1Ndr/Ndr mouse with 10.5% tortuosity has four-fold longer-than-necessary vasculature than the longest Jag1+/+ mouse.

To clarify these differences in how to weigh and assess the individual data points vs the statistically significantly different groups, we will include a paragraph in discussion to address the data spread, statistical analysis, and outlier mice.

Another major problem with this paper is that many statements are not supported by experimental data or statistical analysis.

Every quantification and resulting graph was analyzed for Statistical significance, using stringent statistical analysis, as described in the Materials and Methods and explicitly described in each figure legend (both for significant and non-significant differences).

To facilitate for the reader, we will collect the statistical analyses in a Supplementary Table to enable easy comparisons and a quick overview.

We will check the text to identify and remove any statements that are not supported by data.

Other major concerns
Abstract and introduction: what is the rationale to look for a gender effect in the occurrence of cerebral bleeding in patients with ALGS? I could not find any explanation in the introduction or in the literature.

Thank you for pointing out this omission. In editing to reduce character count, the rationale was only explained in the Discussion ("(subarachnoid hemorrhage/SAH) … (is).. more common in men between age 25-45, and in women between age 55-85. The greater risk of SAH in women over 55 has been speculated to relate to hormonal differences after menopause, but hormone replacement therapy has yielded mixed results. Patients with ALGS may also present with hormonal differences, which are as yet poorly characterized: puberty is delayed in some patients with ALGS, and some are non-responsive to growth hormone"). Furthermore, cross-talk between Notch and the androgen pathway, acting via Hey1 (Belandia et al Mol Cell Biol 2020) may entail different outcomes for loss of Notch signaling in males and females. We agree that for clarity the relevance of sex should indeed be explicitly stated in the introduction.

Revision Plan

We will include a short introduction to the relevance of sex in intracranial bleeds in the introduction.

Results, first section and Figure 1: The classification of cerebral bleedings is not appropriate and all the most surprising: hematoma and hemorrhages are used in clinics to design the same thing; idiopathic means with an unknown mechanism, therefore a subarachnoid hemorrhage caused by the rupture of an aneurysm cannot be classified as "idiopathic". Conversely, a minor trauma can be a triggering factor but cannot be considered as the cause of a hemorrhage.

The definitions of hematoma and hemorrhage are different and generally refer to a smaller delimited bleed that has usually stopped (hematoma, can still be lethal in brain) or a larger/ongoing bleed (hemorrhage). However, if the terms are ambiguous to clinicians, and if they risk being used interchangeably by the authors of the publications on which our review is based, we are grateful for this feedback and will instead refer to "intracranial bleeds (hematoma or hemorrhage)" for data in Fig 1B, and we will remove the first two Venn diagrams. We would like to keep the definitions in Table 1, since the designation of bleeds as hematoma or hemorrhage is based on the original author's description of the bleeds. Regarding the use of the term "idiopathic" and underlying mechanisms, this hinges on what one considers to be an underlying cause. While a ruptured aneurysm in a young child is considered by the reviewer to be the "cause" of the bleed, our view is that an aneurysm in a child is an anomaly, which could be the result of another insult that has not yet been defined (e.g. weakened blood vessels due to JAG1 insufficiency). An aneurysm cannot be the primary cause. To reconcile our viewpoints, we propose to rename the classifications to focus on precipitating factors: "Coagulopathy, Minor trauma, and Spontaneous".

Results, second section (lines 113-151) and Figure 2: Characterization of the bleeding phenotype in the ALGS mouse model is extremely rudimentary. Whereas cerebral hemorrhages in patients with ALGS can occur in the adult, only pups from P0 to P10 were analyzed.

We analyzed and reported brain bleeds in P0-P10 mice (Fig 2N and text) as well as adults (Fig 2M and Fig 4I, Supp Fig 4I).

Screening was limited to a macroscopic analysis of the brain which is unlikely to capture intracerebral hemorrhages and as a result the true prevalence of cerebral bleeding is probably underestimated. More importantly, the origin and the cause of cerebral bleeding were not investigated.

Analysis of brain bleeding included macroscopic brain analysis (Fig 2N, Fig 4I), and Evans blue leakage analysis (Fig 2M, Supp Fig 4I), as well as testing whether increasing blood pressure with AngII worsened Evans Blue leakage (Fig 4I and Supp Fig 4I).

In order to deepen this analysis, we will assess blood brain barrier permeability using 1kDa Cadaverin 555, 3 kDa Dextran-FITC or -TMR, and/or 10kDa Dextran-FITC at P30. Our pilot experiments showed that 3kDa and 10kDa are well delivered to wild type mice by tail vein injection and are easily detectable by overall fluorescence in kidney (please see Fig 1 above in response to Reviewer 2). We will inject wild type and *Jag1*$^{Ndr/Ndr}$ mice at P30 with the fluorescent tracers, and dissect out the brain to quantify leakage of fluorescent tracers. We will use one brain half for homogenization and quantification of fluorescence in a fluorescence spectrometer. The other brain half will be sectioned and imaged if differences in fluorescence are not obvious in the whole brain imaging or fluorescence quantification from homogenized tissue.

Moreover, it is said that Jagged 1 mutant mice exhibit "provoked hemorrhage". I could not find the experimental data supporting this statement.

We apologize for this being unclear. The resin injections presented in former Fig 2J, K, represent provoked hemorrhages. They occur, in the Jag1$^{Ndr/Ndr}$ mice, in the setting of increased intravascular pressure with resin injections.

Also, the statement that "thinner skulls in Jag1ndr/ndr mice likely contributed to nervous system bleeds" is not supported by any experimental data.

Testing this would require inducing trauma to the skull to test whether the thinner bones more easily rupture and lead to brain bleeds. We do not consider this experiment ethical, and our ethical review board would be unlikely to approve the study. This sentence is meant as a statement summarizing the conclusions, but was not meant to represent a result (hence the use of the term “likely”). We will rephrase to “Similar to patients with ALGS, thinner skulls in *Jag1*$^{Ndr/Ndr}$ mice may contribute to nervous system bleeds.”

Results, fourth section, figure 4 and supplementary fig 4. It is said that pericytes in the retinal capillaries were not reduced but supplementary fig 4 does not show any quantification.

We thank the reviewer for focusing on this interesting point. Our initial analysis of this staining revealed large differences in staining quality from one batch of stained animals to another, and we were hesitant to quantify CD13 coverage from these. However, we have now devised a pipeline in which each staining is normalized to the wild type staining from the same batch, allowing us to compare the data quantitatively. We have also added more replicate to this analysis. Overall, there was no difference in CD13 staining between *Jag1*$^{+/+}$ and *Jag1*$^{Ndr/Ndr}$ mice (Fig 2, left panel, t-test not significant, P= 0.3657). However, when we group the mice by sex and genotype, there is tendency towards less staining/coverage in female *Jag1*$^{Ndr/Ndr}$ mice (Fig 2 right panel, Two-way ANOVA with Sidak's multiple comparisons test, Male *Jag1*$^{Ndr/Ndr}$ vs Female *Jag1*$^{Ndr/Ndr}$ P= 0.0619). Because n of 3 per genotype and sex is insufficient to determine whether there are differences, we will add 1-3 mice per sex and genotype and re-analyse these data.

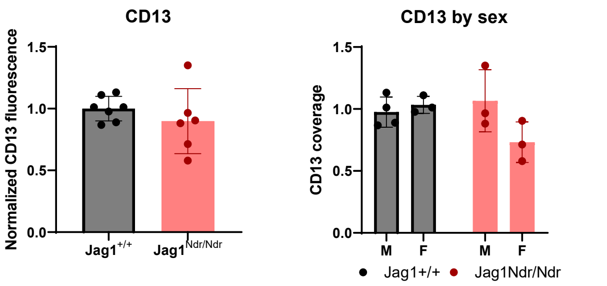

Fig 2. Quantification of CD13+ pericyte coverage in *Jag1*$^{Ndr/Ndr}$ mice. There was no significant difference in CD13 staining between *Jag1*$^{+/+}$ and *Jag1*$^{Ndr/Ndr}$ mice (left panel, t-test not significant, P= 0.3657). However, when mice are grouped by sex and genotype, there is tendency towards less staining/coverage in female *Jag1*$^{Ndr/Ndr}$ mice (right panel, Two-way ANOVA with Sidak's multiple comparisons test, Male *Jag1*$^{Ndr/Ndr}$ vs Female *Jag1*$^{Ndr/Ndr}$ P= 0.0619). This dataset is underpowered to detect true differences and will be extended with new mice and analyses.

It is said that VSMC were less mature in mutant mice, please explain.

VSMCs differentiate from a proliferative, migratory "synthetic" phenotype to a quiescent, non-migratory "contractile" phenotype expressing contractile markers such as ASMA. The mature VSMCs wrap around the arteriole, while the immature VSMCs exhibit a fibroblast-like appearance. The less mature VSMCs in *Jag1*$^{Ndr/Ndr}$ mice were migratory and not wrapped around the arterioles. We will now define mature/immature in the part of the text.
We will also add staining for synthetic VSMC as per comment 2 Reviewer 2 to further this claim.

The finding that Jag1ndr/ndr mice display reduced VSMC coverage is not entirely new given that the ndr mutation is an hypomorphic mutation and that mural cell coverage has been reported to be reduced in Jagged1 KO mice (Benedito et al, Cell 2009). The statement that gaps in VSMC coverage are exacerbated by aging are not supported by quantifications.

Indeed Rui Benedito and Ralf Adams' Cell paper provided numerous new groundbreaking insights into the role of Jag1 and Dll4 in angiogenesis. Their analysis of mural cell coverage was based on low-magnification images of retina in which individual VSMCs could not be

identified (nor quantified), and thus it was not clear what the reduction in staining reflected (fewer VSMCs, less intensely stained VSMCs?)

In our manuscript, we show high-resolution high-magnification images demonstrating both an absence of contact points between VSMCs in adult mice, and larger gaps in VSMC coverage in 1 year old mice. We had quantified the gaps in VSMC coverage in 3-6 month old adults (former Fig 4K), and shown that such gaps were rare in untreated $Jag1^{Ndr/Ndr}$ mice at this stage. We now add quantification of P30 and one year old mice (Fig 3, at right), which demonstrate that no gaps are present at P30, while multiple gaps are present in 50% of $Jag1^{Ndr/Ndr}$ mice at 1 year of age. These new data will be included in an updated VSMC figure (modification of former Fig 4).

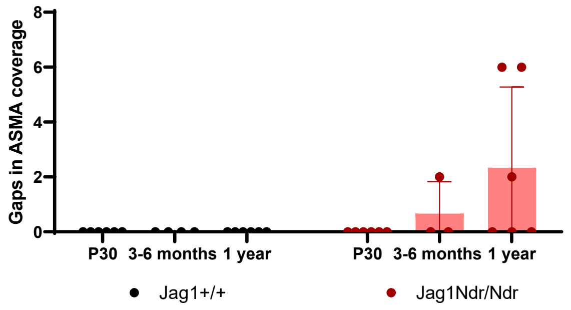

Fig 3. Quantification of ASMA+ VSMC coverage in $Jag1^{Ndr/Ndr}$ mice. There are no gaps in VSMC coverage at any stage examined in $Jag1^{+/+}$ mice. There are no gaps in VSMC coverage in P30 $Jag1^{Ndr/Ndr}$ mice, but gaps are present in 1 of 3 mice at 3-6 months (data from former Fig 4K) and in 3 of 6 mice at 1 year of age, displaying an exacerbation by increasing age.

Regarding novelty and different models: we have now analysed the VSMC phenotype of $Jag1^{\Delta DSL/+}$ mice, which model the liver disease of Alagille syndrome (Thakurdas et al 2015). We include these data in a new Supplementary Fig X (and shown here in Fig 4 at right). Our analyses show that $Jag1^{\Delta DSL/+}$ mice display a similar phenotype to $Jag1^{Ndr/Ndr}$ mice with fewer arteries and veins, and display arteriovenous crossings. $Jag1^{\Delta DSL/+}$ also display overall weaker ASMA staining, but no small or large gaps between VSMCs. The $Jag1^{\Delta DSL/+}$ mice thus exhibit a milder phenotype than $Jag1^{Ndr/Ndr}$ mice. Currently this analysis is based on three circa P30 mice, and two circa one year old mice. We aim to complete the analysis with n=6 at each stage.

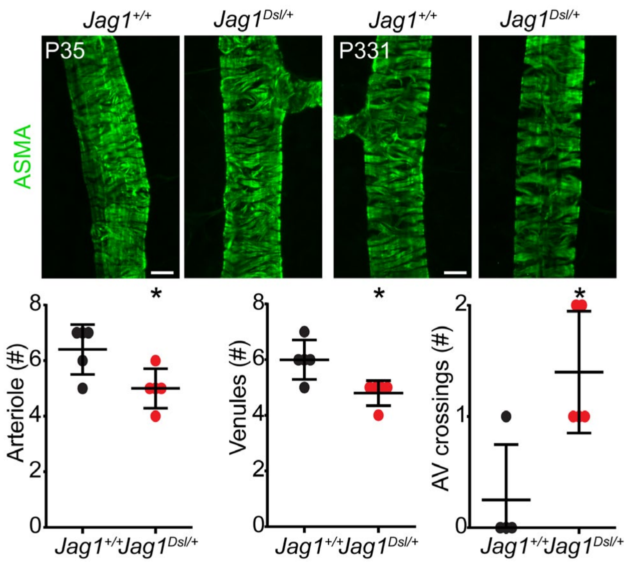

Fig 4. Analysis of VSMC coverage, arteriole and venule number, and arteriovenous crossings shows a relatively mild phenotype in $Jag1^{DDSL/+}$ mice.

In the AngII experiment (panel H), blood pressure measurements are not shown. By the way, pooling mice with age ranging from 3 to 7.5 months in a mouse model with an age-dependent phenotype seems counterintuitive.

Yes, we agree, if it would be possible to breed, obtain and keep matched sets of *Jag1*$^{Ndr/Ndr}$ mice at the same age this would be the best approach and reduce variability in the data. Unfortunately, we had to balance synchronizing the minipump surgeries and blood pressure measurements (which were done and will be included in the revised manuscript, provided as Fig 5 here) with mating and obtaining surviving mice. These mice breed slowly and only one fifth of the 25% of homozygous mice survive to adulthood. Thus, in order to reach n of 8 Jag1Ndr/Ndr mice in the Ang II treated group (of which one died and two did not exhibit an increase in blood pressure, as specified in Materials and Methods), 40 *Jag1*$^{Ndr/Ndr}$ mice had to be generated (as well as 120 mice of other genotypes). Unfortunately, the surviving mice were from a range of ages.

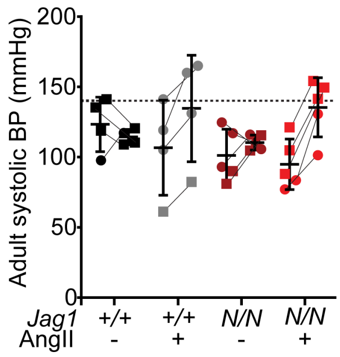

Fig 5. Blood pressure before and after AngII in *Jag1*$^{+/+}$ and *Jag1*$^{Ndr/Ndr}$ mice.

Nonetheless, despite these limitations that we agree could confound analysis, the data clearly demonstrate that an AngII-induced increase in blood pressure leads to an ASMA gap phenotype in all *Jag1*$^{Ndr/Ndr}$ mice (former Fig 4J and 4K), and in fact the phenotype is worse than in 1-year-old mice (in which 50% of *Jag1*$^{Ndr/Ndr}$ mice display gaps, Fig 3 above), suggesting that increased blood pressure is more detrimental than increasing age.

Results, fifth section, Figure 5 (mislabeled figure 6). It is almost counterintuitive to have a reduced density in the ICP and a lower number of vertical sprouts in 3-6 months old mice and a normal density at 1 year.

Thank you for pointing out the mislabelling. We will correct this.

The differences in vascular length at 3-6 months and one year (former Fig 5C and G) are minor, and generally the wild type and *Jag1*$^{Ndr/Ndr}$ data overlap. However, the difference between the 3-6 month and 1-year branching point and vertical sprouting data (former Fig 5 D, H, L and M) is that the *Jag1*$^{Ndr/Ndr}$retina at 3-6 months resembles a 1-year-old *Jag1*$^{+/+}$ retina. But the *Jag1*$^{Ndr/}$Ndr ICP capillary vascular length or branching point/vertical sprouting phenotypes do not worsen between 3-6 months and one year. This explains why there is a difference between *Jag1*$^{+/+}$ and *Jag1*$^{Ndr/mice}$ at 3-6 months but not at one year – they converge. However, the intermediate capillary plexus in 1-year-old Jag1Ndr/Ndr mice contains disorganized branches with gaps in the ICP layer (Fig 5K in manuscript, white arrowheads), which are not present in

similar views of 3-6 months adult Jag1Ndr/Ndr mice. These data show that specific aspects of the capillary phenotype worsen with age, which were not captured by vascular length or branchpoint analysis. We will clarify this in the results section and in the discussion.

Another challenge in interpreting these data is that detrimental phenotypes associated with the death of the mouse will be selected against by the accumulating deaths in the Jag1Ndr/Ndr cohort, meaning that mice analyzed at 1 year will be the healthiest possible Jag1Ndr/Ndr mice. This will also be clarified and discussed in the discussion.

Again, the statement that the RGC axons are healthy in Jag1Ndr/Ndr retinas at P10 is not supported by quantification. Also, the authors show a reduction in the number of RGC axons at P40. Whereas they document VSMC degeneration much later, they claim that the onset of vascular degeneration was associated with RGC degeneration.

We will quantify NF staining in P10 retinas. This was not done originally because the NF antibody is mouse and requires perfusion of the mouse to eliminate mouse IgG in blood (and background staining as in Fig 5O, which must be corrected for in analysis), which is not straightforward in P10 mice. The RGC breakdown was interpreted in the context of a degenerated capillary bed which could reduce oxygenation. We show in Supplementary Fig 3 that that the superficial capillary plexus is underdeveloped at P5, but similar to wild type at P10, which is when the RGCs also look normal. The superficial capillary plexus begins to diverge at P15, and the RGCs appear degraded at P40. We will add analysis of the capillary beds in P30 retinas to determine whether the capillary beds are normal at this stage.

Regarding VSMCs: these are not normal at any stage in *Jag1*$^{Ndr/Ndr}$ mice (Supplementary Fig 4B and Fig 4).

Results, sixth section, figure 6. The rationale to include in the analysis of the retinal fundus CADASIL patients is far from obvious to this Reviewer and does not bring anything. Indeed, whereas ALGS is a developmental disease, CADASIL is a degenerative disease. Moreover, patients with AGLS exhibit cerebral bleeding, whereas cerebral bleeding in patients with CADASIL is extremely rare.

Because VSMCs are abrogated both in *Jag1* loss of function and *Notch3* loss of function mouse models, we propose that the VSMC phenotype in the ALGS mouse model is likely due to Jag1-Notch3 signaling defects. Although multiple consequences of *NOTCH3* mutations have been described in CADASIL (most often toxic gain of function), it has been also been proposed that reduced NOTCH3 signaling can contribute to the VSMC phenotype in CADASIL (and indeed, a CADASIL-like patient was reported with homozygous *NOTCH3* loss of function

and paucity of VSMCs but no GOMs, Pippucci et al EMBO Mol. Med. 2015, and recently reviewed by Samira Hosseini-Alghaderi and Martin Baron, Biomolecules 2021). We therefore considered it of interest to include CADASIL patients in the analysis. Importantly, the presence of multiple vascular defects in ALGS patients but not CADASIL patients suggests that tortuosity, for example, is related to endothelial cell defects (present in Jag1 mutant conditions) rather than VSMC defects (present in both Jag1 and Notch3 mutant conditions). To clarify the rationale, we will provide this context in the beginning of this results section.

Also of note, patients with ALGS exhibit both intracranial bleeding and ischemic events, though the frequency of each has not yet been reported. We have chosen to focus on the intracranial bleeding events in this manuscript. Our data suggest that ALGS is both a developmental and a degenerative disease, with regards to the vasculature.

Reviewer #3 (Significance (Required)):

The Alagille syndrome is a developmental disease primarily affecting the liver, caused by hypomorphic or loss of function in the Jagged1 ligand or more rarely in the Notch2 receptor. Nevertheless, cerebral hemorrhage in patients with ALGS is of clinical significance, causing up to 25% of deaths. Because the penetrance of these hemorrhages is incomplete, identifying risk factors would constitute a significant advance.

Unfortunately, retinal vessel abnormalities (arteriovenous crossings, reduction in blood vessel numbers and increase vessel tortuosity) identified in patients or mice with ALGS in this study are unlikely to be risk factors for cerebral hemorrhage for the reasons entioned in the previous section.

We do not suggest that retinal arteriovenous crossings, reduction in blood vessel numbers and increase vessel tortuosity are risk factors for cerebral hemorrhage, rather that they could be used as biomarkers indicating the presence of compromised vasculature in brain. Such a correlation would be worth investigating in future studies, but a large-scale preclinical investigation of this cannot be justified in humans before showing that patients have defects in retina (this manuscript). Vessel tortuosity is a known indicator of reduced wall elasticity which could lead to bleeds, aberrant artery-vein crossings indicates incorrect vascular pathfinding that could lead to local hypoxia in regions with fewer blood vessels or with compressed vessels, and similarly reduced numbers of blood vessels would limit vascularization and support of the tissue.

Expertise of this Reviewer encompasses clinical neurology, the physiology and pathology of the cerebroretinal vasculature and the Notch signaling pathway.

3. Description of the revisions that have already been incorporated in the transferred manuscript

No revisions have yet been incorporated into the manuscript, since we expect extensive editing with the generation of new data. Several of the requested experiments or analyses have been completed and have generated data as listed under Point 2.

4. Description of analyses that authors prefer not to carry out

There is one experiment that we cannot address experimentally. Reviewer 3 writes: “Also, the statement that "thinner skulls in Jag1ndr/ndr mice likely contributed to nervous system bleeds" is not supported by any experimental data.”

We do not think it is ethically possible to experimentally test in the mice whether the thinner skulls contribute to more frequent brain bleeds. This would necessitate inflicting trauma on the mouse head to test whether an impact that does not break a wild type skull does fracture a *Jag1*$^{Ndr/Ndr}$ skull. Perhaps this could be tested instead with modelling of force and bone strength, but this is beyond the scope of our revision. Because our statement was only intended to convey the logical interpretation that thinner bones could more easily fracture and result in brain bleeds, we propose to address this reviewer feedback by amending the text as follows: “Similar to patients with ALGS, thinner skulls in *Jag1*$^{Ndr/Ndr}$ mice ***may*** contribute to nervous system bleeds.”

4th Feb 2022

Dear Dr. Andersson,

Thank you for the submission of your manuscript to our editorial offices. I have now had the opportunity to read it, together with the referees' reports and your rebuttal letter, and to discuss them with the other members of our editorial team.

We agree that the study fits the scope of the journal, and we appreciate that you are willing to address most of the points raised by the reviewers. We thus encourage you to submit a revised version of your manuscript to our office, including the modifications and revisions described in your point-by-point letter. Acceptance of the manuscript will entail a second round of review. EMBO Molecular Medicine encourages a single round of revision only and therefore, acceptance or rejection of the manuscript will depend on the completeness of your responses included in the next, final version of the manuscript. For this reason, and to save you from any frustrations in the end, I would strongly advise against returning an incomplete revision.

***

When submitting your revised manuscript, please carefully review the instructions that follow below. Failure to include requested items will delay the evaluation of your revision:

We require:

1) A .docx formatted version of the manuscript text (including legends for main figures, EV figures and tables). Please make sure that the changes are highlighted to be clearly visible.

2) Individual production quality figure files as .eps, .tif, .jpg (one file per figure). For guidance, download the 'Figure Guide PDF' (https://www.embopress.org/page/journal/17574684/authorguide#figureformat).

3) A .docx formatted letter INCLUDING the reviewers' reports and your detailed point-by-point responses to their comments. As part of the EMBO Press transparent editorial process, the point-by-point response is part of the Review Process File (RPF), which will be published alongside your paper.

4) A complete author checklist, which you can download from our author guidelines (https://www.embopress.org/page/journal/17574684/authorguide#submissionofrevisions). Please insert information in the checklist that is also reflected in the manuscript. The completed author checklist will also be part of the RPF.

5) It is mandatory to include a 'Data Availability' section after the Materials and Methods. Before submitting your revision, primary datasets produced in this study need to be deposited in an appropriate public database, and the accession numbers and database listed under 'Data Availability'. Please remember to provide a reviewer password if the datasets are not yet public (see https://www.embopress.org/page/journal/17574684/authorguide#dataavailability).
In case you have no data that requires deposition in a public database, please state so in this section. Note that the Data Availability Section is restricted to new primary data that are part of this study.

6) For data quantification: please specify the name of the statistical test used to generate error bars and P values, the number (n) of independent experiments (specify technical or biological replicates) underlying each data point and the test used to calculate p-values in each figure legend. The figure legends should contain a basic description of n, P and the test applied. Graphs must include a description of the bars and the error bars (s.d., s.e.m.). Please indicate exact p values in the figures or their legends.

7) We would also encourage you to include the source data for figure panels that show essential data. Numerical data should be provided as individual .xls or .csv files (including a tab describing the data). For blots or microscopy, uncropped images should be submitted (using a zip archive if multiple images need to be supplied for one panel). Additional information on source data and instruction on how to label the files are available at
.

8) Our journal encourages inclusion of *data citations in the reference list* to directly cite datasets that were re-used and obtained from public databases. Data citations in the article text are distinct from normal bibliographical citations and should directly link to the database records from which the data can be accessed. In the main text, data citations are formatted as follows: "Data ref: Smith et al, 2001" or "Data ref: NCBI Sequence Read Archive PRJNA342805, 2017". In the Reference list, data citations must be labeled with "[DATASET]". A data reference must provide the database name, accession number/identifiers and a resolvable link to the landing page from which the data can be accessed at the end of the reference. Further instructions are available at .

9) We replaced Supplementary Information with Expanded View (EV) Figures and Tables that are collapsible/expandable online. A maximum of 5 EV Figures can be typeset. EV Figures should be cited as 'Figure EV1, Figure EV2" etc... in the text and their respective legends should be included in the main text after the legends of regular figures.
- For the figures that you do NOT wish to display as Expanded View figures, they should be bundled together with their legends in a single PDF file called *Appendix*, which should start with a short Table of Content. Appendix figures should be referred to in the main text as: "Appendix Figure S1, Appendix Figure S2" etc.
- Additional Tables/Datasets should be labeled and referred to as Table EV1, Dataset EV1, etc. Legends have to be provided in a separate tab in case of .xls files. Alternatively, the legend can be supplied as a separate text file (README) and zipped together with the Table/Dataset file.
See detailed instructions here:
.

10) The paper explained: EMBO Molecular Medicine articles are accompanied by a summary of the articles to emphasize the major findings in the paper and their medical implications for the non-specialist reader. Please provide a draft summary of your article highlighting
- the medical issue you are addressing,
- the results obtained and
- their clinical impact.

This may be edited to ensure that readers understand the significance and context of the research. Please refer to any of our published articles for an example.

11) For more information: There is space at the end of each article to list relevant web links for further consultation by our readers. Could you identify some relevant ones and provide such information as well? Some examples are patient associations, relevant databases, OMIM/proteins/genes links, author's websites, etc...

12) Conflict of interest: We updated our journal's competing interests policy in January 2022 and request authors to consider both actual and perceived competing interests. Please review the policy https://www.embopress.org/competing-interests and update your competing interests if necessary.

13) Every published paper now includes a 'Synopsis' to further enhance discoverability. Synopses are displayed on the journal webpage and are freely accessible to all readers. They include a short stand first (maximum of 300 characters, including space) as well as 2-5 one-sentences bullet points that summarizes the paper. Please write the bullet points to summarize the key NEW findings. They should be designed to be complementary to the abstract - i.e. not repeat the same text. We encourage inclusion of key acronyms and quantitative information (maximum of 30 words / bullet point). Please use the passive voice. Please attach these in a separate file or send them by email, we will incorporate them accordingly.
Please also suggest a striking image or visual abstract to illustrate your article as a PNG file 550 px wide x 300-600 px high.

14) As part of the EMBO Publications transparent editorial process initiative (see our Editorial at http://embomolmed.embopress.org/content/2/9/329), EMBO Molecular Medicine will publish online a Review Process File (RPF) to accompany accepted manuscripts.

In the event of acceptance, this file will be published in conjunction with your paper and will include the anonymous referee reports, your point-by-point response and all pertinent correspondence relating to the manuscript. Let us know whether you agree with the publication of the RPF and as here, if you want to remove or not any figures from it prior to publication.
Please note that the Authors checklist will be published at the end of the RPF.

EMBO Molecular Medicine has a "scooping protection" policy, whereby similar findings that are published by others during review or revision are not a criterion for rejection. Should you decide to submit a revised version, I do ask that you get in touch after three months if you have not completed it, to update us on the status.

I look forward to receiving your revised manuscript.

Yours sincerely,

Lise Roth

Lise Roth, PhD
Editor
EMBO Molecular Medicine

***

Rev_Com_number: RC-2021-01147
New_manu_number: EMM-2022-15809
Corr_author: Andersson
Title: Sex differences and risk factors for bleeding in Alagille syndrome

Reviewer #1 (Evidence, reproducibility and clarity (Required)):

In manuscript RC-2021-01147, Hankeova et al. present their findings in regard to the potential risk factors that are associated with intracranial bleeding in patients suffering from a congenital disease called Alagille syndrome (ALGS) and the sexual dimorphism associated with some of ALGS vascular phenotypes. The authors first performed a systematic literature review for case studies of ALGS patients with vascular-related complications with an eye on potential gender-based differences and found that a significantly higher number of idiopathic intracranial hemorrhage instances was reported in girls compared to boys affected with ALGS. The authors then used an ALGS model previously established by their group (*Jag1*$^{Ndr/Ndr}$ mutant mice) to further study the bleeding risk factors associated with reduced JAG1 function and their cellular mechanisms. They found that *Jag1*$^{Ndr/Ndr}$ animals showed an increase in the rate of spontaneous and provoked central nervous system bleeding. Despite cholestasis, the animals did not show abnormal coagulation. However, the mutant animals had thinner skulls and showed fewer and more tortuous blood vessels. One aspect of these vascular defects showed statistically significant sex-specific difference in this mouse model. Moreover, *Jag1*$^{Ndr/Ndr}$ animals displayed a reduction in the coverage of endothelial cells by vascular smooth muscle cells (VSMCs), a phenotype which was worsened by inducing hypertension in these animals for just two weeks. The authors also uncovered specific vascular abnormalities in the retinas of the mutant mice accompanied by an age-dependent reduction in the coverage of the retina by retinal ganglion cell axons. Finally, analysis of retinal fundus photographs indicated a significant increase in venous tortuosity in patient with ALGS compared to healthy controls and patients with another cholestatic disease. Intracranial bleeding is a major cause of morbidity and mortality in patients with ALGS. However, the mechanisms and risk factors for these events are not well understood (perhaps other than the sensitivity of these patients to head trauma). Moreover, an animal model for ALGS vascular defects and hemorrhage in the CNS has not been described previously. The current manuscript therefore makes important contributions to the field by providing a link between idiopathic brain hemorrhage in ALGS patients and the patients' gender. Moreover, the vascular abnormalities described in *Jag1*$^{Ndr/Ndr}$ animals provide a model for future mechanistic and preclinical therapeutic studies on this understudied yet highly important aspects of ALGS. The rapid decline in the VSMC coverage of arteries in response to hypertension reported here in a mouse model of ALGS suggests that blood pressure should be carefully controlled in patients with ALGS, especially females. Finally, the study offers a new non-invasive way to assess whether a given ALGS patient has vascular abnormalities and motivates future studies to examine whether retinal vascular abnormalities have a predictive value for the risk of brain hemorrhage in these patients. In addition to these strengths, in my opinion, the paper has two major shortcomings. First, as explained below, the emphasis on the sex-difference of the phenotypes in mice is exaggerated, to the extent that it overshadows the value of establishing a mouse model for ALGS vascular defects for the first time (regardless of sexual dimorphism). Second, there has not been any attempts to provide a molecular mechanism for the observed phenotypes. However, given the enormous amount of work that has already gone into this manuscript and its potential for future preclinical studies, I don't think mechanistic studies should be considered a requirement for the publication of this work.

It is a pleasure to read your summary of our work, and we thank you for explicitly identifying strengths and weaknesses of the manuscript, allowing us to edit the text productively.

Major points

1) It seems that the only statistically significant sex-specific difference in the mouse analysis is the number of venules in the retina.

There were two significantly different sex-dimorphic phenotypes in the *Jag1*$^{Ndr/Ndr}$ mice: number of venules (Fig 3D), and venous tortuosity (Fig 3H). Although this was stated in the text and statistical testing was descried in the figure legend, the figure panel (graph) itself did not make clear that venule tortuosity was statistically tested for impact of sex. Now, we first tested whether age and genotype interact to impact tortuosity. Because age does not impact tortuosity nor interact with genotype, we merged the P30 and 1 year tortuosity data and re-evaluated the sex differences with all possible replicates. The analysis demonstrates (again) that venous tortuosity is significantly higher in female *Jag1*$^{Ndr/Ndr}$ mice, and that genotype interacts with sex in a two-way ANOVA. Importantly, this is now visualized more clearly in Fig 3E-3H (in particular Fig 3H).

2) In contrast, the phenotypes that would potentially increase the risk of bleeding (like skull thickness) are not shown to be sexually dimorphic, and neither is brain bleeding itself (the gender of the 4 animals without bleeding in Figure 4I is not mentioned). In fact, for skull thickness the authors have almost exclusively presented data from males. However, in the Abstract and elsewhere in the manuscript, the way the authors use "sex-specific defects" in a number of sentences implies a much broader sexual dimorphism in the mouse. For example, the sentence " *Jag1*$^{Ndr/Ndr}$ mice exhibited sex-specific defects with sporadic bleeds, a thin skull, tortuous blood vessels ..." implies that the phenotypes listed after "with" might show a difference between males and females. The title of the paper starts with "Sex differences", but only one phenotype in the ALGS mouse model is sex-specific. The easiest way to address this issue would be to carefully review the text and explicitly mention the only sex-specific phenotype in these instances, followed by other phenotypes that are not sex-specific. The alternative would be to perform statistical analysis separately on males and females for other panels, similar to what is shown in Figure 3B-D.

As we described in the Materials and Methods, experiments generally include mice of both male and female sex. Experiments in which the results suggested sex differences were expanded with additional mice of each sex to determine whether sex differences were present. The reviewer is absolutely correct that the skull analysis is an exception to this generalization. We therefore now scanned and analyzed new skulls from male and female wild type and *Jag1*$^{Ndr/Ndr}$ mice at P30 as it was not possible to generate new mice of 7-8 months of age within this revision period (n of 5-6 per sex and genotype). We did not detect any sex differences in the skull thickness analysis, in which all *Jag1*$^{Ndr/Ndr}$ mice exhibited smaller/thinner skulls (Fig 2 G-I, Fig EV1).

The sex of the animals without bleeding in Fig 4Q are the animals in Fig 4P and are labelled as two females and three males (circles for females, squares for males). We also updated the text to clarify: "However, macroscopic evaluation of the brain revealed that one of five AngII-treated *Jag1*$^{Ndr/Ndr}$ mice (a male) displayed Evans blue leakage outside of the intracranial vessels (Fig 4Q).".

The discovery, based on our systematic review, that female patients are over-represented when it comes to spontaneous intracranial bleeds in Alagille syndrome is an important and novel sex difference, which we think warrants emphasis.

We updated the text to ensure that the conjunction "with" is not used in the fashion the reviewer pointed out, as we agree this is misleading and was not our intention. We have edited the text to be explicit about which features are dimorphic and which are not.

3) Did the postnatal lethality shown in Figure 2A occur similarly for both males and females?

This is an excellent question, and we thank the reviewer for asking it. We initially did not separate the data based on sex for this analysis because we considered the sample size underpowered to address survival (dataset was eight males and eight females). Now, we analyzed 26 *Jag1*$^{Ndr/Ndr}$ males of which 7 died during first 10 days and 20 *Jag1*$^{Ndr/Ndr}$ females of which 7 died during first 10 days. Fewer females were born, and proportionally more females than males died during the first 10 postnatal days, but the difference was not statistically significant (Figure 2A).

4) I understand that the focus on sex difference might have arisen after some of the data were generated. However, given the emphasis of the story on this issue, either additional data needs to be added or the claims need to be toned down.

We now include additional data (complete sex- and age-matched sets of skulls microCT, expanded survival analysis, increased number of replicates for quantification of CD13 coverage, P30 blood brain barrier analysis) to determine whether other phenotypes are sexually dimorphic. None of these were sexually dimorphic. Our former conclusion that there are three sexually dimorphic phenotypes (spontaneous bleeds in patients, venule number and venous tortuosity in the mouse model) is unchanged, but strengthened with further replicates and clearer statistical testing/graphing. Based on the results of these analyses, we stringently edited the text to ensure that there are no overstatements, and that the text reflects the new results.

5) Lines 139-142, description of Figure 2M: Since the Evans blue staining did not show a statistically significant increase in mutants, the authors should remove the sentence "... had mildly increased Evans blue leakage ...".

We removed this phrase. We performed new experiments testing leakage of Dextran 3 kDa and Cadaverin 1 kDa, as described below in a response to Reviewer 2. The three sets of experiments also showed no change in blood brain barrier permeability. We modified the text: "We did not detect any changes in the blood brain barrier permeability of $Jag1^{Ndr/Ndr}$ mice (Fig 2L, Fig EV2G, EV2J).".

6) The way the authors have compared CADASIL and ALGS in the Discussion can be misleading to readers who are not Notch pathway experts. Although both CADASIL mouse models and the ALGS model used in this study show abnormalities in VSMCs, the mechanisms for such abnormalities are likely to be quite different (toxic gain-of-function versus haploinsufficiency). Similarly, talking about Notch3 mutant and CADASIL in the same sentence without further explanation and also using the phrase "defective NOTCH3 in CADASIL" (line 305) would imply to non-experts that loss of NOTCH3 signaling is a major issue in CADASIL. The authors should clarify these issues in the manuscript.

We used ""defective NOTCH3 in CADASIL" as the most careful way of referring to the NOTCH3 mutations in CADASIL, which defy strict definition. We agree that the current text is insufficient, with language shortcuts that arose due to a character count limit adjustment. The discussion was modified to clarify the difference between the mouse models and patients, toxic gain of function vs loss of function, and developmental vs pathological mechanisms.

7) Throughout the manuscript various mouse ages have been used in experiments, between P10 to one year of age. However, it is not clear why those ages were chosen for each experiment. For example, RGC problems studied in figure 5N and 5O (P10 and P40) are suggested to result from vascular defects. However, the vascular defects in these retinas were analyzed between 3-12 months of age.

We apologize if the choice of stages was unclear and are grateful that this was pointed out so that it can be rectified. In general terms, we have chosen to study angiogenesis at P5-P15 ongoing), and vascular homeostasis in adult (3-6 months) and older mice at the onset of senescence/middle age (one year). Indeed, the onset of vascular break-down is an important timepoint to identify, in the context of the RGC breakdown, and we thank the reviewer for pointing this out. We have therefore analyzed P30 retinal blood vessels, including all three capillary plexuses (Fig 5A-5D), and our data show that reduced ICP branching, and reduced vessel density, similar to that observed in adult mice, occur prior to the RGC breakdown demonstrated at P40 (Fig 5E-5H).

Minor comments:

8) The authors suggest in the Discussion that the vascular tortuosity observed in ALGS arises from defects in ECs not from VSMCs. Given the difference in the disease mechanisms of ALGS and CADASIL and the potential crosstalk between these two cell types, this conclusion is rather speculative. Analyzing the expression of Notch pathway targets in these two cell types might have provided some clue into the cell type responsible for the observed phenotypes.

Yes, we agree that Notch target gene analysis in these cell types would have been informative. We had performed three scRNA seq experiments to address this question: (1) Smart Seq2 of whole retina, (2) endothelial cell antibody-enriched and neural cell-depleted endothelial cells and (3) sorted VE-Cad tomato-expressing endothelial cells. However, all three experiments yielded too many cells of the wrong cell type and impure populations of endothelial cells, and we were thus unable to address this interesting question using scRNA seq. We expect different populations of ECs to experience different levels and types of Notch signaling (eg tip cells vs stalk cells) and we therefore did not think it was meaningful to pursue bulk approaches. For this reason, we do not address the mechanisms of tortuosity in the Results section, but have reserved this topic for the Discussion, which we think is a suitable place for speculation. We now clarify in the Discussion that this hypothesis should be addressed with experiments in the future.

9) Please add some text to the Results section to further explain the data shown in Figure S3. In the current version, I think only one short sentence is used to describe this figure.

Indeed, because these data were not novel, and due to character count limits, we had chosen to state only that vascular development in *Jag1*$^{Ndr/Ndr}$ mice phenocopies the *Jag1* EC knockout. We have updated the text to include more information on the data shown in Fig EV3 (former Figure S3): "Retinal angiogenesis takes place postnatally during the first three weeks after birth [34] (Fig EV3A). The *Jag1*$^{Ndr/Ndr}$ vasculature displayed delayed outgrowth (Fig EV3B, EV3C) with abnormal tip cell morphology (Fig EV3D-EV3F). Primary vascular plexus remodeling was defective during the first fifteen postnatal days in *Jag1*$^{Ndr/Ndr}$ mice (Fig EV3G-EV3M). The delay in vascular growth and remodeling was accompanied by decreased EC proliferation (Fig EV3N) and DLL4 upregulation in the vascular front (Fig EV3O-P). "

10) Lines 165-166, "an effect that was driven by significantly increased tortuosity specifically in female *Jag1*$^{Ndr/Ndr}$ mice at P30": This cannot be concluded from the presented data in figure 3G, as the statistics was performed for all animals combined, not just the females. Please rephrase.

This is a misunderstanding, due to our unclear data/analysis presentation. Statistical analysis had been done on separate sexes, as described in the former figure legend. "(*Jag1*^{CTRL} n=6, *Jag1*$^{Ndr/Ndr}$ n=8, unpaired t test, **P=0.0085, and One-way ANOVA, **P=0.0013, followed by

Tukey's multiple comparisons test to test for sex effect, $Jag1^{CTRL}$ vs $Jag1^{Ndr/Ndr}$ females ***P=0.001, $Jag1^{CTRL}$ vs $Jag1^{Ndr/Ndr}$ males ns, P=0.2320, $Jag1^{Ndr/Ndr}$ females vs $Jag1^{Ndr/Ndr}$ males *P=0.0286). We have updated and modified this analysis. First, we test the impact of age on tortuosity, showing age does not impact tortuosity. We therefore merge P30 and 1-year-old data, and instead separate the data by sex (Fig 3H). The analysis showed a significant interaction between sex and genotype, demonstrating that venous tortuosity is highest in female $Jag1^{Ndr/Ndr}$ mice.

11) What is the purple staining in Figure 2P?

Thank you for noting this, a panel label disappeared during formatting. The purple staining is ASMA and was now corrected in the figure (Fig 2O).

12) Line 52: Alagille syndrome (ALGS) is 'a' pediatric disorder ...

Line 53: 'facies' is used more commonly than 'faces'.

Line 199: Are the authors discussing the aneurysm in 4B? 4G does not show an image.

Line 229: 'Fig. 5J' instead of 'Fig. 6J'.

Line 289: 'mouse' instead of 'moude'.

Line 290: 'venous' instead of 'evenous'.

Line 717: 'Fig. 5' instead of 'Fig. 6'.

Thank you for the helpful text and figure call-out corrections, very much appreciated.

Reviewer #1 (Significance (Required)):

Please see above.

Reviewer #2 (Evidence, reproducibility and clarity (Required)):

Emma Andersson and colleagues report about Allagille syndrome, a rare congenital disorder affecting blood vessels and bile ducts. The responsible mutations are frequently found in the JAG1 gene, which is a Notch ligand. Some patients have NOTCH2 gene mutations. As such, it

is a disorder due to impaired Notch signaling. While the disease has been characterized quite well in recent years there is still little known about sex-specific differences in the phenotype. Andersson et al., have recently published a very interesting mouse model for Alagille syndrome which contains a Jag1 H268Q missense mutation (Gastroenterology, 2018 Mar;154(4):1080-1095). These mice were called Jag1+/Ndr mice. However, so far mostly the liver phenotype was studied in these mice and not so much the vascular phenotype which most likely plays a major role in the pathogenesis of this disease.

We thank the reviewer for referring to our previous work and identifying the novel aspects of this manuscript. Indeed, a model for vascular defects in Alagille syndrome has not yet been described at all.

The paper is well written, however sometimes one might have the feeling that it could be worthwhile to split it in two manuscripts. The images are of high quality. Materials and Methods are detailed. Introduction and Discussion are interesting and well balanced.

We thank the reviewer for the positive comments on the structure of the manuscript and the quality of the images. We agree that it is a large manuscript, and indeed discussed at length whether to prepare one or two manuscripts. We opted for one large manuscript in order to be able to report and integrate the discussion and analysis.

Otherwise I have only few comments:

1)The number of analyzed Ndr/Ndr mice n=17 is a bit low, especially as some of the defects and brain bleedings occur at rather low frequency e.g. 1/17.

We apologize for the misunderstanding – the brain bleeds occurred in 2 of 53 $Jag1^{Ndr/Ndr}$ mice (Previously stated on page 6: "Fifty-three $Jag1^{Ndr/Ndr}$ pups were monitored daily from birth until P10 (including the 16 described above) and macroscopically obvious brain hemorrhages occurred in two $Jag1^{Ndr/Ndr}$ pups (…)"). This section was rewritten to clarify the numbers of mice analyzed. We have further deepened our analysis while collecting additional data for survival and sex differences (Fig 2, see comment 3 Reviewer 1). Including the new data set, we have followed 83 $Jag1^{Ndr/Ndr}$ pups daily between P0-P10. However, the number of spontaneous intracranial bleeds has not increased, although spontaneous deaths and cannibalism by the mother precludes identifying cause of death in the majority of the $Jag1^{Ndr/Ndr}$ pups that died.

2) Analysis of vascular permeability was only done with Evan's blue. This showed that there is no gross defect. However, this assay is not specific enough to detect slight disturbance of vessel permeability in particular also at the blood brain barrier. The Betsholtz group

established similar assays using low molecular weight tracers and it is strongly recommended to extent this investigation.

We agree this is an interesting experiment and we thank you for the suggestion. We have expanded upon the vessel permeability assay by injecting a mix of Dextran 3 kDa FITC and Cadaverin 1 kDa 555 in P30 mice (Fig EV2D – EV2I). However, the mice at this stage still suffer from liver cholestasis and have increased bilirubin, which is highly auto fluorescent, and could complicate interpretation of the results. We performed macroscopic evaluations of brains and kidney and measured fluorescence intensity in brains, kidneys and plasma. The analysis revealed high fluorescence in plasma of *Jag1*$^{Ndr/Ndr}$ mice, likely reflecting auto fluorescent albumin-bilirubin, and a significant increase in fluorescence in kidneys of *Jag1*$^{Ndr/Ndr}$ mice, which was most pronounced for the 555 channel (Fig EV2F, EV2I). While the increase in fluorescence could reflect either fluorescent tracer or albumin-bound bilirubin, we did not detect any changes in fluorescence in brain samples from *Jag1*$^{Ndr/Ndr}$ mice, indicating neither bilirubin-albumin nor fluorescent tracer crossed the blood-brain-barrier (Fig EV2G, EV2J).

3)The VSMC phenotype is very interesting and somehow fits to other Notch-related pathologies like CADASIL. It would be very interesting to see if also the cellular phenotype of VSMCs changes in terms of plastic vs. contractile.

Thank you for the positive words, we agree this is interesting. We analyzed the expression of contractile (ASMA, SM22, MYH11) and synthetic (VIM, COL1) VSMC markers in arterioles from 3 months to 1-year old mice (Fig EV4D, EV4E). We focused on areas in which contractile VSMC markers were weakly expressed or absent. In ASMA-negative gaps the contractile VSMC markers were similarly reduced 80-90% compared to ASMA+ areas, whereas VIM was reduced by 25% and COL1 by 65% (Fig EV4F, EV4G). The overall intensity of Vim was not significantly upregulated in *Jag1*$^{Ndr/Ndr}$ mice. There is thus no upregulation of synthetic VSMC markers in *Jag1*$^{Ndr/Ndr}$ mice.

4)The authors claim that there are strong sex-specific differences in the Ndr/Ndr mice. Indeed, some of the experiments indicate in this direction. However, a thorough investigation of all phenotypes including bleeding and statistical testing is missing. Moreover, a table to give an overview about this topic would be helpful.

Statistical testing was done as described in the Materials and Methods: "Experiments generally include mice of both male and female sex. Experiments in which the results suggested sex differences were expanded with additional mice of each sex to determine whether sex differences were present." There were two statistically phenotypes for which sex was a modifier of phenotype: venule number (fewer in *Jag1*$^{Ndr/Ndr}$ females than *Jag1*$^{Ndr/Ndr}$ males) and P30 venule tortuosity (more tortuous in *Jag1*$^{Ndr/Ndr}$ females than *Jag1*$^{Ndr/Ndr}$ males). See also point 10 Reviewer 1 for further explanation. These statistics were reported in the Figure legends and now are also included in the Source data files. We appreciate that a

summary analysis would be helpful to the reader. We collected all statistical analyses in Appendix Table 1 to provide a simplified overview.

One set of phenotypes, specifically increased VSMC ASMA-negative gaps and VSMC apoptosis seem to be more common in 1-year-old *Jag1*$^{Ndr/Ndr}$ males compared to *Jag1*$^{Ndr/Ndr}$ females. The number of replicates is too small to test statistically. Unfortunately, we have been unable to collect additional 1-year old mice, within the time frame of this submission and because of the high mortality of *Jag1*$^{Ndr/Ndr}$ mice. It is also difficult to compare mice meaningfully at this age, since we expect that a large proportion of the more severely affected mice will have died before the age of 1 year.

Reviewer #2 (Significance (Required)):

Here the authors report two major findings: 1) there are substantial sex-specific differences in Alagille patients in terms of idiopathic intracranial hemorrhage, one of the major cause for death in these young patients. This is a very interesting finding. 2) The authors further characterize their Jag1 Ndr mice. Now as homozygous Ndr/Ndr and with much greater and detailed focus on the vascular phenotype. This gives a number of interesting findings which validate this transgenic mouse as an excellent Alagille model system. In addition, the authors report that also in mice they observed some sex-specific differences in the vascular phenotype.

The strengths of the manuscript are obvious: the sex-specific differences are very interesting and will inspire future research. Secondly, the characterization of the Jag1 Ndr/Ndr mice show that this is an excellent Alagille model system.

The weakness of this paper is that we do not learn about any new mechanistic insight. Neither a potential reason for the sex-specific differences not in the vascular pathogenesis.

We thank the reviewer for identifying the strengths and weaknesses of the manuscript. We agree that this manuscript focuses on reporting a new mouse model for disease, and we also show that it predicted phenotypes that could be observed in patient retinas non-invasively, which we hope will be clinically useful. As the reviewer also points out in their earlier comments, this manuscript could have been two manuscripts – there is a wealth of new insights, and we think this could be an important model for pre-clinical research.

Reviewer #3 (Evidence, reproducibility and clarity (Required)):

In this paper, authors aimed at identifying risk factors of cerebral bleeding in the Alagille

syndrome. They first performed a literature review of cerebral bleedings in patients with Alagille syndrome (ALGS). They then screened a mouse model of ALGS for cerebral bleeding and vascular defects. They further analyzed the retina of patients with ALGS and or CADASIL, looking for vascular defects. The authors conclude that retinal vessel abnormalities (arteriovenous crossings, reduction in blood vessel numbers and increase vessel tortuosity) are risk factors of cerebral bleeding in ALGS patients that can be detected non-invasively by looking at the retinal fundus.

We thank the reviewer for summarizing our work. We would like to clarify that by "risk factors" we were referring to skull thickness and coagulopathy, both of which have been implicated in brain hemorrhages in children with ALGS, and female sex (which we report here as a potential risk factor). The endothelial cell defects and vascular smooth muscle cell defects that we report we consider to be the pathology itself, that may underlie the bleeding, but are not technically a "risk factor". As such, the arteriovenous crossings, reduction in blood vessel numbers and increased vessel tortuosity could instead serve as biomarkers (but will have to be studied in larger cohorts including patients with bleeding events).

The literature review was a Systematic Review, following PRISMA guidelines.

We have revised the text to clarify the difference between risk factors and potential biomarkers.

1. Unfortunately, this conclusion is not supported by the results. First, characterization of the bleeding phenotype in the ALGS mouse model is extremely rudimentary and abnormalities of cerebral vessels underlying cerebral hemorrhage have not been identified.

The neural retina is the state-of-the-art tissue for analysis of vascular development and homeostasis in the nervous system, as it can be flattened and systematically imaged and analyzed. To address whether defects identified in the retina are also present in the brain, we have now collected brains from P10 $Jag1^{+/+}$ and $Jag1^{Ndr/Ndr}$ mice (n=6 per genotype), and analyzed brain vasculature, specifically the middle cerebral artery (MCA), since patients with ALGS have been reported with MCA aneurysms (Kamath et al Circulation. 2004 and Emerick et al J Pediatr Gastroenterol Nutr. 2005). We measured MCA tortuosity, which was increased in one male and one female $Jag1^{Ndr/Ndr}$ mouse (Fig 3 J, 3K) and distances between MCA branches (Fig 3 L) that was again greater in one male and one female $Jag1^{Ndr/Ndr}$ mouse. Further, we quantified the number of ASMA+ vascular smooth muscle cells around the MCA and detected a significant decrease in $Jag1^{Ndr/Ndr}$ mice (Fig 4 K, 4L). The reduction of VSMCs in brain is similar to the reduction seen in the retina. We also stained and imaged brains from older mice, but unfortunately these experiments were too not possible to analyze due to the

large size of the tissue and antibody penetration issues, precluding analysis of later stages or experiments with Angiotensin.

2. Second, at no time did the authors show any correlation between arteriovenous crossings, reduction in blood vessel numbers and increase vessel tortuosity in the retina and the occurrence of cerebral bleeding.

We agree with the reviewer that this is a limitation of the study, and acknowledge and discuss this in the discussion.

The brain bleeds are observed in dead or dying mice, which confounds any analysis of blood vessels, since these may be degrading due to the death of the animal (although we expect arteriovenous crossings to not be affected by the death of the animal). Indeed, correlating bleeding events (observed in 2-3% of *Jag1*$^{Ndr/Ndr}$ mice, a genotype of which 50% survives to P10, and only 25% to adulthood) would be extremely challenging. This question could be addressed in patients or mice with non-invasive imaging of retina and follow-up of bleeding events, which we intend to pursue after this manuscript is published and such a plan can be justified to funders and ethical boards.

3. Third, the observation that these abnormalities are present in the vast majority of mutant mice whereas less than 5% of the mutant mice develop cerebral bleedings belies their conclusion.

While we were able to identify a gross intracranial bleed in 2 of 83 *Jag1*$^{Ndr/Ndr}$ mice, 75% of *Jag1*$^{Ndr/Ndr}$ mice die between P0 and 3 months (50% of which in the first 10 days, Fig 2A, and Andersson et al Gastroenterology 2018). Because the mother often cannibalizes the young, and because many organ systems are disrupted in the mouse model (in particular cardiovascular defects and liver defects) it is not possible for us to determine cause of death for the majority of the mice. It is possible that vascular defects contribute to some of the death of the 75%, beyond the 5% that could be identified prior to death or very soon after death, prior to cannibalization.

The statement that the abnormalities are present in the majority of the homozygous mice, and therefore cannot be causative of the sporadic bleeds, does not take into account the spread of the data and instead focuses on the summary statistical analysis. While two groups can be significantly different (for example with regards to venous tortuosity at P30, Fig 3G, 3H), there is a non-negligible spread in the data for the *Jag1*$^{Ndr/Ndr}$ mice. Jag1+/+ mice exhibit a low venous tortuosity (range 1-2.5%) while *Jag1*$^{Ndr/Ndr}$ mice range from 3% - 10.5%. It is unlikely that 3% is biologically meaningfully different from 2.5%, but the one *Jag1*$^{Ndr/Ndr}$

mouse with 10.5% tortuosity has four-fold longer-than-necessary vasculature than the longest Jag1+/+ mouse, and this mouse with greater vascular pathology is likely at greater risk of vascular accidents. Future studies can build on our results with non-invasive vascular imaging of pups and follow these for vascular outcomes.

4. Another major problem with this paper is that many statements are not supported by experimental data or statistical analysis.

We have added quantifications and statistical analysis for every experiment performed. Every quantification and resulting graph were analyzed for Statistical significance, using stringent statistical analysis, as described in the Materials and Methods and Source Data (both for significant and non-significant differences).

We also removed any statements that are not supported by data.

Other major concerns

5. Abstract and introduction: what is the rationale to look for a gender effect in the occurrence of cerebral bleeding in patients with ALGS? I could not find any explanation in the introduction or in the literature.

Thank you for pointing out this omission. In editing to reduce character count, the rationale was only explained in the Discussion "(subarachnoid hemorrhage/SAH) … (is).. more common in men between age 25-45, and in women between age 55-85. The greater risk of SAH in women over 55 has been speculated to relate to hormonal differences after menopause, but hormone replacement therapy has yielded mixed results. Patients with ALGS may also present with hormonal differences, which are as yet poorly characterized: puberty is delayed in some patients with ALGS, and some are non-responsive to growth hormone"). Furthermore, crosstalk between Notch and the androgen pathway, acting via Hey1 (Belandia et al Mol Cell Biol 2020) may entail different outcomes for loss of Notch signaling in males and females."

We agree that for clarity the relevance of sex should indeed be explicitly stated in the introduction and Results section. We now include a short introduction to the relevance of sex in intracranial bleeds in the introduction: "Furthermore, although no sex differences have been reported in ALGS, female sex is a risk factor for prevalence of intracranial aneurysms, aneurysm growth, and subarachnoid hemorrhage". And we include context in the results section: "Sex is not thought to impact the prevalence of vascular defects or intracranial bleeds in ALGS [3,24,25]. However, sex-based differences in cardiovascular disease, stroke, and intracranial bleeds in the general population have been reported [26,27]."

6. Results, first section and Figure 1: The classification of cerebral bleedings is not appropriate and all the most surprising: hematoma and hemorrhages are used in clinics to design the same thing; idiopathic means with an unknown mechanism, therefore a subarachnoid hemorrhage caused by the rupture of an aneurysm cannot be classified as "idiopathic". Conversely, a minor trauma can be a triggering factor but cannot be considered as the cause of a hemorrhage.

The definitions of hematoma and hemorrhage are different and generally refer to a smaller delimited bleed that has usually stopped (hematoma, can still be lethal in brain) or a larger/ongoing bleed (hemorrhage). However, if the terms are ambiguous to clinicians, and if they risk being used interchangeably by the authors of the publications on which our review is based, we are grateful for this feedback and we instead refer to "intracranial bleeds (hematoma or hemorrhage)" for data in Fig 1B, and we removed the first two Venn diagrams. We kept the definitions in Table 1, since the designation of bleeds as hematoma or hemorrhage is based on the original authors' descriptions of the bleeds. Regarding the use of the term "idiopathic" and underlying mechanisms, this hinges on what one considers to be an underlying cause. While a ruptured aneurysm in a young child is considered by the reviewer to be the "cause" of the bleed, our view is that an aneurysm in a child is an anomaly, which is the result of another insult that has not yet been defined (e.g., weakened blood vessels due to JAG1 insufficiency). An aneurysm cannot be the primary cause – since something led to the aneurysm. To reconcile our viewpoints, we renamed the classifications to focus on precipitating factors: "Coagulopathy, Minor trauma, and Spontaneous".

7. Results, second section (lines 113-151) and Figure 2: Characterization of the bleeding phenotype in the ALGS mouse model is extremely rudimentary. Whereas cerebral hemorrhages in patients with ALGS can occur in the adult, only pups from P0 to P10 were analyzed.

We analyzed and reported brain bleeds in P0-P10 mice (Fig 2M and text) as well as adults (Fig 2L and Fig 4P, 4Q). We have observed mortality in adult $Jag1^{Ndr/Ndr}$ mice, but we were unable to determine the cause of death. See also response to comment 1.

8. Screening was limited to a macroscopic analysis of the brain which is unlikely to capture intracerebral hemorrhages and as a result the true prevalence of cerebral bleeding is probably underestimated. More importantly, the origin and the cause of cerebral bleeding were not investigated.

Analysis of brain bleeding included macroscopic brain analysis (Fig 2M, Fig 4Q), and Evans blue leakage analysis (Fig 2L), as well as testing whether increasing blood pressure with AngII worsened Evans Blue leakage (Fig 4P). We have now also added analysis of MCA architecture and SMC coverage, as well as extended blood brain barrier permeability assays with small fluorescent tracers (for details see response to comment 1).

9. Moreover, it is said that Jagged 1 mutant mice exhibit "provoked hemorrhage". I could not find the experimental data supporting this statement.

We apologize for this being unclear. The resin injections presented in Fig 2J, 2K, represent provoked portal vein hemorrhages. They occur, in the *Jag1*$^{Ndr/Ndr}$ mice, in the setting of increased intravascular pressure with resin injections.

10. Also, the statement that "thinner skulls in *Jag1*$^{Ndr/Ndr}$ mice likely contributed to nervous system bleeds" is not supported by any experimental data.

Testing this would require inducing trauma to the skull to assess whether the thinner bones more easily break and lead to brain bleeds. We do not consider this experiment ethical, and our ethical review board would be unlikely to approve the study. This sentence is meant as a statement summarizing the conclusions but was not meant to represent a result (hence the use of the term “likely”). We rephrased to “As described in some patients with ALGS and intracranial hemorrhage, thinner intracranial bones in *Jag1*$^{Ndr/Ndr}$ mice may also contribute to nervous system bleeds.”

11. Results, fourth section, figure 4 and supplementary fig 4. It is said that pericytes in the retinal capillaries were not reduced but supplementary fig 4 does not show any quantification.

We thank the reviewer for focusing on this interesting point. We have added more replicates to this analysis and quantified CD13 coverage. Overall, there was no difference in CD13 staining between *Jag1*$^{+/+}$ and *Jag1*$^{Ndr/Ndr}$ mice (EV4A, EV4B), confirming our initial conclusion.

12. It is said that VSMC were less mature in mutant mice, please explain.

VSMCs are very plastic cells that respond to changes in their environment. Developmentally, they differentiate from a proliferative, migratory “synthetic” phenotype to a quiescent, non-migratory “contractile” phenotype. The mature VSMCs wrap around the arteriole, while the immature VSMCs exhibit a fibroblast-like appearance (Owens et al, Physiological Review 2004). We intended the term “less mature” VSMCs in *Jag1*$^{Ndr/Ndr}$ mice to refer to the migratory and disorganized cellular morphology, rather than marker status since the VSMC are ASMA positive). We have adjusted the text to “At P10, α-smooth muscle cell actin positive (αSMA+) VSMC morphology in *Jag1*$^{Ndr/Ndr}$ retinas was more migratory and less mature with some cell bodies oriented parallel to the blood vessel axis rather than perpendicular (Fig 4A top panel, yellow arrows, magnified in inset). At P15, *Jag1*$^{Ndr/Ndr}$ VSMC arteriole coverage was incomplete

and parallel-oriented VSMCs were still present (Fig 4B yellow arrows, boxed region). At P30, *Jag1*$^{Ndr/Ndr}$ arteriolar VSMCs exhibited a more mature morphology with mostly perpendicular VSMCs, but with sparse coverage and occasional parallel orientation (Fig 4C yellow arrows)".

We also added staining for synthetic VSMC as per comment 2 Reviewer 2 to further corroborate/test this claim (Fig EV4).

13. The finding that *Jag1*$^{Ndr/Ndr}$ mice display reduced VSMC coverage is not entirely new given that the ndr mutation is an hypomorphic mutation and that mural cell coverage has been reported to be reduced in Jagged1 KO mice (Benedito et al, Cell 2009). The statement that gaps in VSMC coverage are exacerbated by aging are not supported by quantifications.

Indeed, Rui Benedito and Ralf Adams' Cell paper provided numerous new groundbreaking insights into the role of Jag1 and Dll4 in angiogenesis. Their analysis of mural cell coverage was based on low-magnification images of retina in which individual VSMCs could not be identified (and the phenotype was not quantified), and thus it was not clear what the reduction in staining reflected (fewer VSMCs, less intensely stained VSMCs?) In our manuscript, we show high-resolution high-magnification images demonstrating both an absence of contact points between VSMCs in adult mice (Fig 4H, Fig EV4C), and larger gaps in VSMC coverage in 1 year old mice (Fig 4E, 4I), which were not previously reported in any Jag1 mouse model. We have now quantified the gaps in VSMC coverage in P30, 3-6 month and 1 year old adults (Fig 4F, 4G), and show that ASMA-gaps were absent at P30, but present in 30% of 3-6 months old and increased to 50% of 1 year old *Jag1*$^{Ndr/Ndr}$ mice.

Regarding novelty and different models: we have now analyzed the VSMC phenotype of *Jag1*$^{\Delta DSL/+}$ mice, which model the liver disease of Alagille syndrome (Thakurdas et al 2016). We include these data in a new Appendix Fig 2. Our analyses show that *Jag1*$^{\Delta DSL/+}$mice display a similar phenotype to *Jag1*$^{Ndr/Ndr}$ mice with fewer arteries and veins, and increased arteriovenous crossings. *Jag1*$^{\Delta DSL/+}$ also display overall weaker ASMA staining, but no small or large gaps between VSMCs. The *Jag1*$^{\Delta DSL/+}$ mice thus exhibit a milder phenotype than *Jag1*$^{Ndr/Ndr}$ mice.

14. In the AngII experiment (panel H), blood pressure measurements are not shown. By the way, pooling mice with age ranging from 3 to 7.5 months in a mouse model with an age-dependent phenotype seems counterintuitive.

Yes, we agree, if it would be possible to breed, obtain and keep matched sets of *Jag1*$^{Ndr/Ndr}$ mice at the same age this would be the best approach and reduce variability in the data. Unfortunately, we had to balance synchronizing the minipump surgeries and blood pressure measurements with mating and obtaining surviving mice. Only one fifth of the 25% of homozygous mice survive to adulthood. Thus, in order to reach n of 8 *Jag1*$^{Ndr/Ndr}$ mice in the

Ang II treated group (of which one died and two did not exhibit an increase in blood pressure, as specified in Materials and Methods), 40 *Jag1*$^{Ndr/Ndr}$ mice had to be generated (as well as 120 mice of other genotypes). Unfortunately, the surviving mice were from a range of ages.

Nonetheless, despite these limitations that we agree could confound analysis, the data clearly demonstrate that an AngII-induced increase in blood pressure leads to an ASMA gap phenotype in all *Jag1*$^{Ndr/Ndr}$ mice (Fig 4R and 4S), and in fact the phenotype is worse than in 1-year-old mice (in which 50% of *Jag1*$^{Ndr/Ndr}$ mice display gaps, Fig 4F, 4G), suggesting that increased blood pressure is more detrimental than increasing age. We now include the non-treated mean blood pressure measurements before induction of AngII (Fig 4N) and systolic blood pressure values for before and during AngII treatment (Fig 4O).

15. Results, fifth section, Figure 5 (mislabeled figure 6). It is almost counterintuitive to have a reduced density in the ICP and a lower number of vertical sprouts in 3-6 months old mice and a normal density at 1 year.

Thank you for pointing out the mislabeling, now corrected.

The differences in vascular length at 3-6 months and one year (Fig 5G and EV5C) are minor, and generally the wild type and *Jag1*$^{Ndr/Ndr}$ data overlap. However, the difference between the 3-6 month and 1-year branching point and vertical sprouting data (former Fig 5H, K, L, M and EV5D) is that the *Jag1*$^{Ndr/Ndr}$ retina at 3-6 months resembles a 1-year-old *Jag1*$^{+/+}$ retina. But the *Jag1*$^{Ndr/Ndr}$ ICP capillary vascular length or branching point/vertical sprouting phenotypes do not worsen between 3-6 months and one year. This explains why there is a difference between *Jag1*$^{+/+}$ and *Jag1*$^{Ndr/mice}$ at 3-6 months but not at one year – they converge. However, the intermediate capillary plexus in 1-year-old *Jag1*$^{Ndr/Ndr}$ mice contains disorganized branches with gaps in the ICP layer (Fig 5K in manuscript, white arrowheads), which are not present in similar views of 3-6 months adult *Jag1*$^{Ndr/Ndr}$ mice. These data show that specific aspects of the capillary phenotype worsen with age, which were not captured by vascular length or branchpoint analysis. We now clarify this in the results section.

Another challenge in interpreting these data is that detrimental phenotypes associated with the death of the mouse will be selected against by the accumulating deaths in the *Jag1*$^{Ndr/Ndr}$ cohort, meaning that mice analyzed at 1 year will be the healthiest possible *Jag1*$^{Ndr/Ndr}$ mice. This is also now clarified and discussed in the discussion.

16. Again, the statement that the RGC axons are healthy in *Jag1*$^{Ndr/Ndr}$ retinas at P10 is not supported by quantification. Also, the authors show a reduction in the number of RGC axons at P40. Whereas they document VSMC degeneration much later, they claim that the onset of vascular degeneration was associated with RGC degeneration.

To improve the analysis of RGC and the link to vascular degeneration, we have now quantified the RGC axonal phenotype at P10, which showed no statistical difference, as suggested by the images. We further extended the analysis of retinal vasculature to P30 (Fig 4C, 4F, 4G, 5A-5D), demonstrating a decrease in VSMC coverage, reduced vessel density and a reduction in the number of branching points in the ICP in $Jag1^{Ndr/Ndr}$ mice at this stage. The retinal endothelial cell defects observed in P30 $Jag1^{Ndr/Ndr}$ mice are similar to those at the 3-6 months stage. These new findings strengthen our claims that the RGC phenotype is related to vascular degeneration.

Regarding VSMCs: these are not normal at any stage investigated in the $Jag1^{Ndr/Ndr}$ mice, from P10 onwards (Fig 4A-G).

17. Results, sixth section, figure 6. The rationale to include in the analysis of the retinal fundus CADASIL patients is far from obvious to this Reviewer and does not bring anything. Indeed, whereas ALGS is a developmental disease, CADASIL is a degenerative disease. Moreover, patients with AGLS exhibit cerebral bleeding, whereas cerebral bleeding in patients with CADASIL is extremely rare.

Our data suggest that ALGS vascular pathology includes both developmental and degenerative aspects. Because VSMCs are abrogated both in *Jag1* loss of function and *Notch3* loss of function mouse models, we propose that the VSMC phenotype in the ALGS mouse model is likely due to Jag1-Notch3 signaling defects. Although multiple consequences of *NOTCH3* mutations have been described in CADASIL (most often toxic gain of function), it has been also proposed that reduced NOTCH3 signaling can contribute to the VSMC phenotype in CADASIL (and indeed, a CADASIL-like patient was reported with homozygous *NOTCH3* loss of function and paucity of VSMCs but no GOMs, Pippucci et al EMBO Mol. Med. 2015, and recently reviewed by Samira Hosseini-Alghaderi and Martin Baron, Biomolecules 2021). We therefore considered it of interest to include CADASIL patients in the analysis. Importantly, the presence of multiple vascular defects in ALGS patients but not CADASIL patients suggests that tortuosity, for example, is related to endothelial cell defects (present in Jag1 mutant conditions) rather than VSMC defects (present in both Jag1 and Notch3 mutant conditions). To clarify the rationale, we now provide this context in the beginning of this results section.

Also of note, patients with ALGS exhibit both intracranial bleeding and ischemic events, though the frequency of each has not yet been reported. We have chosen to focus on the intracranial bleeding events in this manuscript.

Reviewer #3 (Significance (Required)):

The Alagille syndrome is a developmental disease primarily affecting the liver, caused by hypomorphic or loss of function in the Jagged1 ligand or more rarely in the Notch2 receptor. Nevertheless, cerebral hemorrhage in patients with ALGS is of clinical significance, causing up to 25% of deaths. Because the penetrance of these hemorrhages is incomplete, identifying risk factors would constitute a significant advance.

Unfortunately, retinal vessel abnormalities (arteriovenous crossings, reduction in blood vessel numbers and increase vessel tortuosity) identified in patients or mice with ALGS in this study are unlikely to be risk factors for cerebral hemorrhage for the reasons mentioned in the previous section.

We do not suggest that retinal arteriovenous crossings, reduction in blood vessel numbers and increase vessel tortuosity are risk factors for cerebral hemorrhage, rather that they could be used as biomarkers indicating the presence of compromised vasculature in brain. Such a correlation would be worth investigating in future studies, but a large-scale preclinical investigation of this cannot be justified in humans before showing that patients have defects in retina (this manuscript). Vessel tortuosity is a well-established indicator of reduced wall elasticity which can lead to bleeds; aberrant artery-vein crossings indicate incorrect vascular pathfinding that could lead to local hypoxia in regions with fewer blood vessels or with compressed vessels, and similarly reduced numbers of blood vessels would limit vascularization and support of the tissue.

As mentioned above, we now show that VSMC paucity is also present in brain vasculature in $Jag1^{Ndr/Ndr}$ mice, as early as P10 (Fig 4K).

Expertise of this Reviewer encompasses clinical neurology, the physiology and pathology of the cerebroretinal vasculature and the Notch signaling pathway.

8th Sep 2022

Dear Dr. Andersson,

Thank you for the submission of your manuscript to EMBO Molecular Medicine and please accept my apologies for the unusual delay in getting back to you, which is due to the fact that the reports came back when I was on annual leave and that I also further consulted with the referees on their reports.
As you will see from the enclosed reports, while referees #1 and #2 are satisfied with the revisions, referee #3 still raises a few concerns on your study. Following internal discussion with my colleagues and further consultation with the referees, we decided that no additional experiment would be needed at this point. However, in a revised version of your manuscript, it will be important to address the remaining concerns from referee #3 in writing. In particular, this referee mentioned:
"I will be very vigilant with regards to the following points:
1) Vascular defects have been exclusively characterized in the retina and this should be clearly indicated in the abstract.
2) The study has 2 important limitations which should be explicitly acknowledged: the origin and cause of cerebral bleedings have not been identified; the relationship between the presence of retinal vascular defects and the occurrence of bleedings is unclear."

Moreover, kindly address the editorial issues listed below.

1/ Main manuscript text:
- Please address the queries from our data editors in track changes mode in the main manuscript file labelled 'Data edited MS file'. Kindly use this file for any further modification, accept the changes and only keep in track changes mode any new modification.
- Material and methods: Human samples: include the complete statement that the experiments conformed to the principles set out in the WMA Declaration of Helsinki and the Department of Health and Human Services Belmont Report.
- Please place the Data availability section after the Material and Methods section. Please remove the sentence "Source Data are provided with each figure".
- Author contribution: CRediT has replaced the traditional author contributions section because it offers a systematic machine-readable author contributions format that allows for more effective research assessment. Please remove the Authors Contributions from the manuscript and use the free text boxes beneath each contributing author's name in our system to add specific details on the author's contribution. More information is available in our guide to authors.
- Please merge the Funding with the Acknowledgement section.
- Conflict of interest: We updated our journal's competing interests policy in January 2022 and request authors to consider both actual and perceived competing interests. Please review the policy https://www.embopress.org/competing-interests and update your competing interests if necessary. Kindly also update the title of this section to "Disclosure and competing interests statement".
- References should be listed in alphabetical order, with 10 authors before et al.

2/ Figures and Appendix:
- Please provide exact p values directly in the EV figures and Appendix figures (or in their legends).
- Appendix: please correct the nomenclature to Appendix Figure S1, Appendix Tables S1, etc. Please add the Supplementary Material and Methods to the Appendix file. Please make sure all figures/tables are properly referenced in the text.
- Please zip a legend to each movie file.
- Figure callouts: "Data 1" is mentioned in the manuscript text, but no corresponding dataset.

3/ Source Data:
Thank you for providing Source Data. Please make sure the files are correctly labelled (i.e. figure 2).

4/ Every published paper now includes a 'Synopsis' to further enhance discoverability. Synopses are displayed on the journal webpage and are freely accessible to all readers. They include a short stand first (maximum of 300 characters, including space) as well as 2-5 one-sentences bullet points that summarizes the paper. Please write the bullet points to summarize the key NEW findings. They should be designed to be complementary to the abstract - i.e. not repeat the same text. We encourage inclusion of key acronyms and quantitative information (maximum of 30 words / bullet point). Please use the passive voice. Please attach these in a separate file or send them by email, we will incorporate them accordingly.

Please also suggest a striking image or visual abstract to illustrate your article as a PNG file 550 px wide x 300-600 px high.

5/ As part of the EMBO Publications transparent editorial process initiative (see our Editorial at http://embomolmed.embopress.org/content/2/9/329), EMBO Molecular Medicine will publish online a Review Process File (RPF) to accompany accepted manuscripts.
This file will be published in conjunction with your paper and will include the anonymous referee reports, your point-by-point

response and all pertinent correspondence relating to the manuscript. Let us know whether you agree with the publication of the RPF.
Please note that the Authors checklist will be published at the end of the RPF.

I look forward to receiving your revised manuscript.

Yours sincerely,

Lise Roth

Lise Roth, PhD
Senior Editor
EMBO Molecular Medicine

***** Reviewer's comments *****

Referee #1 (Remarks for Author):

The authors have sufficiently addressed all of my concerns. I agree that high bilirubin in blood plasma interferes in the analysis of permeability. As such, the manuscript will make a strong contribution in the ALGS field.

Referee #2 (Remarks for Author):

The authors have done an admirable job in addressing the comments raised by this and other reviewers. Even when there was a choice between rephrasing and performing additional experiments, the authors often chose to do additional experiments. They have thoroughly revised the text as well. The only suggestion I have for the authors is to make the usage of circle (F) and square (M) legends uniform across the figures. Currently, many panels in Figure 2 have these labels but most other figures don't have them. It might be better to show it at least once in each figure that uses circles and squares.

Referee #3 (Comments on Novelty/Model System for Author):

In this paper, authors aimed at identifying risk factors of cerebral bleeding in the Alagille syndrome. Although the paper has been extensively revised, major issues raised in the first reviewing have not been addressed. In particular, characterization of the

bleeding phenotype in the brain of this ALGS mouse model is still rudimentary. Screening was limited to a macroscopic analysis of the brain which is unlikely to capture intracerebral hemorrhages. Moreover, the origin and cause of cerebral bleeding have not been thoroughly investigated and there is no convincing analysis of cerebral vessels. This could have been easily done by conducting an histological analysis of the brain at P0, P5 and P10.
Although the authors provide an extensive descriptive analysis of vascular defects in the retina, they did not attempt to establish any relationship between the presence of these defects and the occurrence of bleeding. Therefore, the medical impact of identifying vascular defects in the retina of mice or patients with ALGS is extremely limited if one cannot correlate the presence of these defects with the bleeding risk.
In sum, I'm not convinced that the goals of this paper have been achieved.

Referee #3 (Remarks for Author):

In this revised version, the authors have performed additional studies and the paper has been significantly improved. However, there are still major issues.
First, the origin and the cause of cerebral bleeding have not been thoroughly investigated. Second, there are still no convincing data showing that vascular defects, similar to those observed in the retina are observed in the brain of Jagged 1 mutant mice. On figure 3K, I cannot see any defect in SMC coverage in the MCA. Moreover, immunostaining should also include either a marker of endothelial cells or basement membrane to ensure that the potential discontinuity in the SMA staining is not artefactual. Third, although the authors provide an extensive descriptive analysis of vascular defects in the retina, they did not attempt to establish any relationship between the presence of these defects and the occurrence of bleeding. These limitations should be explicitly acknowledged in the paper, and in the abstract, it should be stated that vascular defects have been detected in the retina.

Other comments
In Table 1, please remove idiopathic and replace by spontaneous. Clinically speaking, an hemorrhage caused by the rupture of an aneurysm cannot be considered as an idiopathic hemorrhage, even though the authors think the opposite.
Page 10, line 234, "VSMC morphology was more migratory". A migratory phenotype cannot be evaluated by a simple immunostaining. Please correct.
Page, 11, lines 278-279 " Jag1Ndr/Ndr arterial VSMC exhibited disorganized cellular orientation, with fewer VSMCs per area (Fig 4K, 4L)". The figure is not convincing and there is not any quantification. Please correct accordingly.
Page 14, lines 345 "mice bearing CADASIL mutant NOTCH3 display sparse VSMCs". This statement is incorrect and to the best of knowledge of this Reviewer, SMC defects in CADASIL mice have never been reported. Indeed, in the paper quoted by the authors (reference 50), mice identified as "CADASIL mice" are mice expressing a CADASIL loss-of-function Notch3 mutation backcrossed on a Notch3 null background. Therefore, the phenotype of these mice is merely a Notch3KO phenotype.
Discussion, lines 396-397, "However, sporadic vascular events in Jag1Ndr/Ndr mice may also contribute to, or be correlated with, the sporadic bleeds". What do you mean? Please clarify
Discussion, "These vascular events were sporadic and could not be statistically evaluated". Please rephrase, the number of mice analyzed in this study was too small to conduct statistical analysis.
Discussion, "sporadic sex-specific events" What do you mean, please clarify
Discussion, lines 428-431 "The retinal vascular abnormalities detected in ALGS retinographs were not present in CADASIL, suggesting vascular tortuosity (identified in both ALGS and the mouse model) arises from EC (JAG1) defects rather than being secondary to VSMC (JAG1-NOTCH3) defects". I unfortunately believe that this interpretation is erroneous. The finding that CADASIL patients do not exhibit retinal vascular abnormalities detected in ALGS retinographs may be due to the fact that CADASIL mutations are not loss of function mutations, which is a very likely possibility. It might have been wiser to compare the phenotype of Jagged 1 mutant mice and Notch3KO mice.
Discussion, page 17, lines 439-440, "vessel tortuosity........... is associated with ischemic stroke". This statement should not be taken for granted since it has been reported in a single paper. It would be fairer to delete this sentence, especially as the present paper deals with brain hemorrhage and not ischemic stroke.

EMM-2022-15809-V2 Revision Round 2, Minor Revision

Thank you for the opportunity to revise this manuscript to address feedback from reviewer 3.

We updated the Abstract and Discussion as instructed in editorial comments, emphasizing that the study was mostly undertaken in retina, and acknowledging the limitations. We agree with the publication of the RPF.

Referee #1 (Remarks for Author):

The authors have sufficiently addressed all of my concerns. I agree that high bilirubin in blood plasma interferes in the analysis of permeability. As such, the manuscript will make a strong contribution in the ALGS field.

Thank you for these kind comments as well as the previous feedback.

Referee #2 (Remarks for Author):

The authors have done an admirable job in addressing the comments raised by this and other reviewers. Even when there was a choice between rephrasing and performing additional experiments, the authors often chose to do additional experiments. They have thoroughly revised the text as well. The only suggestion I have for the authors is to make the usage of circle (F) and square (M) legends uniform across the figures. Currently, many panels in Figure 2 have these labels but most other figures don't have them. It might be better to show it at least once in each figure that uses circles and squares.

Thank you, and we appreciate you pointing out this accidental omission. We have updated all applicable figures (Fig 3,4,5,6, Fig EV2,3,4,5, and Appendix Fig S2) with this legend.

Referee #3 (Comments on Novelty/Model System for Author):

In this paper, authors aimed at identifying risk factors of cerebral bleeding in the Alagille syndrome. Although the paper has been extensively revised, major issues raised in the first reviewing have not been addressed. In particular, characterization of the bleeding phenotype in the brain of this ALGS mouse model is still rudimentary. Screening was limited to a macroscopic analysis of the brain which is unlikely to capture intracerebral hemorrhages.

The brain analysis included macroscopic analysis, 3D imaging of whole brain vasculature, and quantitative tracer analyses of three different molecular weight tracers. We believe that the quantitative approaches taken here would capture intracerebral hemorrhages. In total, tracers were used in 25 *Jag1*$^{Ndr/Ndr}$ mice, at P30 and adult stages, identifying a few sporadic outliers with slightly higher tracer amounts in brains of *Jag1*$^{Ndr/Ndr}$ mice. The data suggest that spontaneous intracranial bleeds are rare, but do occur, in *Jag1*$^{Ndr/Ndr}$ mice.

Specifically, brain analyses included:

- Macroscopic analysis of brain, and quantification of Evans blue in brain lysates from 7 *Jag1*$^{+/+}$ and 8 *Jag1*$^{Ndr/Ndr}$ mice, to investigate blood brain barrier and bleeds in physiological conditions

(Fig 2L, 2M), and in brain lysates from 5 *Jag1*$^{+/+}$ and 5 *Jag1*$^{Ndr/Ndr}$ mice after treatment with Angiotensin II to test the impact of increased blood pressure on intracranial bleeds (Fig4P, 4Q).

- Quantification of fluorescent tracers Cadaverin and Dextran in brain lysates from 12 *Jag1*$^{+/+}$ and 12 *Jag1*$^{Ndr/Ndr}$ mice (6 of each sex) to investigate whether smaller-sized tracers would traverse the blood brain barrier or be informative of smaller bleeds (Fig EV2 H,I). Because the mice are cholestatic and bilirubin is auto-fluorescent, it was not possible to accurately measure tracer amounts in serum, but there was no increase in fluorescence in *Jag1*$^{Ndr/Ndr}$ brain samples, suggesting neither bilirubin nor fluorescent tracers leak across the blood brain barrier in the majority of *Jag1*$^{Ndr/Ndr}$ mice in unstressed conditions.
- Whole mount staining for ASMA and 3D imaging of whole brains at P10 in 6 *Jag1*$^{+/+}$ and 6 *Jag1*$^{Ndr/Ndr}$ mice:
  - segmentation of the middle cerebral artery (MCA) for analysis (Fig 3J-L, Fig 4K,L):
    - quantification of vascular tortuosity (Fig 3J,K)
    - quantification of blood vessel branching (Fig 3K,L)
    - quantification of ASMA coverage of the MCA (Fig 4K, L)
    - ➔ This analysis showed that ASMA+ VSMC coverage in brain MCA is significantly reduced in *Jag1*$^{Ndr/Ndr}$ mice, and 2 of 6 *Jag1*$^{Ndr/Ndr}$ mice showed divergent tortuosity and branching defects. This brain analysis thus corroborated VSMC paucity which is consistent in all mutant animals, and more severe defects in tortuosity and branching that are more sporadically present.

Moreover, the origin and cause of cerebral bleeding have not been thoroughly investigated and there is no convincing analysis of cerebral vessels. This could have been easily done by conducting an histological analysis of the brain at P0, P5 and P10.

Although the authors provide an extensive descriptive analysis of vascular defects in the retina, they did not attempt to establish any relationship between the presence of these defects and the occurrence of bleeding. Therefore, the medical impact of identifying vascular defects in the retina of mice or patients with ALGS is extremely limited if one cannot correlate the presence of these defects with the bleeding risk.

In sum, I'm not convinced that the goals of this paper have been achieved.

We agree that analyzing vascularization of the brain would be interesting. Brain vascularization initiates around embryonic day 9.5, and we have previously shown that brain vascularization is compromised in *Jag1*$^{Ndr/Ndr}$ mice at this stage (Hansson et al JCS 2010, Fig 1C). However, in this manuscript we aimed to establish whether there are bleeds and/or vascular defects in postnatal *Jag1*$^{Ndr/Ndr}$ mice, as a possible model for Alagille syndrome. We used the neural retina which is a gold standard in the field for analysis of vascular development and architecture, and we validated the vascular smooth muscle cell paucity in 3D analysis of brain at P10. We also show that 2 of 6 *Jag1*$^{Ndr/Ndr}$ mice exhibit vascular tortuosity and branching defects at P10 in the middle cerebral artery (in the brain).

The rare occurrence of spontaneous bleeding in *Jag1*$^{Ndr/Ndr}$ mice was not possible to test for correlation with the degree of vascular pathology, and we agree that this is a limitation of this study. Unfortunately, mice with de facto bleeds (eg Fig 2M) are unsuitable for retinal vascular analysis since the tissue has started degrading. This is now explicitly acknowledged in the Discussion section:

“Another limitation of the study is that we were unable to identify the exact origin and cause of spontaneous central nervous system bleedings or to test whether retinal vasculopathy correlates with bleeding events, in the limited numbers of mice with bleeds and tissue suitable for analysis. Of note, *Jag1*$^{Ndr/Ndr}$ mice that died between birth and P10 were often cannibalized by the mother and thus not possible to analyze for bleeding events or vasculopathy. Experiments aimed at specifically collecting large cohorts of mice with and without bleeds at matched stages would thus allow for testing correlations.”

Referee #3 (Remarks for Author):

In this revised version, the authors have performed additional studies and the paper has been significantly improved. However, there are still major issues.

First, the origin and the cause of cerebral bleeding have not been thoroughly investigated.

Please see response above, this limitation (not showing a direct link between bleeding and one of the specific phenotypes of the many reported here) has now been acknowledged in Discussion.

Second, there are still no convincing data showing that vascular defects, similar to those observed in the retina are observed in the brain of Jagged 1 mutant mice. On figure 3K, I cannot see any defect in SMC coverage in the MCA.

The close-up and quantification of SMCs in the MCA is presented in Fig 4K, 4L.

Moreover, immunostaining should also include either a marker of endothelial cells or basement membrane to ensure that the potential discontinuity in the SMA staining is not artefactual.

We understand and appreciate this comment, which correctly points out that discontinuity could arise from breaks in the tissue. As can be seen in the overview (Fig 3K) and close-up (Fig 4K) we are not reporting large gaps or discontinuities in VSMC coverage in *Jag1*$^{Ndr/Ndr}$ brain vasculature at P10, rather an overall lower density of VSMCs (Fig 4K, 4L) which is seen/quantified in “intact” vessels with continuous but sparse VSMC coverage at P10.

Third, although the authors provide an extensive descriptive analysis of vascular defects in the retina, they did not attempt to establish any relationship between the presence of these defects and the occurrence of bleeding. These limitations should be explicitly acknowledged in the paper, and in the abstract, it should be stated that vascular defects have been detected in the retina.

We have amended the abstract to clarify that the extensive blood vessel analysis during development and homeostasis was performed in retina. “We investigated vascular development, homeostasis, and bleeding in Jag1Ndr/Ndr mice, using retina as a model.”

We have updated the Discussion to acknowledge that we could not statistically test for a correlation between bleeding and vascular pathology, since bleeds were rare events leading to tissue unsuitable for analysis, as also described above.

Other comments

In Table 1, please remove idiopathic and replace by spontaneous. Clinically speaking, an hemorrhage caused by the rupture of an aneurysm cannot be considered as an idiopathic hemorrhage, even though the authors think the opposite.

Thank you for pointing out that we missed updating the terminology in this Table. This is now corrected, as well as in Source Data.

Page 10, line 234, "VSMC morphology was more migratory". A migratory phenotype cannot be evaluated by a simple immunostaining. Please correct.

We have removed this statement.

Page, 11, lines 278-279 " Jag1Ndr/Ndr arterial VSMC exhibited disorganized cellular orientation, with fewer VSMCs per area (Fig 4K, 4L)". The figure is not convincing and there is not any quantification. Please correct accordingly.

The VSMC number quantification/graph was/is presented in Fig 4L, with close-ups of the MCA in Fig 4K, in addition to the whole MCA overview in Fig 3K, showing that there is a significant decrease in VSMC coverage. If the reviewer was referring to quantification of the phenomenon of “disorganization”, we now omit this word.

Page 14, lines 345 "mice bearing CADASIL mutant NOTCH3 display sparse VSMCs". This statement is incorrect and to the best of knowledge of this Reviewer, SMC defects in CADASIL mice have never been reported. Indeed, in the paper quoted by the authors (reference 50), mice identified as "CADASIL mice" are mice expressing a CADASIL loss-of-function Notch3 mutation backcrossed on a Notch3 null background. Therefore, the phenotype of these mice is merely a Notch3KO phenotype.

There are multiple reports showing SMC defects in various CADASIL-mutant mice, in addition to the C455R mutant we cited, we now include references to the following two mouse models:

R1031C NOTCH3 mice: Arboleda-Velasquez JF et al 2011 describe VSMC thinning in aorta of R1031C Notch3 mice (their Fig 6). (Hypomorphic Notch 3 alleles link Notch signaling to ischemic cerebral small-vessel disease. Proc Natl Acad Sci U S A. 2011)

R90C NOTCH3 mutant mice: Gaps between VSMCs, specifically increase in Subendothelial Space and Intersmooth Muscle Cell Space in Tail Arteries (Their Table 1). (Ruchoux MM, Domenga V, Brulin P, Maciazek J, Limol S, Tournier-Lasserve E, Joutel A. Transgenic mice expressing mutant Notch3 develop vascular alterations characteristic of cerebral autosomal dominant arteriopathy with subcortical infarcts and leukoencephalopathy. Am J Pathol. 2003 Jan;162(1):329-42. doi: 10.1016/S0002-9440(10)63824-2. PMID: 12507916; PMCID: PMC1851116.)

We modified the sentence as follows to better explain the functional context of each NOTCH3 variant:

“Since Notch3 knockout mice (Henshall et al, 2015b), and mice bearing CADASIL loss-of-function — NOTCH3^{C455R} (Machuca-Parra et al, 2017a) or a signaling-competent CADASIL-NOTCH3^{R90C} (Ruchoux et al, 2003) display sparse VSMCs or gaps similar to that identified in *Jag1*$^{Ndr/Ndr}$ mice (Fig 4A-E), and mice bearing CADASIL-mutant NOTCH3^{R1031C} similarly exhibit VSMC thinning (Arboleda-Velasquez et al, 2011), we considered it of interest to include CADASIL patients in this analysis.”

Discussion, lines 396-397, "However, sporadic vascular events in Jag1Ndr/Ndr mice may also contribute to, or be correlated with, the sporadic bleeds". What do you mean? Please clarify

Here, we aimed to address the fact that there are:

1. Highly penetrant vascular defects in *Jag1*$^{Ndr/Ndr}$ mice
2. Sex-dependent vascular defects in *Jag1*$^{Ndr/Ndr}$ mice
3. Sporadic vascular defects in *Jag1*$^{Ndr/Ndr}$ mice

As the reviewer previously pointed out, the highly penetrant vascular defects cannot directly cause the bleeds because then all *Jag1*$^{Ndr/Ndr}$ mice would exhibit bleeds. However, even the statistically significant highly penetrant defects show a range of pathology. For example, *Jag1*$^{Ndr/Ndr}$ mice exhibit anywhere between 0 and 5 crossings per retina (compared to 0-1 in wild type), and 3-6 arterioles per retina (6-7 in wild type). We propose that the more severe phenotypes, whether highly penetrant or sporadically present are the most likely to contribute to bleeds, for example extremes of the AV crossings, fewest blood vessels, or most divergent MCA architecture.

For clarification, we have omitted the sentence "However, sporadic vascular events in Jag1Ndr/Ndr mice may also contribute to, or be correlated with, the sporadic bleeds " and expanded the explanation in a whole paragraph starting "This study shows that *Jag1*$^{Ndr/Ndr}$ mice exhibit a spectrum of vascular phenotypes that differ both in frequency (ubiquitous, sex-dependent, sporadic) and severity…."

To further clarify, the following text was amended: "There are thus both statistically significant highly penetrant and sex-specific differences in *Jag1*-abrogated vasculature, as well as sporadic events in both sexes. Which phenotype, or phenotype severity, correlates with bleeding events would be important to test in future studies."

Discussion, "These vascular events were sporadic and could not be statistically evaluated". Please rephrase, the number of mice analyzed in this study was too small to conduct statistical analysis.

Thank you for pointing out this sentence. Because the sentence is referring to sporadic events across different experiments (different assays, different organs etc), it would not be correct to merge and statistically assess the data. We apologize if this was unclear. We have opted to remove the sentence to avoid confusion.

Discussion, "sporadic sex-specific events" What do you mean, please clarify

Thank you again for noting this phrase, this phrase was unclear/incorrect. We amended the sentence to: "There are thus both statistically significant sex-specific differences in *Jag1*-abrogated vasculature, as well as sporadic events in both sexes."

Discussion, lines 428-431 "The retinal vascular abnormalities detected in ALGS retinographs were not present in CADASIL, suggesting vascular tortuosity (identified in both ALGS and the mouse model) arises from EC (JAG1) defects rather than being secondary to VSMC (JAG1-NOTCH3) defects". I unfortunately believe that this interpretation is erroneous. The finding that CADASIL patients do not exhibit retinal vascular abnormalities detected in ALGS retinographs may be due to the fact that CADASIL mutations are not loss of function mutations, which is a very likely possibility. It might have been wiser to compare the phenotype of Jagged 1 mutant mice and Notch3KO mice.

In this experiment and figure we were asking whether patients with Alagille syndrome or CADASIL exhibit similar retinal pathology. Vascular defects in CADASIL retinas had already been reported, with increased vascular diameter in patients (Alten et al PLoS One 2014). Intriguingly, this report also showed a retinograph from a patient with CADASIL, with very tortuous blood vessels (their Figure 3B). We therefore expected to find increased tortuosity in CADASIL retinas. However, we agree that the majority of CADASIL NOTCH3 mutations are not NOTCH3 loss of function, and have amended the sentence as follows: "The retinal vascular abnormalities detected in ALGS retinographs were not present in CADASIL, suggesting vascular tortuosity is specific to ALGS."

We agree that it would be very interesting to analyze Notch3Ko mice to resolve the contribution of Notch3 signaling to blood vessel tortuosity.

Discussion, page 17, lines 439-440, "vessel tortuosity........... is associated with ischemic stroke". This statement should not be taken for granted since it has been reported in a single paper. It would be fairer to delete this sentence, especially as the present paper deals with brain hemorrhage and not ischemic stroke.

The referenced paper is a case-control study investigating 557 ischemic stroke cases and 557 controls, specifically assessing whether retinal vascular tortuosity correlates with ischemic stroke. They find that both arterial and venous tortuosity correlate with ischemic stroke. There are additional references that can be included, for example the following one which investigated 1185 participants and found that retinal venous tortuosity was associated with cerebral infarcts and stroke:

1. Hughes AD, Falaschetti E, Witt N, Wijetunge S, Thom SA, Tillin T, Aldington SJ, Chaturvedi N. Association of Retinopathy and Retinal Microvascular Abnormalities With Stroke and Cerebrovascular Disease. Stroke. 2016 Nov;47(11):2862-2864. doi: 10.1161/STROKEAHA.116.014998. Epub 2016 Oct 11. PMID: 27729577; PMCID: PMC5082730.

Although we focused on bleeds in this manuscript, we also show (in Appendix Fig S1B and Table S1) that patients with Alagille syndrome present with ischemic events. These could also have an underlying vascular cause, and may be related to the venous tortuosity, and thus deserve mention in the Discussion. To be clearer, we have amended this section as follows:

"Retinal vessel tortuosity, observed both in patients with ALGS and *Jag1*$^{Ndr/Ndr}$ mice, is an indicator of vessel wall dysfunction (Rim *et al*, 2020) and is associated with ischemic stroke (Hughes *et al*, 2016; Ong *et al*, 2013). Furthermore, retinal vascular changes are correlated with cerebral small vessel disease (Kwa *et al*, 2002) and with other abnormal vessels in the body (Kim & Fulton, 2007). Although we focused on bleeding events in this report, it is worth noting that ischemic events have also been reported in patients with ALGS (Appendix Fig S1B and Table S1)."

5th Oct 2022

Dear Dr. Andersson,

Thank you for providing the revised files. I am pleased to inform you that your manuscript is now accepted for publication in EMBO Molecular Medicine!

Before we can transfer your manuscript to our publisher, could you please address the following:

1/ I slightly modified your synopsis, please let me know if you agree with the following:
Spontaneous bleeds are a significant cause of death in the rare genetic disease Alagille syndrome, but little is known about the risk factors contributing to the bleeding events, or about the vasculature development and maintenance in the course of the disease.
- Alagille syndrome is modeled by Jag1Ndr/Ndr mice, including high mortality and sporadic bleeding.
- More female than male patients with Alagille syndrome are reported with intracranial bleeds, and certain vascular phenotypes are more severe in female Jag1Ndr/Ndr mice.
- Bleeding risk in Jag1Ndr/Ndr mice may be further modified by thin skull bones, fragile vascular smooth muscle cells, premature vascular aging and increased venous tortuosity.
- Developmental and homeostatic vascular defects were detected in both endothelial cells and vascular smooth muscle cells in Jag1Ndr/Ndr mice.
- The vascular defects observed in Jag1Ndr/Ndr mice could be visualized, quantified, and validated in retinographs from patients, suggesting a non-invasive method to assess vascular health.

2/ Figure 1 and Appendix Figure 1 Source data: Would it be correct to split this file into 2 files, i.e. Figure 1 Source Data and Appendix Figure 1 Source Data? Do you want to keep the word document in the Source Data file?

Congratulations on your interesting work!

With kind regards,

Lise Roth

Lise Roth, Ph.D
Senior Editor
EMBO Molecular Medicine

EMBO Press Author Checklist

Corresponding Author Name: Emma R Andersson	
Journal Submitted to: EMBO Molecular Medicine	
Manuscript Number: EMM-2022-15809	

USEFUL LINKS FOR COMPLETING THIS FORM
The EMBO Journal - Author Guidelines
EMBO Reports - Author Guidelines
Molecular Systems Biology - Author Guidelines
EMBO Molecular Medicine - Author Guidelines

Reporting Checklist for Life Science Articles (updated January 2022)
This checklist is adapted from Materials Design Analysis Reporting (MDAR) Checklist for Authors. MDAR establishes a minimum set of requirements in transparent reporting in the life sciences (see Statement of Task: 10.31222/osf.io/9sm4x). Please follow the journal's guidelines in preparing your manuscript.
Please note that a copy of this checklist will be published alongside your article.

Abridged guidelines for figures
1. Data
The data shown in figures should satisfy the following conditions:
- the data were obtained and processed according to the field's best practice and are presented to reflect the results of the experiments in an accurate and unbiased manner.
- ideally, figure panels should include only measurements that are directly comparable to each other and obtained with the same assay.
- plots include clearly labeled error bars for independent experiments and sample sizes. Unless justified, error bars should not be shown for technical replicates.
- if n<5, the individual data points from each experiment should be plotted. Any statistical test employed should be justified.
- Source Data should be included to report the data underlying figures according to the guidelines set out in the authorship guidelines on Data Presentation.

2. Captions
Each figure caption should contain the following information, for each panel where they are relevant:
- a specification of the experimental system investigated (eg cell line, species name).
- the assay(s) and method(s) used to carry out the reported observations and measurements.
- an explicit mention of the biological and chemical entity(ies) that are being measured.
- an explicit mention of the biological and chemical entity(ies) that are altered/varied/perturbed in a controlled manner.
- the exact sample size (n) for each experimental group/condition, given as a number, not a range;
- a description of the sample collection allowing the reader to understand whether the samples represent technical or biological replicates (including how many animals, litters, cultures, etc.).
- a statement of how many times the experiment shown was independently replicated in the laboratory.
- definitions of statistical methods and measures:
  - common tests, such as t-test (please specify whether paired vs. unpaired), simple χ2 tests, Wilcoxon and Mann-Whitney tests, can be unambiguously identified by name only, but more complex techniques should be described in the methods section;
  - are tests one-sided or two-sided?
  - are there adjustments for multiple comparisons?
  - exact statistical test results, e.g., P values = x but not P values < x;
  - definition of 'center values' as median or average;
  - definition of error bars as s.d. or s.e.m.

Please complete ALL of the questions below.
Select "Not Applicable" only when the requested information is not relevant for your study.

Materials

Newly Created Materials	Information included in the manuscript?	In which section is the information available? (Reagents and Tools Table, Materials and Methods, Figures, Data Availability Section)
New materials and reagents need to be available; do any restrictions apply?	Not Applicable	

Antibodies	Information included in the manuscript?	In which section is the information available? (Reagents and Tools Table, Materials and Methods, Figures, Data Availability Section)
For **antibodies** provide the following information:<br>- Commercial antibodies: RRID (if possible) or supplier name, catalogue number and or/clone number<br>- Non-commercial: RRID or citation	Yes	Material and Methods

DNA and RNA sequences	Information included in the manuscript?	In which section is the information available? (Reagents and Tools Table, Materials and Methods, Figures, Data Availability Section)
**Short novel DNA or RNA including primers, probes**: provide the sequences.	Not Applicable	

Cell materials	Information included in the manuscript?	In which section is the information available? (Reagents and Tools Table, Materials and Methods, Figures, Data Availability Section)
**Cell lines:** Provide species information, strain. Provide accession number in repository **OR** supplier name, catalog number, clone number, and/**OR** RRID.	Not Applicable	
**Primary cultures:** Provide species, strain, sex of origin, genetic modification status.	Not Applicable	
Report if the cell lines were recently **authenticated** (e.g., by STR profiling) and tested for mycoplasma contamination.	Not Applicable	

Experimental animals	Information included in the manuscript?	In which section is the information available? (Reagents and Tools Table, Materials and Methods, Figures, Data Availability Section)
**Laboratory animals or Model organisms:** Provide species, strain, sex, age, genetic modification status. Provide accession number in repository **OR** supplier name, catalog number, clone number, **OR** RRID.	Yes	Material and Methods
**Animal observed in or captured from the field:** Provide species, sex, and age where possible.	Not Applicable	
Please detail **housing and husbandry conditions**.	Yes	Material and Methods

Plants and microbes	Information included in the manuscript?	In which section is the information available? (Reagents and Tools Table, Materials and Methods, Figures, Data Availability Section)
**Plants:** provide species and strain, ecotype and cultivar where relevant, unique accession number if available, and source (including location for collected wild specimens).	Not Applicable	
**Microbes:** provide species and strain, unique accession number if available, and source.	Not Applicable	

Human research participants	Information included in the manuscript?	In which section is the information available? (Reagents and Tools Table, Materials and Methods, Figures, Data Availability Section)
If collected and within the bounds of privacy constraints report on age, sex and gender or ethnicity for all study participants.	Yes	Material and Methods

Core facilities	Information included in the manuscript?	In which section is the information available? (Reagents and Tools Table, Materials and Methods, Figures, Data Availability Section)
If your work benefited from core facilities, was their service mentioned in the acknowledgments section?	Yes	Ackwnoledgments

Design

Study protocol	Information included in the manuscript?	In which section is the information available? (Reagents and Tools Table, Materials and Methods, Figures, Data Availability Section)
If study protocol has been **pre-registered, provide DOI in the manuscript**. For clinical trials, provide the trial registration number **OR** cite DOI.	Not Applicable	
Report the **clinical trial registration number** (at ClinicalTrials.gov or equivalent), where applicable.	Not Applicable	

Laboratory protocol	Information included in the manuscript?	In which section is the information available? (Reagents and Tools Table, Materials and Methods, Figures, Data Availability Section)
Provide DOI OR other citation details if **external detailed step-by-step protocols** are available.	Not Applicable	

Experimental study design and statistics	Information included in the manuscript?	In which section is the information available? (Reagents and Tools Table, Materials and Methods, Figures, Data Availability Section)
Include a statement about **sample size** estimate even if no statistical methods were used.	Yes	Material and Methods
Were any steps taken to minimize the effects of subjective bias when allocating animals/samples to treatment (e.g. **randomization procedure**)? If yes, have they been described?	Yes	Material and Methods
Include a statement about **blinding** even if no blinding was done.	Yes	Material and Methods
Describe **inclusion/exclusion criteria** if samples or animals were excluded from the analysis. Were the criteria pre-established?<br><br>If sample or data points were omitted from analysis, report if this was due to attrition or intentional exclusion and provide justification.	Yes	Material and Methods
For every figure, are **statistical tests** justified as appropriate? Do the data meet the assumptions of the tests (e.g., normal distribution)? Describe any methods used to assess it. Is there an estimate of variation within each group of data? Is the variance similar between the groups that are being statistically compared?	Yes	Material and Methods

Sample definition and in-laboratory replication	Information included in the manuscript?	In which section is the information available? (Reagents and Tools Table, Materials and Methods, Figures, Data Availability Section)
In the figure legends: state number of times the experiment was **replicated** in laboratory.	Yes	Material and Methods
In the figure legends: define whether data describe **technical or biological replicates**.	Yes	Figure legend

Ethics

Ethics	Information included in the manuscript?	In which section is the information available? (Reagents and Tools Table, Materials and Methods, Figures, Data Availability Section)
Studies involving **human participants**: State details of **authority granting ethics approval** (IRB or equivalent committee(s), provide reference number for approval.	Yes	Material and Methods
Studies involving **human participants**: Include a statement confirming that **informed consent** was obtained from all subjects and that the experiments conformed to the principles set out in the WMA Declaration of Helsinki and the Department of Health and Human Services Belmont Report.	Yes	Material and Methods
Studies involving **human participants:** For publication of **patient photos**, include a statement confirming that consent to publish was obtained.	Yes	Material and Methods
Studies involving experimental **animals**: State details of **authority granting ethics approval** (IRB or equivalent committee(s), provide reference number for approval. Include a statement of compliance with ethical regulations.	Yes	Material and Methods
Studies involving **specimen and field samples:** State if relevant **permits** obtained, provide details of authority approving study; if none were required, explain why.	Not Applicable	

Dual Use Research of Concern (DURC)	Information included in the manuscript?	In which section is the information available? (Reagents and Tools Table, Materials and Methods, Figures, Data Availability Section)
Could your study fall under dual use research restrictions? Please check biosecurity documents and list of **select agents and toxins** (CDC): https://www.selectagents.gov/sat/list.htm	Not Applicable	
If you used a select agent, is the security level of the lab appropriate and reported in the manuscript?	Not Applicable	
If a study is subject to dual use research of concern regulations, is the name of the **authority granting approval and reference number** for the regulatory approval provided in the manuscript?	Not Applicable	

Reporting

The MDAR framework recommends adoption of discipline-specific guidelines, established and endorsed through community initiatives. Journals have their own policy about requiring specific guidelines and recommendations to complement MDAR.

Adherence to community standards	Information included in the manuscript?	In which section is the information available? (Reagents and Tools Table, Materials and Methods, Figures, Data Availability Section)
State if relevant guidelines or checklists (e.g., **ICMJE, MIBBI, ARRIVE, PRISMA**) have been followed or provided.	Yes	Source Data 1, Appendix Material and Methods
For **tumor marker prognostic studies,** we recommend that you follow the **REMARK** reporting guidelines (see link list at top right). See author guidelines, under 'Reporting Guidelines'. Please confirm you have followed these guidelines.	Not Applicable	
For **phase II and III randomized controlled trials**, please refer to the **CONSORT** flow diagram (see link list at top right) and submit the CONSORT checklist (see link list at top right) with your submission. See author guidelines, under 'Reporting Guidelines'. Please confirm you have submitted this list.	Not Applicable	

Data Availability

Data availability	Information included in the manuscript?	In which section is the information available? (Reagents and Tools Table, Materials and Methods, Figures, Data Availability Section)
Have **primary datasets** been deposited according to the journal's guidelines (see 'Data Deposition' section) and the respective accession numbers provided in the Data Availability Section?	Yes	Dava Availability
Were **human clinical and genomic datasets** deposited in a public access-controlled repository in accordance to ethical obligations to the patients and to the applicable consent agreement?	Not Applicable	
Are **computational models** that are central and integral to a study available without restrictions in a machine-readable form? Were the relevant accession numbers or links provided?	Not Applicable	
If publicly available data were reused, provide the respective **data citations in the reference list**.	Not Applicable	
